# Ternary Complex Modeling, Induced Fit Docking and Molecular Dynamics Simulations as a Successful Approach for the Design of VHL‐Mediated PROTACs Targeting the Kinase FLT3

**DOI:** 10.1002/ardp.202500102

**Published:** 2025-04-14

**Authors:** Husam Nassar, Anne‐Christin Sarnow, Ismail Celik, Mohamed Abdelsalam, Dina Robaa, Wolfgang Sippl

**Affiliations:** ^1^ Department of Medicinal Chemistry, Institute of Pharmacy Martin‐Luther University Halle‐Wittenberg Halle (Saale) Germany; ^2^ Department of Pharmaceutical Chemistry, Faculty of Pharmacy Erciyes University Kayseri Turkey; ^3^ Department of Pharmaceutical Chemistry, Faculty of Pharmacy Alexandria University Alexandria Egypt

**Keywords:** FLT3, induced‐fit docking, MD simulation, PROTACs, VHL

## Abstract

Proteolysis targeting chimeras (PROTACs) have proven to be a novel approach for the degradation of disease‐causing proteins in drug discovery. One of the E3 ligases for which efficient PROTACs have been described is the Von Hippel‐Lindau factor (VHL). However, the development of PROTACs has so far often relied on a minimum of computational tools, so that it is mostly based on a trial‐and‐error process. Therefore, there is a great need for resource‐ and time‐efficient structure‐based or computational approaches to streamline PROTAC design. In this study, we present a combined computational approach that integrates static ternary complex formation, induced‐fit docking, and molecular dynamics (MD) simulations. Our methodology was tested using four experimentally derived ternary complex structures of VHL PROTACs, reported for BRD4, SMARCA2, FAK, and WEE1. In addition, we applied the validated approach to model a recently in‐house developed FLT3‐targeted PROTAC (MA49). The results show that static ternary models generated with a protein–protein docking method implemented in the software MOE have a high predictive power for reproducing the experimental 3D structures. The induced‐fit docking of different active PROTACs to their respective models showed the reliability of this model for the development of new VHL‐mediated degraders. In particular, the induced‐fit docking was sensitive to structural changes in the PROTACs, as evidenced by the failed binding modes of the PROTAC negative controls. Furthermore, MD simulations confirmed the stability of the generated complexes and emphasized the importance of dynamic studies for understanding the relationship between PROTAC structure and function.

## Graphical abstract

An efficient computational approach is presented for designing VHL‐mediated PROTACs by integrating static modeling, induced‐fit docking, and MD simulations. After being validated on four experimental ternary complexes targeting BRD4, SMARCA2, FAK, and WEE1, it was applied to an FLT3‐targeted PROTAC, MA49. MD simulations confirmed stability and provided insights into the structure–function relationship.

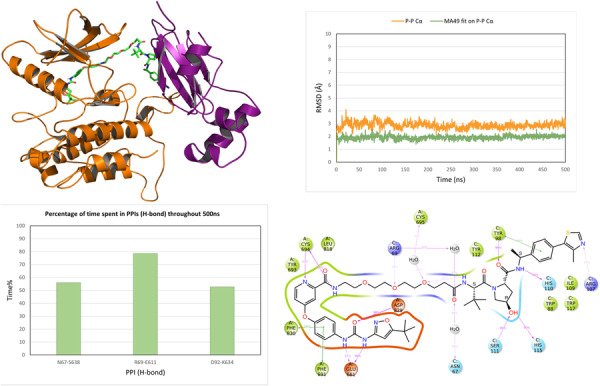

## 1 Introduction

Proteolysis targeting chimeras (PROTACs) are heterobifunctional molecules designed to degrade proteins in cells by leveraging the cell's intrinsic protein degradation machinery. These molecules consist of three main components: a ligand that binds to the targeted protein, a ligand that binds to an E3 ubiquitin ligase, and a linker that connects the two moieties [1]. Once the PROTAC binds to both the target protein and the E3 ligase, the target protein is brought into proximity with the ubiquitination machinery, allowing the E2 ligase subunit to ubiquitinate the protein, marking it for degradation by the proteasome. This process leads to the complete elimination of the target protein, rather than merely inhibiting its activity [2, 3]. The significance of PROTACs in modern drug discovery lies in their ability to selectively degrade disease‐causing proteins at low concentrations. The degradation is offering a more sustained and complete reduction in protein activity. PROTACs can also target mutant proteins that evade natural degradation, expanding therapeutic possibilities for challenging diseases [4].

More than 600 E3 ligases have been characterized in the human genome, with Cereblon (CRBN) and Von Hippel‐Lindau (VHL) being the most frequently utilized in PROTAC development to date. Other less commonly utilized E3 ligases include Inhibitor of Apoptosis Proteins (IAPs), DDB1 and Cul4 Associated Factor 15 (DCAF15), and Mouse Double Minute 2 (MDM2). In this study, we focus on PROTACs that employ VHL ligands, which represent the second most commonly used class of E3 ligase recruiters after CRBN ligands [5, 6, 7, 8]. The prominence of VHL‐based mechanisms in PROTAC development is likely ascribed to the availability of small molecules that bind to VHL with high selectivity and potency. These molecules have a well‐defined structure–activity relationship and exhibit favorable physicochemical characteristics [9, 10, 11, 12]. A recent review article categorized VHL‐based PROTACs by disease area and target class, highlighting over 40 proteins, including previously undruggable targets, that can be effectively degraded using VHL‐mediated PROTACs [13].

The degradation of target proteins involves distinct phases and structures before successful ubiquitination, making the application of computational methods to rationalize PROTAC design a complex task [14, 15]. The propensity for a ternary complex of target protein, PROTAC, and VHL to form is the first step that must be analyzed in the structural modeling of PROTAC‐mediated degradation [16]. Additionally, the strength of protein–protein interactions (PPIs) as well as the spatial orientation and accessibility of lysine residues on the target protein, which are to be ubiquitinated, are essential properties that should be analyzed to assess the stability and productivity of the generated ternary complex models [17, 18].

Various modeling techniques have been developed to facilitate PROTAC design by predicting the protein–protein and PROTAC linker conformations within ternary complex models. These techniques have been validated using experimentally derived ternary complex structures. Such predictions serve as a foundation for more detailed analyses, including evaluating the accessibility of the binding pocket conformations for different PROTACs and optimizing the PROTACs themselves [19, 20, 21]. Although static models of ternary complexes can help draft new active PROTACs, they might be insufficient to link structure to function due to the considerable flexibility of these structures [22]. The structure and length of the linker motif of PROTACs play a crucial role in ternary complex dynamics in solution and significantly affect its conformational change and subsequently productivity [23, 24]. Therefore, molecular dynamics (MD) simulations are crucial in many aspects of the modeling of target protein degradation and the design of PROTACs. By capturing atomic‐level movements, MD simulations can provide insights into the stability, flexibility, and conformational changes of ternary complexes, which are necessary for understanding their structure‐to‐function relationship.

While increased rigidity of a PROTAC in the ternary complex does not always result in improved degradation efficiency—as a highly stable complex may hinder a PROTAC's catalytic turnover rate—an overly dynamic complex can reduce the likelihood of successful ubiquitination [25, 26]. Therefore, achieving an optimal level of ternary complex stability is crucial for effective degradation. This can be achieved by optimally adapting the linkers of the PROTACs. Moreover, strong PPIs could compensate for weak PROTAC affinity and drive degradation potency. Therefore, a PROTAC's ability to induce a stable ternary complex is the driving force behind its activity rather than the PROTAC's affinity for either of the monomers [27]. Among PPIs, salt bridges play a key role in stabilizing ternary complexes by promoting positive cooperativity, extending the half‐life, and ensuring long residence times with a low dissociation rate, which in turn supports efficient ubiquitination [28, 29].

**Figure 1:**
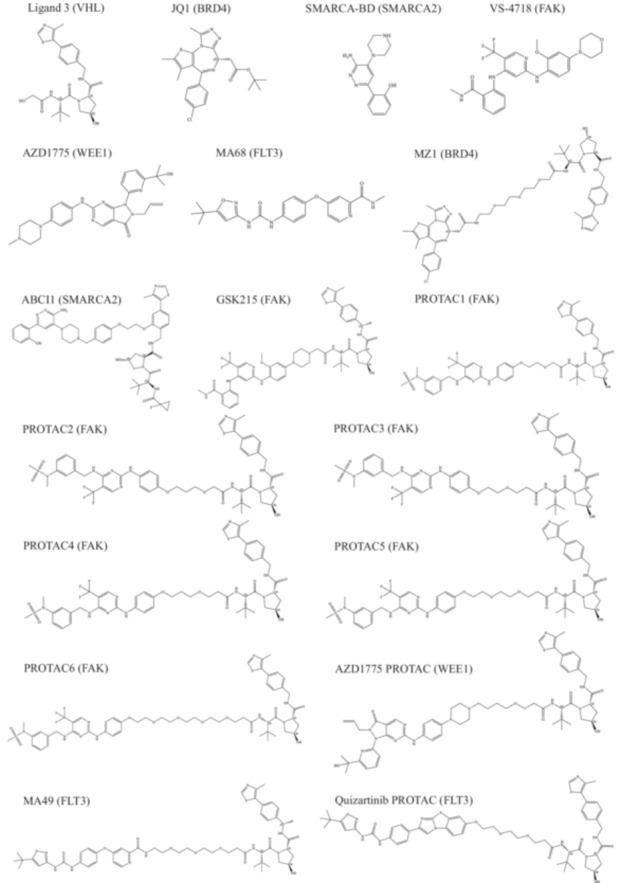
2D structures and names of ligands and PROTACs included in the study. Target names are shown in brackets.

In the current work, we describe the development of a modeling approach for VHL‐mediated PROTACs, which was retrospectively validated. The 2D structures of the studied PROTACs are depicted in Figure 1. Initially, two protein–protein docking approaches—Method4B in MOE and PRosettaC—were utilized [30, 31]. In both methods, a conformational ensemble of the PROTAC is generated and aligned on the bound ligands in the predicted protein–protein docking solutions to construct ternary complex models. These models were validated based on their ability to reproduce available ternary complex 3D structures, including the root mean square deviation (RMSD) of the proteins’ Cα atoms and the PROTAC atoms, as well as the reproduction of relevant PPIs and ubiquitination accessibility. MD simulations demonstrated the stability of the experimentally derived 3D structures of ternary complexes and were subsequently used to test the stability of the generated models. We then tested the generated models to evaluate whether various PROTACs could be accurately modeled through induced fit docking and MD simulations.

The validated modeling method was subsequently applied to another target protein, the mutated kinase FLT3‐ITD, for which no ternary complex structure is available so far. For this target, we used a data set comprising both in‐house developed and published VHL‐based PROTACs [32, 33]. The presented modeling strategy offers a resource‐efficient and time‐saving approach for developing effective PROTACs in realistic scenarios where 3D structures of ternary complexes are unavailable. The generated ternary complex structures can serve as templates for designing optimized degrader molecules by performing large‐scale induced fit docking screenings with in silico‐generated PROTAC candidates.

## 2 Results

### 2.1 Preparation of FAK– and FLT3–Inhibitor Complexes

Glide SP docking was performed to generate FAK–VS‐4718 and FLT3–MA68 complex monomers for use in ternary complex modeling. The docking protocol was validated by assessing its ability to reproduce the binding modes of the cocrystallized FAK‐bound inhibitor, BI‐4464 (PDB ID 6I8Z), and the cocrystallized FLT3‐bound inhibitor, quizartinib (PDB ID 4XUF). FLT3 structure from PDB 4XUF has a long missing loop (residues 708 to 782). This FLT3 structure is crystallized with quizartinib in the DFG‐out conformation, which is necessary to accommodate type 2 inhibitors such as MA68. As no PDB file contains this loop in a crystallized form, it was modeled using the SWISS‐MODEL web tool before docking the FLT3 ligands.

**Figure 2:**
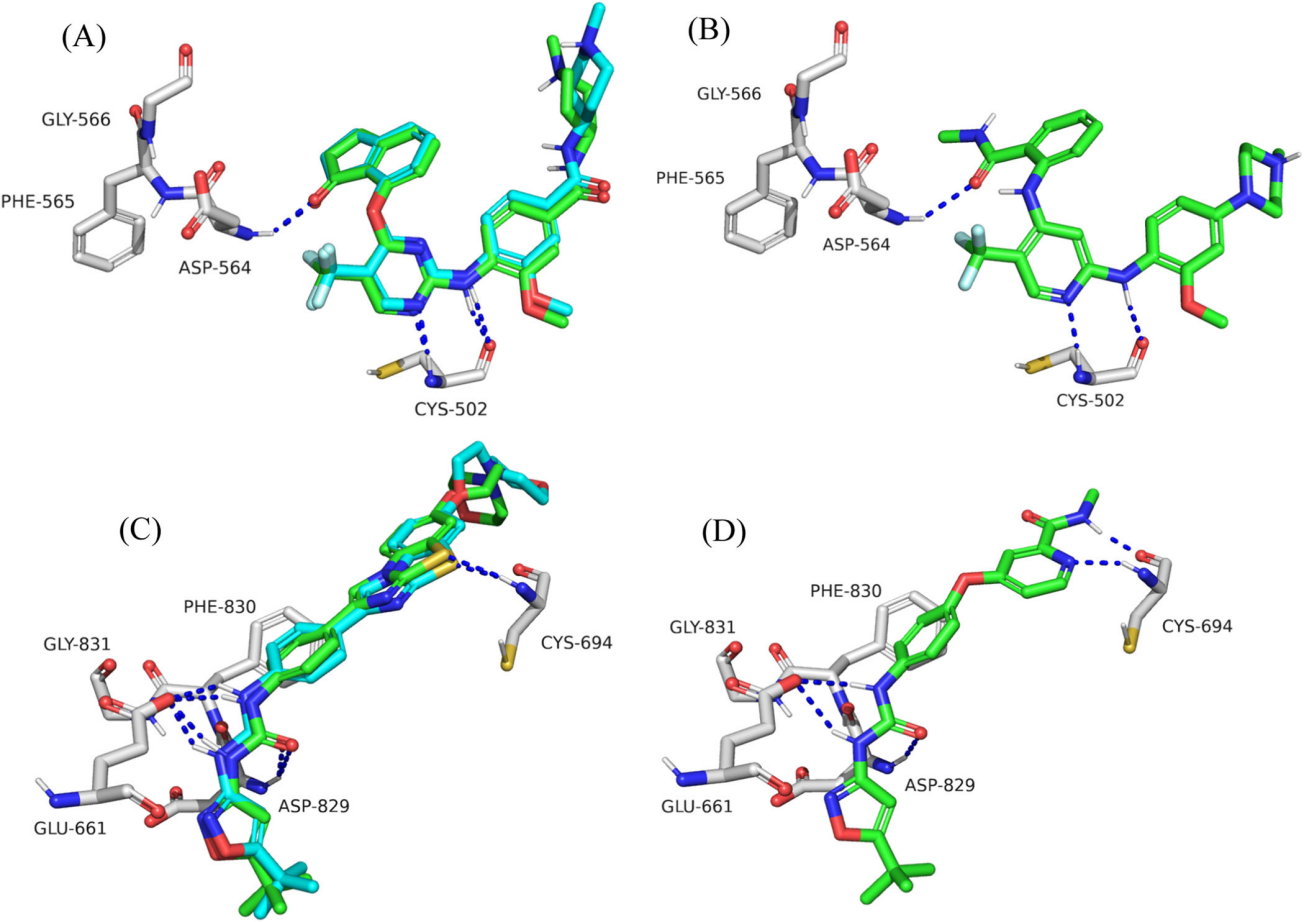
Detailed view of the docking results within FAK and FLT3 ATP binding pockets. (A) BI‐4464 docked pose (green) superposed on its crystallographic pose (cyan, PDB ID 6I8Z) within FAK binding pocket. (B) Interactions of docked VS‐4718 with FAK amino acid residues. (C) Quizartinib docked pose (green) superposed on its crystallographic pose (cyan, PDB ID 4XUF) within FLT3 binding pocket. (D) Interactions of docked MA68 with FLT3 amino acid residues. Hydrogen bonds are shown as blue dashed lines.

The docked poses closely matched the crystallographic data, with RMSD values of 0.87 Å for docked BI‐4464 and 0.94 Å for docked quizartinib (Figure 2A,C). The obtained VS‐4718 docked pose closely resembled its binding mode observed in GSK215 PROTAC inside the FAK binding site. The hydrogen bond donor/acceptor sequence of the aminopyridine moiety was projected toward the hinge Cys502 residue forming two hydrogen bonds. Moreover, the amide oxygen showed a hydrogen bond to Asp564 in the DFG‐in loop (Figure 2B). On the other hand, the MA68 docked pose demonstrated the common type 2 inhibitor binding mode within the binding pocket of FLT3. The *N*‐methyl‐picolinamide moiety of MA68 was oriented toward the hinge region near the solvent‐exposed area, forming two hydrogen bonds with Cys694. The urea oxygen and phenyl ring were directed toward the DFG‐out motif, where they formed a hydrogen bond and π–stacking interactions with Asp829 and Phe830, respectively (Figure 2D).

To further assess the stability of the MA68 pose and evaluate the dynamics of modeled loop residues at positions 708‐782, the FLT3–MA68 complex was simulated for 500 ns. The MA68 binding mode remained stable, while the modeled loop was found to be flexible (Supporting Information S1: Figure S1). Consequently, it was deemed appropriate to exclude its contribution to RMSD values in the MD simulation studies as depicted in Supporting Information S1: Figure S2. FAK with docked VS‐4718 and FLT3 with docked MA68 were then saved as PDB files for use as inputs in ternary complex generation, alongside the crystallized structures of BRD4BD2 (PDB ID 5T35), SMARCA2 (PDB ID 7S4E), WEE1 (PDB ID 5V5Y), and VHL (PDB ID 5NVV) in complex with their cognate ligands.

### 2.2 Validation of Ternary Complex Modeling Protocols

**Figure 3:**
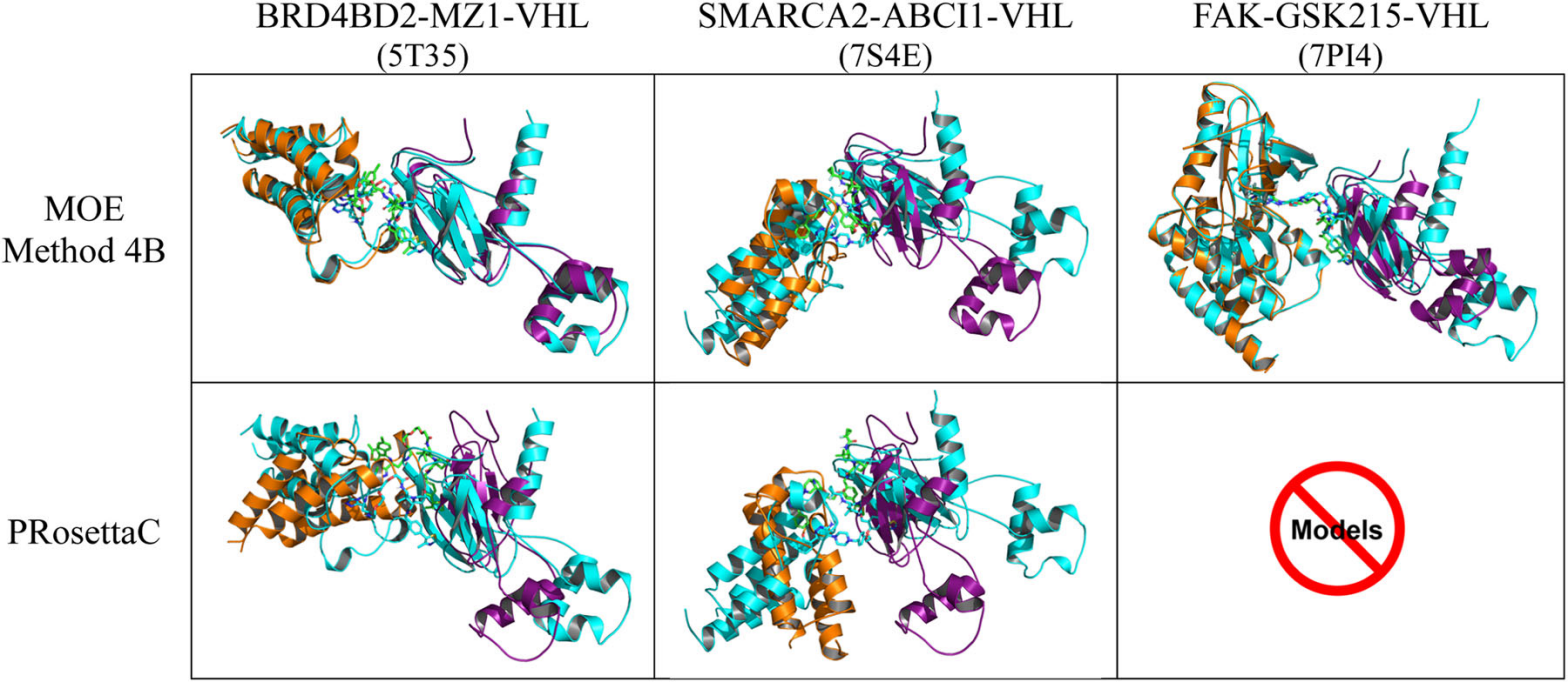
Top scored models of the most populated clusters generated by MOE Method 4B and PRosettaC, with the target protein shown in orange and VHL in purple, superposed on the crystal structures (cyan). MOE Method 4B models for BRD4BD2‐MZ1‐VHL (PDB ID 5T35), SMARCA2‐ABCI1‐VHL (PDB ID 7S4E), and FAK‐GSK215‐VHL (PDB ID 7PI4) are shown in the upper row, while PRosettaC models for PDB 5T35 and PDB 7S4E are presented in the lower row.

We started by validating the ability of MOE Method 4B and PRosettaC to reproduce the experimental structures of the ternary complex conformations of BRD4BD2‐MZ1‐VHL (PDB ID 5T35), SMARCA2‐ABCI1‐VHL (PDB ID 7S4E), and FAK‐GSK215‐VHL (PDB ID 7PI4). The primary criteria for evaluation were Cα RMSD, PROTAC RMSD, PPIs, and ubiquitination accessibility. For each protocol, the top three models from the most populated cluster were selected, minimized to resolve steric clashes between the PROTACs and protein side chains and then compared with the experimental 3D structures. As shown in Figure 3, MOE Method 4B outperformed PRosettaC in closely resembling the experimentally derived complexes. For the top three models of PDB 5T35 (BRD4BD2) and 7PI4 (FAK), the Cα RMSD values ranged between 1.50 and 5.21 Å, while the PROTAC RMSD values fell within the 2.12–4.82 Å range. Similarly, for PDB 7S4E (SMARCA2), the top three models showed Cα and PROTAC RMSD values of 4.51–5.54 Å (Table 1). Interacting residues observed in the 3D structures of PDB 5T35 (BRD4BD2) and 7PI4 (FAK) were projected toward each other in their minimized, modeled structures, with donor–acceptor distances ranging between 3 and 6 Å.

**Table 1:** Cα RMSD, PROTAC RMSD, and P‐P docking scores (S) calculated for the top three models obtained by MOE Method 4B.

Model	BRD4BD2‐MZ1‐VHL (5T35)			SMARCA2‐ABCI1‐VHL (7S4E)			FAK‐GSK215‐VHL (7PI4)		
	P‐P Cα RMSD	PROTAC RMSD	P‐P docking score (S)	P‐P Cα RMSD	PROTAC RMSD	P‐P docking score (S)	P‐P Cα RMSD	PROTAC RMSD	P‐P docking score (S)
Model 1	1.50	2.99	–33.35	5.01	5.26	–44.15	4.96	3.50	–43.43
Model 2	2.48	3.29	–32.13	4.82	5.14	–40.69	3.05	2.97	–38.09
Model 3	1.84	2.12	–31.08	4.51	5.54	–39.14	5.21	4.82	–35.33

In the case of the modeled PDB 7S4E (SMARCA2), donor–acceptor distances between only two interacting residues of each monomer fell within a similar range, while the other residues showed greater distances. The MD simulation runs, described later, induced better juxtaposition of interacting residues immediately upon relaxation. All PPIs of the experimentally derived and modeled complexes are depicted in Supporting Information S1: Figure S3. In contrast, PRosettaC failed to predict native‐like conformations for both PDB 5T35 (BRD4BD2) and 7S4E (SMARCA2), producing models with Cα and PROTAC RMSD values exceeding 10 Å as depicted in Figure 3 and Table 2. Additionally, PRosettaC was unable to generate any model for PDB 7PI4 (FAK).

**Table 2:** Cα RMSD and PROTAC RMSD calculated for the top 3 models obtained by PRosettaC.

Model	BRD4BD2‐MZ1‐VHL (5T35)		SMARCA2‐ABCI1‐VHL (7S4E)		FAK‐GSK215‐VHL (7PI4)	
	P‐P Cα RMSD	PROTAC RMSD	P‐P Cα RMSD	PROTAC RMSD	P‐P Cα RMSD	PROTAC RMSD
Model 1	10.76	13.21	23.17	17.19	NA	
Model 2	11.52	13.11	22.62	18.35		
Model 3	11.54	13.02	23.02	17.23		

Given these results, the models produced by MOE Method 4B, particularly those with the best S‐scores (i.e., lowest protein–protein interaction energy), were selected for further analysis to avoid bias toward RMSD values. This was accomplished to resemble the real scenario where the 3D conformation is unknown. To assess ubiquitination accessibility, top‐scored models were aligned on the modeled ubiquitination machinery superimposed via VHL. The distances between Ser111 of the E2 ligase and the solvent‐accessible lysine residues on the targets were calculated in Pymol. The resulting models successfully reproduced the location of lysines to Ser111, with distances of 50–60 Å, consistent with the observed experimental 3D structures (Supporting Information S1: Figure S4).

**Figure 4:**
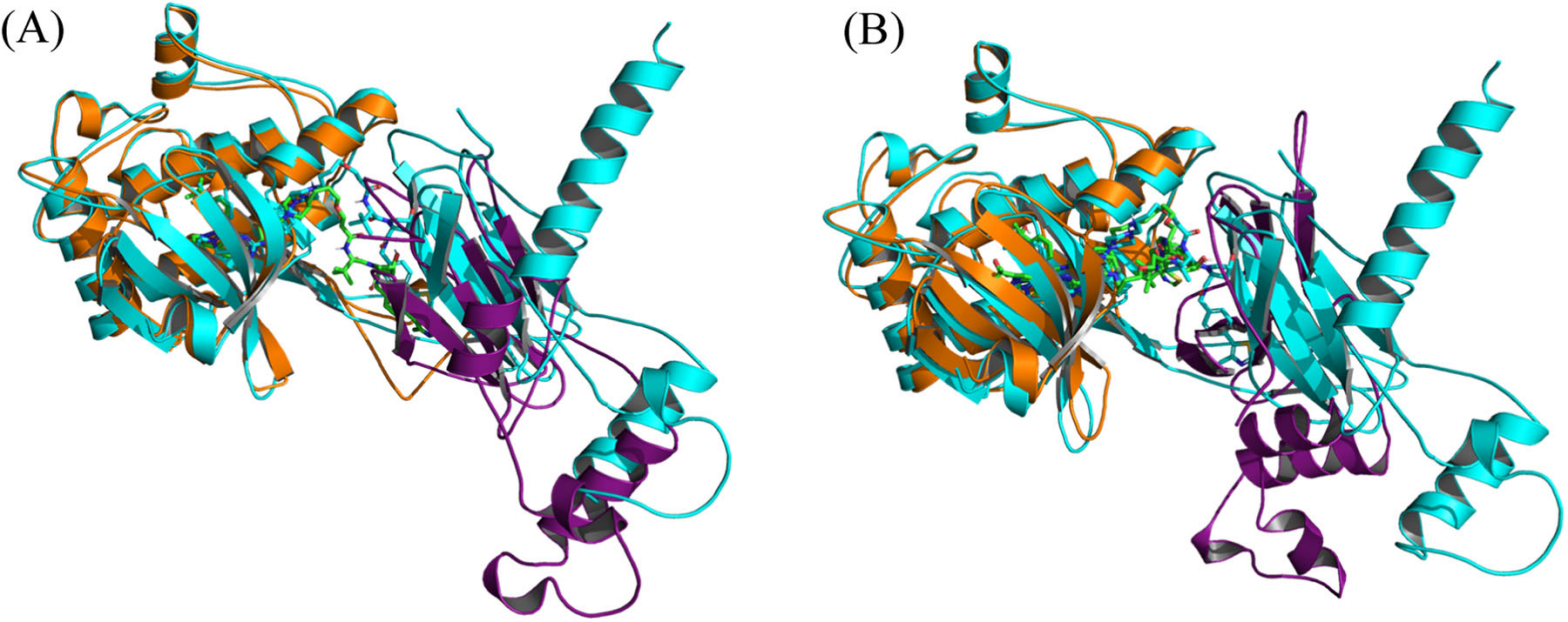
Top‐scored models generated by MOE Method 4B for the WEE1‐AZD1775 PROTAC‐VHL ternary complex (A) in the presence and (B) absence of the WEE1 loop of residues 438‐456.

**Figure 5:**
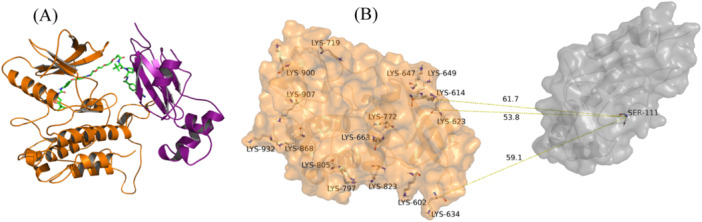
FLT3‐MA49‐VHL ternary complex. (A) Cartoon representation of the top‐scored model generated by MOE Method 4B. (B) Distances between solvent‐exposed lysines of FLT3 and Ser111 of the E2 ligase, indicated by yellow dashed lines.

MOE Method 4B was also chosen to model the WEE1‐AZD1775 PROTAC‐VHL ternary complex (PDB ID 8WDK). Two modeling runs, in the presence and the absence of the flexible WEE1 loop of residues 438‐456, were conducted to assess the impact of flexible kinase domain loops on the modeling results. The generated models and their RMSD values compared with the crystal structure are shown in Figure 4 and Table 3. The top model obtained for the retained WEE1 loop showed Cα RMSD of 5.81 Å with respect to the experimental structure while that obtained for the removed loop showed Cα RMSD of 9.88 Å with VHL protein pose partly occupying the site of the removed loop. Given that the FLT3 flexible loop (residues 708–782) is missing in its crystal structure, a similar risk of structural deviation and misrepresentation in protein–protein docking exists. To avoid this, it was important to model the FLT3 loop using SWISS‐MODEL before applying MOE Method 4B to predict the FLT3‐MA49‐VHL ternary complex structure. Similarly, the top models in the most populated double cluster were visually analyzed and the model with the best S score was selected (Figure 5A). The conformation of the generated model was also tested for its target ubiquitination accessibility. Figure 5B illustrates the three solvent‐exposed lysines (Lys614, Lys623, and Lys634) that were found to be accessible to ubiquitination. Eventually, the accessibility of FAK‐GSK215‐VHL and FLT3‐MA49‐VHL models to different active PROTACs as well as their stability were further evaluated using induced fit docking and MD simulations.

**Table 3:** Cα RMSD, PROTAC RMSD, and P‐P docking scores (S) calculated for the top three models of WEE1‐AZD1775 PROTAC‐VHL obtained by MOE Method 4B in the presence and absence of WEE1 flexible loop (residues 438‐456).

Model	WEE1 loop retained			WEE1 loop removed		
	P‐P Cα RMSD	PROTAC RMSD	P‐P docking score (S)	P‐P Cα RMSD	PROTAC RMSD	P‐P docking score (S)
Model 1	5.81	4.12	–61.62	9.88	7.05	–57.11
Model 2	4.10	2.17	–58.62	10.48	7.35	–54.44
Model 3	5.31	3.35	–56.49	9.42	7.22	–53.89

### 2.3 Induced‐Fit Docking of FAK and FLT3 PROTACs

**Figure 6:**
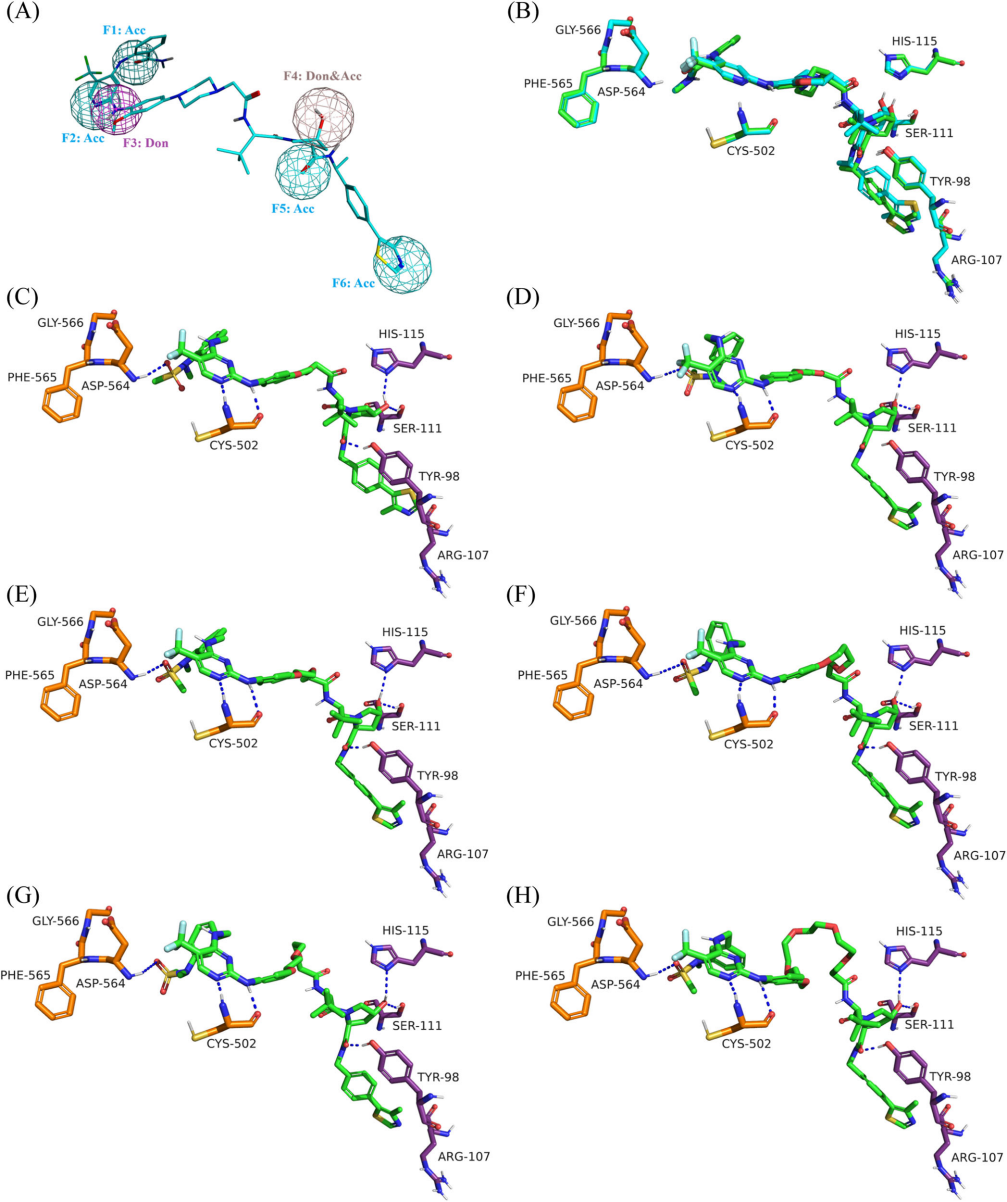
Detailed view of the docking results within the FAK‐GSK215‐VHL ternary model. (A) Modeled GSK215 pharmacophore features used to guide the placement of docked FAK PROTACs. (B) Superposition of the docked GSK215 pose (green) with the MOE Method 4B modeled GSK215 pose (cyan). (C–H) Interactions of PROTACs 1 through 6 with the amino acids in the FAK and VHL binding pockets. Hydrogen bonds (distance below 2.5 Å) are shown as blue dashed lines.

Induced‐fit docking was initially performed in the modeled FAK‐GSK215‐VHL (PDB ID 7PI4) ternary complex to assess its ability to accommodate different active PROTACs using MOE. Six, previously published, active VHL‐mediated degraders recruiting the FAK inhibitor, defactinib, were used in this analysis [34]. The docked poses were required to align with the pharmacophoric features assigned to the GSK215 pose in the modeled ternary complex structure as described in Section 5.6 and depicted in Figure 6A. As shown in Figure 6B–H, the docking results revealed that the modeled FAK‐GSK215‐VHL ternary conformation was capable of accommodating PROTACs with significantly varying linker lengths despite its difference from the crystal structure by Cα RMSD of 4.96 Å. The top‐ranked poses of these degraders demonstrated successful alignment with the assigned pharmacophore. The linkers in all PROTACs adopted conformations that were embedded at the interface between both proteins while preserving the main key interactions within each protein binding site of the modeled complex. Specifically, in the FAK binding pocket, the sulfoxide oxygen and aminopyridine ring of the FAK inhibitor (defactinib) formed hydrogen bonds with the DFG Asp564 and hinge Cys502, respectively. In the VHL ligand, the *R*‐hydroxyl group of the pyrrolidine ring formed two hydrogen bonds with Ser111 and His115, while the adjacent carbonyl oxygen was anchored to Tyr98. The aminothiazole nitrogen of all FAK PROTACs was oriented toward the Arg107 residue of VHL; however, it did not meet the distance and angle criteria necessary for hydrogen bond formation. This interaction was captured during MD simulations, where other key interactions—including those with the FAK hinge residue Cys502, the DFG motif residue Asp564, and VHL residues Ser111, His115, and Tyr98—remained stable.

**Figure 7:**
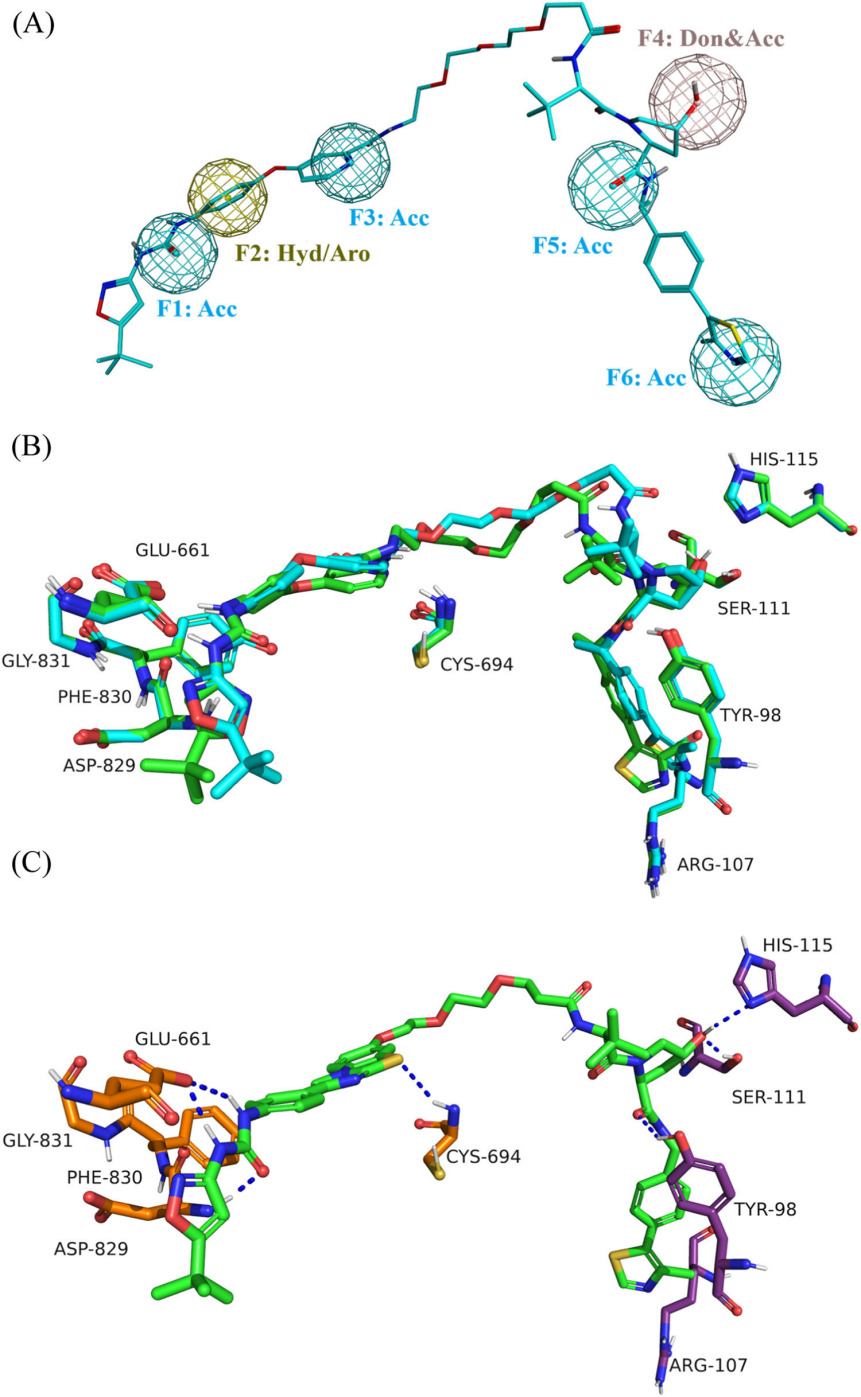
Detailed view of the docking results within the FLT3‐MA49‐VHL ternary model. (A) Modeled MA49 pharmacophore features used to guide the placement of docked quizartinib‐based PROTAC. (B) Docked MA49 and quizartinib‐based PROTAC fitting into the binding pockets of the FLT3‐MA49‐VHL model. (C) Superposition of the docked GSK215 pose (green) with the MOE Method4B modeled GSK215 pose (cyan). Hydrogen bonds (distance below 2.5 Å) are shown as blue dashed lines.

In light of this observation, induced fit docking was also applied to the FLT3‐MA49‐VHL ternary complex to evaluate its accessibility to other FLT3‐targeting PROTACs. The study focused on docking the quizartinib‐based FLT3 degrader, requiring it to adhere to the pharmacophoric features of the modeled MA49 pose (Figure 7A). Similar to FAK PROTACs, the docking results showed that the FLT3‐MA49‐VHL ternary complex was accessible to the MA49 and quizartinib‐based FLT3 PROTAC (Figure 7B,C). The top‐scored pose showed quizartinib and VHL ligands fitting well into their corresponding binding pockets. Quizartinib formed a hydrogen bond with Asp829 and established a π–stacking interaction with Phe830, maintaining its common binding mode as a type 2 inhibitor shown in the crystal structure (PDB ID 4XUF). The VHL ligand also exhibited its characteristic binding pattern with hydrogen bonds formed between the *R*‐hydroxyl group and Ser111 and His115, as well as between the carbonyl oxygen and Tyr98. Ultimately, docking of the negative controls for GSK215 and MA49 (MA72) in their respective ternary models revealed an unsuccessful binding mode of the VHL ligand, due to the opposite chirality of the hydroxy pyrrolidine‐ethanone moiety (Supporting Information S1: Figure S5).

### 2.4 MD Simulations of Crystallized Structures

MD simulations of experimental 3D structures were initiated by investigating the stability of VHL protein in complex with ligand 3 (PDB ID 5NVV) for 500 ns using Desmond. This was conducted to explore the dynamic behavior of VHL before simulating ternary complexes. The VHL ligand exhibited strong stability, with the key hydrogen bonds remaining stable throughout the simulation time. However, RMSD values were significantly elevated ranging from 4 to 6.5 Å for the Cα atoms and 2 to 4 Å for cocrystallized VHL ligand after fitting to the Cα atoms of all protein residues. This elevated RMSD was attributed to the flexibility of the VHL α‐helical tail, comprising residues 155–202, as indicated by the high RMSF values for these residues (3–5 Å). The VHL α‐helical tail includes residues involved in interactions with Elongin B/C proteins, which are part of the VHL E3 ubiquitin ligase complex. The high RMSD values of the α‐helical tail were attributed to the absence of these two proteins in the simulated complex. Notably, the Cα RMSD of the β‐sheet residues (59–154) remained within 0.5–1.5 Å (Supporting Information S1: Figures S6 and S7). Consequently, the flexible loop was excluded from the RMSD analysis in subsequent simulations.

**Figure 8:**
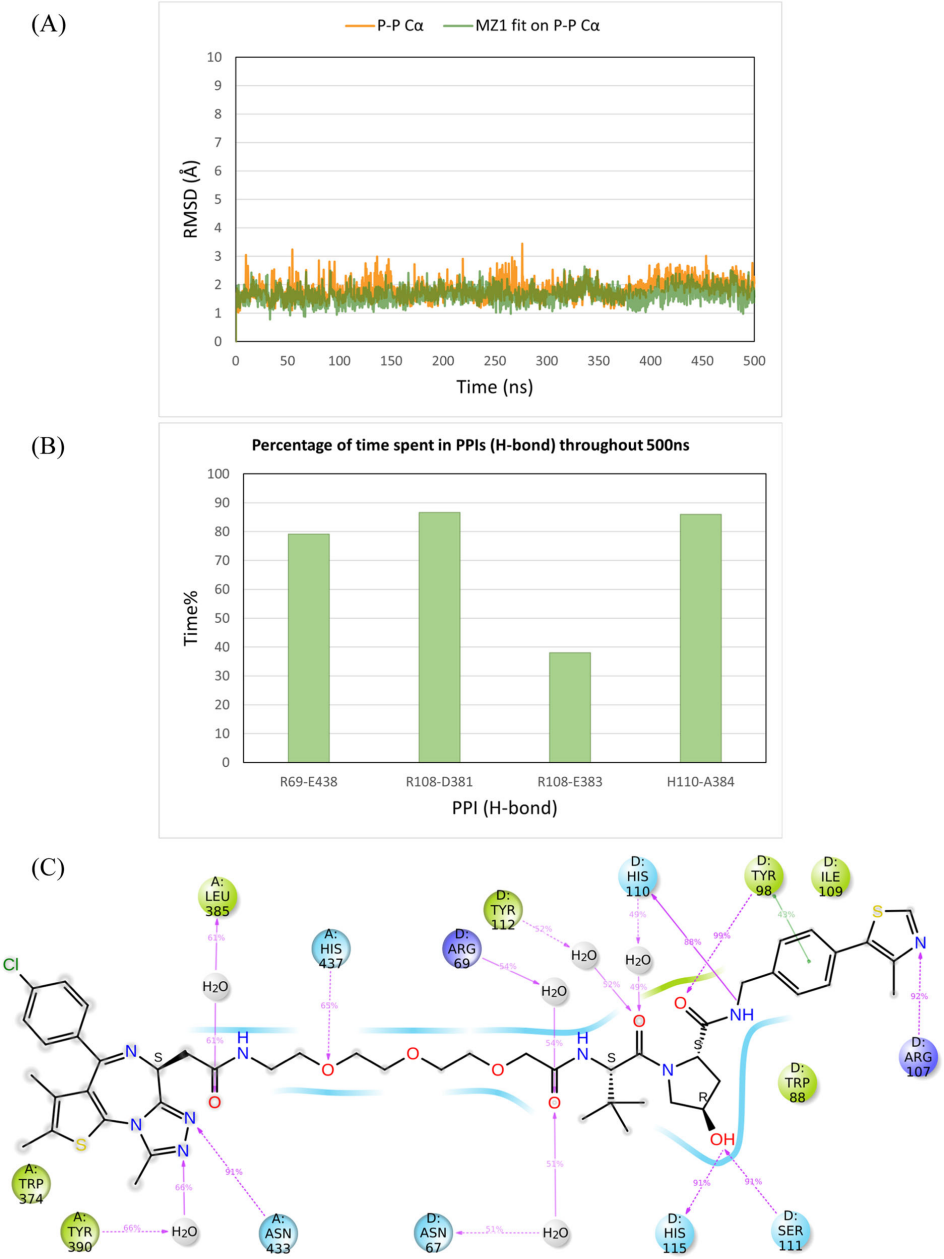
Analysis of the 500 ns MD simulation of the BRD4BD2‐MZ1‐VHL ternary complex (PDB ID 5T35). (A) RMSD values of the protein Cα (orange) and MZ1 fitting on the protein Cα (green). (B) The percentage of time spent in PPIs (H‐bonds) over the entire 500 ns. (C) Schematic representation of detailed MZ1 atom interactions with protein residues.

Next, the ternary complex BRD4BD2‐MZ1‐VHL (PDB ID 5T35) was simulated for 500 ns to assess whether our simulation protocol could accurately reflect the stability of a well‐characterized ternary complex. This crystal structure was selected since the same protein–protein conformation is crystallized with different PROTACs showing different linker lengths, attachment points, and VHL ligands, by different research groups, indicating strong stability [35, 36, 37, 38]. The ternary complex remained stable with RMSD values around 2 Å for the Cα atoms as well as the PROTAC MZ1 throughout the simulation time (Figure 8A). MZ1 interactions within both the BRD4BD2 and VHL binding pockets were preserved and the key PPIs of Arg69‐Glu438, Arg108‐Asp381, Arg108‐Glu383, and His110‐Ala384, observed in the 3D structure, remained intact over time (Figure 8B,C). These PPIs, particularly the salt bridges formed between arginine and either aspartate or glutamate residues, are known to stabilize this ternary conformation. The experimental ternary complex WEE1‐AZD1775 PROTAC‐VHL (PDB ID 8WDK) was also simulated for 500 ns. Similarly, the ternary complex was stable throughout the simulation time with Cα RMSD of 3 Å and PROTAC RMSD of 2 Å. Six PPIs of Ser68‐Arg393, Arg69‐Glu390, Gly106‐Asn441, Arg108‐Asn441, Arg108‐Asn455, and Tyr112‐Glu390 were identified in the 3D structure and showed strong stability over time. AZD1775 PROTAC also showed robust interactions within the binding pockets of the two protein monomers (Supporting Information S1: Figure S8).

**Figure 9:**
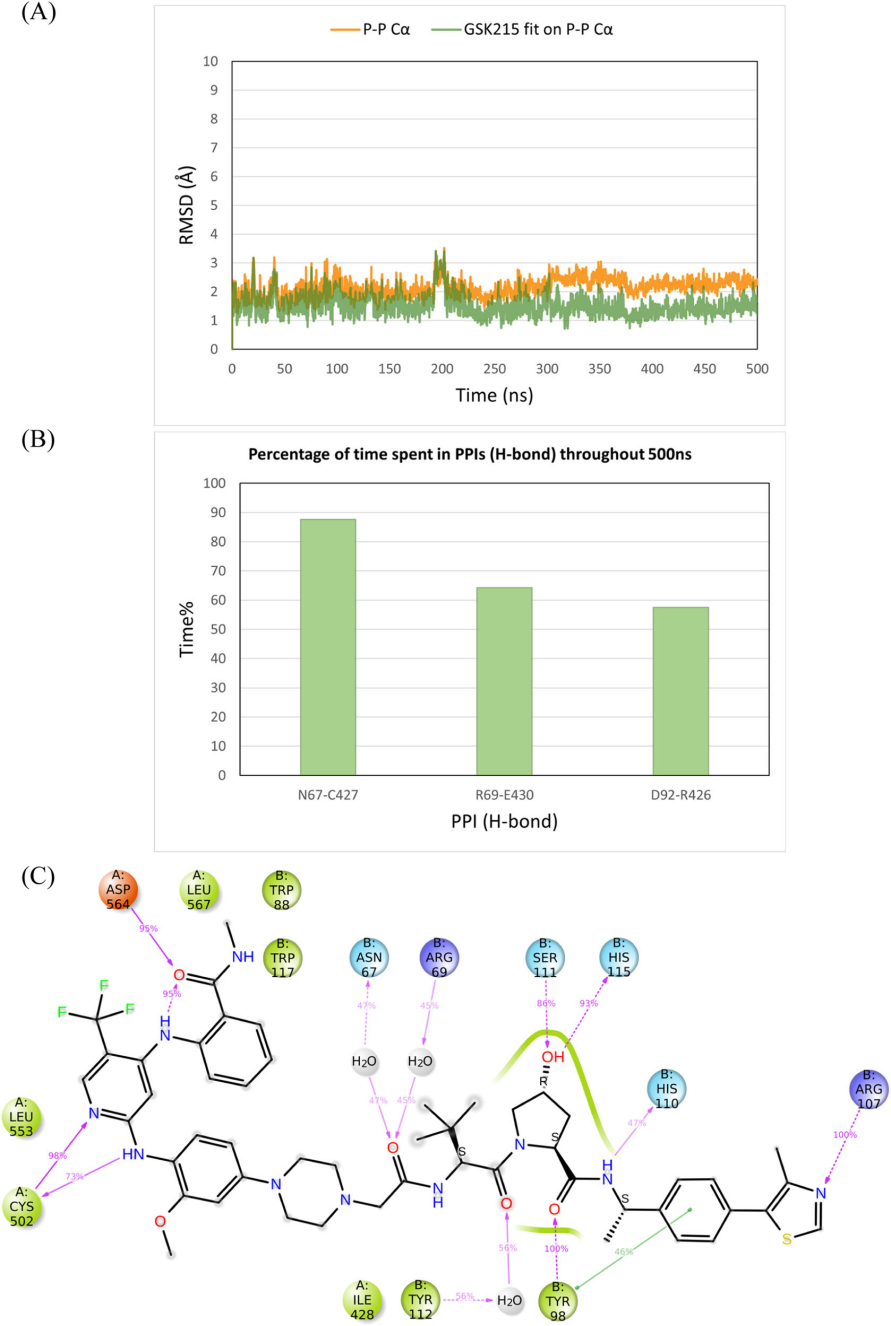
Analysis of the 500 ns MD simulation of the modified FAK‐GSK215‐VHL ternary complex. (A) RMSD values of the protein Cα (orange) and GSK215 fitting on the protein Cα (green). (B) The percentage of time spent in PPIs (H‐bonds) over the entire 500 ns. (C) Schematic representation of detailed GSK215 atom interactions with protein residues.

On the other hand, MD simulations of the FAK‐GSK215‐VHL ternary complex (PDB ID 7PI4) revealed structural instability throughout the 500 ns simulation time. The conformation of the complex began to deviate after 200 ns and failed to stabilize into any new consistent structure despite the PROTAC maintaining its interactions within the respective protein binding sites (Supporting Information S1: Figures S9 and S10). A critical observation was the absence of a loop (residues 572–582) in the 7PI4 3D structure located away from the interaction interface between FAK and VHL. To investigate whether the missing loop was responsible for the structural instability, the FAK monomer from the 7PI4 structure was replaced with the FAK structure from the PDB file 6I8Z, in which residues 572–582 are solved in the 3D structure. This newly reconstructed ternary complex was minimized and subjected to a 500 ns MD simulation. The results showed marked improvement in stability with a steady Cα and PROTAC RMSD of around 2 Å, confirming the importance of the previously missing loop for the structural integrity of the complex (Figure 9A). Also, the PPIs of Asn67‐Cys427, Arg69‐Glu430, and Asp92‐Arg426 as well as the PROTAC interactions remained stable and consistent with those identified in the experimental data throughout the simulation time (Figure 9B,C). These findings validate the robustness of the MD simulation protocol used in this study and indicate that the stability of a modeled ternary complex structure further demonstrates the success in capturing the native‐like conformation. Moreover, the results highlight the critical role of loop regions in maintaining structural stability, even when located outside the primary interaction interfaces of ternary complexes. Therefore, all loops of the kinase domain are crucial for ternary complex modeling, not only to prevent false protein–protein docking solutions but also to avoid unrealistic dynamic results.

### 2.5 MD Simulations of Modeled Ternary Complex Structures

**Figure 10:**
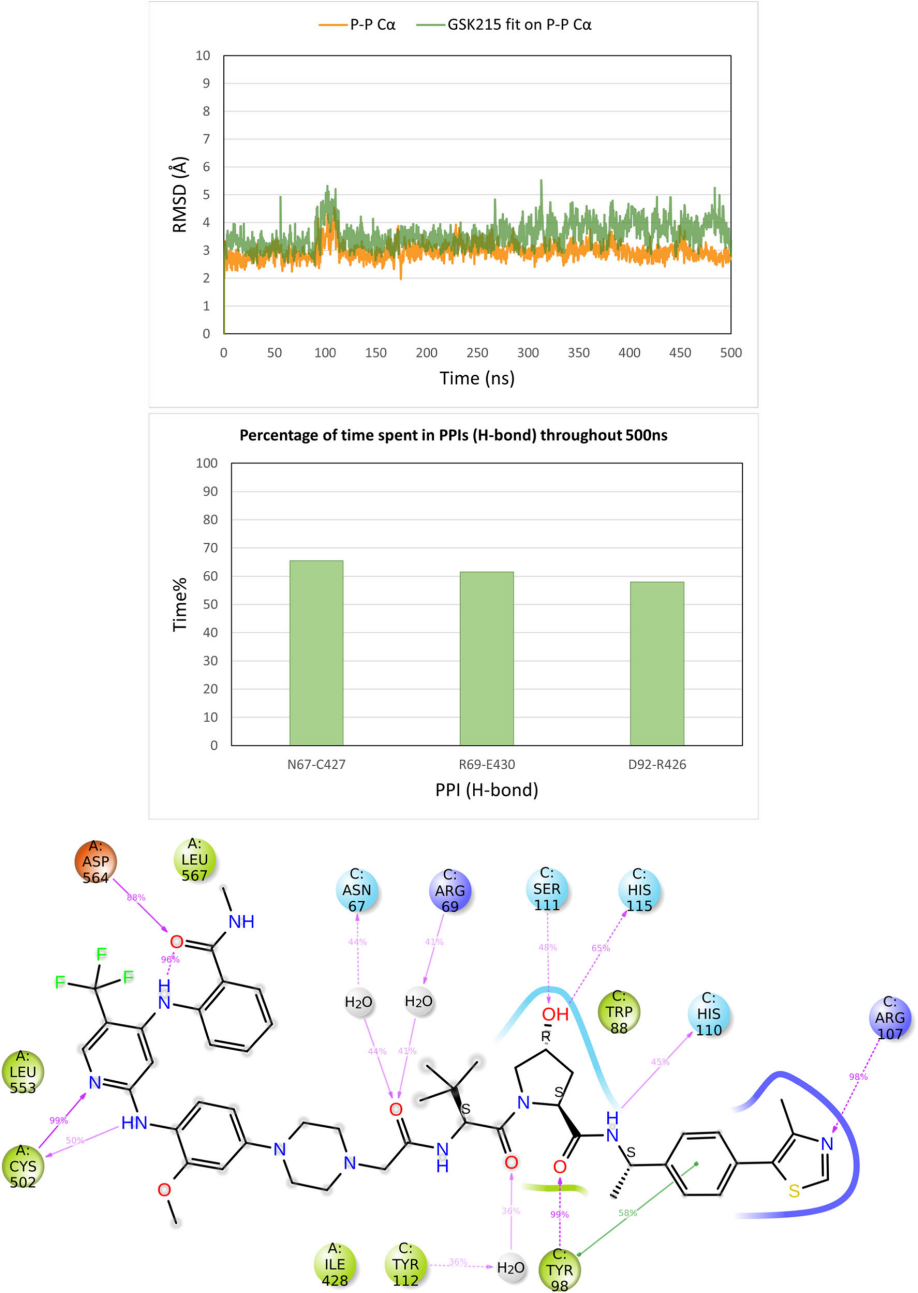
Analysis of 500 ns MD simulation of the modeled FAK‐GSK215‐VHL ternary complex obtained by MOE Method 4B. (A) RMSD values of the protein Cα (orange) and GSK215 fitting on protein Cα (green). (B) The percentage of time spent in PPIs (H‐bonds) over the entire 500 ns. (C) Schematic representation of detailed GSK215 atom interactions with protein residues.

The modeled ternary complex structures of FAK with GSK215, PROTAC1, PROTAC3 and PROTAC6 were also simulated for 500 ns. This was done to check whether the GSK215 ternary model generated by MOE Method 4B, which deviated from the experimental structure by an RMSD of 4.96 Å, would be adjusted and stabilized at the experimentally determined conformation. Furthermore, the simulations of PROTAC1, PROTAC3, and PROTAC6 ternary complexes, generated through induced fit docking, were conducted to assess whether the protein–protein conformation remains stable with different active PROTACs. The modeled GSK215 ternary complex structure showed Cα and GSK215 RMSD values ranging between 2.5 and 3.5 Å when aligned on Frame 0 as depicted in Figure 10A. When the experimental 3D structure was used as a reference, Cα and GSK215 RMSD stabilized at 1–2 Å, demonstrating successful relaxation and adjustment of the modeled conformation to better resemble the experimental one (see Supporting Information). PPIs and GSK215 interactions were consistent throughout the simulation time as observed with the experimental 3D structure (Figure 10B,C). PROTAC1 and PROTAC3 ternary complex models showed the same dynamic behavior, further demonstrating that the same protein–protein conformation can fit different active PROTACs (Supporting Information S1: Figures S11 and 12).

**Figure 11:**
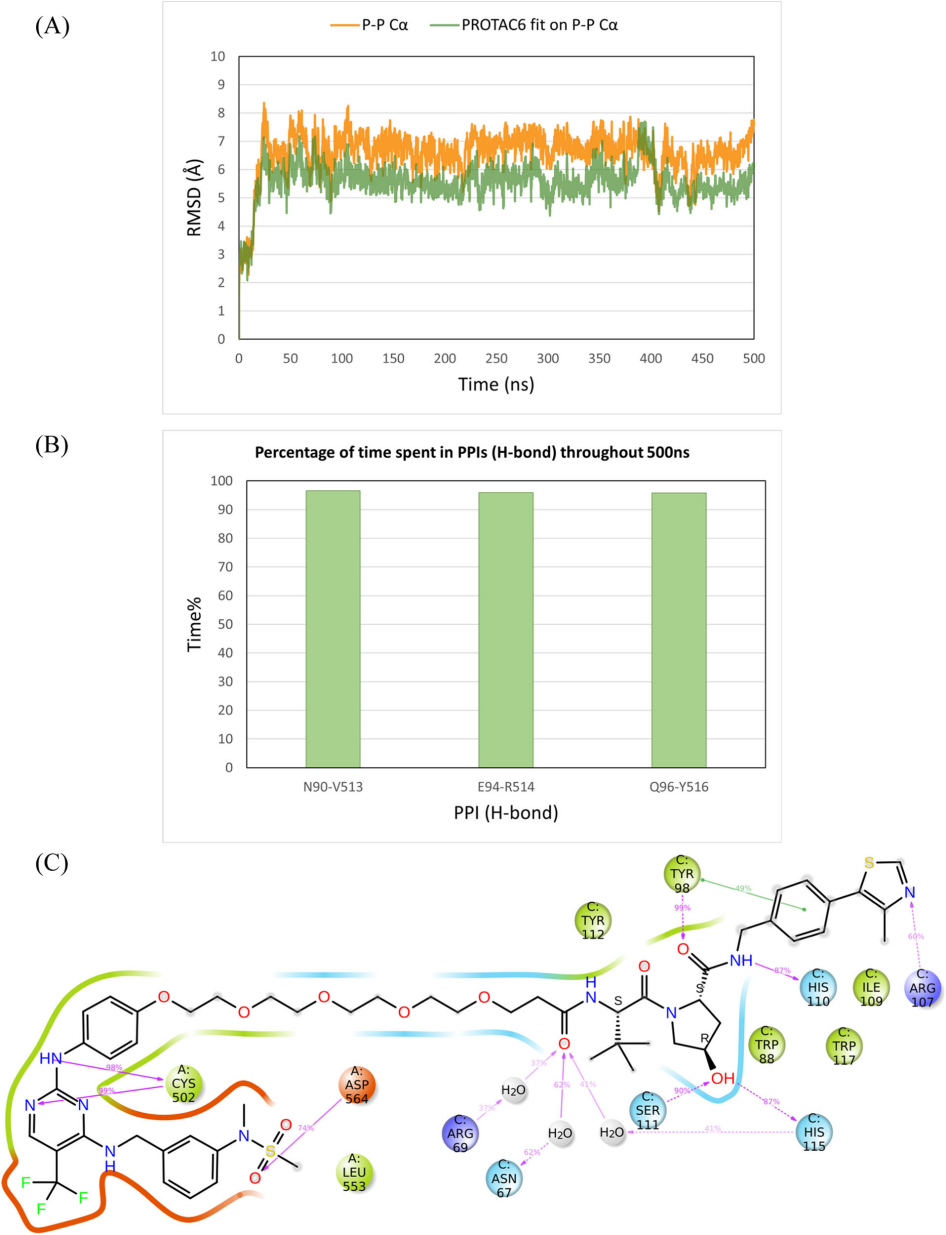
Analysis of the 500 ns MD simulation of the modeled FAK‐PROTAC6‐VHL ternary complex obtained via induced fit docking. (A) RMSD values of the protein Cα (orange) and PROTAC6 fitting on the protein Cα (green). (B) The percentage of time spent in PPIs (H‐bonds) over the entire 500 ns. (C) Schematic representation of detailed PROTAC6 interactions with protein residues.

**Figure 12:**
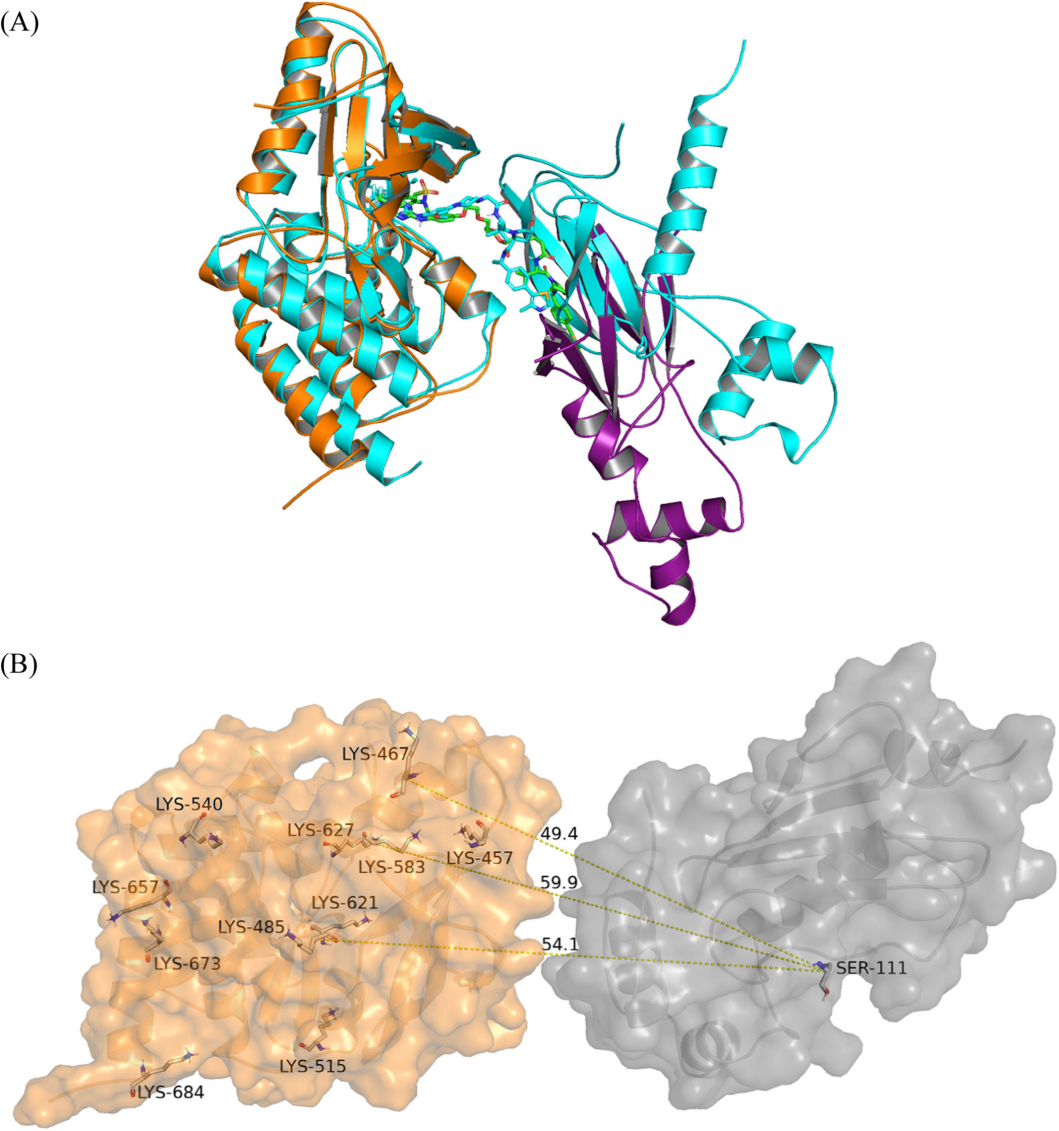
New adopted conformation of the PROTAC6 ternary complex model. (A) Cartoon representation with FAK in orange and VHL in purple, superposed on the 7PI4 crystal structure (cyan). (B) Distances between solvent‐exposed lysines of FAK in the new conformation and Ser111 of the E2 ligase, indicated by yellow dashed lines.

However, the PROTAC6 ternary model changed its protein–protein conformation after 25 ns, stabilizing at a new conformation while keeping the PROTAC interactions stable inside the protein binding pockets. Cα and PROTAC6 RMSD values were stabilizing at 7 and 6 Å, respectively (Figure 11A). New stable PPIs were formed between Asn90 and Val513, Glu94, and Arg514 as well as Gln96 and Tyr516 (Figure 11B). The occupancy rates of PROTAC6 interactions within the binding pockets are detailed in Figure 11C. Visual inspection of trajectory snapshots revealed a significant adjustment of the VHL binding mode to FAK to accommodate the long PROTAC6 linker (Figure 12A). Subsequently, this new protein–protein conformation was compared with the experimentally derived conformation in terms of productivity (distance of E3‐ligase and lysine residues of target protein) and prime interaction energy. The conformation of the last frame of the PROTAC6 simulation trajectory was exported and aligned with the modeled ubiquitination machinery. Three new solvent‐exposed lysine residues were positioned within 50–60 Å of Ser111 of the E2 ligase (Figure 12B). Additionally, the prime interaction energy of protein–protein conformations was calculated for each frame per 1 ns over the last 40 ns of the simulation trajectories (Table 4). The net energy of the PROTAC6 protein–protein conformation was similar to that of the GSK215 experimental 3D structure as well as the modeled structures ranging between –10396.92 and –10421.58 kcal/mol. However, PROTAC6 showed a protein–protein interaction energy of –42.33 Kcal/mol, which was slightly more negative than other FAK PROTACs. To test whether this conformation is unique to PROTAC6 and cannot be induced by other PROTACs, we performed induced fit docking for all FAK ROTACs using the conformation from the last frame of the PROTAC6 MD trajectory. The poses produced were only for PROTAC5 and PROTAC6, which were successfully fitting in the new conformation (Supporting Information S1: Figure S13).

**Table 4:** Total binary complex energy (Prime) and protein–protein interaction energy (MMGBSA) calculated from snapshots taken every 1 ns during the final 40 ns of each MD trajectory.

Ternary complex	Average interaction energy ∆H (Kcal/mol ± SD)	
	Prime energy	MMGBSA
Experimental FAK‐GSK215‐VHL	–10399.49 ± 34.75	–36.94 ± 5.25
Modeled FAK‐GSK215‐VHL	–10396.92 ± 22.79	–36.40 ± 6.75
FAK‐PROTAC1‐VHL	–10404.65 ± 35.51	–34.69 ± 6.83
FAK‐PROTAC3‐VHL	–10395.25 ± 32.20	–30.94 ± 5.21
FAK‐PROTAC6‐VHL	–10421.58 ± 43.46	–42.33 ± 5.35
FLT3‐MA49‐VHL	–12482.58 ± 28.92	–77.60 ± 5.49
FLT3‐Quizartinib PROTAC‐VHL	–12496.52 ± 24.63	–75.09 ± 3.65

**Figure 13:**
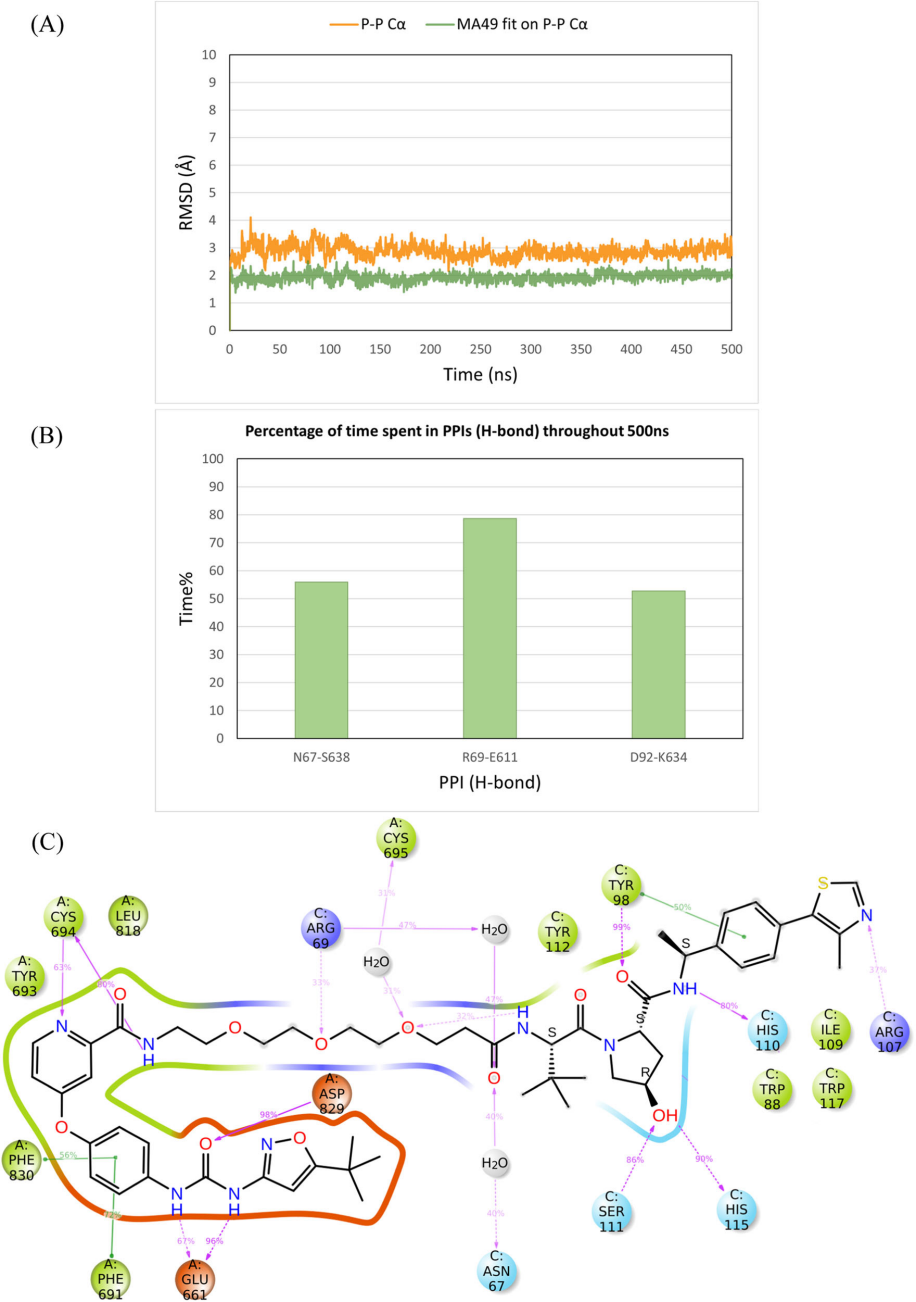
Analysis of the 500 ns MD simulation of the modeled FLT3‐MA49‐VHL ternary complex obtained by MOE Method 4B. (A) RMSD values of the protein Cα (orange) and MA49 fitting on the protein Cα (green). (B) The percentage of time spent in PPIs (H‐bonds) over the entire 500 ns. (C) Schematic representation of detailed MA49 atom interactions with protein residues.

**Figure 14:**
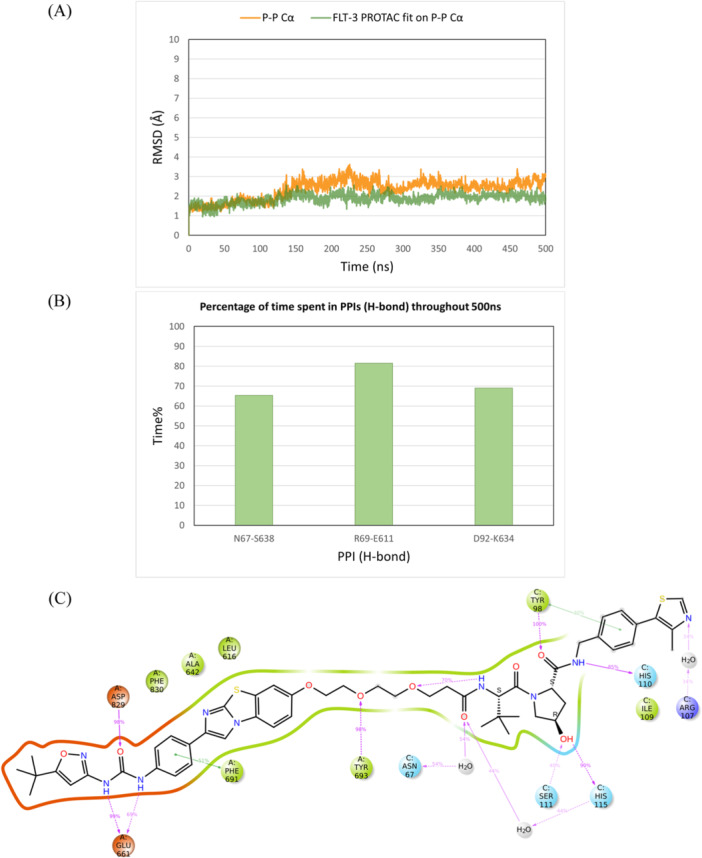
Analysis of the 500 ns MD simulation of the modeled FLT3 quitartinib‐based PROTAC ternary complex obtained by MOE Method 4B. (A) RMSD values of the protein Cα (orange) and PROTAC fitting on the protein Cα (green). (B) The percentage of time spent in PPIs (H‐bonds) over the entire 500 ns. (C) Schematic representation of detailed PROTAC atom interactions with protein residues.

MD simulations of the MA49 ternary complex, obtained by MOE Method 4B, and the quizartinib‐based PROTAC ternary complex, obtained via induced fit docking, were subsequently conducted. Both ternary complex structures showed high stability over the simulation time, with Cα and PROTAC RMSD values stabilizing at 2–3 Å (Figures 13A and 14A). Interestingly, the active VHL residues observed in the FAK‐VHL structure were the same as those interacting with FLT3. Robust PPIs were observed between Asn67 and Ser638, Arg69, and Glu611, and Asp92 and Lys634, as depicted in Figure 13B and Figure 14B. MA49 and the quizartinib‐based PROTAC demonstrated stable interactions within the FLT3 and VHL binding sites (Figures 13C and 14C). Moreover, the total energy of the binary complex and the protein–protein interaction energy in each ternary complex were comparable, with values of –12482.58 and –77.60 for MA49, and –12496.52 and –75.09 for Quizartinib PROTAC, respectively.

Finally, to further assess the observed stability results of the FAK and FLT3 PROTACs, 500 ns MD simulation runs were performed for the ternary complex models of negative controls of GSK215 and MA49 (MA72). Specifically, we wanted to test whether all stable complexes observed in MD simulations correlate with efficient degradation or if some stable complexes fail to induce degradation. This helps refine the interpretation of stability as a predictor of PROTAC efficacy. The RMSD plots and interaction occupancy rates of both negative controls are provided in Supporting Information (Supporting Information S1: Figure S14). The models generated for the PROTACs negative controls remained stable throughout the 500 ns MD simulation. The protein–protein conformations of FAK‐VHL and FLT3‐VHL exhibited Cα RMSD values ranging between 2.5 and 3.5 Å over the entire simulation period. Additionally, the false binding modes of the negative controls of GSK215 and MA49 (MA72), remained stable, with RMSD values of 3–4 Å and 2–3 Å, respectively. The loss of interactions with Ser111, His115, and Tyr98 within the VHL binding site remained consistent throughout the simulation. The reversed chirality of the hydroxyproline moiety and the carbonyl oxygen of the VHL warhead in the two PROTACs prevented the formation of interactions with these residues. However, the stability of the binary conformations, along with interactions established by other functional groups of the negative PROTACs, contributed to maintaining the PROTAC within the ternary complex. This underscores the importance of induced‐fit docking as a pre‐screening tool in PROTAC development. MD simulations can then be applied to provide insights into the dynamics of well accommodated PROTACs and evaluate their structure to function relationship.

## 3 Discussion

To initiate protein degradation, PROTACs must bridge the target protein with the E3 ligase, bringing the target protein into contact with the ubiquitination and degradation machinery. They differ from the traditional binding‐based pharmacology of small‐molecule inhibitors by enabling the complete elimination of the target protein [39, 40]. The human genome encodes more than 600 E3 ligases. To date, ligands that target CRBN are the most commonly used in PROTAC development, followed by those that target VHL. Other less commonly utilized E3 ligases include IAP, DCAF15, and MDM2 [5]. The tendency to form a ternary complex of target protein, PROTAC, and E3 ligase is essential for the functionality of PROTACs. Ternary complex modeling aims to optimize the design of various PROTAC linkers with respect to their length, flexibility, and positioning [41]. Modeling also provides insights into how different active PROTACs can influence the dynamic behavior of ternary complexes, thereby contributing to a better understanding of the structure‐function relationship [23, 24].

Several computational tools were developed to model PROTACs. Available tools include ternary complex generators (e.g., MOE, PRosettaC, and ICM) and binary complex generators (e.g., HADDOCK, ZDOCK, and AlphaFold‐multimer) [30, 31, 42, 43, 44]. Briefly, MOE, PRosettaC, and ICM start with the 3D structures of the two proteins bound to their ligands followed by the superposition of PROTAC conformations on the binary complex warheads to generate ternary complexes [30, 31]. HADDOCK and ZDOCK dock 3D structures of proteins bound to their ligands with defined interacting residues whereas AlphaFold‐multimer requires two protein sequences as input and uses deep learning to build a binary complex [42, 43, 44]. Ternary complex generation tools offer the advantage of incorporating the PROTAC structure in the protein–protein complex. However, they assume that the interacting proteins remain rigid. This simplification does not account for the dynamic nature of ternary complexes or their ability to undergo conformational adaptation in response to different PROTAC linkers. Moreover, in MOE, the pregenerated static protein–protein docking solutions can only be reused to generate Method 4B ternary complexes if the protein warheads are similar to those used in the PROTAC. Therefore, to accurately evaluate and optimize PROTACs with varying linker designs and warheads, incorporating other computational tools such as induced fit docking and MD simulations is essential. A combined approach of induced fit docking and MD simulations has been shown to accurately predict ligand–receptor structures, offering valuable insights for structure‐based drug discovery, particularly for challenging targets [45]. Harnessing these tools for the modeling of VHL‐mediated PROTACs further broadens the scope of their applicability. This approach captures the flexibility and adaptability of the ternary complex, providing more reliable insights into PROTAC stability and functionality.

Our objective was to develop a highly accurate and efficient modeling approach to optimize the structural design of VHL‐mediated PROTACs, with a particular focus on our in‐house MA49 PROTAC targeting FLT3. This study addressed the challenge of modeling ternary complexes without known 3D structures by using unbound monomers to create the ternary models. By using unbound monomeric structures, we avoided bias toward experimentally derived structures and ensured that the modeling was not influenced by predetermined side‐chain orientations [46]. Accordingly, the VHL protein in complex with the VHL ligand 3 was used in all modeling runs for ternary complexes. The target proteins BRD4BD2 and SMARCA2 were obtained in complex with their respective warheads from their 3D ternary structures. The FAK‐VS4718 complex was obtained through docking into the PDB structure of 6I8Z while the WEE1‐AZD1775 complex was retrieved from the PDB file, 5V5Y. Our decision to compare two different modeling methods–MOE Method 4B and PRosettaC–aimed to identify the most reliable and accurate approach for generating ternary complexes for VHL PROTACs. The results showed that MOE Method 4B consistently reproduced the experimental 3D structures better than PRosettaC. Furthermore, the MOE Method 4B modeling of WEE1‐AZD1775 PROTAC‐VHL demonstrated the critical impact of missing loops on the modeling reproducibility of experimental 3D structures and emphasized the importance of including all loops of the kinase domain in the protein–protein docking. Therefore, the FLT3‐MA49‐VHL ternary complex was generated using the FLT3 structure with a modeled loop of residues 708–782 that were missing in the crystal structure. To assess whether we could rely on the modeled structures when creating new PROTAC candidates, previously published active PROTACs were docked into the FAK‐GSK215‐VHL and FLT3‐MA49‐VHL models using induced fit docking. This strategy saved time by avoiding the need to create a new protein–protein docking ensemble with MOE Method 4B for each PROTAC that uses a different target inhibitor. The six studied defactinib‐based FAK PROTACs, as well as the FLT3‐based PROTAC with quizartinib, successfully fit into their respective ternary models and reproduced the common binding patterns in each protein‐binding site. In contrast, docking negative controls of GSK215 and MA49 into each ternary model revealed unsuccessful binding patterns of the VHL ligands, attributed to the reversed chirality of the hydroxyproline moiety. In both compounds, the hydroxyl group of hydroxyproline was oriented away from Ser111 and His115, while the adjacent carbonyl oxygen was positioned opposite to Tyr98. The success of these docking experiments thus provides a solid foundation for designing new active degraders using the modeled structures. Additionally, this observation aligns with reports of the accessibility of a single identical conformation of the BRD4BD1‐VHL, BRD4BD2‐VHL, and SMARCA2‐VHL binary complexes to various sets of active PROTACs [35, 36, 37, 38, 47, 48, 49].

After ternary complex modeling and induced fit docking, MD simulations were conducted to investigate the dynamic behavior of the generated models. In our Desmond MD protocol, A 500‐ns timeframe was sufficient to observe the stability of the 3D structures of BRD4BD2‐MZ1‐VHL, FAK‐GSK215‐VHL, and WEE1‐AZD1775 PROTAC‐VHL in two independent runs. This finding contrasts with the reported instability of crystallized VHL ternary complexes observed in three independent MD simulation runs conducted using a different simulation protocol [20]. However, for the FAK‐GSK215‐VHL ternary complex, the FAK structure had to be replaced with that from PDB ID 6I8Z to evaluate the stability of the complex. Notably, the 3D ternary complex lacked the FAK loop comprising residues 572–582 located adjacent to the FAK‐VHL interaction interface. While these residues do not directly interact with the VHL protein, their absence affected the stability of the interaction interface between FAK and VHL. These loop residues act as crucial scaffolding, anchoring binding partners in place to reduce conformational variations and promote a stable interaction surface. Their absence can result in higher flexibility, weaker binding, and lower overall stability of the ternary complex. Further MD simulations of the FAK PROTACs, GSK215, PROTAC1, and PROTAC3 revealed stable ternary models. The FAK‐PROTAC6‐VHL model, however, changed its conformation after 25 ns and stabilized in a new conformation over the remaining 500‐ns MD trajectory. PROTAC5 and PROTAC6 have the longest linkers among the FAK PROTACs (10 and 16 atoms, respectively), and the new conformation of the ternary complex was unique to these PROTACs. Long linkers offer significant flexibility, allowing the target protein and VHL to explore a broader conformational space. This can result in the discovery or stabilization of a new protein–protein conformation not observed with short linkers. Additionally, long linkers induce a comfortable distance between the two proteins to avoid steric clashes. This distance cannot be accommodated by short linkers as demonstrated by the unsuccessful docking of GSK215, PROTAC1, PROTAC2, PROTAC3, and PROTAC4 into the new PROTAC6 protein–protein conformation. This highlights the importance of PROTAC linkers for ternary complex conformation and the need for MD simulations to gain a holistic view of the relationship between PROTAC structure and degradation potential. The new conformation of the PROTAC6 ternary complex was accessible to ubiquitination and showed protein–protein interaction energy comparable to that of the experimental GSK215 and the other PROTAC ternary complex structures. The ubiquitination accessibility of lysine residues on a target protein is a crucial determinant of PROTAC‐mediated degradation activity and selectivity. Lv et al. demonstrated that computational modeling can effectively identify the location and orientation of accessible lysines, providing valuable insights for the design of PROTACs targeting BCL‐xL and BCL‐2 proteins. Furthermore, they found that modifying the PROTAC linker can induce conformational changes in the ternary complex, thereby exposing different lysines on the target protein's surface [50]. Notably, the newly observed PROTAC6 ternary complex conformation revealed the same number of ubiquitination‐accessible lysines as the original structure but at different locations, explaining its preserved degradation activity despite the significant conformational change.

Subsequent MD simulations of FLT3 PROTAC models of MA49, as well as the quizartinib‐based FLT3 PROTAC, also showed conformational stability and comparable protein–protein interaction energies. We previously reported MA49 as a highly potent VHL‐mediated PROTAC candidate with potential for the treatment of FLT3‐ITD‐driven acute myeloid leukemia (AML). MOLM‐3 AML cells were treated with varying concentrations of MA49, resulting in FLT3‐ITD degradation with a DC₅₀ of 11.15 nM after 24 h of incubation. Additionally, MA49 induced cellular apoptosis with an IC₅₀ of 4.83 nM following 72 h of incubation [32]. The quizartinib‐based FLT3 PROTAC reported by Burslem et al. also demonstrated similar degradation potency against FLT3‐ITD. Immunoblotting revealed a loss of FLT3 protein levels at a 5 nM concentration, while a cellular assay demonstrated cytotoxicity with an IC₅₀ of 0.6 nM following 24‐h and 72‐h treatments of MV4‐11 AML cells, respectively. Both PROTACs demonstrated the superiority of FLT3 degradation over inhibition by the small molecules MA68 and quizartinib [33]. The modeled FLT3‐MA49‐VHL and FLT3‐quizartinib PROTAC‐VHL ternary complex structures successfully accommodated the different linkers of the two PROTACs and showed identical PPIs. Three solvent‐exposed lysine residues were observed to be accessible for ubiquitination, while key PROTAC interactions as well as PPIs remained stable in MD simulations. This structural analysis aligns well with the observed activity of MA49 and quizartinib PROTACs and provides insights into their structure–activity relationships. The most common PPIs observed across all ternary complexes were salt bridges formed between arginine residues and either glutamate or aspartate residues. These strong interactions contributed to the stability observed during MD simulations. In the new conformation of the PROTAC6 ternary complex, salt bridges also played a key role in maintaining stability. The formation of these salt bridges could be an important factor in the stabilization of the ternary complexes to facilitate efficient ubiquitination.

Ultimately, MD simulations of the modeled structures of the negative controls demonstrated stable binary conformations and false PROTAC binding modes. While this observation highlights the robustness of the modeled FAK‐VHL and FLT3‐VHL binary conformations, it also underscores that ternary complex stability does not necessarily correlate with degradation potential. Therefore, induced fit docking alongside ubiquitination accessibility tests serve as crucial prescreening steps for identifying effective PROTACs before performing MD simulations. PROTACs that fail to establish the necessary interactions within protein binding pockets cannot effectively recruit both proteins and are likely to be inactive. On the other hand, MD simulations serve as a crucial tool to evaluate the stability of well‐accommodated PROTACs in the protein binding pockets and assess the adaptation of ternary complexes to different PROTAC linkers.

## 4 Conclusion

This study introduced an efficient computational approach to support the structural design of VHL‐mediated PROTACs, especially in cases where ternary complex 3D structures are unavailable. By using unbound monomers, we minimized bias toward predetermined conformations. The conducted retrospective induced‐fit docking experiments showed that active PROTACs fit into their corresponding models. MD simulations also confirmed the stability of the ternary complexes over time, with one notable case, FAK‐PROTAC6‐VHL, exhibiting a significant conformational shift due to its longer linker, yet still retaining its degradation activity. Overall, the results demonstrate the utility of this PROTAC modeling approach and suggest that it can be successfully applied to new VHL‐mediated PROTACs. Ongoing chemical optimizations aimed at obtaining new, potent FLT3 PROTACs will further validate this approach.

## 5 Experimental

### 5.1 Protein Preparation

Protein 3D structures were retrieved from the Protein Data Bank (PDB) and prepared using the protein preparation wizard module in Schrödinger Suite v2021 [51]. The VHL 3D structure in complex with (2*S*,4 *R*)‐4‐hydroxy‐1‐[(2*S*)‐2‐(2‐hydroxyacetamido)‐3,3‐dimethylbutanoyl]‐*N*‐{[4‐(4‐methyl‐1,3‐thiazol‐5‐yl)phenyl]methyl}pyrrolidine‐2‐carboxamide (Ligand 3), PDB file: 5NVV, was used in all cases for ternary complex generation [12]. 3D structures of the studied target proteins BRD4‐BD2, SMARCA2, FAK, WEE1 and FLT3 were retrieved from the PDB (ID 5T35 [35], 7S4E [49], 6I8Z [52], 4XUF [53], 5V5Y [54] and 8WDK, respectively). The VHL protein, its ligand and the linker were removed from the ternary complex structures of PDB 5T35 and PDB 7S4E before preparation, keeping tert‐butyl 2‐[(9*S*)‐7‐(4‐chlorophenyl)‐4,5,13‐trimethyl‐3‐thia‐1,8,11,12‐tetrazatricyclo[8.3.0.0^2,6^]trideca‐2(6),4,7,10,12‐pentaen‐9‐yl]acetate (JQ1 ligand) and 2‐[6‐amino‐5‐(piperazin‐1‐yl)pyridazin‐3‐yl]phenol (SMARCA‐BD ligand) in each target binding site, respectively. Ligand structures are shown in 2D in Figure 1. The preparation procedures involved adding hydrogen atoms, missing side chains, and missing loops while removing ions and water molecules from the protein structures. The protonation states and tautomeric forms of amino acids were optimized using the PROPKA tool at pH 7.0 [55]. Finally, the a restrained energy minimization of the prepared structures was performed using the OPLS4 force field and the default setup [56]. For MD simulations of the 3D structures, ternary complexes of PDB 5T35 (BRD4BD2‐MZ1‐VHL) and PDB 7PI4 (FAK‐GSK215‐VHL) were prepared using the same settings [35, 57]. However, the FAK monomer in PDB 7PI4 had a missing loop of residues at positions 572 to 582, which the Prime module failed to model. Therefore, it was replaced with the FAK structure from the PDB 6I8Z file, obtained with 3‐methoxy‐*N*‐(1‐methylpiperidin‐4‐yl)‐4‐({4‐[(3‐oxo‐2,3‐dihydro‐1H‐inden‐4‐yl)oxy]‐5‐(trifluoromethyl)pyrimidin‐2‐yl}amino)benzamide (BI‐4464 ligand), where the missing loop was resolved (See Supporting Information). This was carried out to test the effect of this loop on the structural integrity of the ternary complex during simulation. Similarly, the FLT3 structure from PDB 4XUF had a long missing loop (residues 708–782), which the Prime module also failed to model. PDB 4XUF structure is crystallized with quizartinib in the DFG‐out conformation, which is necessary to accommodate such type 2 inhibitors that we wanted to study in this study. As no PDB file contains this loop in a crystallized form, it was modeled using the SWISS‐MODEL web tool before being subjected to the preparation procedures (See supplementary) [58]. In the case of WEE1 (PDB ID 5V5Y) crystalized with 1‐[6‐(2‐hydroxypropan‐2‐yl)pyridin‐2‐yl]‐6‐{[4‐(4‐methylpiperazin‐1‐yl)phenyl]amino}‐2‐(prop‐2‐en‐1‐yl)pyrazolo[3,4‐d]pyrimidin‐3‐one (AZD1775), the prime modeled loop of residues 438–456 was removed to investigate the impact of missing kinase domain loops on the ternary complex modeling results. Subsequently, 2‐[(2‐{[2‐methoxy‐4‐(morpholin‐4‐yl)phenyl]amino}‐5‐(trifluoromethyl)pyridin‐4‐yl)amino]‐*N*‐methylbenzamide (VS‐4718 ligand) and 3‐(4‐{[(5‐tert‐butyl‐1,2‐oxazol‐3‐yl)carbamoyl]amino}phenoxy)‐*N*‐methylbenzamide (MA68 [32]) were docked into the prepared structures of FAK and FLT3.

### 5.2 Ligand Preparation

The 3D structures of FAK and FLT3 inhibitors in addition to the studied PROTACs (Figure 1) were prepared and energy‐minimized using the OPLS4 force field by the LigPrep module. Available negative controls of GSK215 and MA49 PROTACs (MA72, wrong VHL stereoisomer i.e., unable to bind to VHL) were assigned by reversing the chirality of the hydroxy proline moiety. The ionization state was kept with no change while that of the piperazine moiety was monitored by MarvinSketch at pH 7.0 and assigned manually. No tautomers were generated and the specified chiralities were retained. Target protein inhibitor scaffolds were subsequently used as input for Glide SP molecular docking. The prepared PROTAC structures were saved as SDF files to be used as input for ternary complex generation.

### 5.3 Glide SP Docking

Molecular docking of the FAK and FLT3 inhibitors was conducted using the Standard Precision (SP) mode by Glide Ligand Docking module. The grid box was generated using the co‐crystallized ligands, BI‐4464 and quizartinib, as the centroid with an inner box size of 10 × 10 × 10 Å, using the receptor grid generation module. To validate the docking protocol, redocking of the co‐crystallized inhibitors was performed, along with the inhibitor scaffolds used in the designed PROTACs. The RMSD values of top‐scoring poses with respect to co‐crystallized inhibitors were below 1.0 Å, aligning closely with the crystallographic data. A maximum of 10 docking poses were generated for each inhibitor, with all other settings kept at their default values. The generated poses were scored using the Glide docking score. Finally, the Glide top‐scoring poses were taken in complex with their respective monomers as input for ternary complex generation.

### 5.4 Ternary Complex Generation

Ternary complex models were generated using two different modeling programs, Method 4B implemented in MOE version 2019.01 [30, 59] and PRosettaC [31] VHL was paired with each of BRD4BD2, SMARCA2, FAK, WEE1 and FLT3, complexed with their cognate inhibitors, to generate ternary complex models for the MZ1, ACBI1, GSK215, AZD1775 PROTAC and MA49, respectively. In Method 4B in MOE, protein–protein docking was first conducted using residues within 4.5 Å of each bound ligand as the interaction site. Subsequently, a conformational ensemble of the PROTAC is generated using the conformational search tool, after which the PROTAC conformers are fitted onto the bound ligands in each protein pocket of the docking solutions. Only those conformers showing an RMSD of the maximum common substructure (MCS) of less than 3.5 Å are retained. In this study, protein–protein docking solutions showing a maximum contact area of 1340 Å^2^ were used during the conformational search step. Eventually, the ternary complex models generated are double‐clustered based on the Cα RMSD of the moving protein with a threshold of 10 Å and PROTACs RMSD threshold ranging from 1 to 3 Å, in increments of 0.5 Å. Since, the most populated double cluster mostly contains native‐like structures, the top three ternary complex models of the top cluster were visually inspected and taken further into analysis [30]. In PRosettaC, a collection of random ligand positions is generated, starting with a distance of 1 Å between anchor atoms. For each ligand pair, a random PROTAC conformation is generated, keeping the ligand positions fixed. The distances between successful ligand pairs are used as constraints in protein–protein docking. Initial protein–protein complexes are produced by PatchDock, ensuring that the distance between ligands falls within the set constraints. These binary complexes are then refined by Rosetta, which allows the proteins to adjust their conformation while keeping the ligands fixed. A collection of PROTAC conformations is then generated and aligned on the ligands of the binary complexes with a threshold of 0.5 Å. In the final step, ternary complex models with high Rosetta energy values are discarded, and the top‐scoring ternary complexes are clustered [31]. The produced models for BRD4BD2‐MZ1‐VHL, SMARCA2‐ABCI1‐VHL, and FAK‐GSK215‐VHL from both protocols were ultimately compared with the respective experimental 3D structures by calculating Cα RMSD, PROTAC RMSD, and PPIs in addition to their ubiquitination productivity.

Furthermore, MOE Method 4B was utilized to model the WEE1‐AZD1775 PROTAC‐VHL ternary complex both in the presence and absence of the flexible WEE1 loop of residues 438–456. MOE Method 4B was performing better while emphasizing the critical role of all kinase domain loops in preventing the generation of false ternary complex conformations. Consequently, MOE method 4B was applied to model the FLT3‐MA49‐VHL ternary complex. Ternary models of FAK‐GSK215‐VHL and FLT3‐MA49‐VHL were then taken further for induced fit docking of further active PROTACs.

### 5.5 Modeling of Ubiquitination Machinery

The ubiquitination system of CRL2VHL (VHL‐EB‐EC‐Cul2‐Rbx1‐E2‐NEDD8) was generated using PyMOL. The Rbx1‐E2‐NEDD8 arm was modeled by aligning the structure of Rbx1‐E2‐NEDD8‐Cul1‐Dcn1 (PDB ID 4P5O) on VHL‐EB‐EC‐Cul2‐Rbx1 complex (PDB ID 5N4W) superposed via the Rbx1 and Cullin subunits [60, 61]. The complex was then prepared and minimized as described in Section 2.1 (See Supporting Information). To check ubiquitination productivity, accessible lysine residues of the target protein were identified as those showing a solvent‐accessible surface area of more than 25% using Biovia Discovery Studio visualizer v2016. The target protein of the ternary models was added to the modeled ubiquitination machinery by superposing on the VHL subunit. Finally, the distance between the Cα atoms of the accessible lysine residues of the target and the catalytic Ser111 of the E2 ligase was calculated in PyMOL.

### 5.6 Induced‐Fit Docking

Induced‐fit docking implemented in MOE 2019.01 was performed to assess the ability of the modeled FAK‐GSK215‐VHL and FLT3‐MA49‐VHL structures to accommodate other previously published active degraders. Six active FAK PROTACs [34] and one FLT3 PROTAC [33] in addition to the negative controls of GSK215 and MA49 (MA72) were docked into their respective ternary models after being prepared by the LigPrep tool. The MOE induced fit docking places the active ligand into a user‐defined binding site inside a target receptor whose residue side chains are allowed to move freely during the refinement stage. PROTAC atoms in each ternary complex structure were used to define the active site. Pharmacophore placement was used to position the docked poses by applying the generated pharmacophore as a filter on the final docked poses. Three pharmacophore features were assigned for the target inhibitor scaffold and three for the VHL ligand with a sphere of 2 Å radius in each case. The specified features of the FAK inhibitor in GSK215 included the carbonyl oxygen and pyridine nitrogen as two hydrogen bond acceptors and the amine group as a hydrogen bond donor. In the FLT3 inhibitor of MA49 (MA68), the assigned features were the urea oxygen and phenyl ring of the ureido‐phenoxy moiety as a hydrogen bond acceptor and an aromatic group, respectively, while the nitrogen of the pyridine was allocated as a hydrogen bond acceptor. In both cases, the pharmacophore properties of the VHL ligand included the *R*‐hydroxyl group of the pyrrolidine as both a hydrogen bond donor and acceptor feature, along with the carbonyl oxygen and thiazole nitrogen serving as two hydrogen bond acceptors. MOE induced fit docking generated 1000 initial placement poses from which the top 100 poses based on the London dG scoring function were passed on to a refinement step. The refinement used the Generalized‐Born Volume Integral/Weighted Surface Area (GBVI/WSA) scoring function to retain the final 50 poses. GBVI/WSA is a force field‐based scoring function that determines the binding free energy (kcal/mol) of the docked compound from a given pose [62, 63]. This induced fit docking protocol demonstrated success in reproducing the GSK215 and MA49 poses in each ternary model with RMSD values below 2 Å.

### 5.7 MD Simulation

The dynamics of the VHL, FLT3, and FAK—ternary complexes (PDB IDs 5T35 and 7PI4) as well as the modeled FAK and FLT3 PROTAC ternary complex structures were checked by performing MD simulations for 500 ns. The Desmond simulation package was employed to set up the systems and run the MD simulations [64]. The systems were solvated using the TIP3P water model in a Periodic Boundary Conditions orthorhombic box of 10 Å and neutralized with either Na^+^ or Cl^‐^ ions [65]. To avoid the interference of ions in the interactions, they were excluded from placement within a distance of 15 Å from PROTAC atoms. For all the simulation runs, the OPLS4 force field, *NVT* and *NPT* (number of particles (N), volume (V), pressure (P) and temperature (T)) ensembles were utilized. Before performing the production simulation, the default Desmond protocol for energy minimization and model relaxation was applied. Initially, solvent molecules and ions were energy‐minimized while restraining the protein‐ligand complex, followed by minimization of the entire system. The system was then equilibrated in multiple stages for 12 ps per each stage: an initial *NVT* equilibration at 10 K with small restraints, followed by *NPT* equilibration with gradual restraint removal. A final unrestrained NPT equilibration ensured system stability before initiating the simulation production run. A cutoff of 9 Å was used to smoothly truncate the Lennard–Jones interactions and short‐range coulombic interactions. The Particle Mesh Ewald (PME) summation was used to calculate the long‐range electrostatic interactions [66]. Finally, 500 ns with a trajectory interval of 250 ps were carried out at a temperature of 300 K and a pressure of 1.01325 bar in the *NPT* ensemble using a Nose‐Hoover chain thermostat and a Martyna–Tobias–Klein barostat [67, 68]. The trajectories were saved at 2 fs intervals for further analysis using Simulation Interaction Diagram (SID) and Simulation Event Analysis (SEA) tools implemented in the Desmond MD package. SID was used to analyze the PROTAC interactions inside the protein binding pockets of the ternary complex structure. The geometric criteria for H‐bonds were acceptor‐donor maximum distance of 2.5 Å, donor minimum angle of 120, and acceptor minimum angle of 90 while those for π–stacking were centroid to centroid distance of 4.5 Å. SEA was used to monitor the stability of the complexes by examining Cα and PROTAC RMSD values over time using frame zero as a reference. PPIs (H‐bonds) were also monitored using SEA with geometric criteria of acceptor‐donor maximum distance of 2.5 Å, donor minimum angle of 120, and acceptor minimum angle of 90. To confirm the results of the MD simulations, each run was repeated twice at a different random seed (see Supporting Information).

### 5.8 Binding Free Energy Calculations

Binding free energy calculations were performed using the Molecular Mechanics Generalized Born Surface Area (MMGBSA) method to compare the protein–protein interaction energies [69]. Protein–protein snapshots per 1 ns from the final 40 ns of each MD trajectory were used for the calculation of the binding free energy. The target protein was considered as the receptor while the VHL was assigned as the ligand.

The binding free energies were calculated using the following equation:

$$\Delta G_{\text{BE}}=G_{\text{Binary}\;\text{complex}}–(G_{\text{Target}}+G_{\text{VHL}}).$$

The absolute free energy for each component of the equation is computed as a sum of the gas‐phase free energy *E*_MM_ plus the solvation free energy change ∆*G*_solvation_ according to the following equation:

$$G=E_{\text{MM}}+\Delta G_{\text{solvation}}.$$

The entropic contribution ‐*T*∆*S* was not considered [70]. Thus, the change of enthalpy (*E*_MM_ + ∆*G*_solvation_) was considered to indicate the binding free energy. Prime residue interaction energy was calculated for the whole binary complex (total energy), while MMGBSA was calculated for protein–protein interaction energy. Binary structures were solvated in a Variable Solvent Generalized Born (VSGB) solvation model and minimized using the OPLS4 force field, while other settings were kept as default.

## Conflicts of Interest

The authors declare no conflicts of interest.

## Supporting information

Supplementary_material_Revised.

## Data Availability

The data that support the findings of this study are available on request from the corresponding author. The data are not publicly available due to privacy or ethical restrictions.
