# Supplementary material for: Ternary Complex Modeling, Induced Fit Docking and Molecular Dynamics Simulations as a Successful Approach for the Design of VHL‐Mediated PROTACs Targeting the Kinase FLT3
Source: Arch Pharm (Weinheim). 2025 Apr 14;358(4):e3126. doi: 10.1002/ardp.202500102 (PMC11995253; doi:10.1002/ardp.202500102)
Supplement: Supplementary file 1 — Supplementary_material_Revised. [file ARDP-358-e3126-s001.docx]

**Supplementary information for:**

**Ternary Complex Modeling, Induced Fit Docking and Molecular Dynamics Simulations as a Successful Approach for the Design of VHL-mediated PROTACs Targeting the Kinase FLT3**

Husam Nassar^1^, Anne-Christin Sarnow^1^, Ismail Celik^1,2^, Mohamed Abdelsalam^1,3^, Dina Robaa^1^ and Wolfgang Sippl^1*^

^1^ Department of Medicinal Chemistry, Institute of Pharmacy, Martin-Luther University Halle-Wittenberg, Halle (Saale), Germany.

^2^Department of Pharmaceutical Chemistry, Faculty of Pharmacy, Erciyes University, Kayseri, Turkey

^3^Department of Pharmaceutical Chemistry, Faculty of Pharmacy, Alexandria University, Alexandria, Egypt

^*^Corresponding author: Wolfgang Sippl

Email: [wolfgang.sippl@pharmazie.uni-halle.de](mailto:wolfgang.sippl@pharmazie.uni-halle.de)

**Table S1:** Structures of the studied PROTACs with their targets and degradation activity.

| **PROTAC ID** | **PROTAC 2D structure** | **Target** | **Activity (nm)** |
| --- | --- | --- | --- |
| MZ1 |  | BRD4BD2 | DC_50_ < 2.5 |
| ABCI1 |  | SMARCA2 | DC_50_ < 32.0 |
| GSK215 |  | FAK | DC_50_ = 4.0 |
| PROTAC1 |  | FAK | DC_50_ = 23.2 |
| PROTAC2 |  | FAK | DC_50_ = 7.6 |
| PROTAC3 |  | FAK | DC_50_ = 3.0 |
| PROTAC4 |  | FAK | DC_50_ = 4.0 |
| PROTAC5 |  | FAK | DC_50_ = 20.8 |
| PROTAC6 |  | FAK | DC_50_ = 48.1 |
| WEE1 PROTAC |  | WEE1 | NA |
| MA49 |  | FLT3 | DC_50_ = 11.1 |
| FLT3 PROTAC |  | FLT3 | IC_50_ < 0.6 |

E2 ligase

Rbx1

**Cullin2**

EC

Ubiquitin

FAK

| 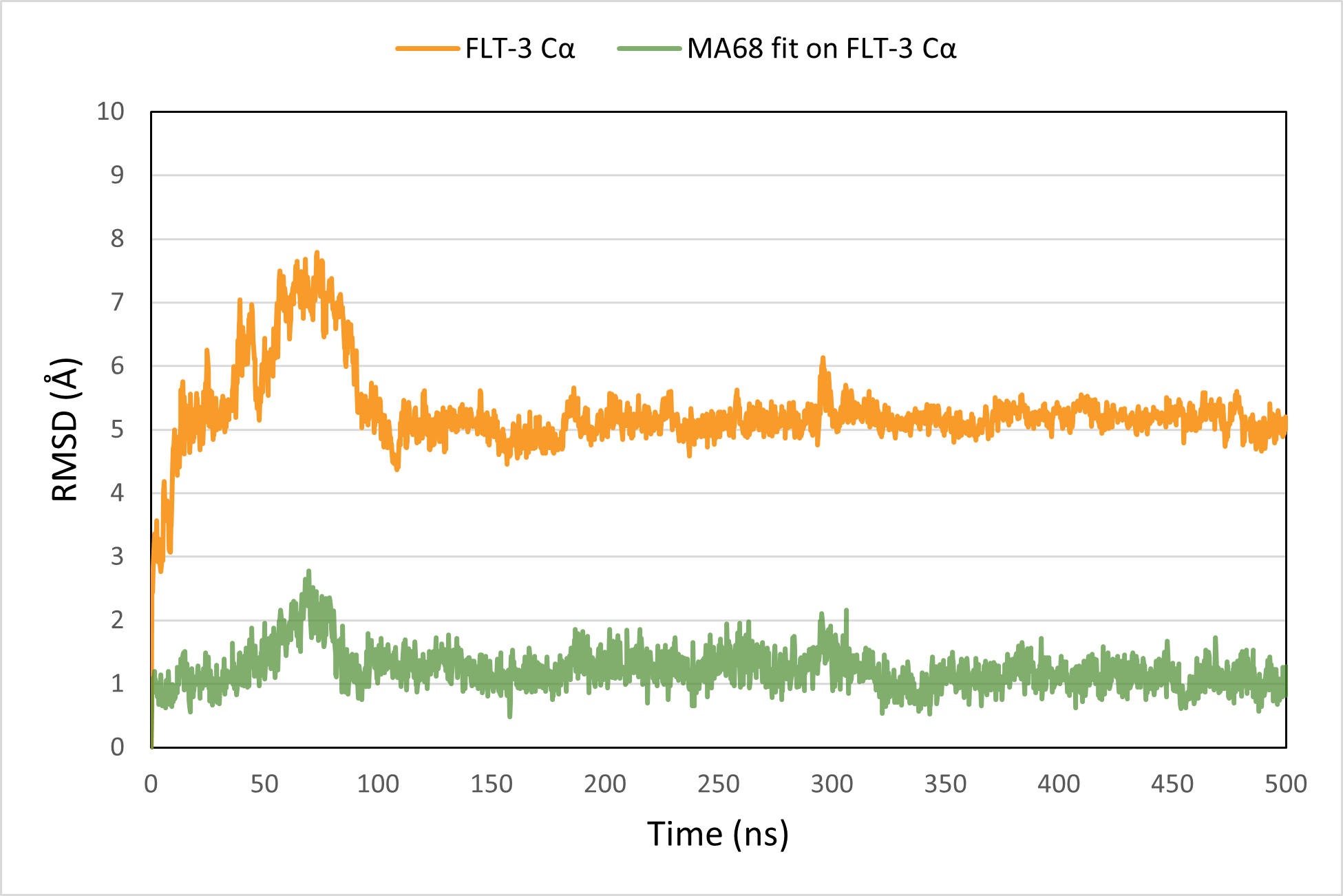 |
| --- |
| 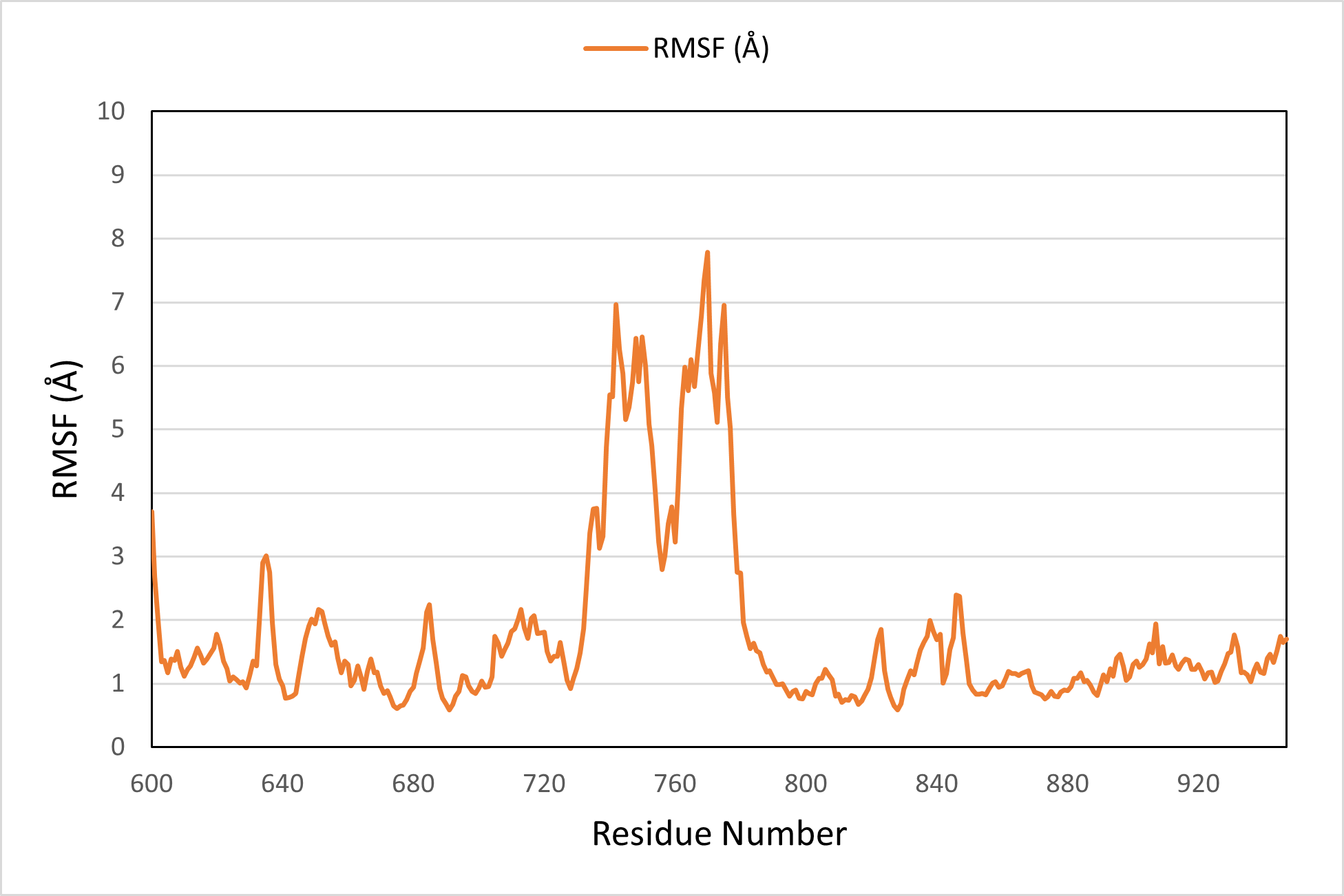 |
| 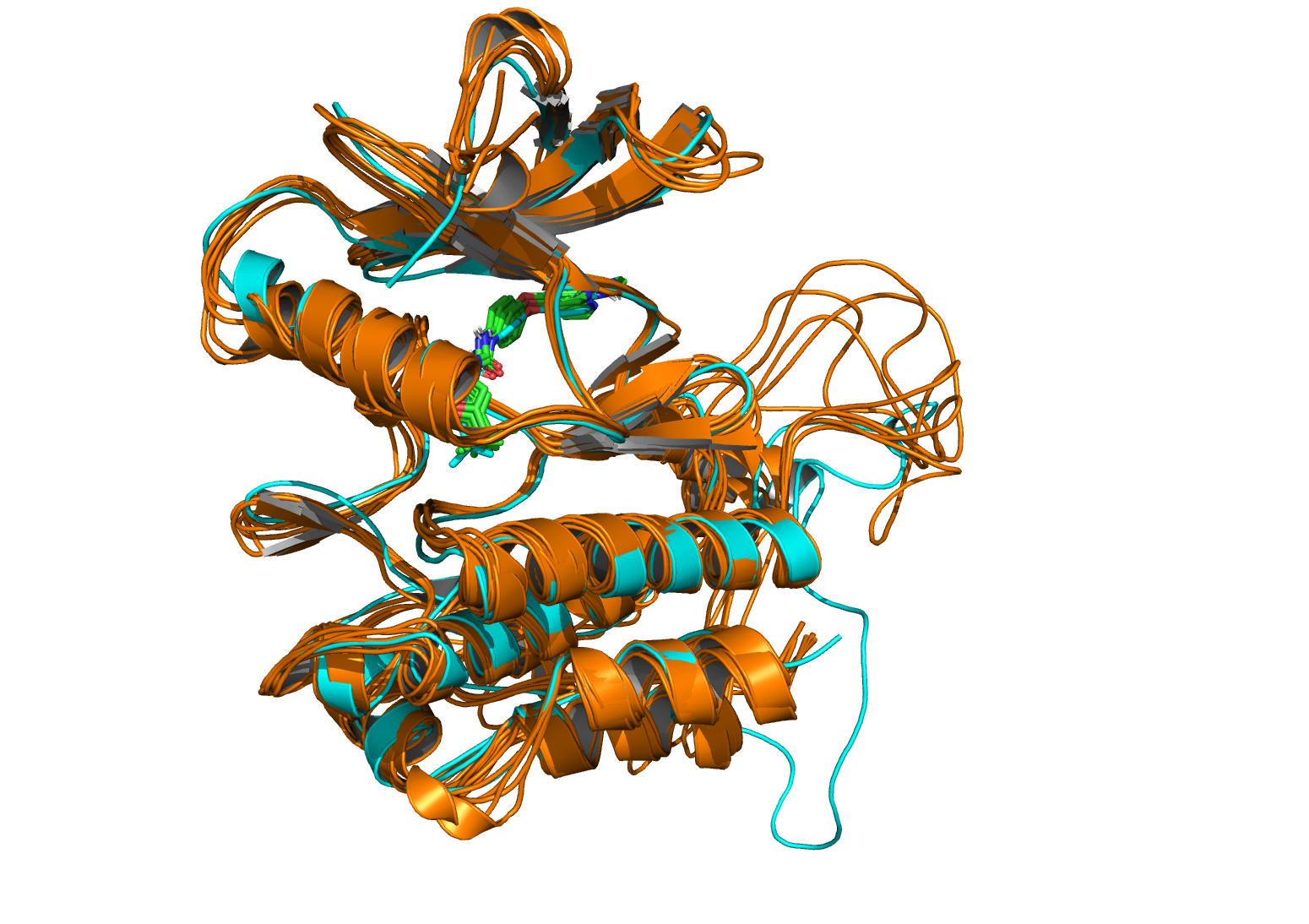 |

**Figure S1:** Analysis of 500 ns MD simulation of the modelled FLT3 ternary complex obtained by SWISS-MODEL. (A) RMSD values of the protein Cα (orange) and MA68 fitting on protein Cα (green). (B) RMSF values of the protein Cα (C) FLT3-MA68 complex conformations every 100 ns (orange) superimposed on the modelled structure before simulation production (cyan).

(C)

(B)

(A)

Residues 708-782

(A)

| 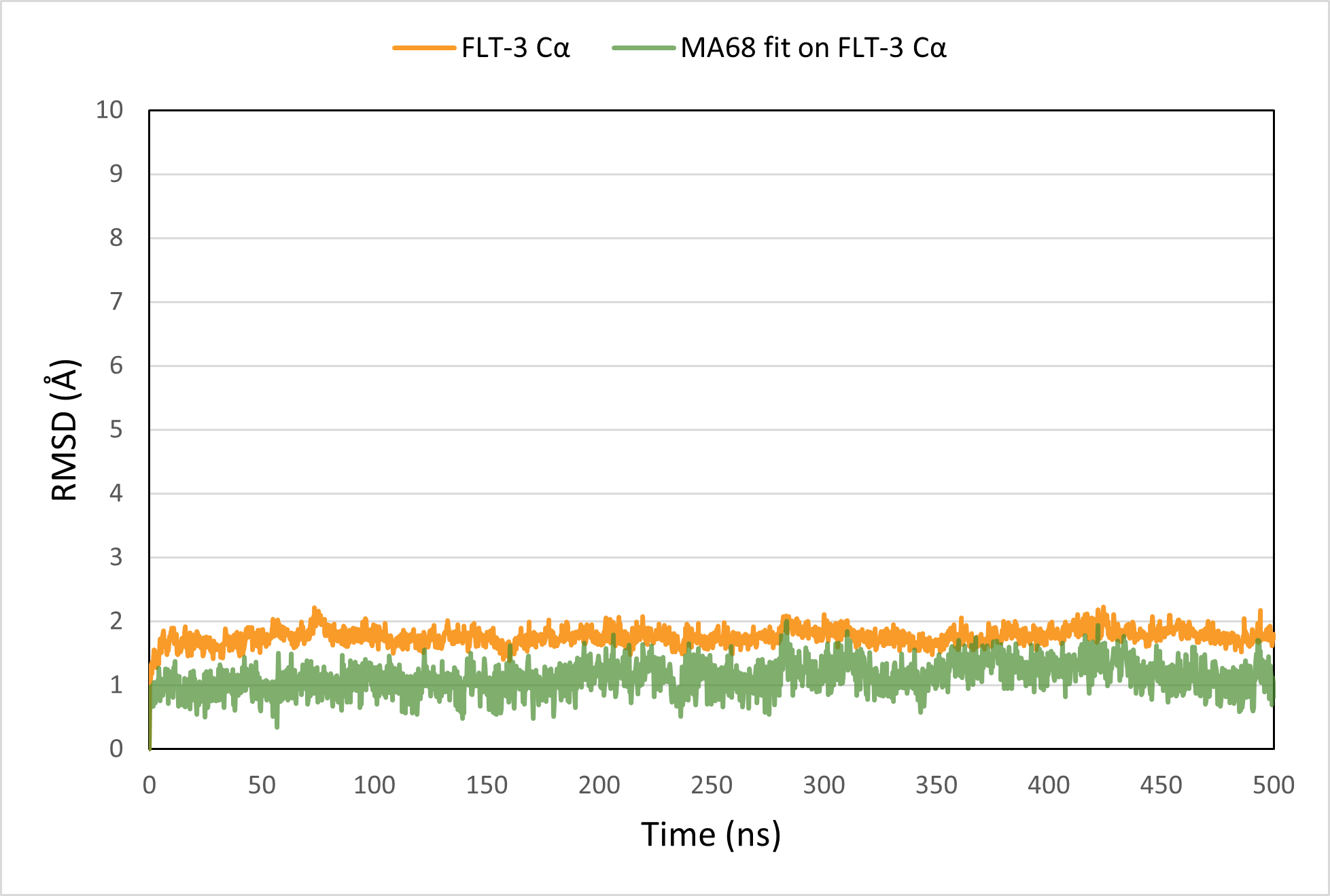 |
| --- |
| 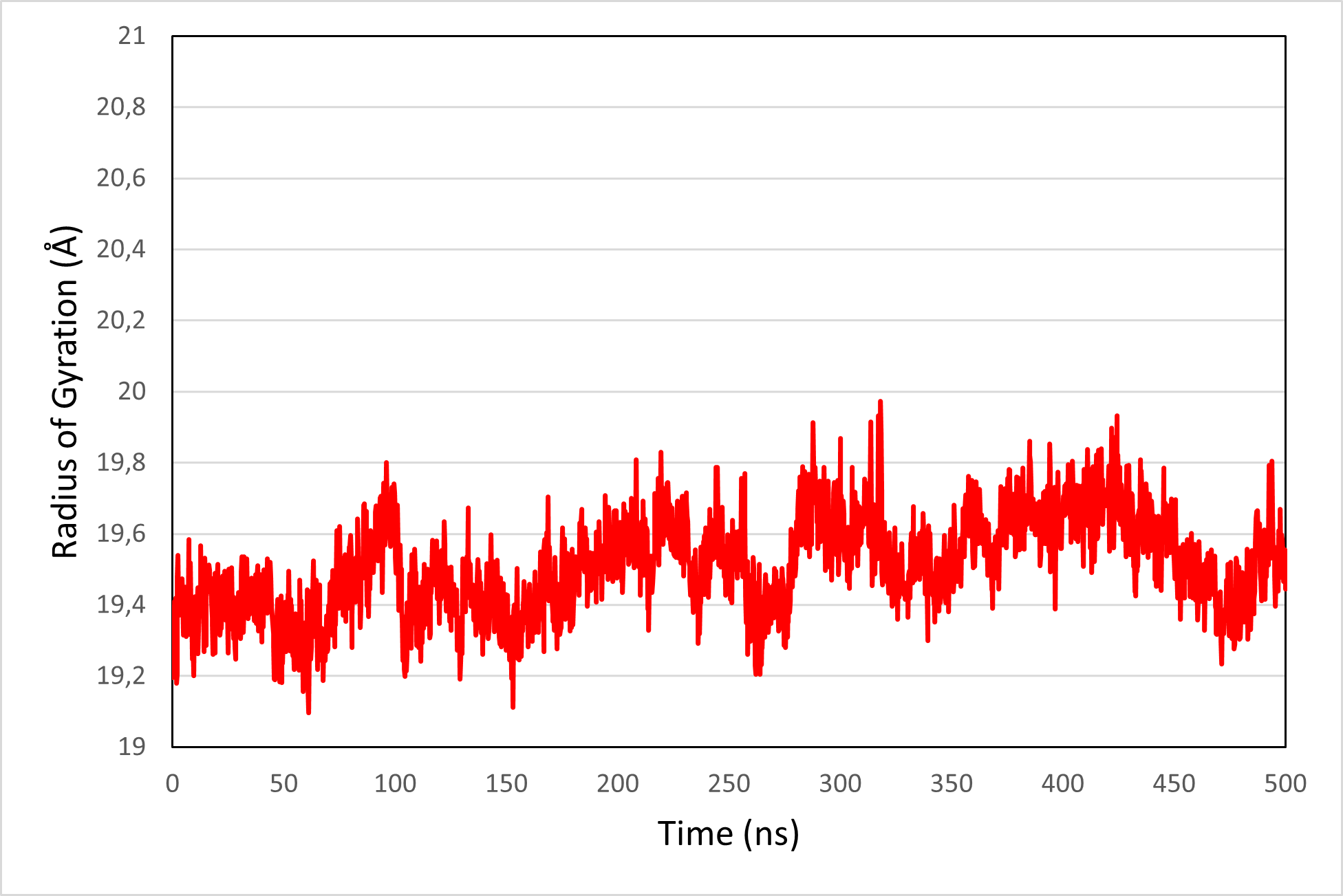 |
| 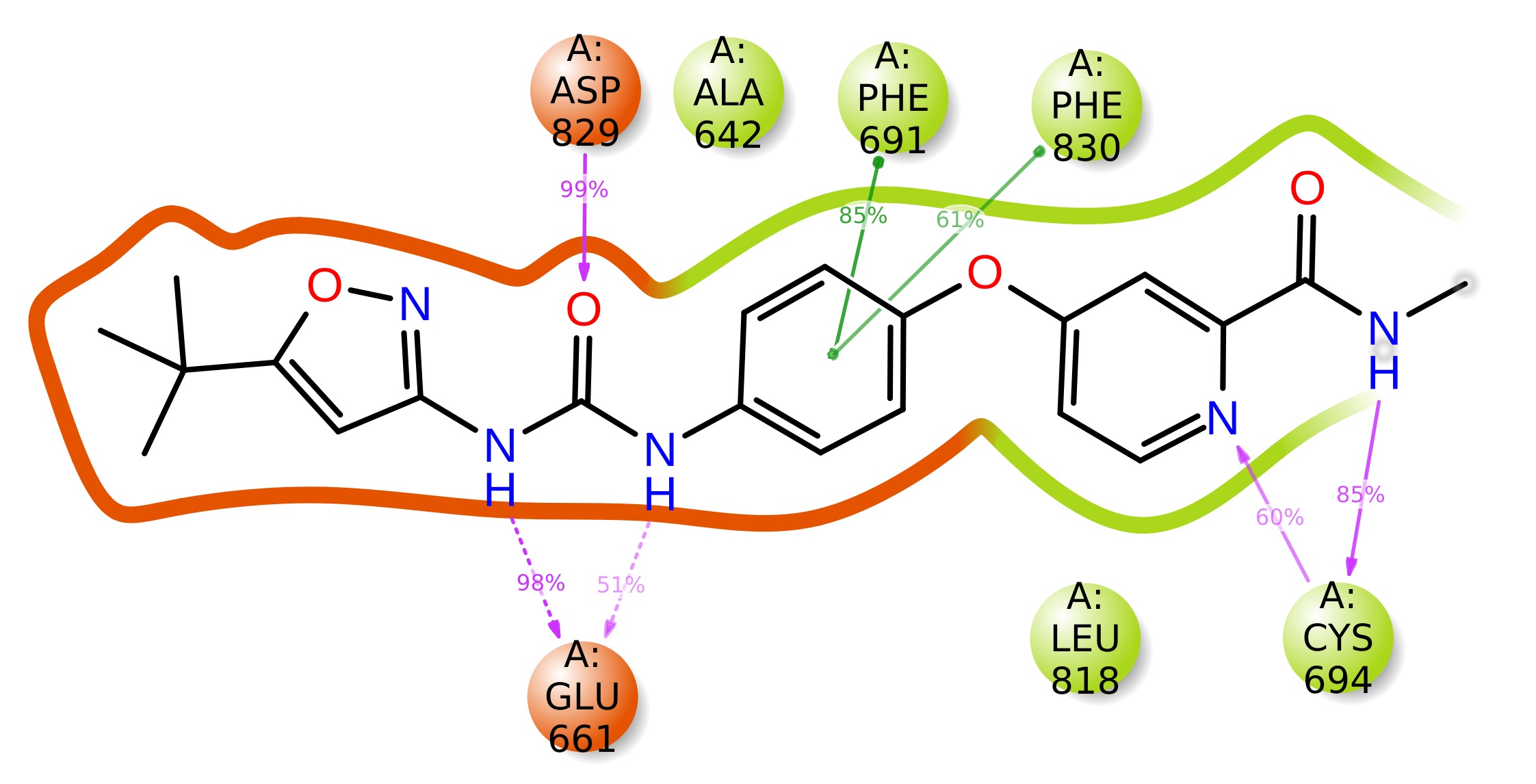 |

**Figure S2:** Analysis of 500 ns MD simulation of the modelled FLT3 ternary complex obtained by SWISS-MODEL, excluding the flexible loop of residues 708-782. (A) RMSD values of the protein Cα (orange) and MA68 fitting on protein Cα (green). (B) Radius of gyration the FLT3-MA68 complex (C) Schematic representation of detailed MA68 atom interactions with protein residues.

(C)

(B)

| 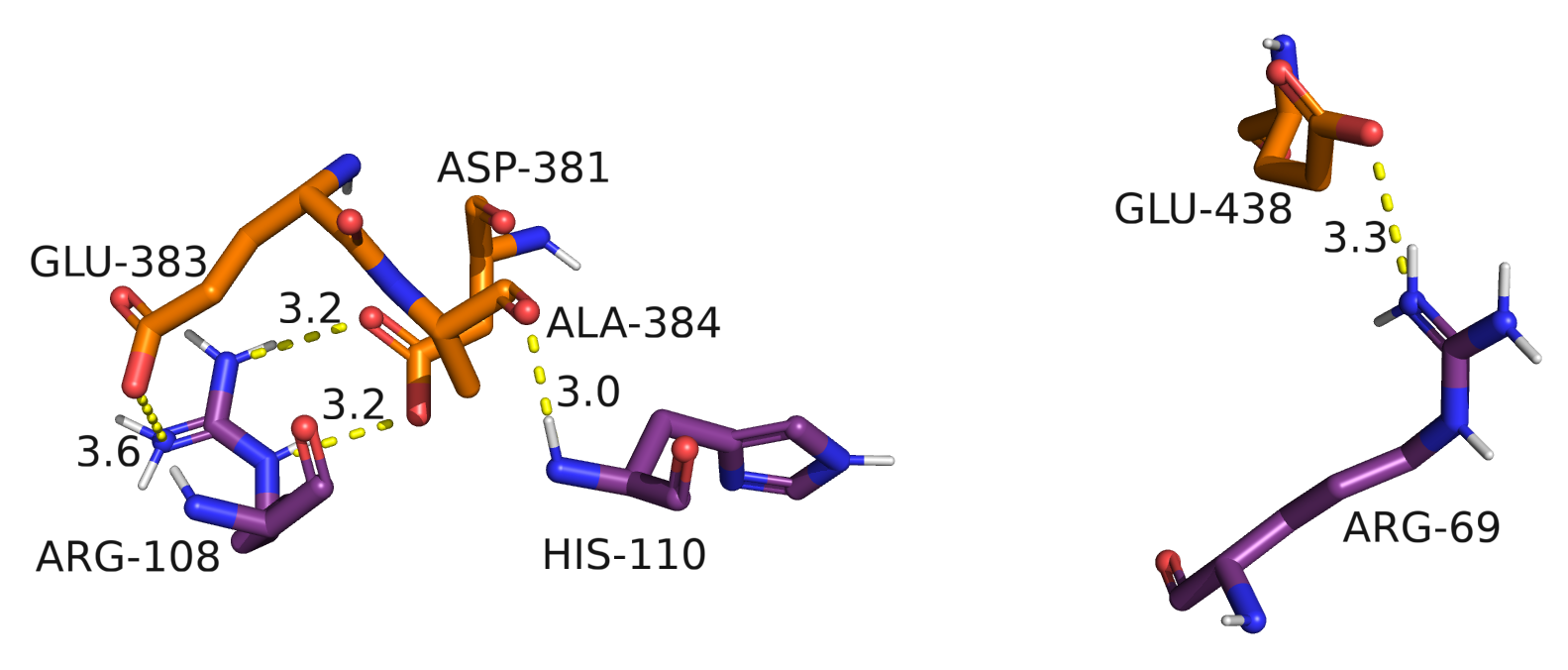 | 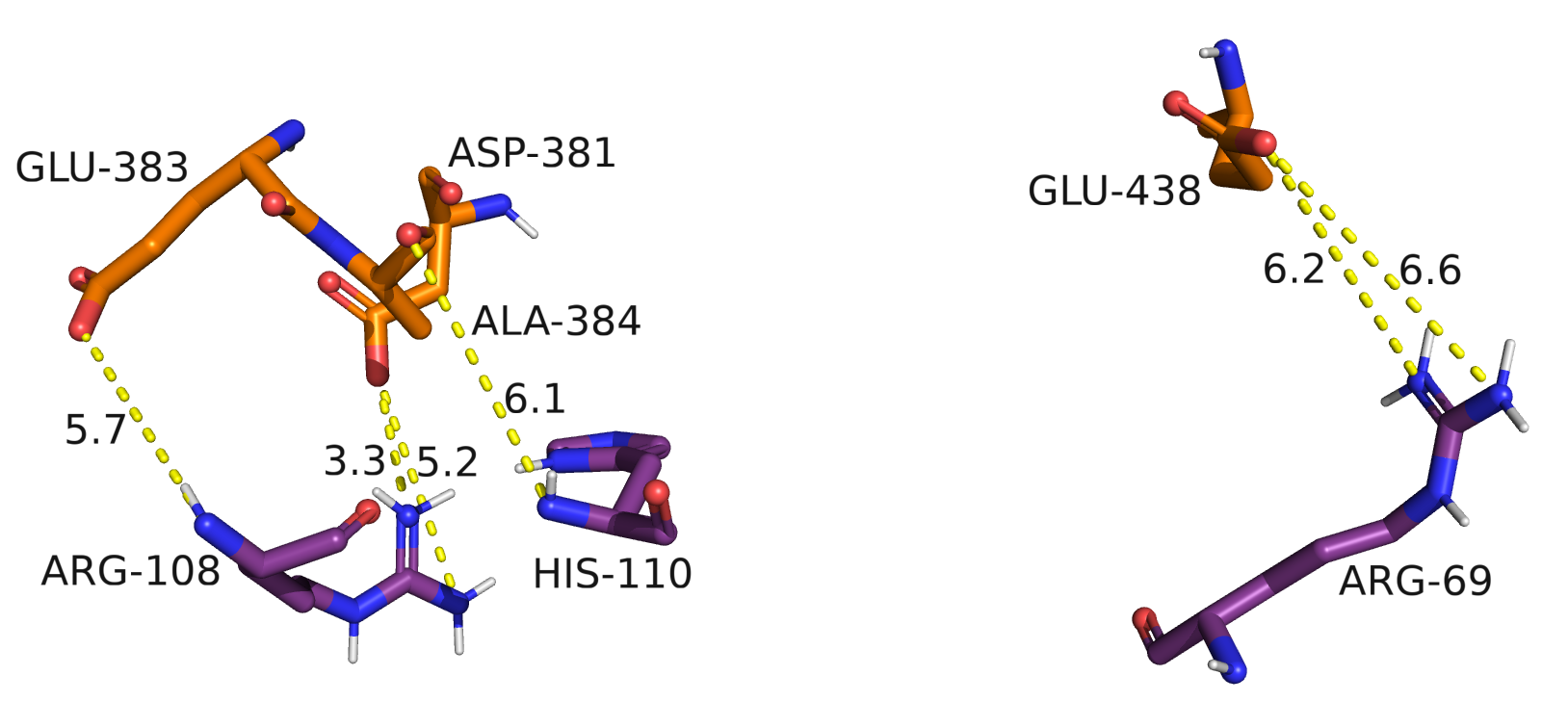 |
| --- | --- |
| 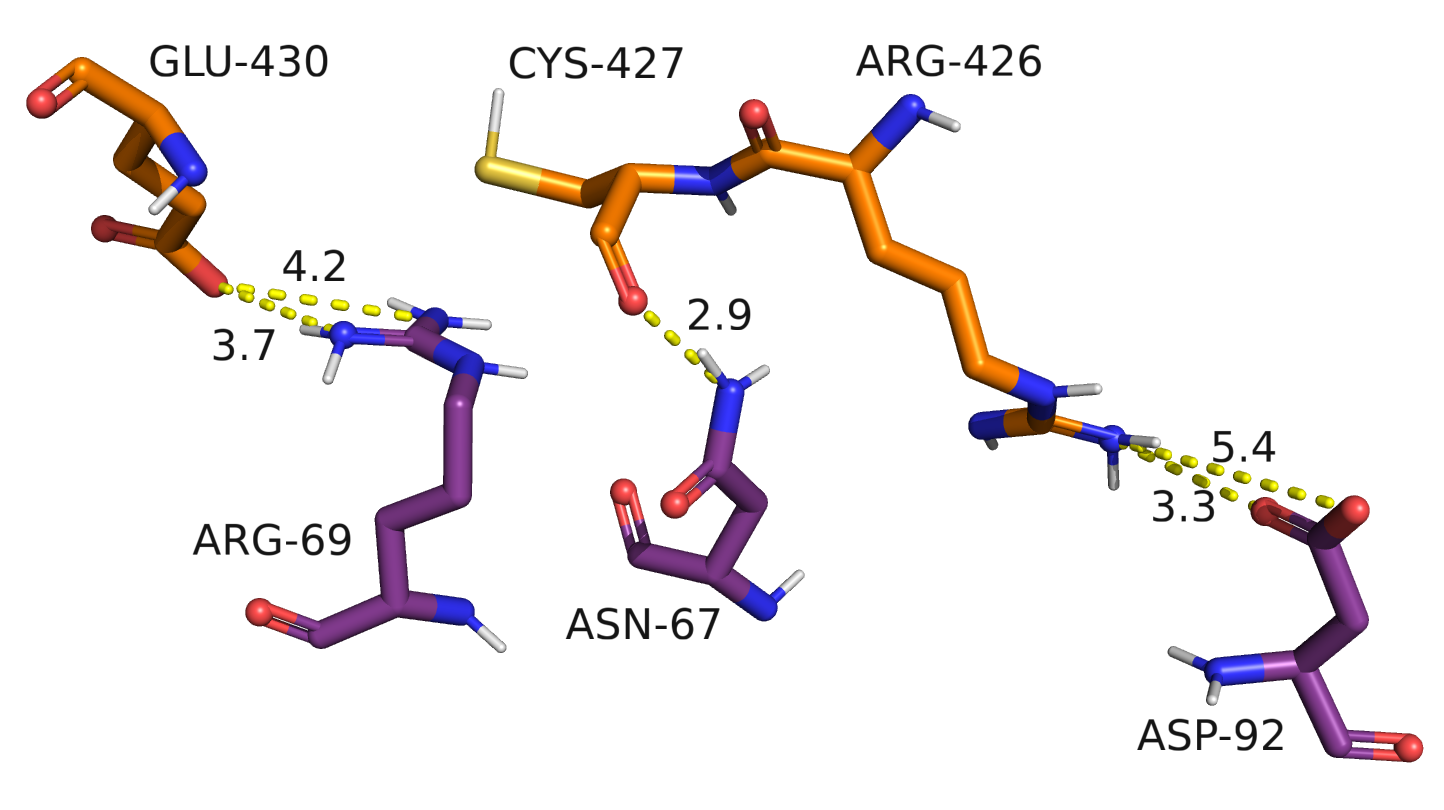 | 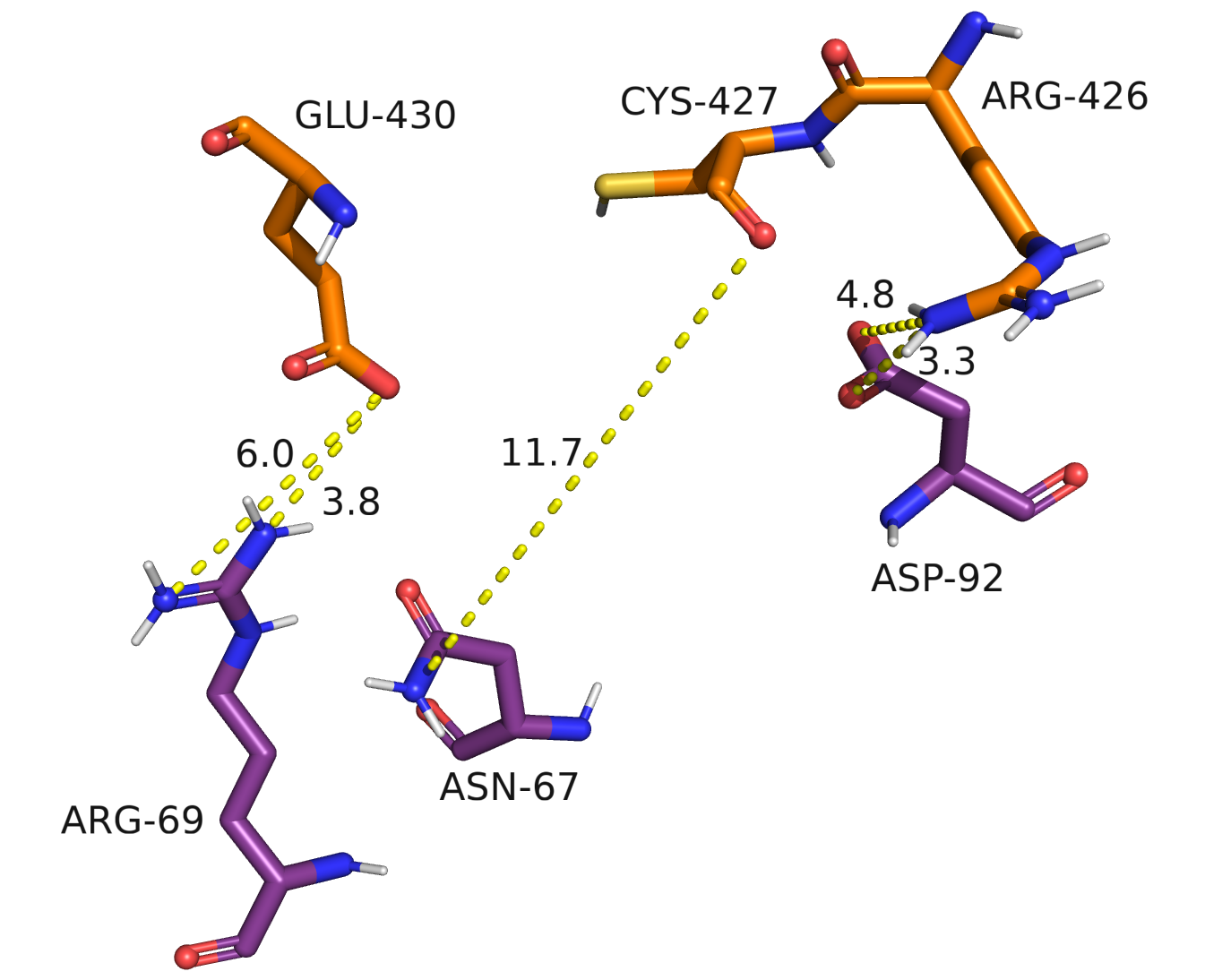 |
| 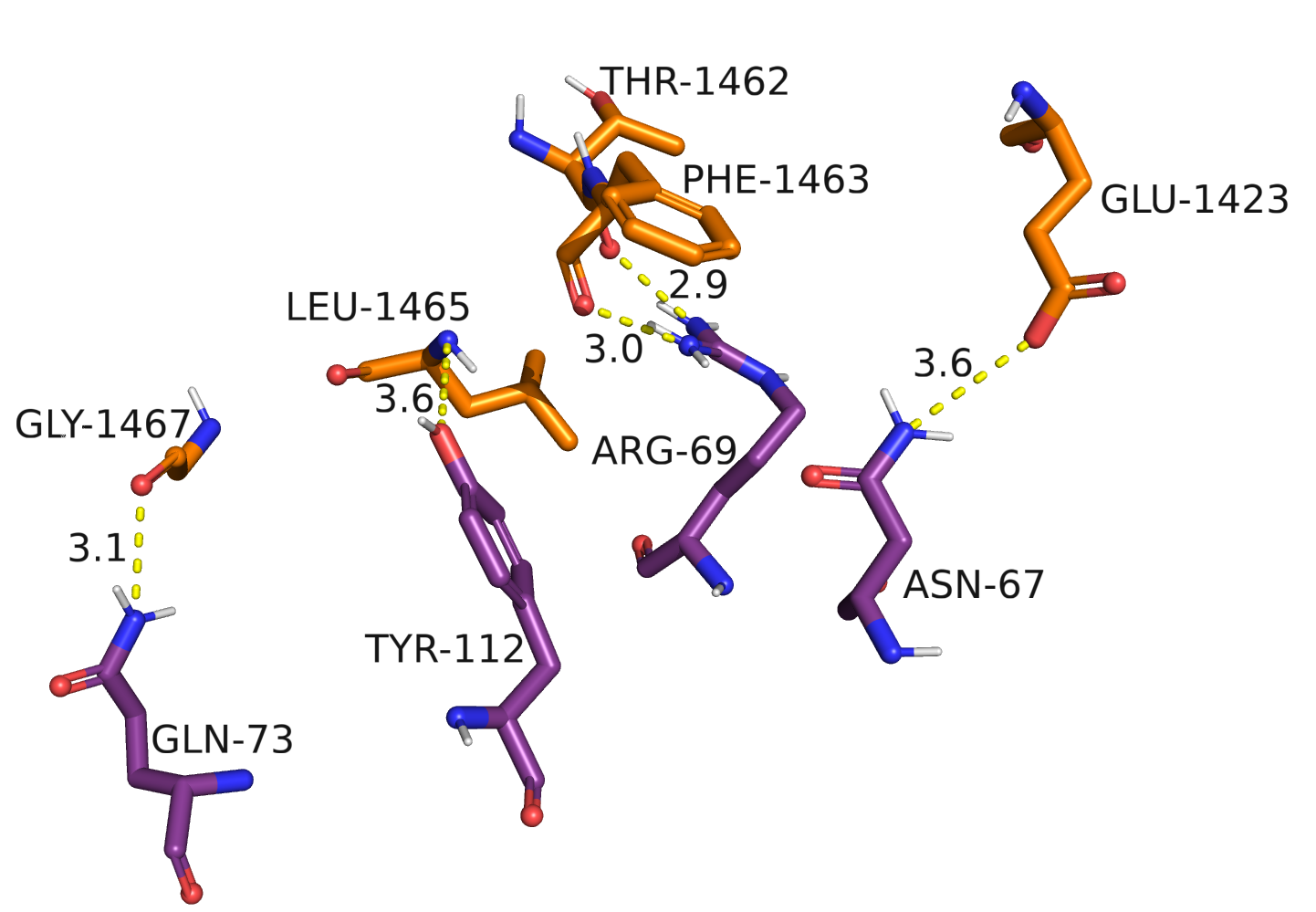 | 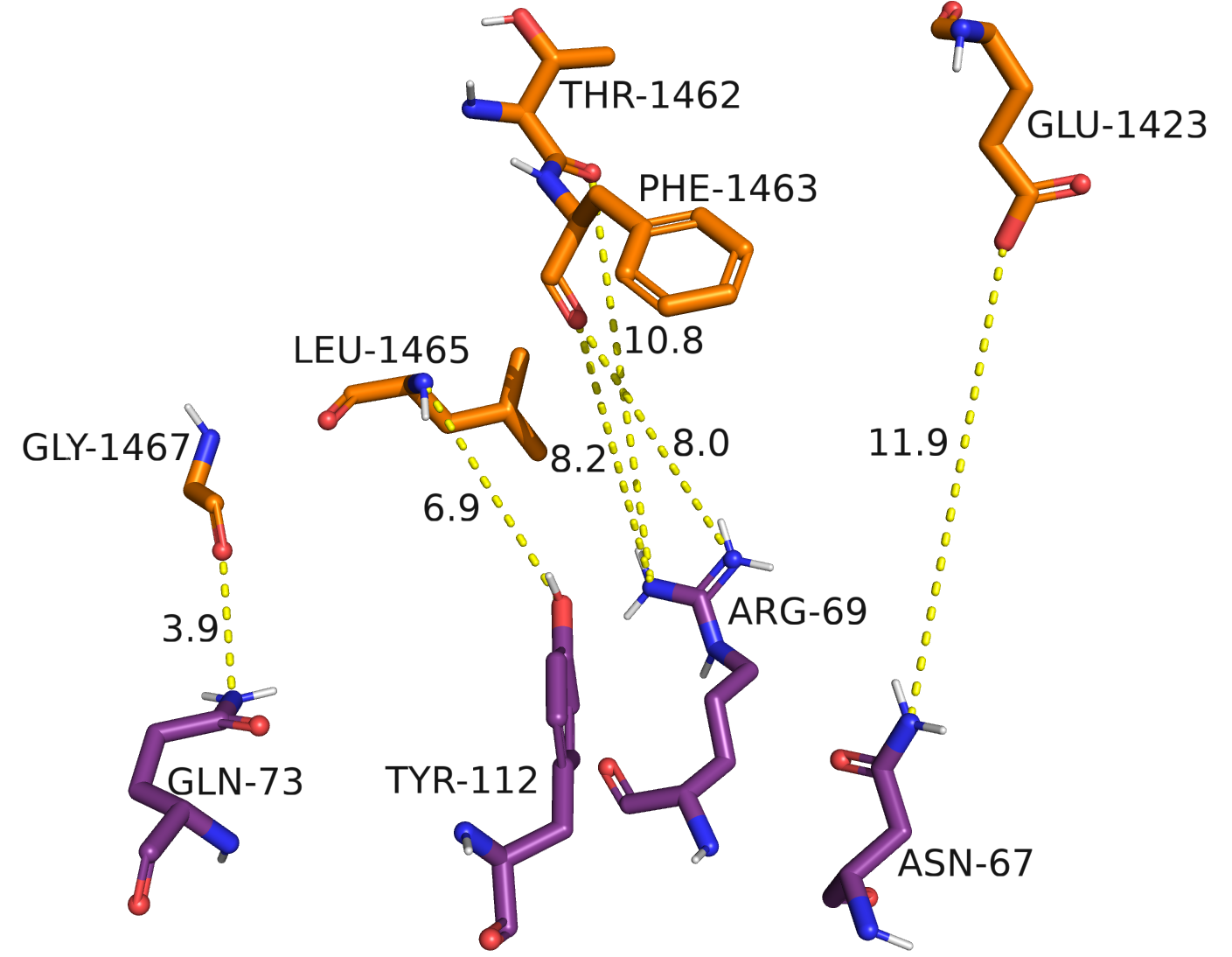 |
| 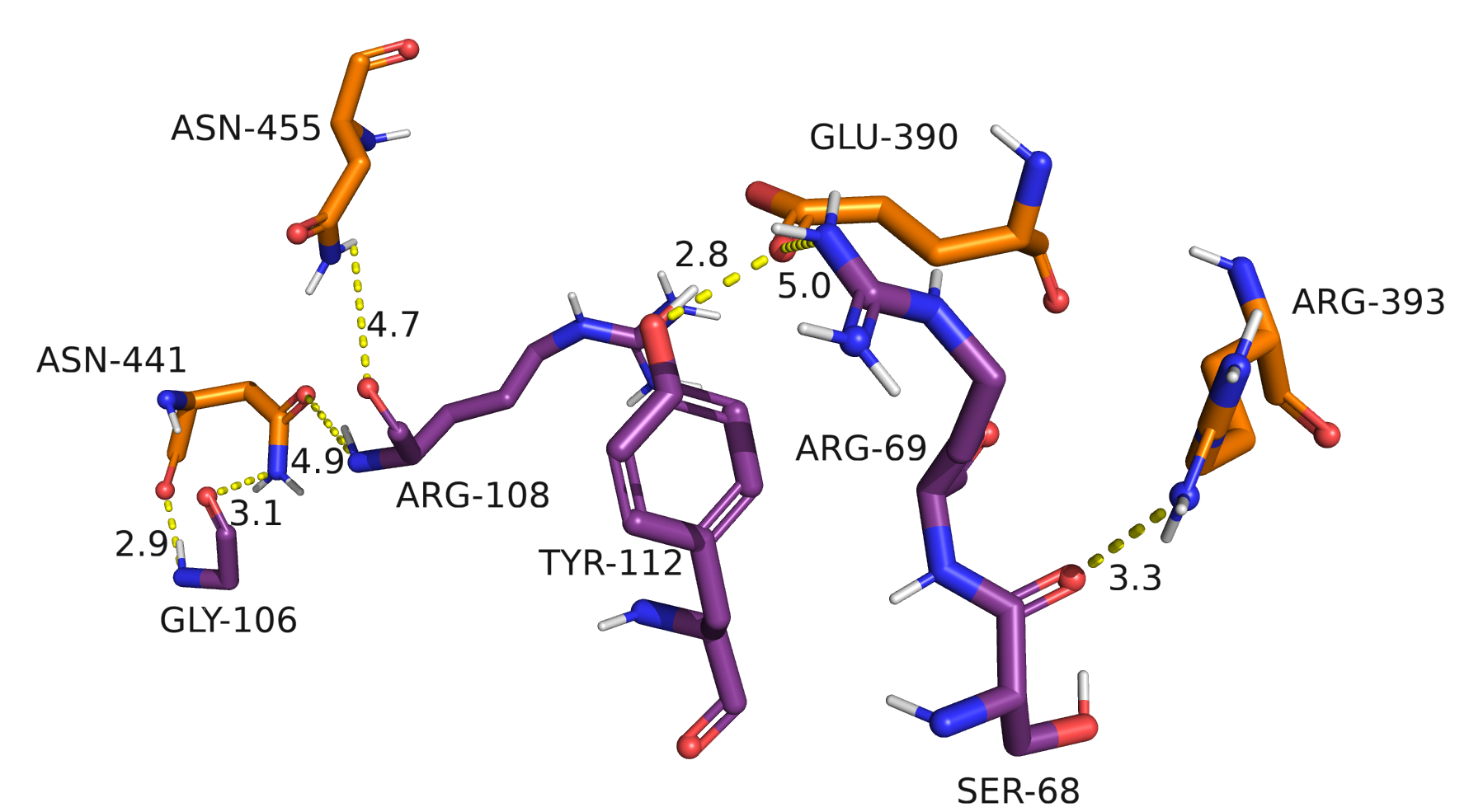 | 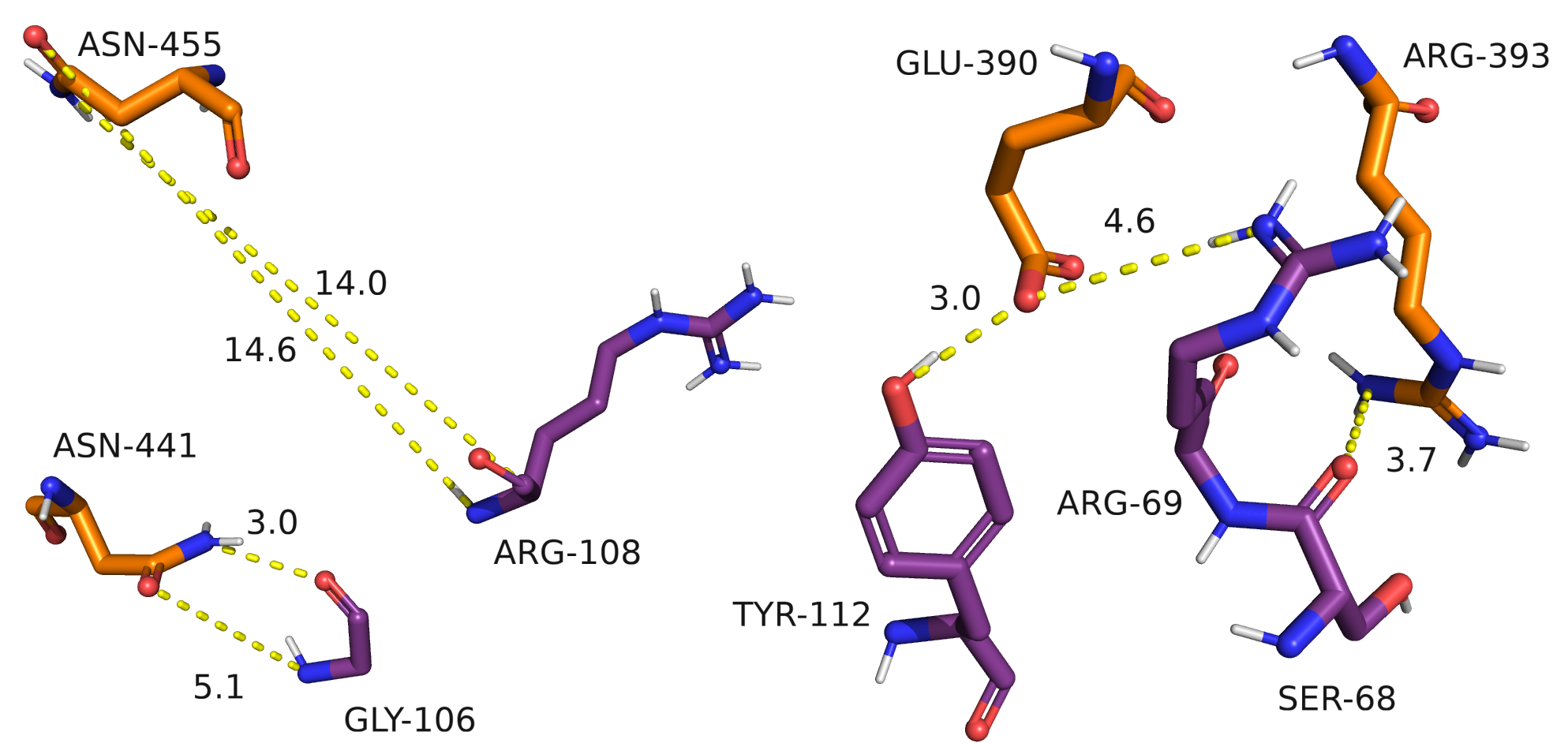 |

**Figure S3:** Comparison of PPIs between crystallized (left) and modeled (right) structures. (A) and (B) represent the 5T35 (BRD4BD2-MZ1-VHL) complex, (C) and (D) represent the 7S4E (SMARCA2-ABCI1-VHL) complex, (E) and (F) represent the 7PI4 (FAK-GSK215-VHL) complex and (G) and (H) represent the 8WDK (WEE1-AZD1775 PROTAC-VHL) complex. Target residues are colored orange while VHL residues are colored purple. Distances are indicated by yellow dashed lines.

(B)

(A)

(C)

(D)

(G)

(H)

(F)

(E)

(B)

(H)

(F)

(D)

(A)

(G)

(E)

(C)

| 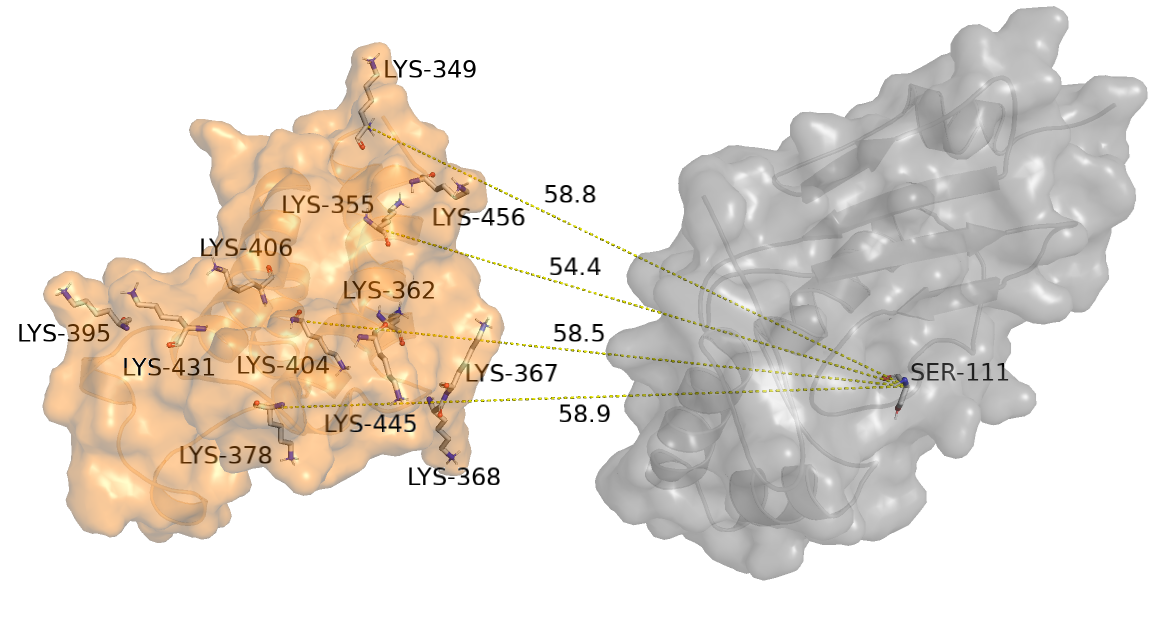 | 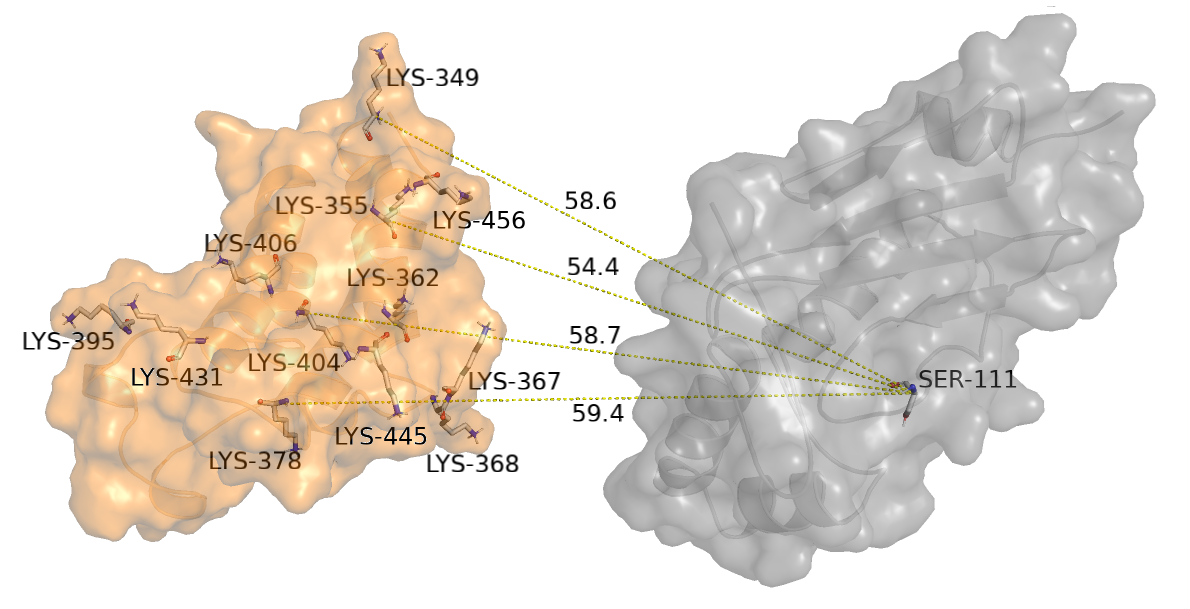 |
| --- | --- |
| 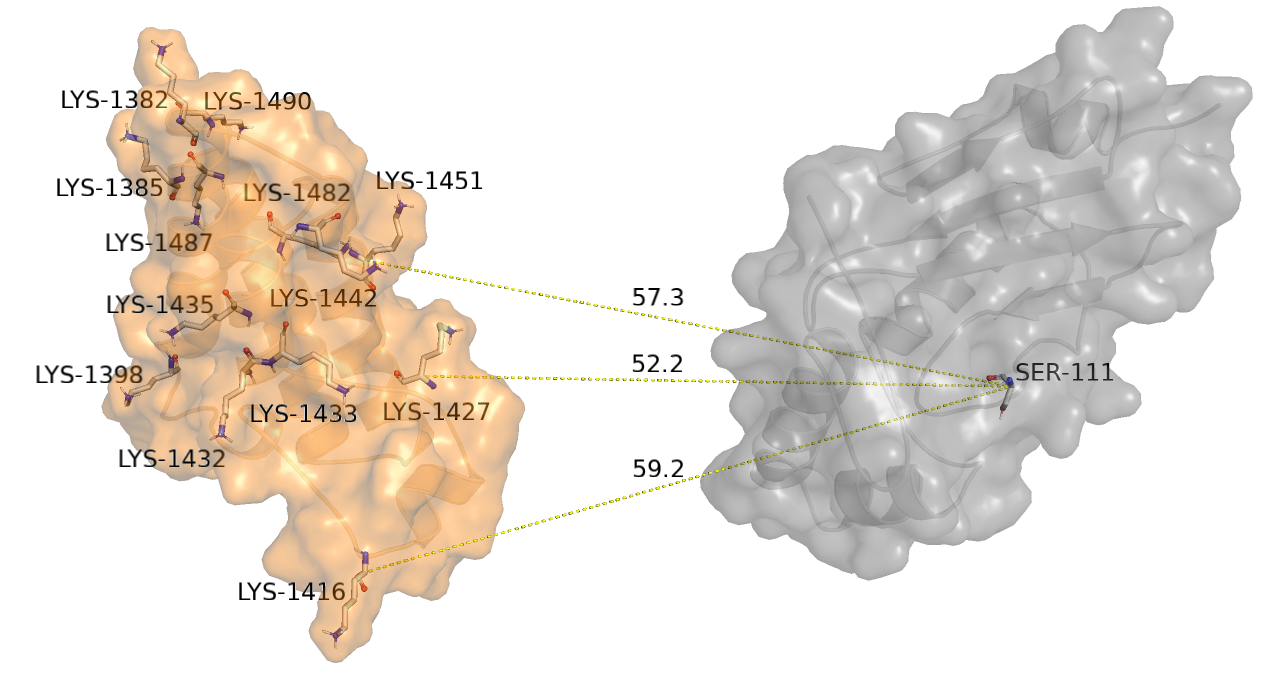 | 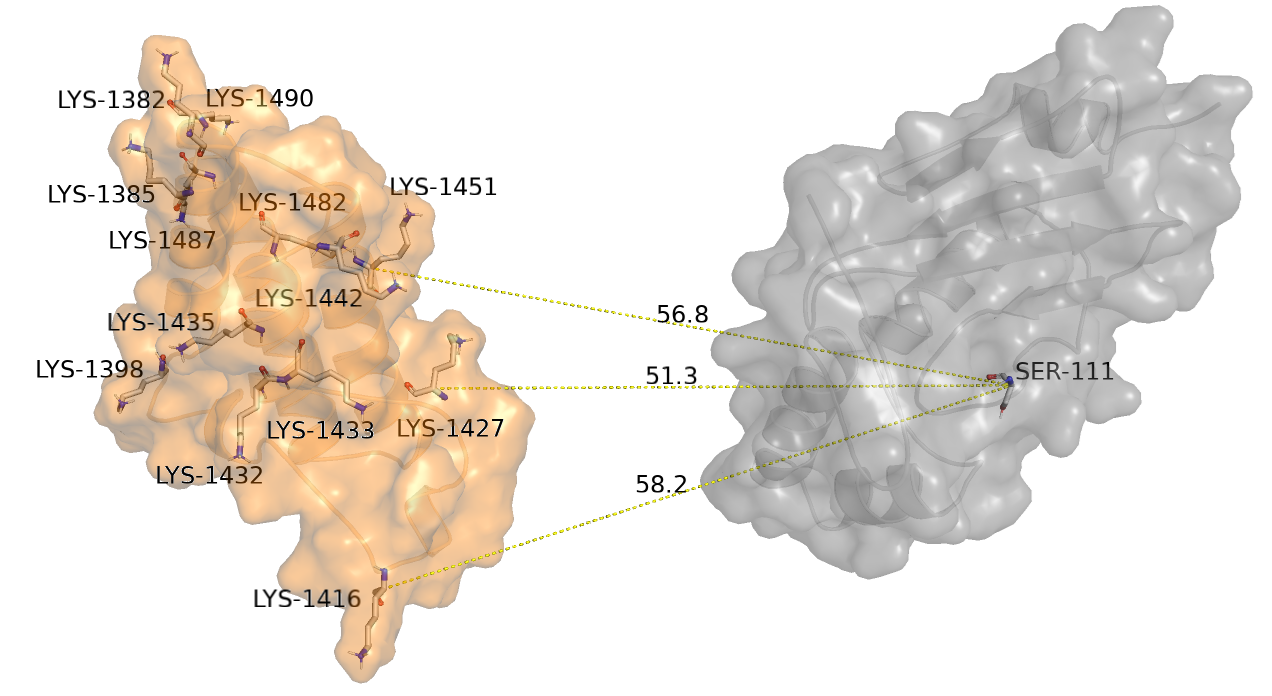 |
| 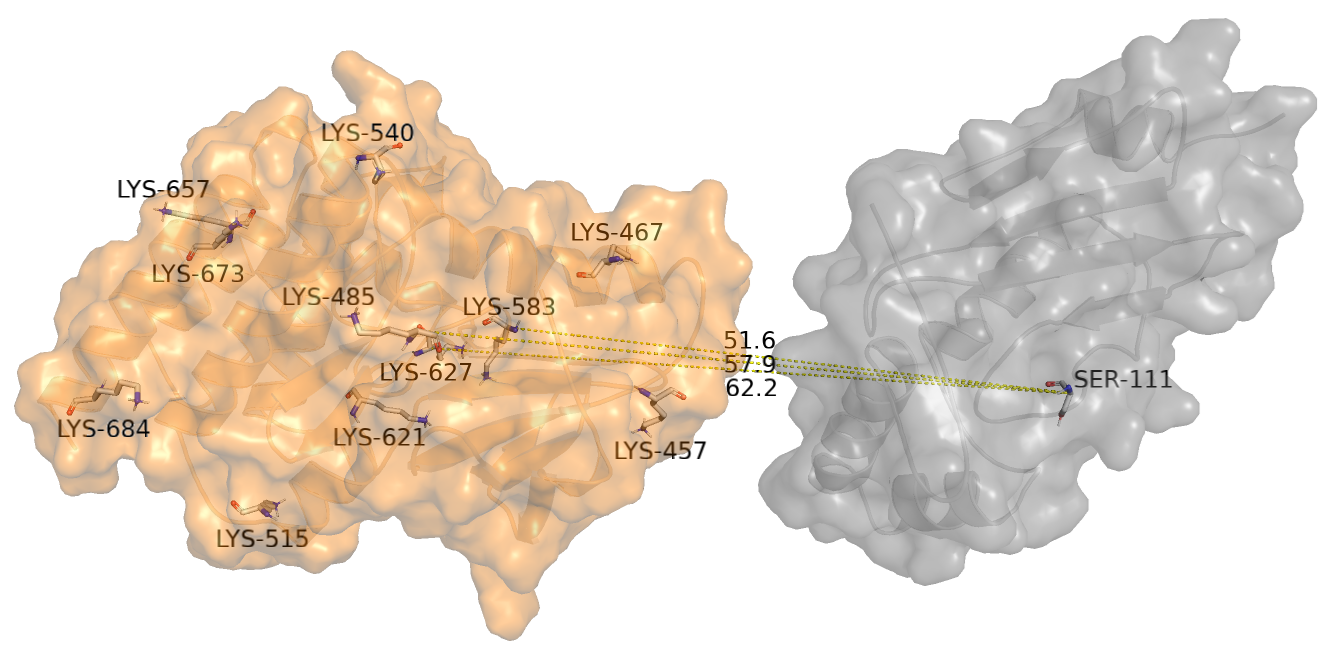 | 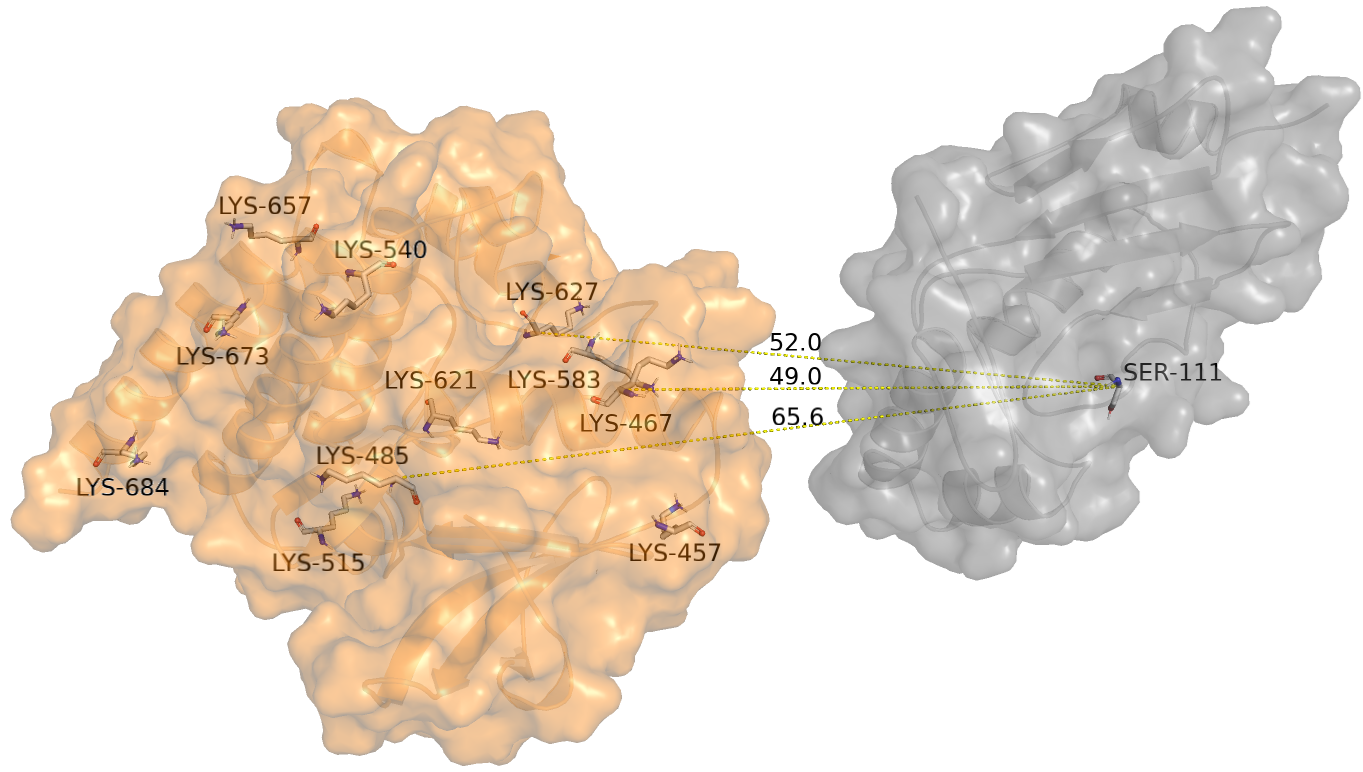 |
| 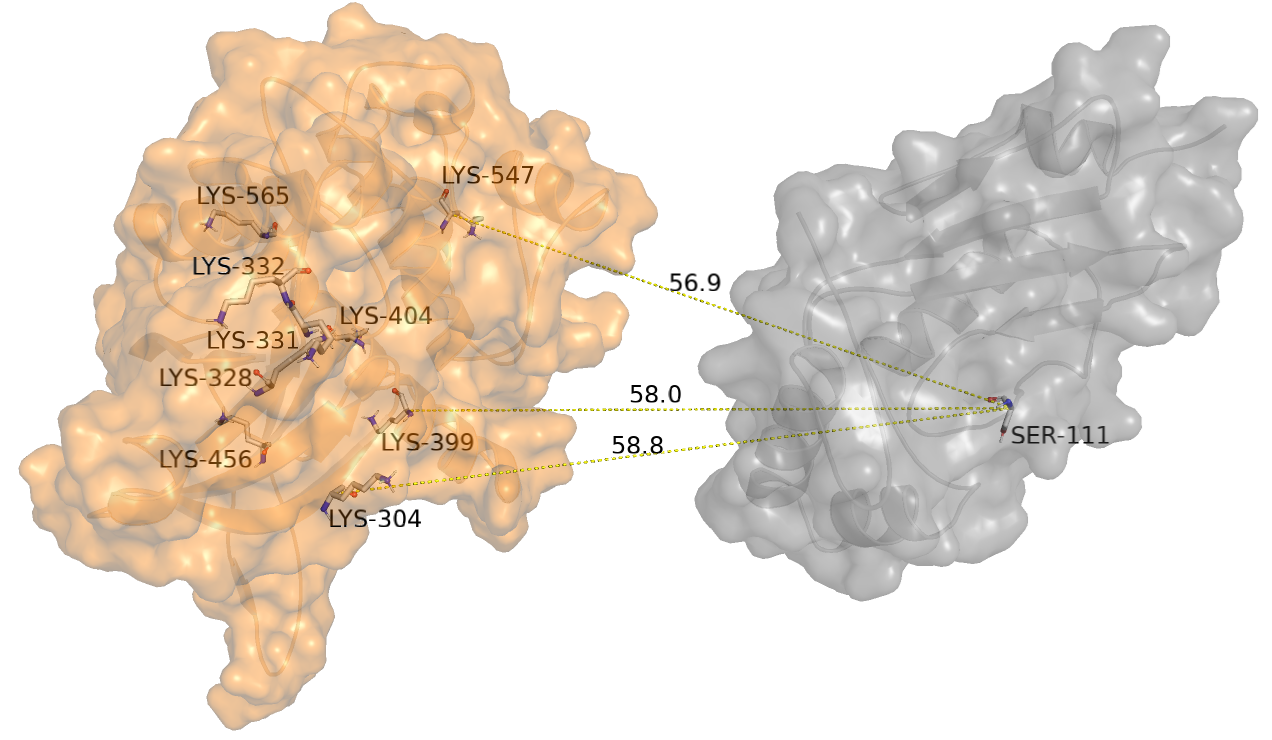 | 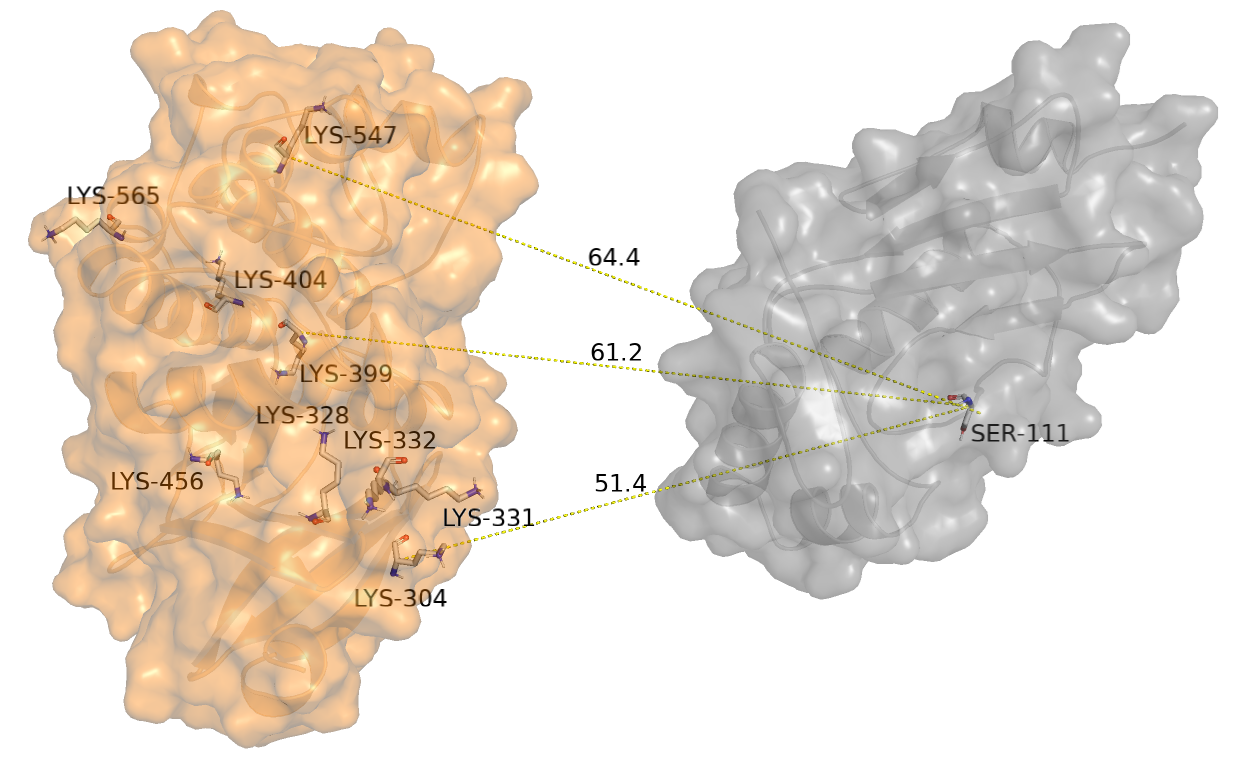 |

**Figure S4:** Comparison of calculated distances between solvent-exposed lysines of the target protein and Ser111 of E2 ligase in experimental (left) and modeled (right) structures. (A) and (B) represent the 5T35 (BRD4BD2-MZ1-VHL) complex, (C) and (D) represent the 7S4E (SMARCA2-ABCI1-VHL) complex, (E) and (F) represent the 7PI4 (FAK-GSK215-VHL) complex and (G) and (H) represent the 8WDK (WEE1-AZD1775 PROTAC-VHL) complex. Target protein is colored orange while E2 ligase is colored grey. Distances are indicated by yellow dashed lines.

(B)

(A)

| 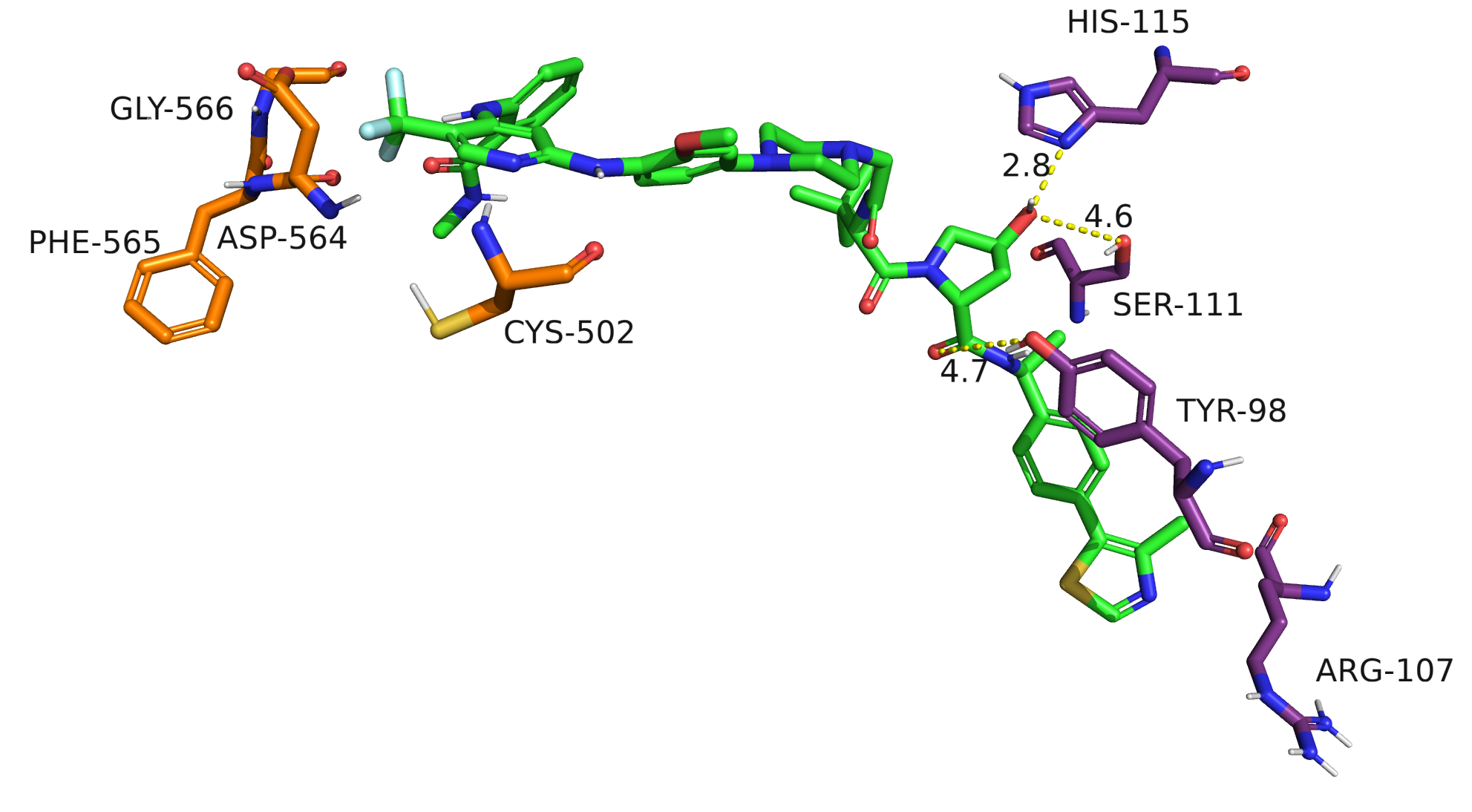 |
| --- |
| 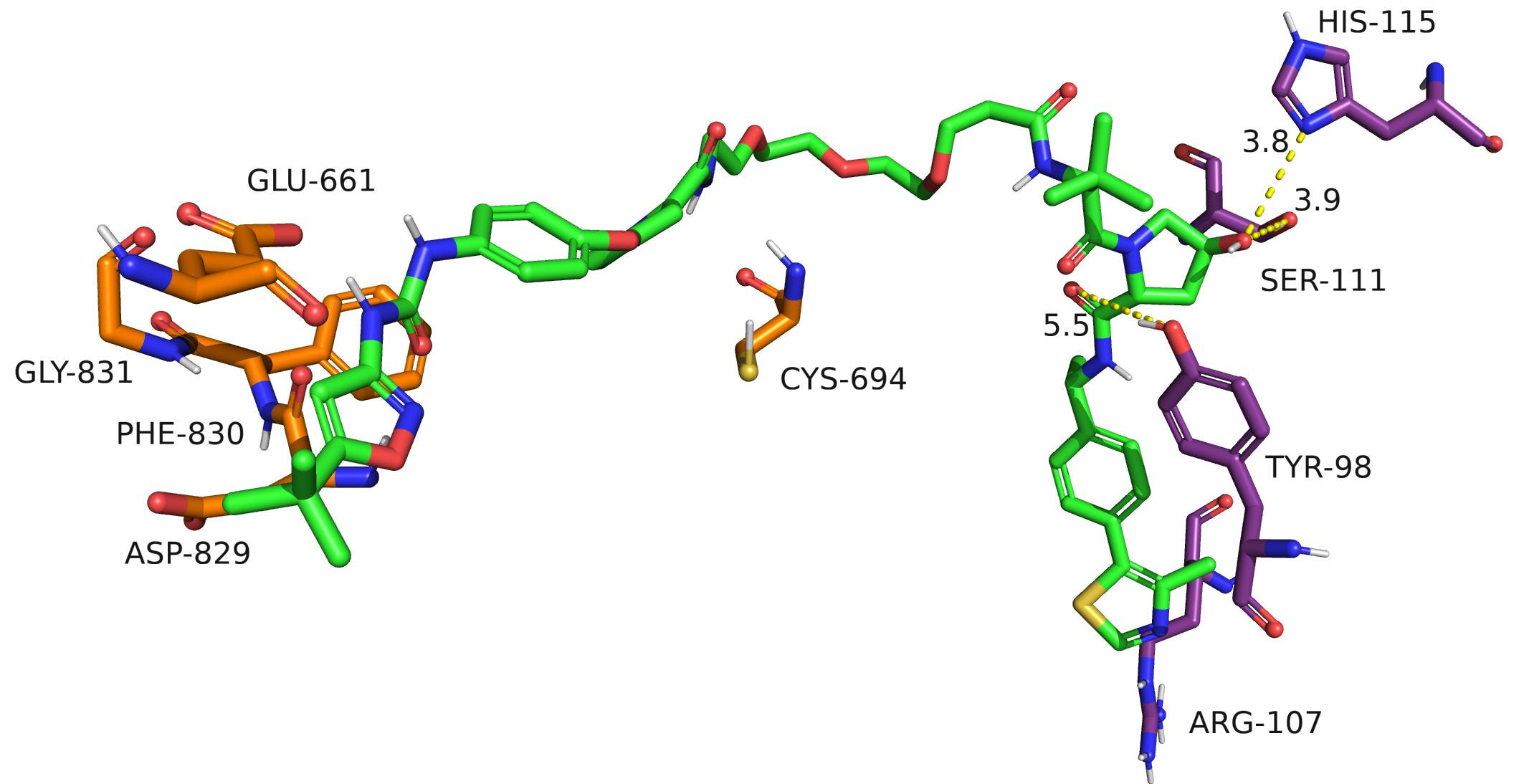 |

**Figure S5:** Detailed view of docking results for the negative controls of (A) GSK215 and (B) MA49 (MA72) within their respective ternary models. Distances are indicated by yellow dashed lines.

(B)

(A)

Residues 155-202

(A)

| 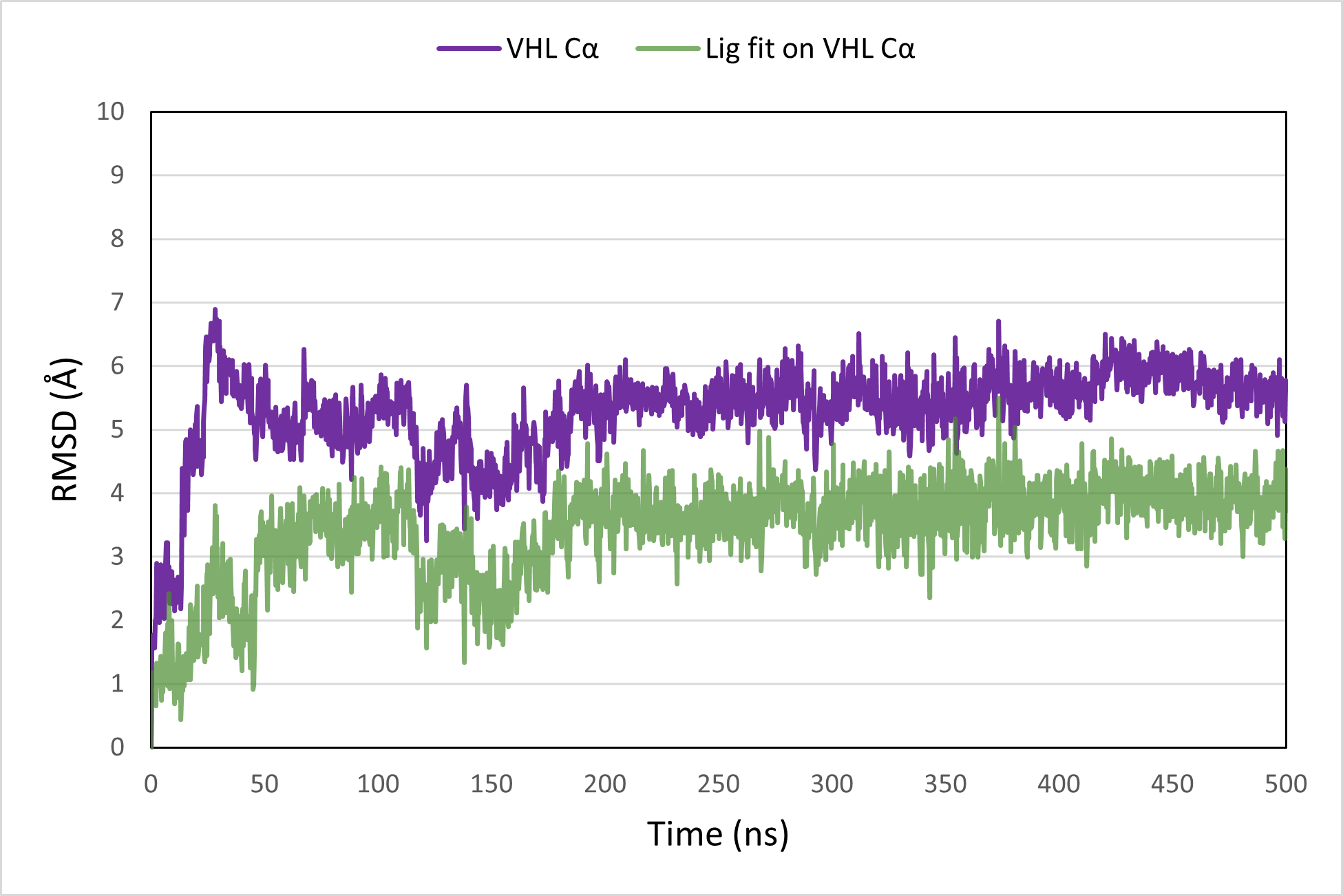 |
| --- |
| 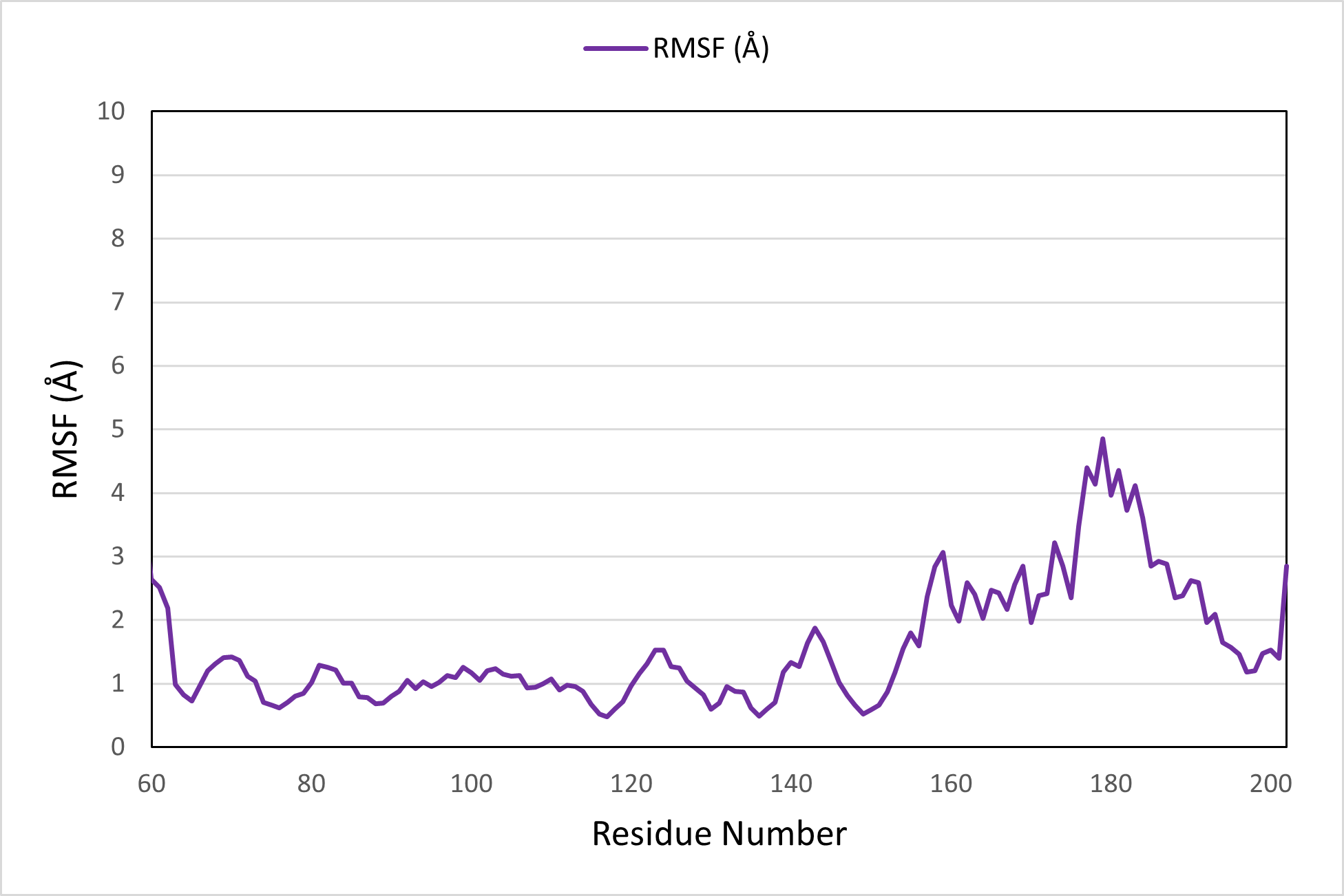 |
| 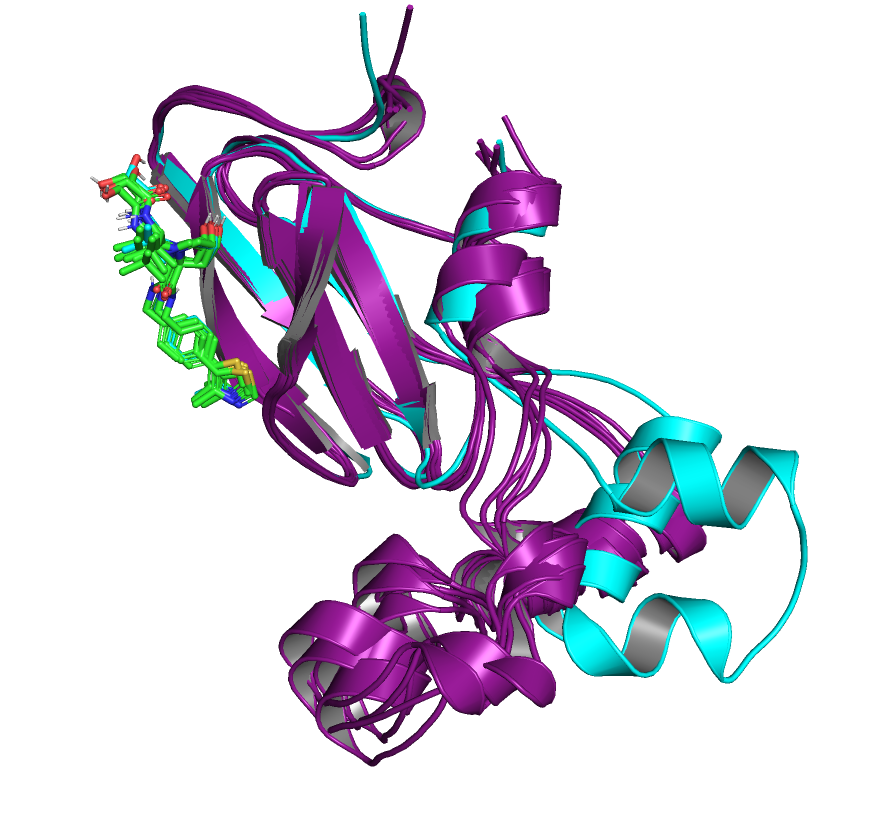 |

**Figure S6:** Analysis of 500 ns MD simulation of the crystal VHL structure (PDB ID: 5NVV). (A) RMSD values of the protein Cα (purple) and ligand 3 fitting on protein Cα (green). (B) RMSF values of the protein Cα. (C) VHL-ligand 3 complex conformations every 100 ns (purple) superimposed on the crystal structure before simulation production (cyan).

(C)

(B)

| 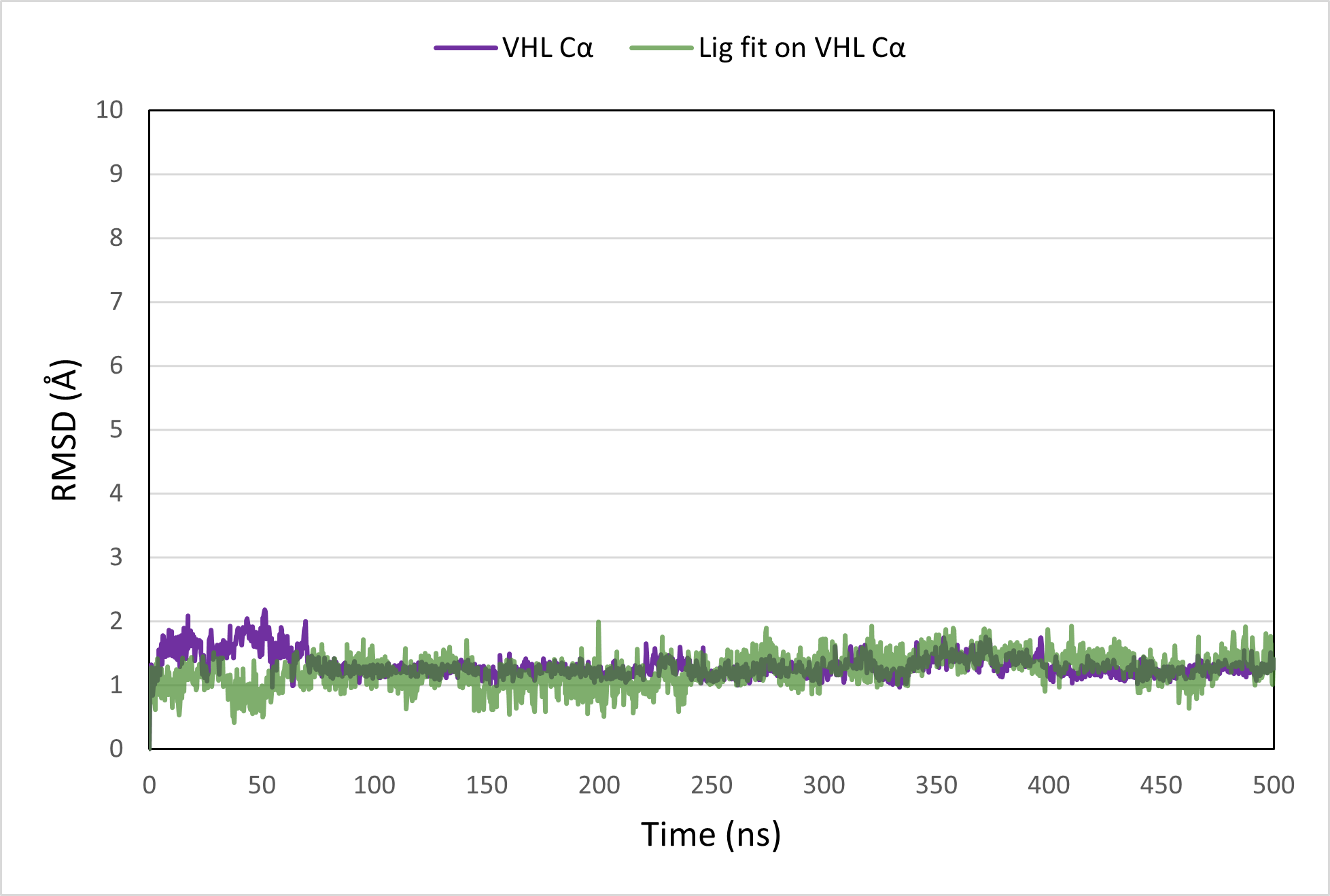 |
| --- |
| 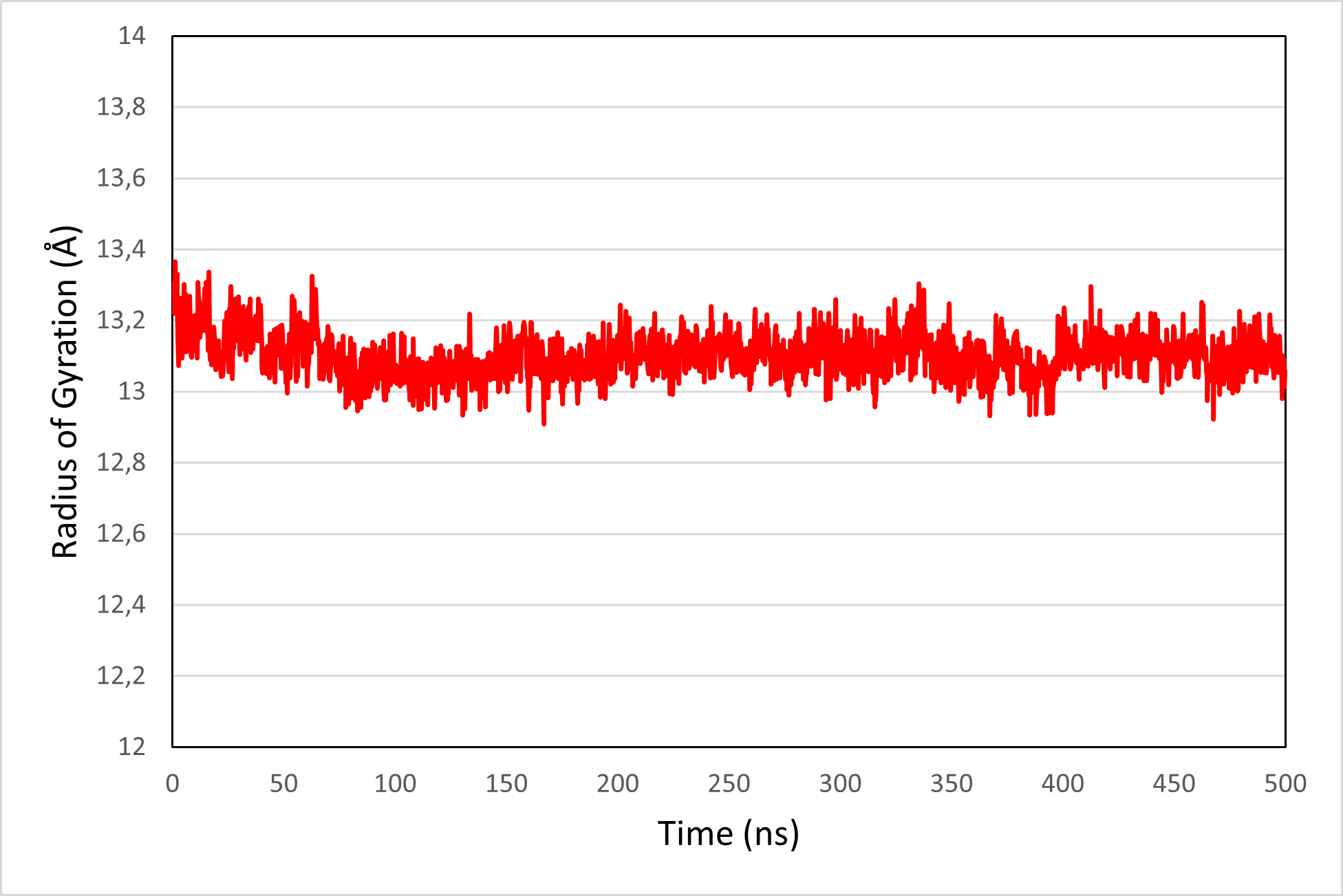 |
| 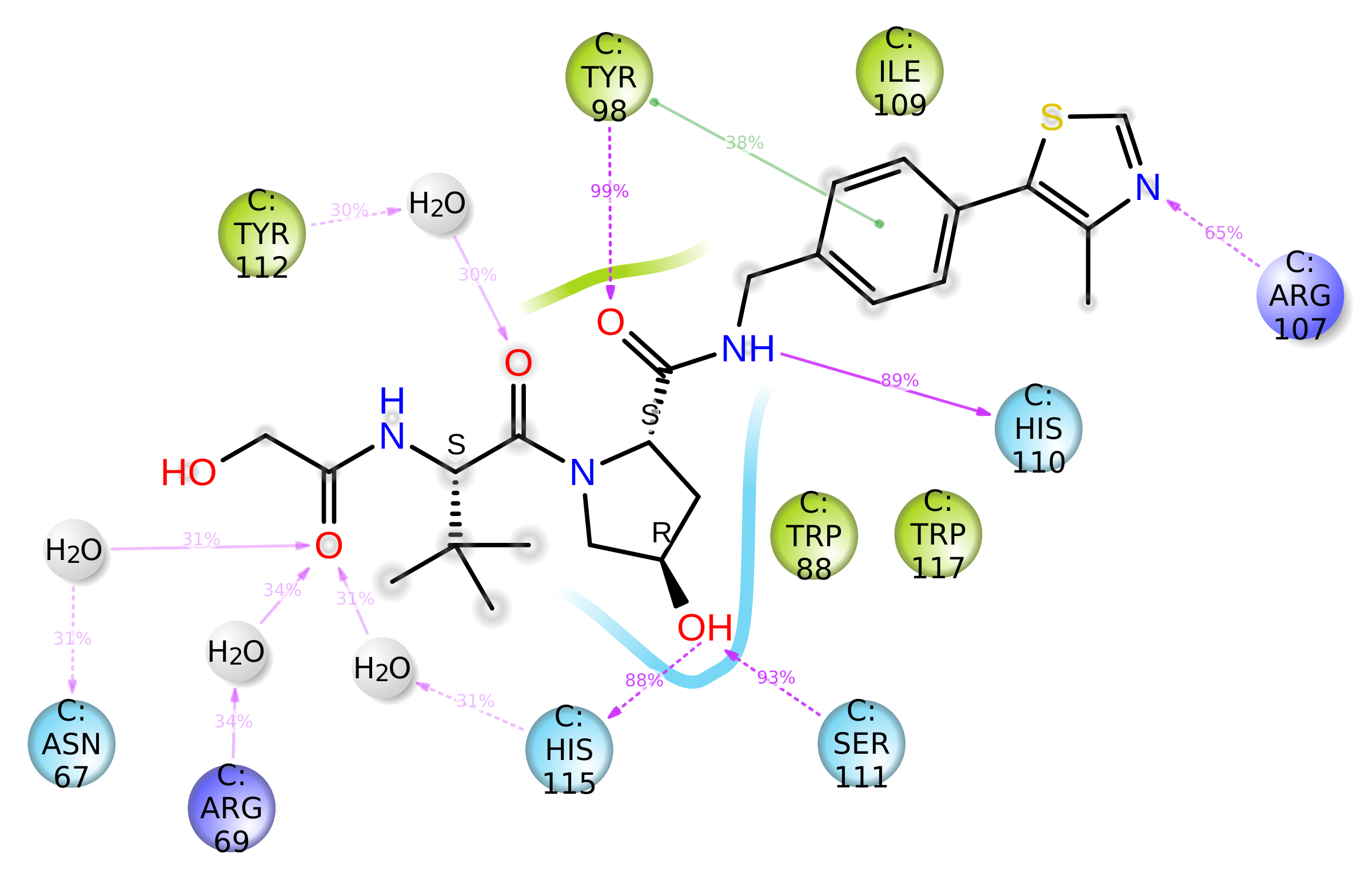 |

**Figure S7:** Analysis of 500 ns MD simulation of the crystal VHL structure (PDB ID: 5NVV), excluding the flexible loop of residues 155-202. (A) RMSD values of the protein Cα (orange) and ligand 3 fitting on protein Cα (green). (B) Radius of gyration the VHL-ligand 3 complex (C) Schematic representation of detailed ligand 3 atom interactions with protein residues.

(A)

(C)

(B)

(A)

(B)

(C)

| 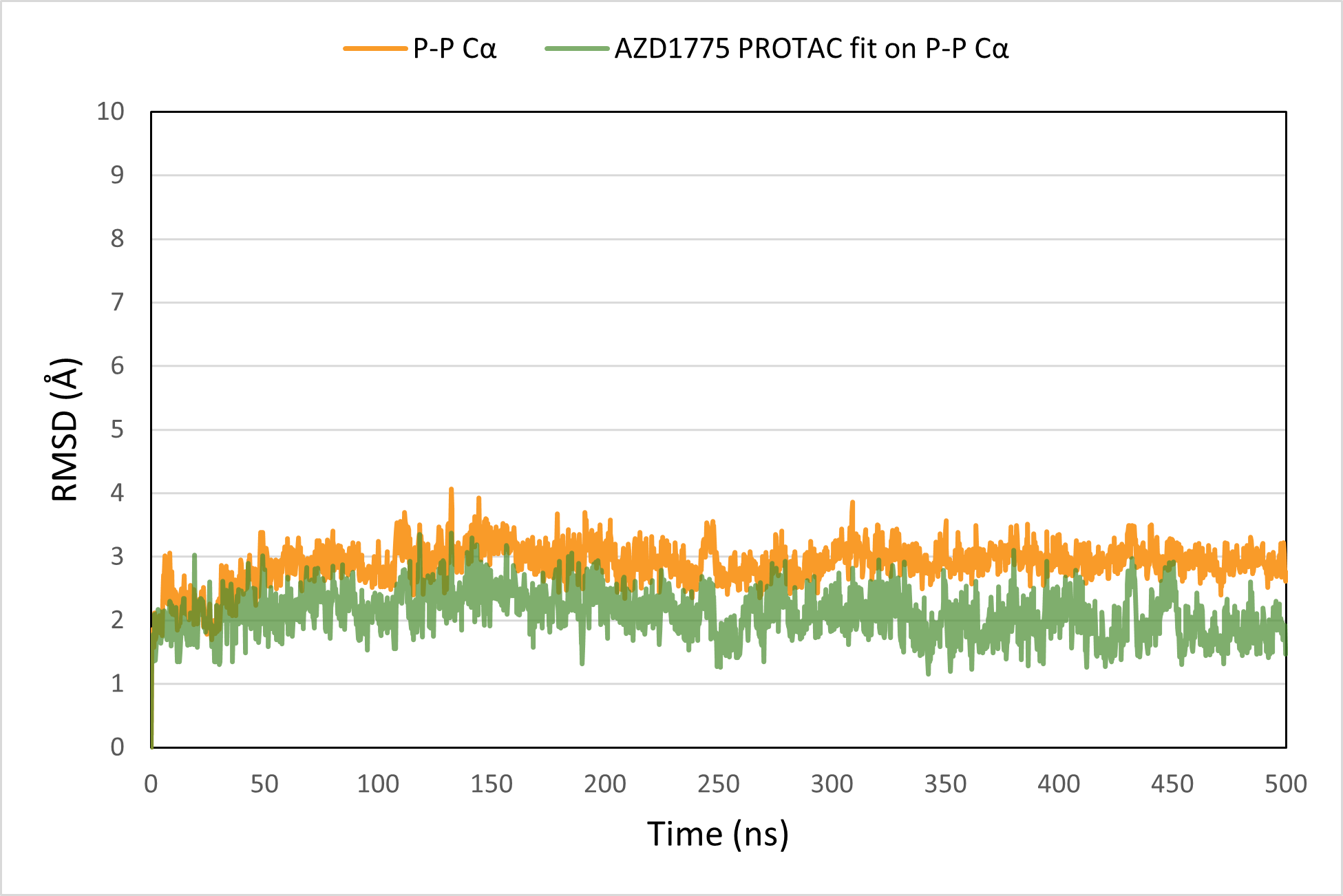 |
| --- |
| 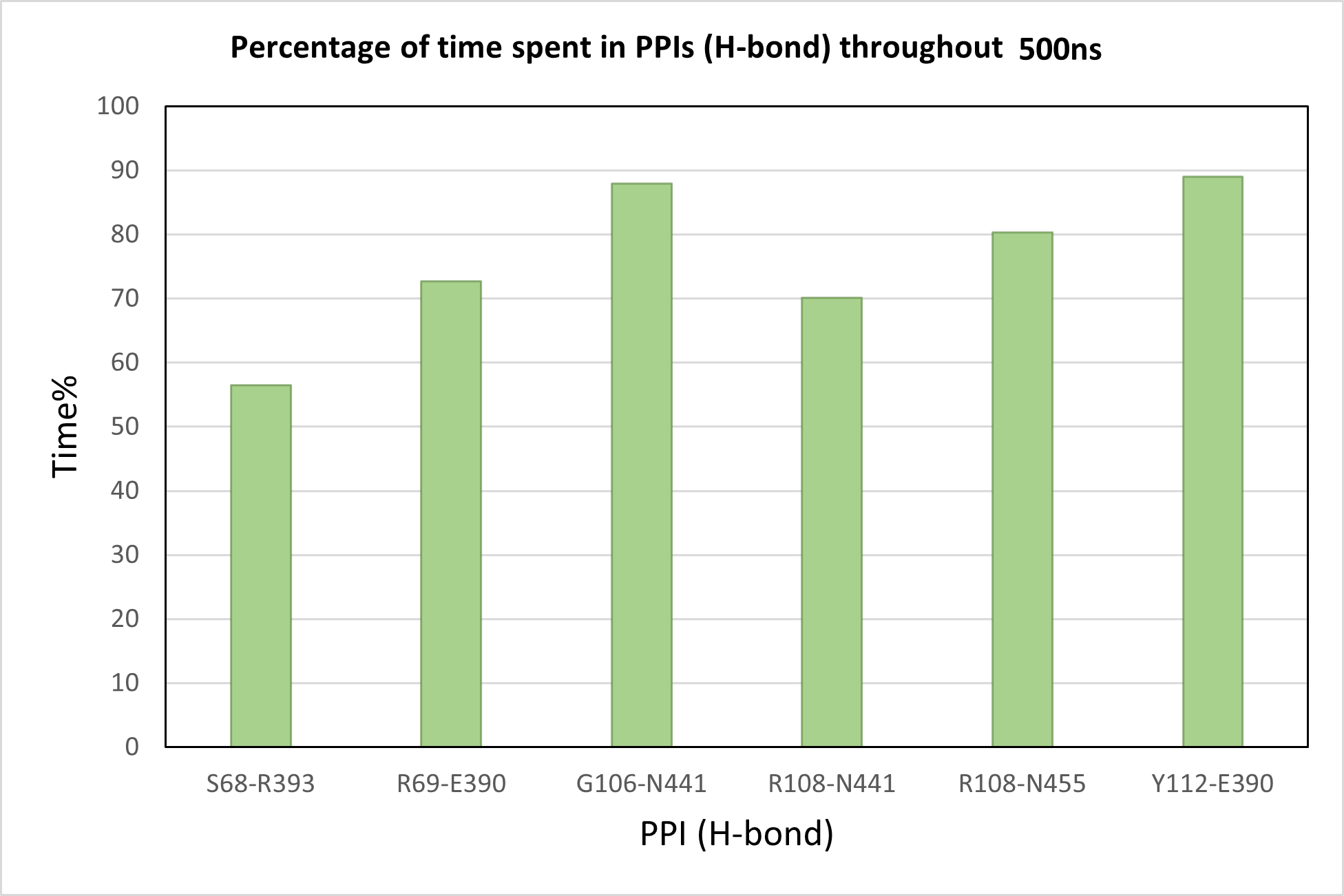 |
| 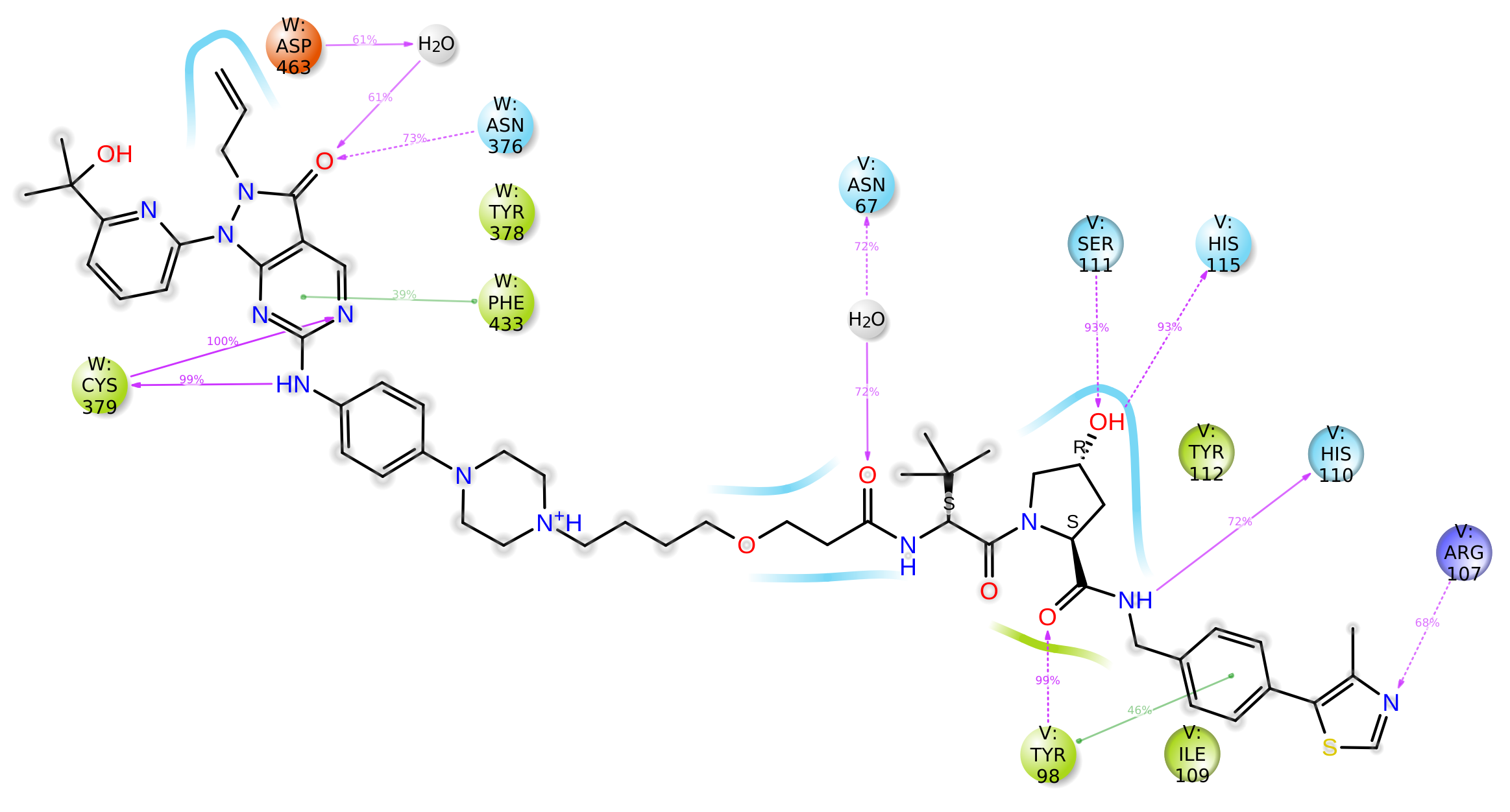 |

**Figure S8:** Analysis of the 500 ns MD simulation of the WEE1-AZD1775 PROTAC-VHL ternary complex (PDB ID 8WDK). (A) RMSD values of the protein Cα (orange) and AZD1775 PROTAC fitting on the protein Cα (green). (B) The percentage of time spent in PPIs (H-bonds) over the entire 500 ns. (C) Schematic representation of detailed AZD1775 PROTAC atom interactions with protein residues.

(B)

(A)

| 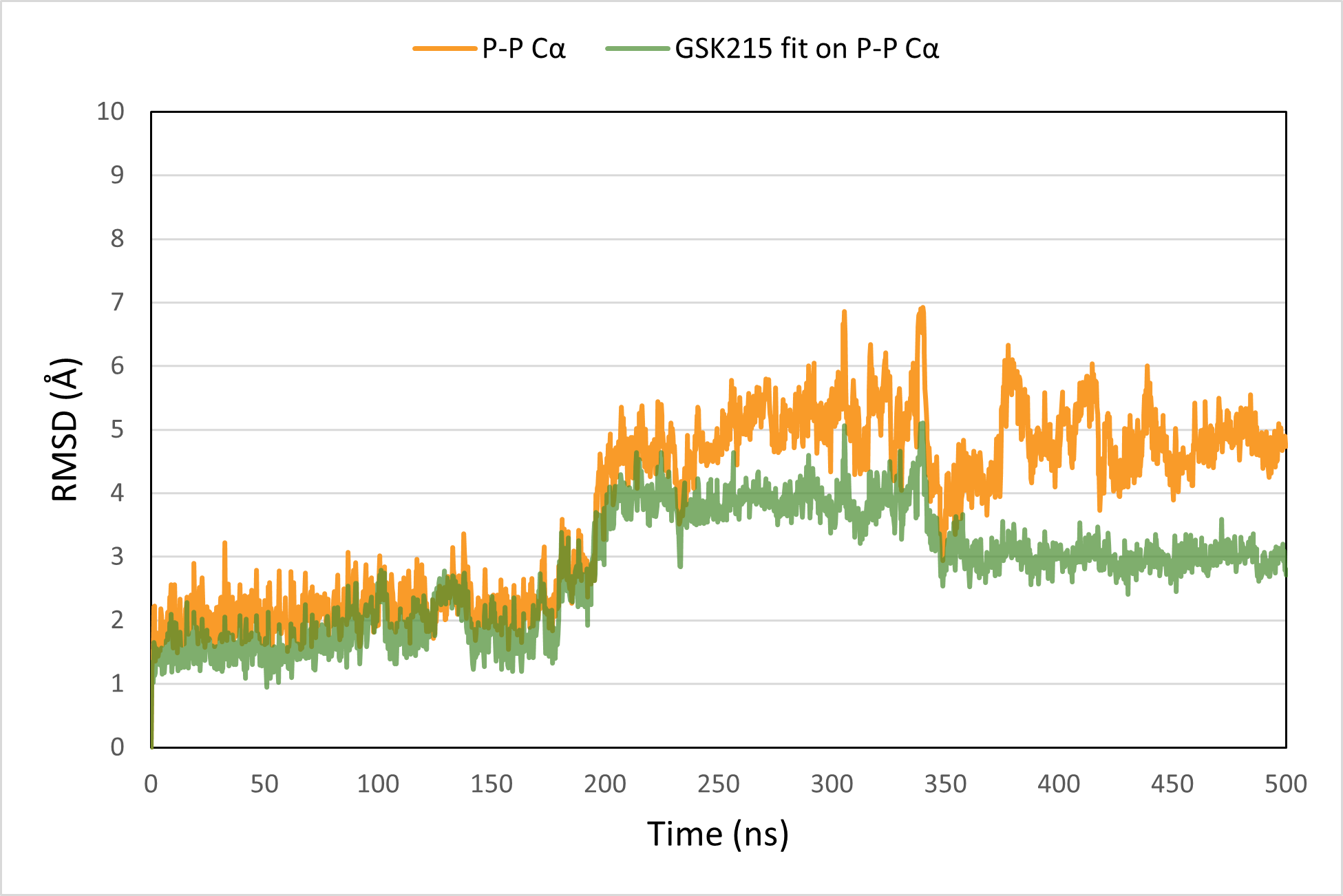 |
| --- |
| 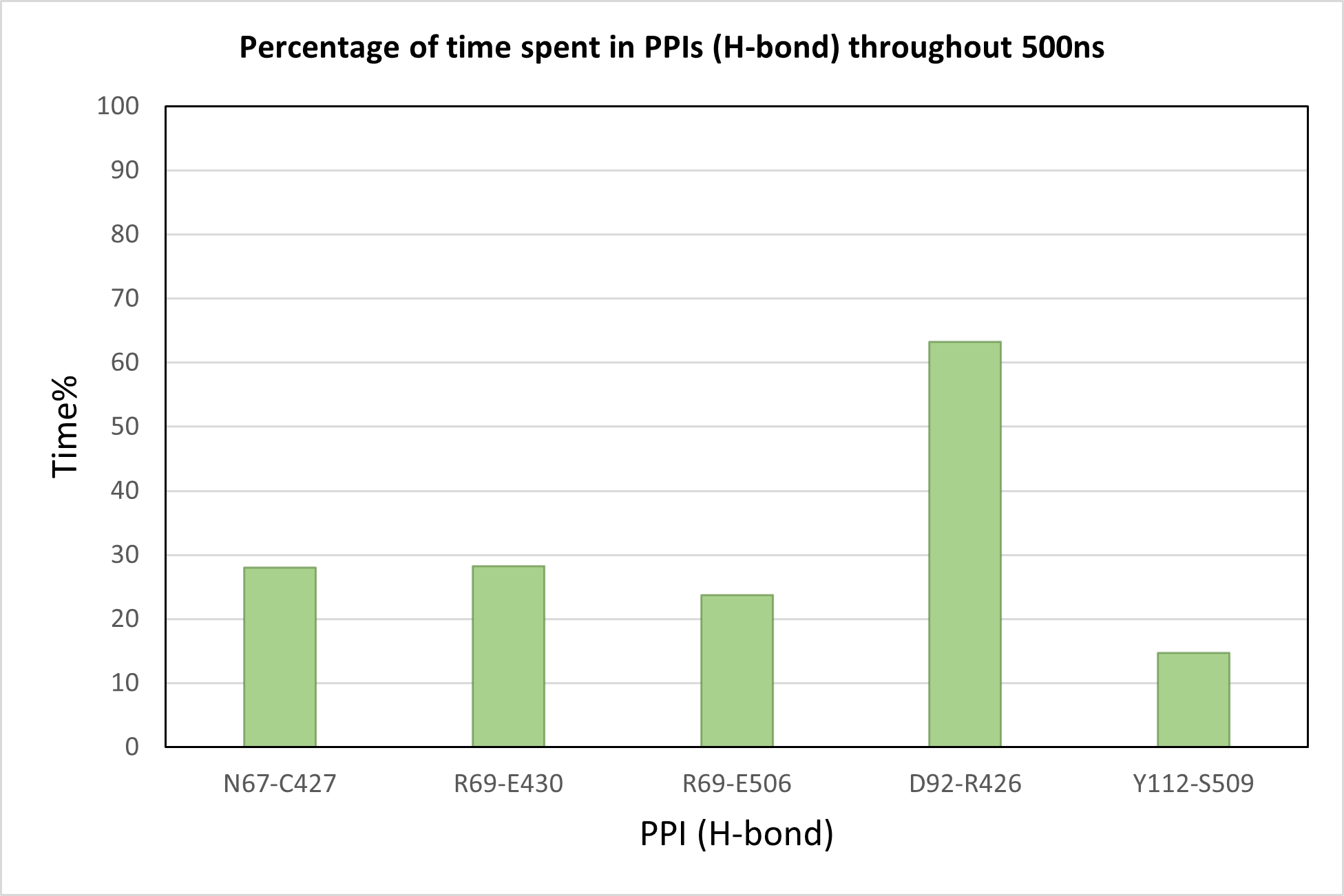 |
| 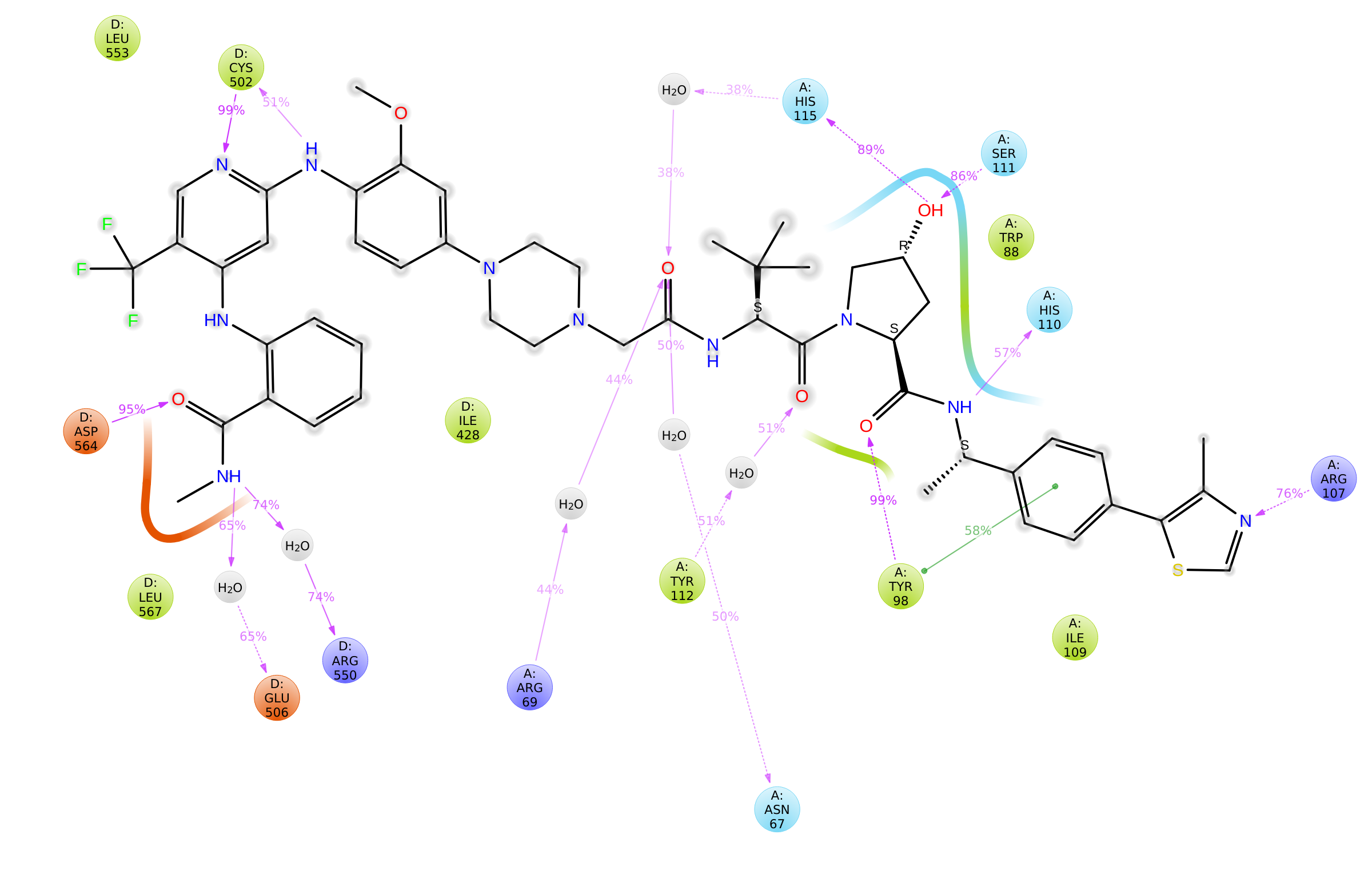 |

**Figure S9:** Analysis of the 500 ns MD simulation of the crystal FAK-GSK215-VHL ternary complex (PDB ID: 7PI4). (A) RMSD values of the protein Cα (orange) and GSK215 fitting on the protein Cα (green). (B) The percentage of time spent in PPIs (H-bonds) over the entire 500 ns. (C) Schematic representation of detailed GSK215 atom interactions with protein residues.

(C)

(C)

(B)

(A)

| 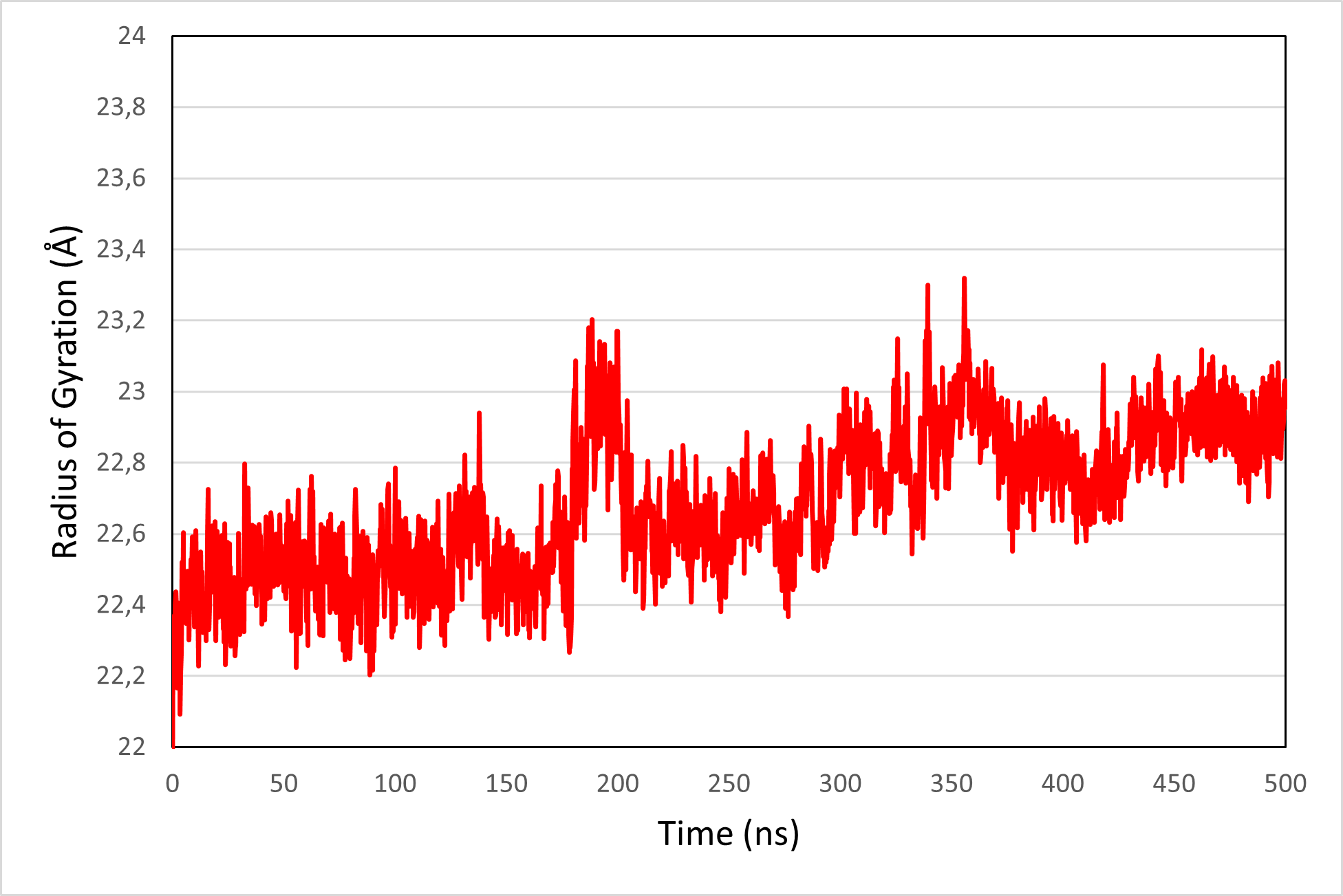 |
| --- |
| 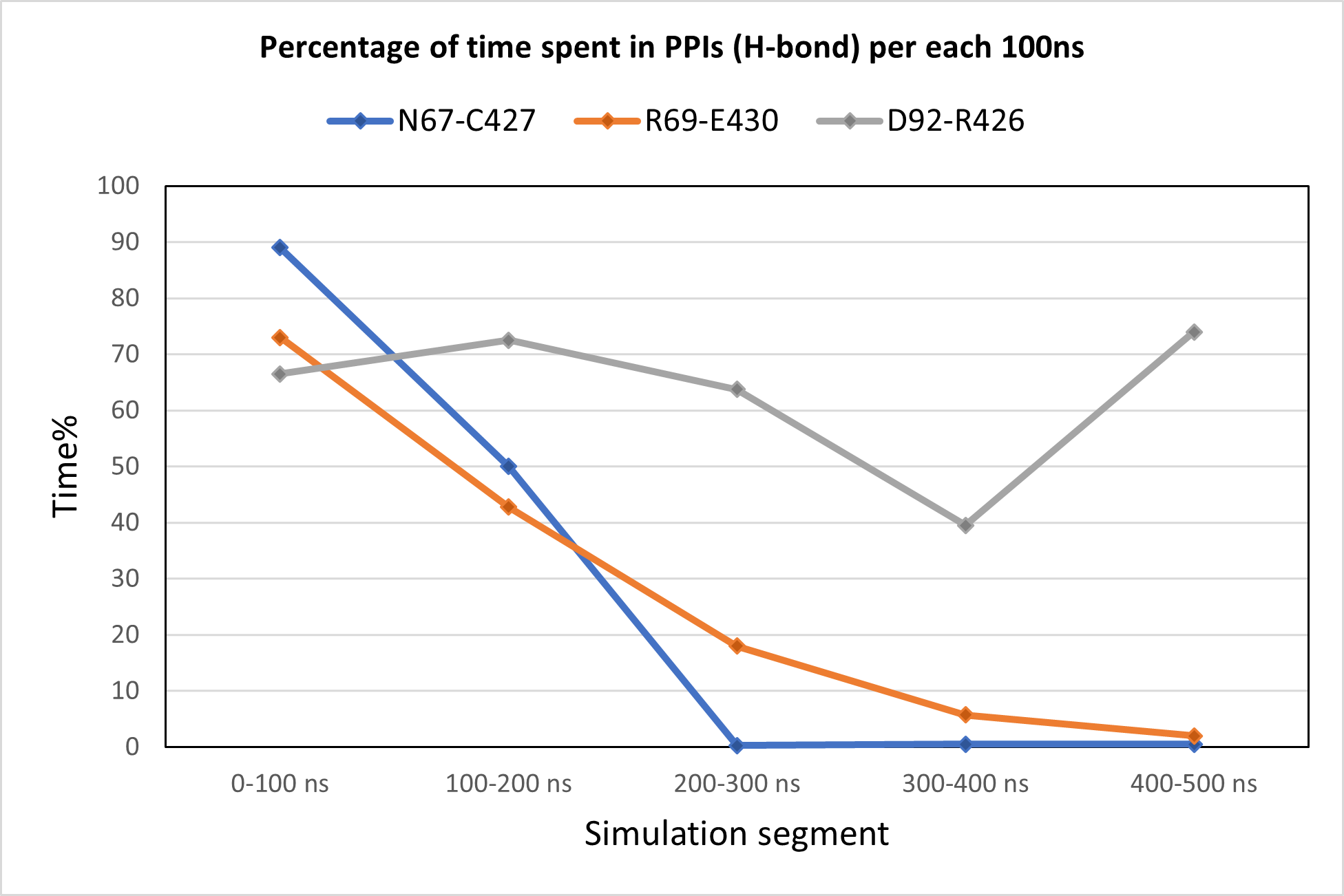 |
| 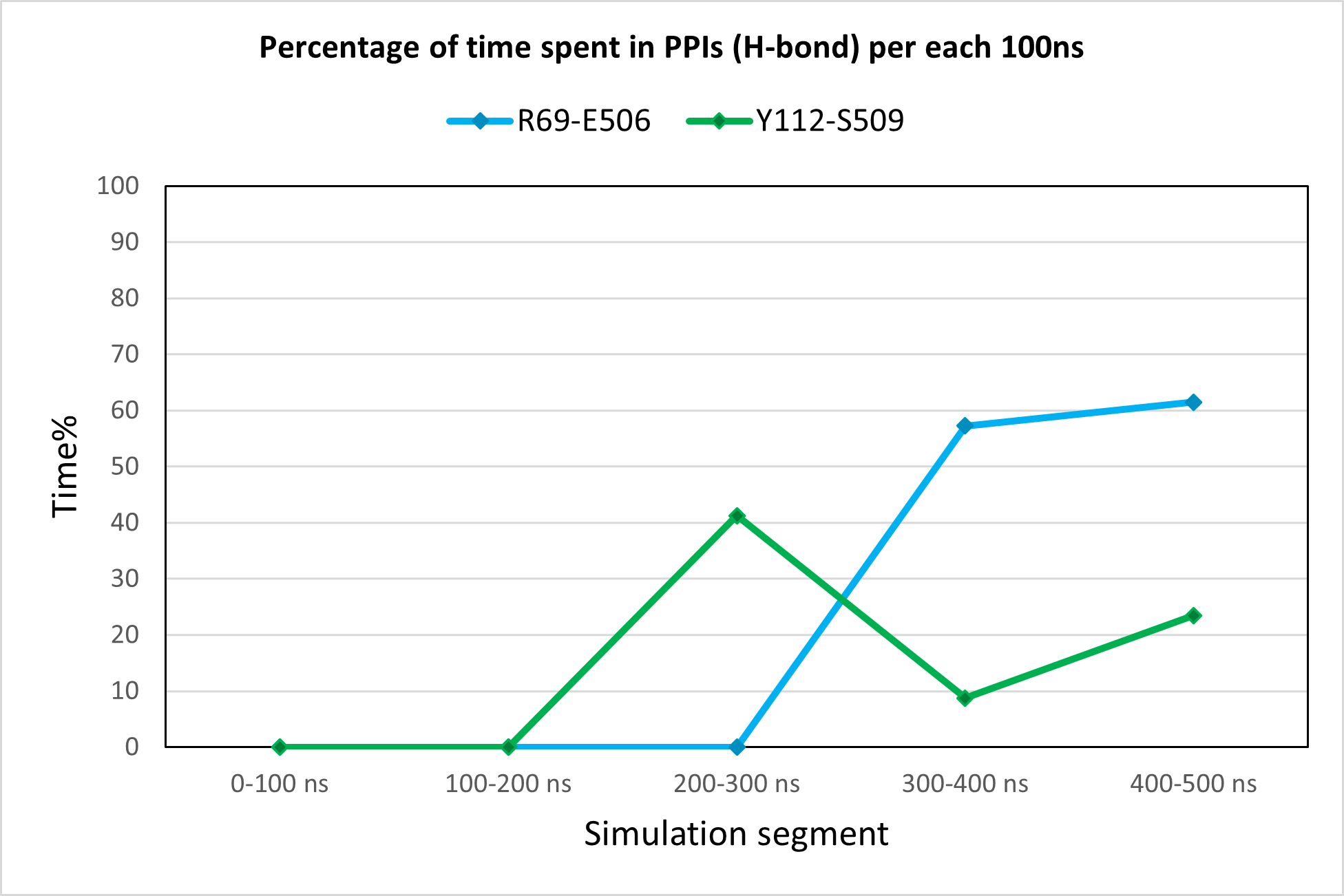 |

**Figure S10:** Analysis of the 500 ns MD simulation of the crystal FAK-GSK215-VHL ternary complex (PDB ID: 7PI4). (A) Radius of gyration of the ternary complex. (B) and (C) show the percentage of time spent in PPIs (H-bonds) in each 100 ns interval.

| 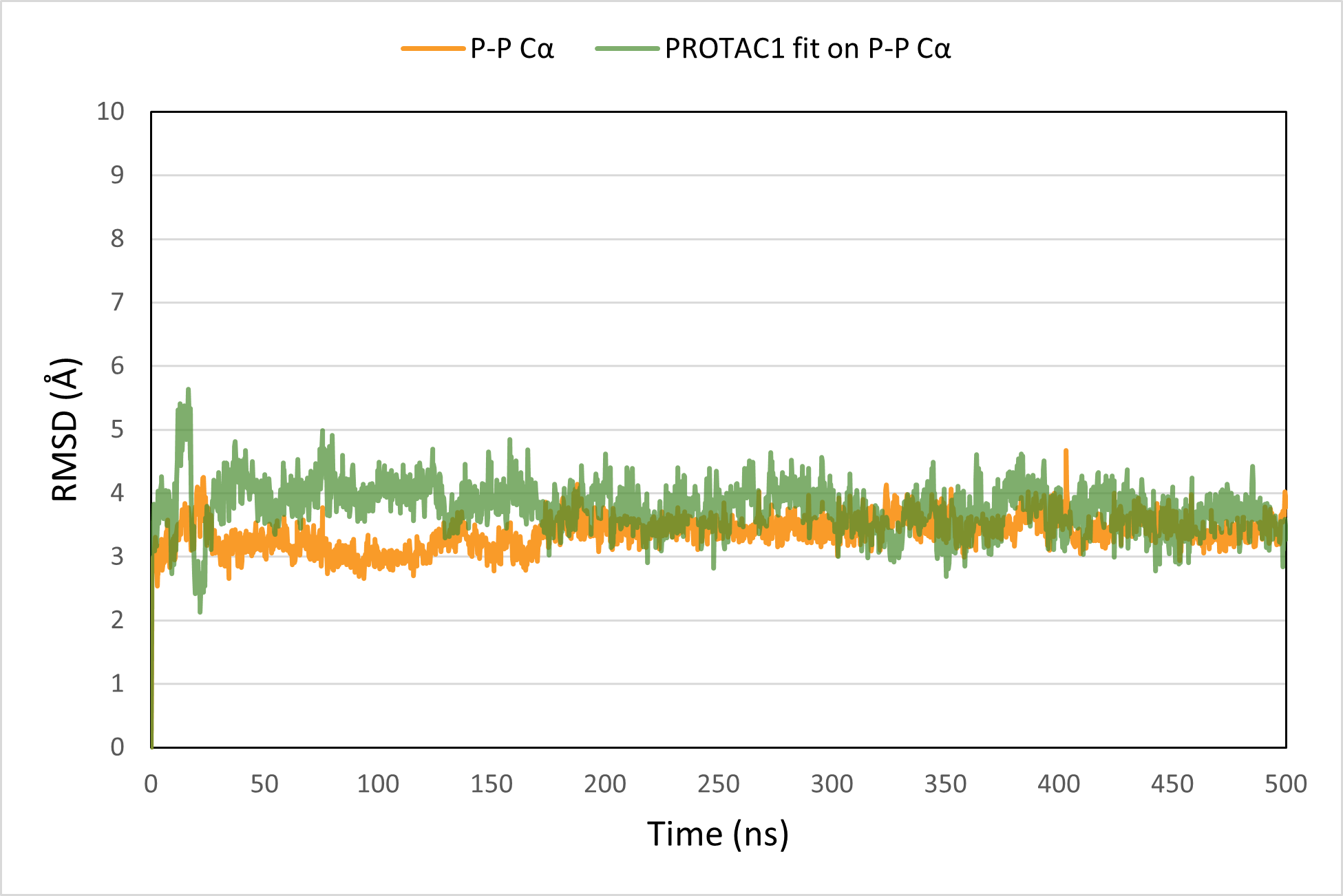 |
| --- |
| 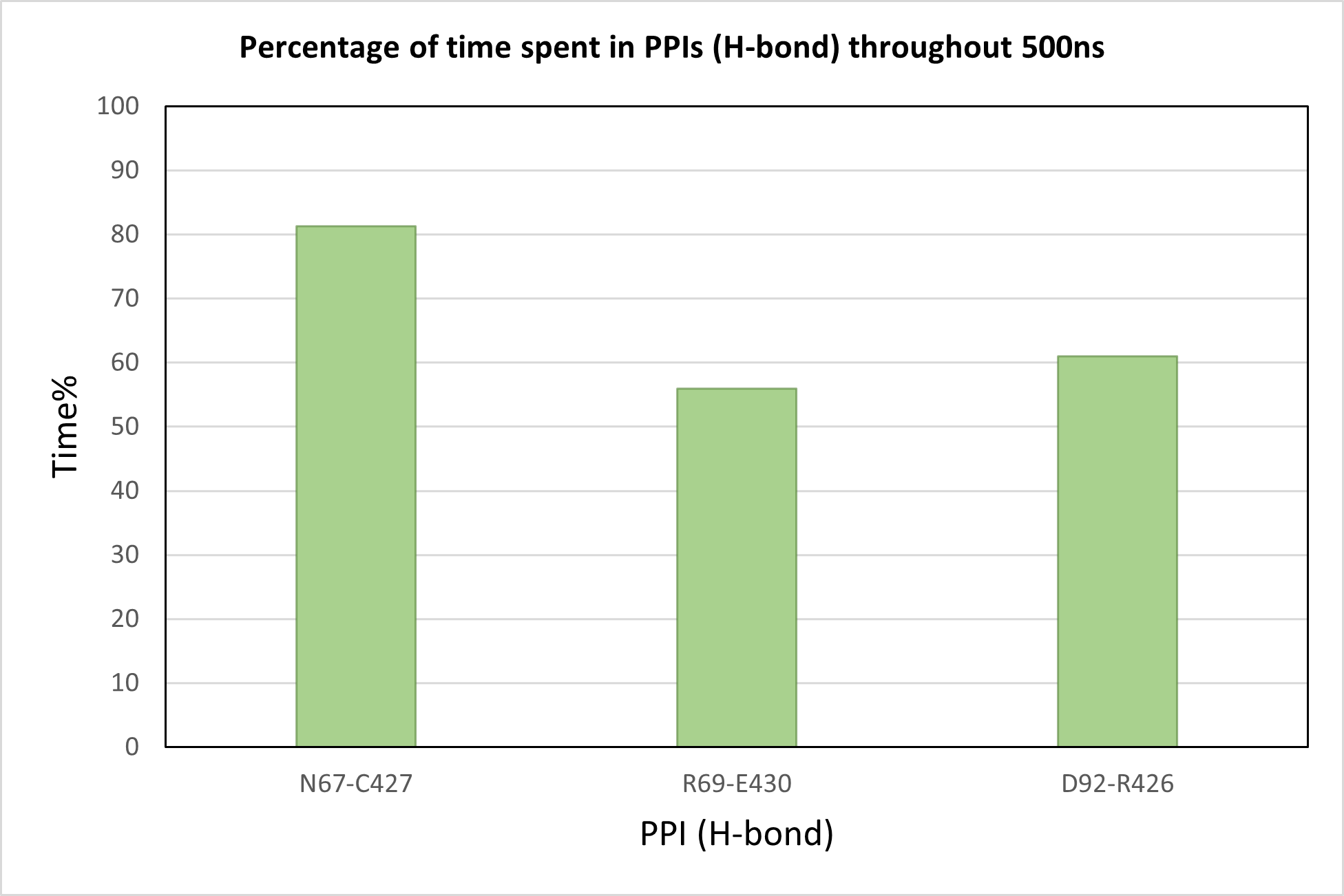 |
| 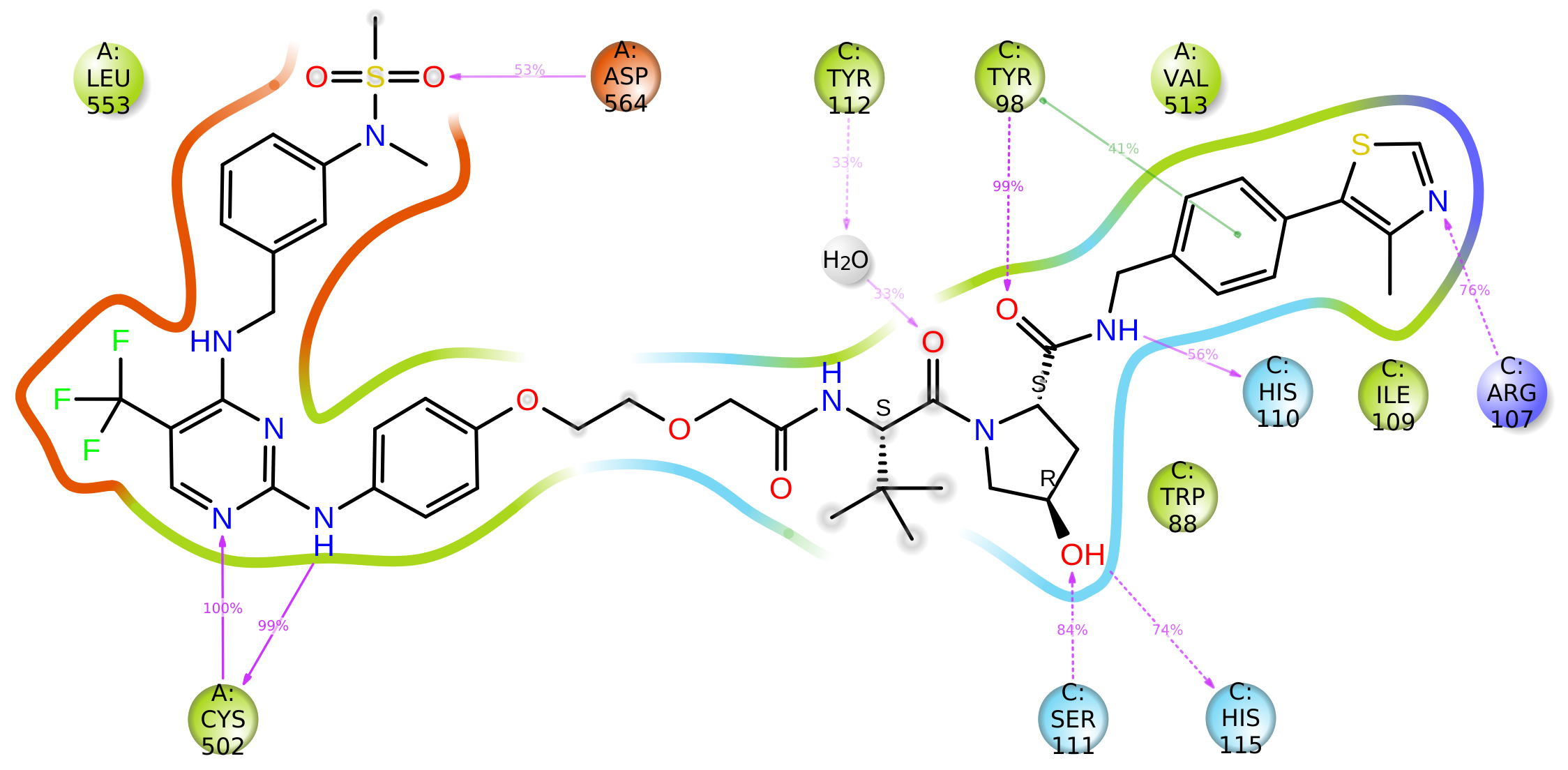 |

**Figure S11:** Analysis of the 500 ns MD simulation of the modeled FAK-PROTAC1-VHL ternary complex obtained via induced fit docking. (A) RMSD values of the protein Cα (orange) and PROTAC1 fitting on the protein Cα (green). (B) The percentage of time spent in PPIs (H-bonds) over the entire 500 ns. (C) Schematic representation of detailed PROTAC1 atom interactions with protein residues.

(C)

(A)

(B)

| 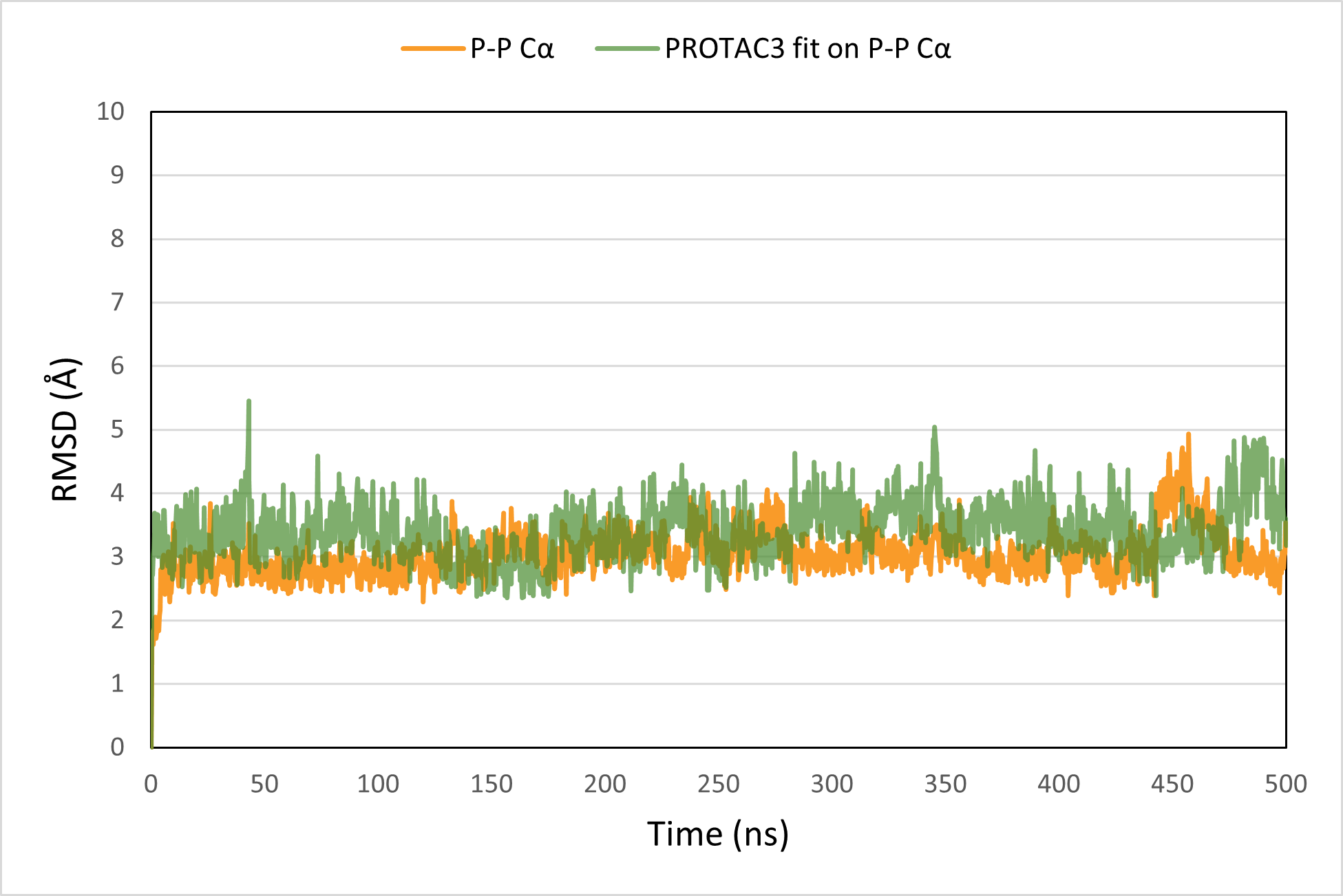 |
| --- |
| 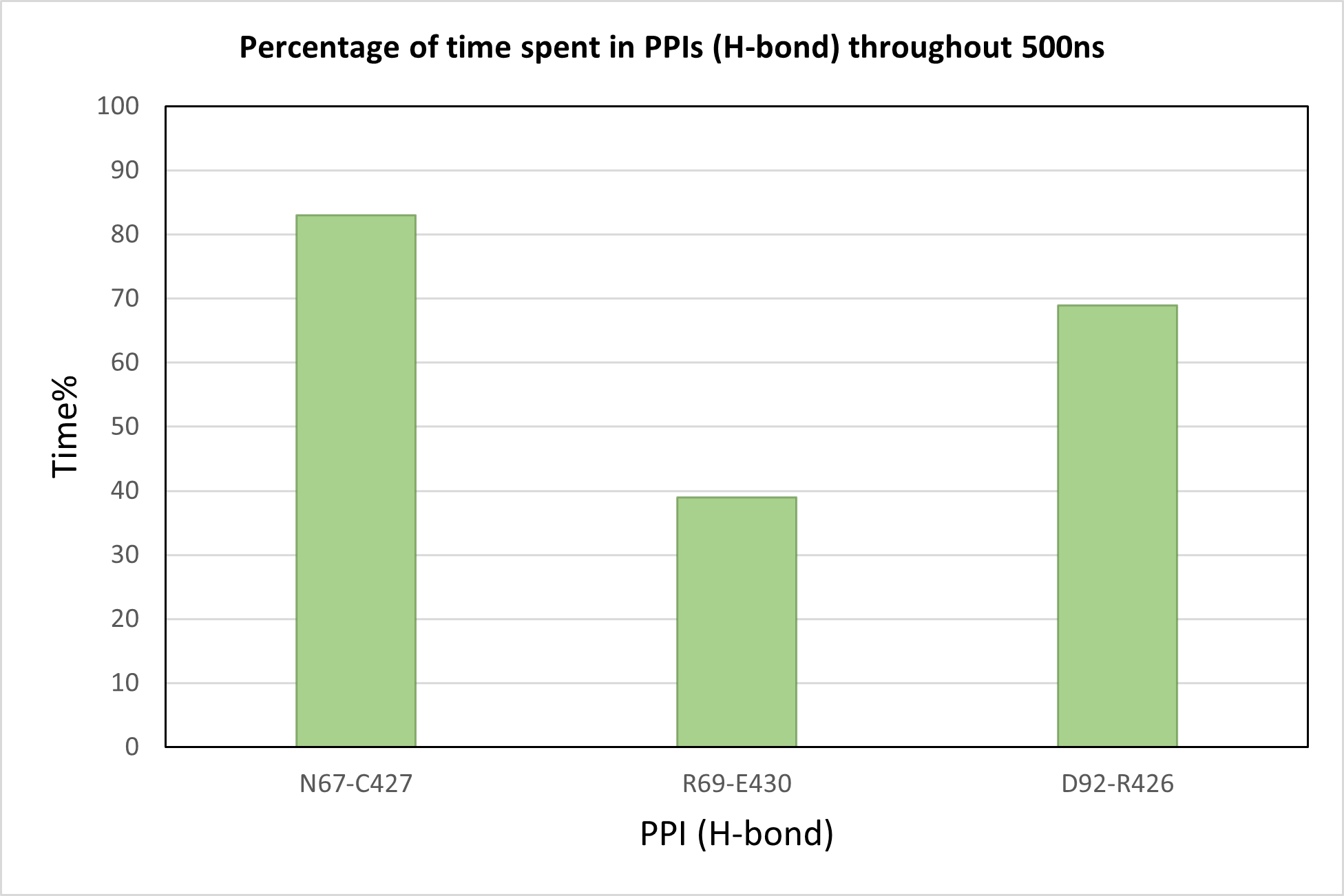 |
| 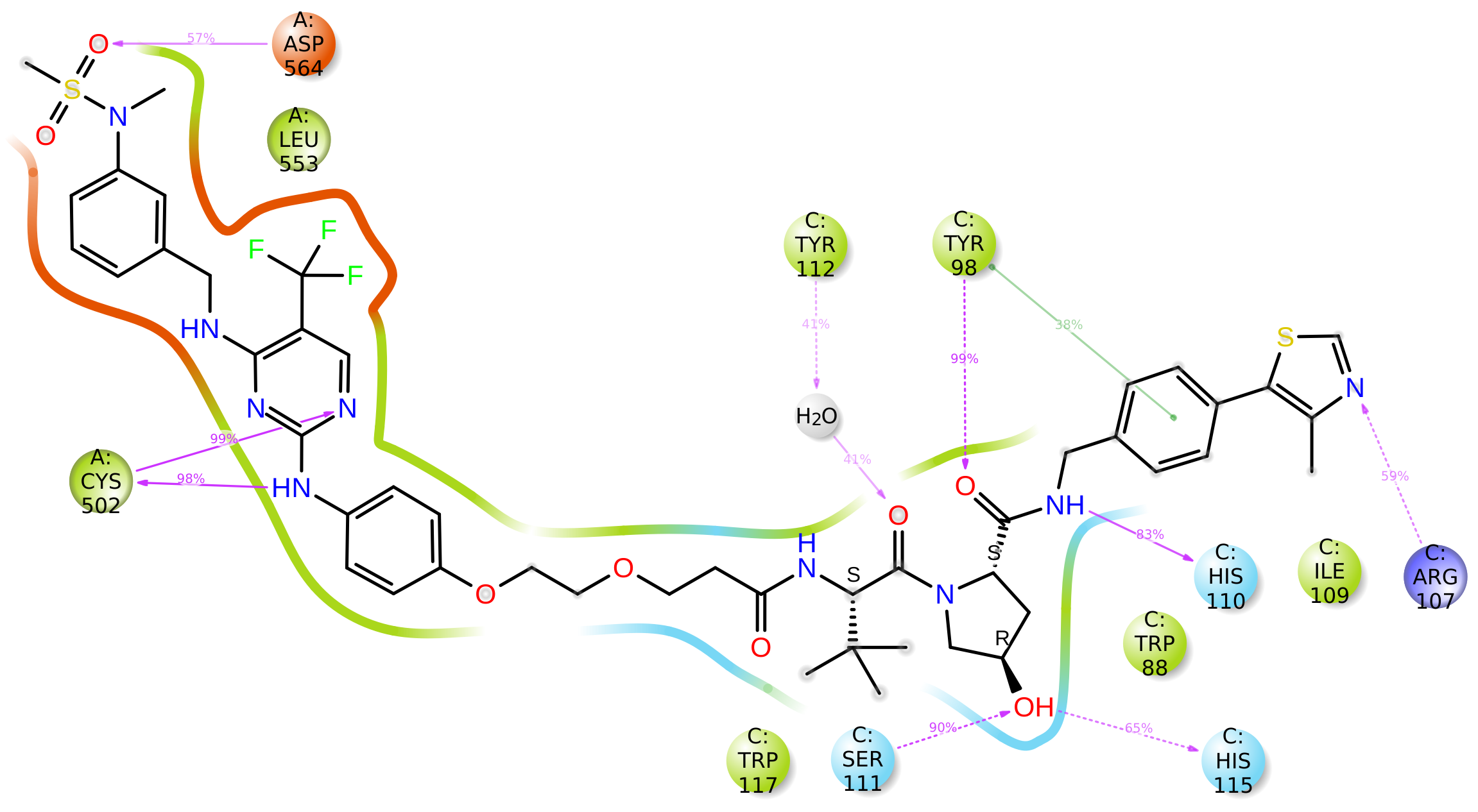 |

**Figure S12:** Analysis of the 500 ns MD simulation of the modeled FAK-PROTAC3-VHL ternary complex obtained via induced fit docking. (A) RMSD values of the protein Cα (orange) and PROTAC3 fitting on the protein Cα (green). (B) The percentage of time spent in PPIs (H-bonds) over the entire 500 ns. (C) Schematic representation of detailed PROTAC3 atom interactions with protein residues.

(A)

(C)

(B)

(B)

(A)

| 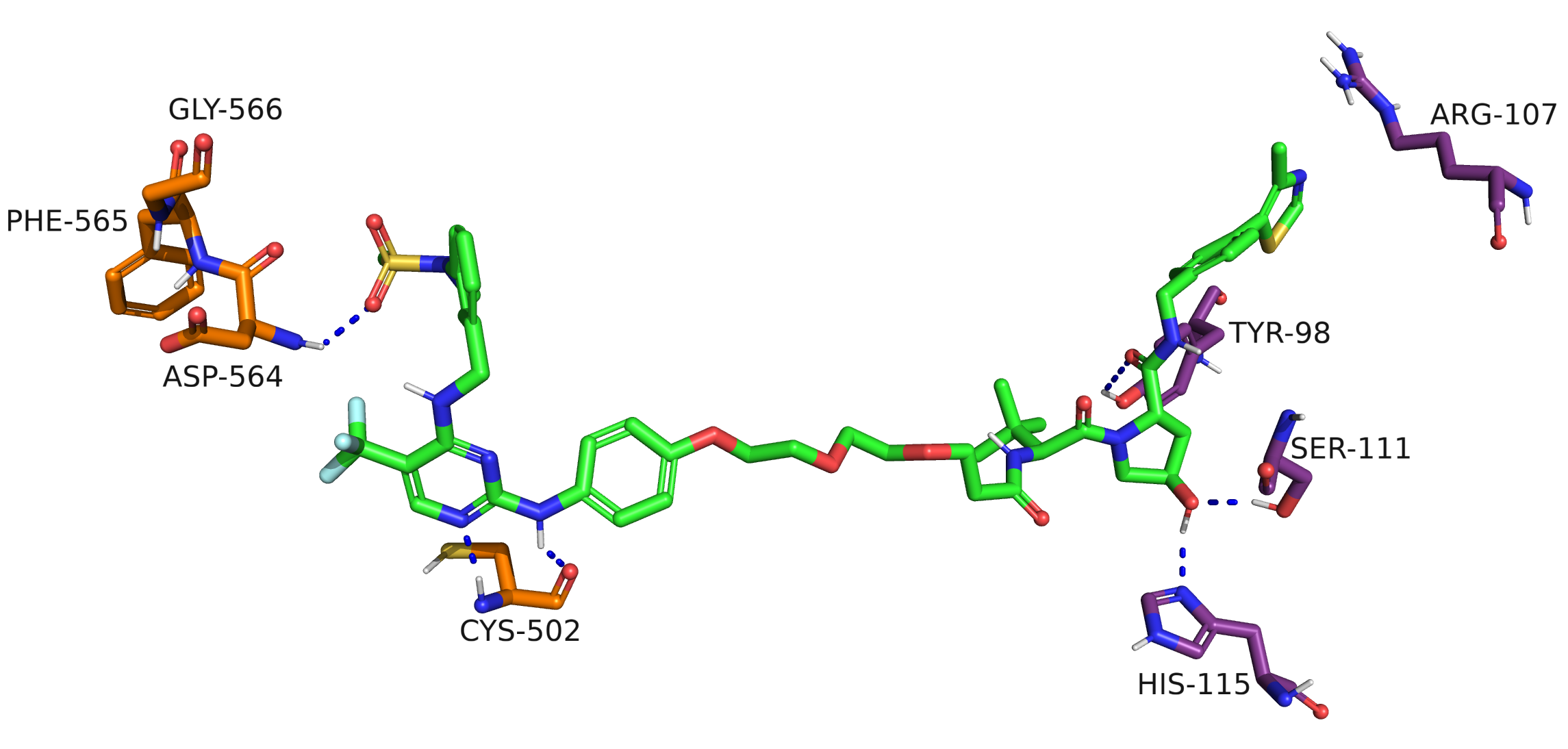 |
| --- |
| 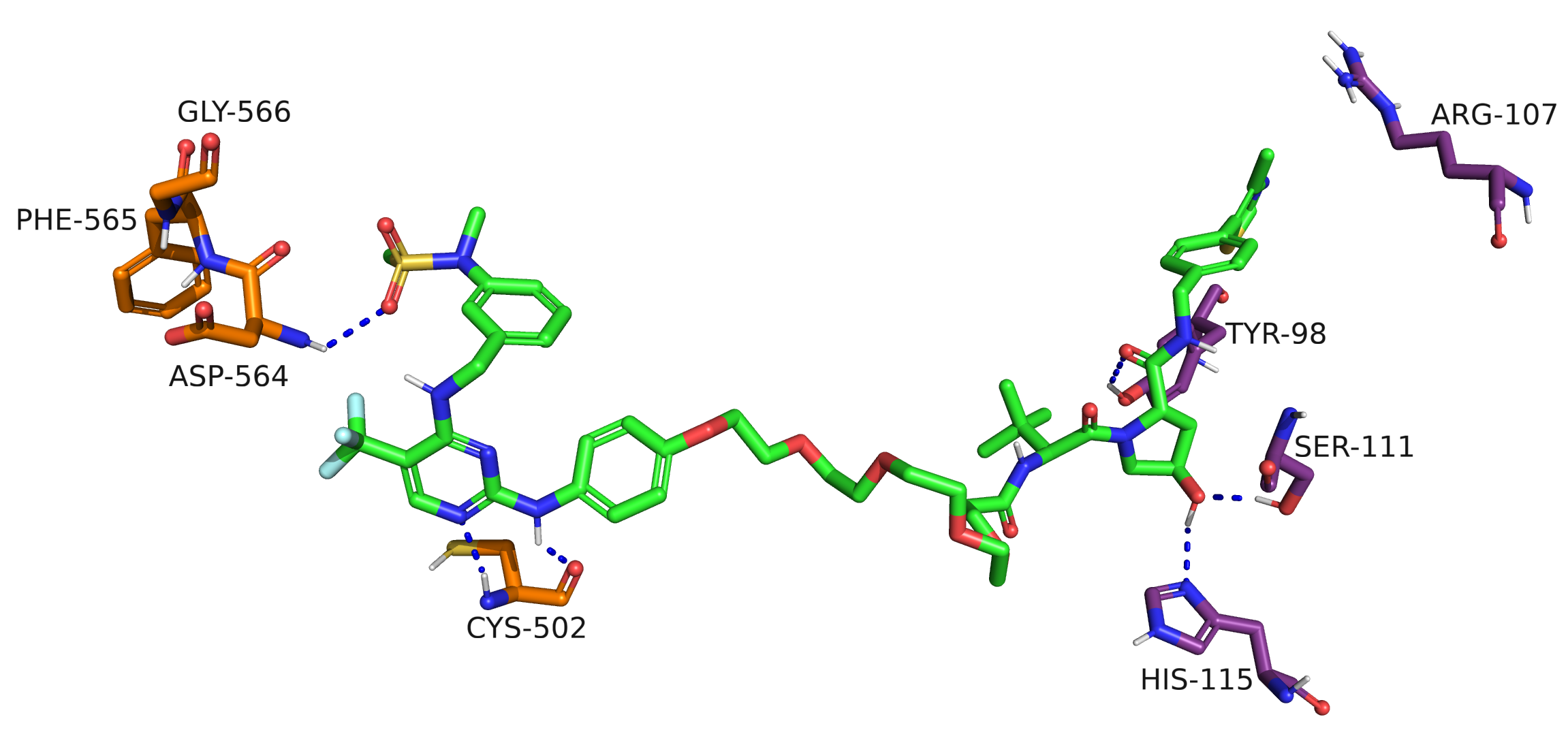 |

**Figure S13:** Detailed view of docking results of (A) PROTAC5 and (B) PROTAC6 within the last frame of the MD trajectory of FAK-PROTAC6-VHL model. Hydrogen bonds (destance below 2.5 Å) are shown as blue dashed lines.

(D)

(C)

| 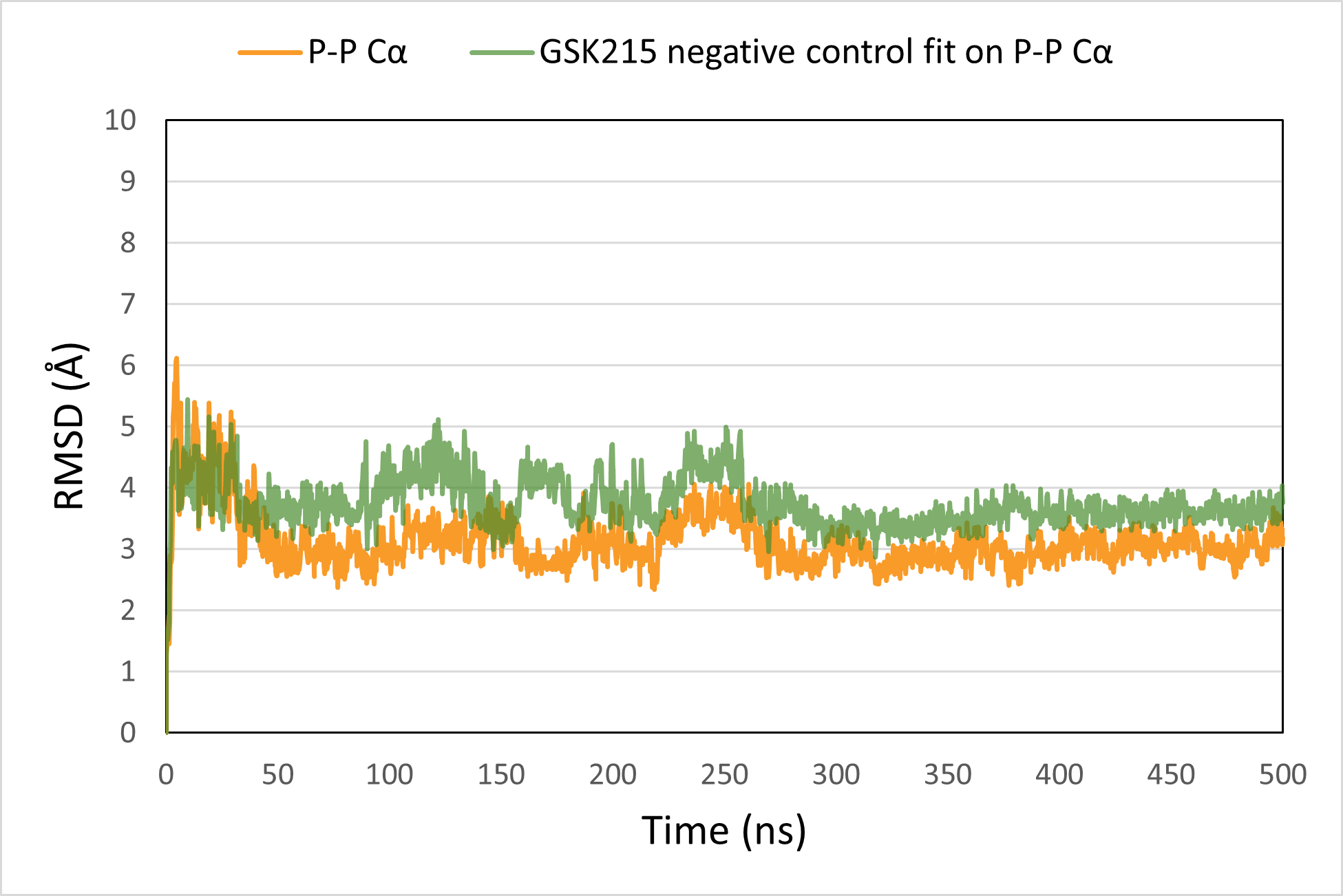 | 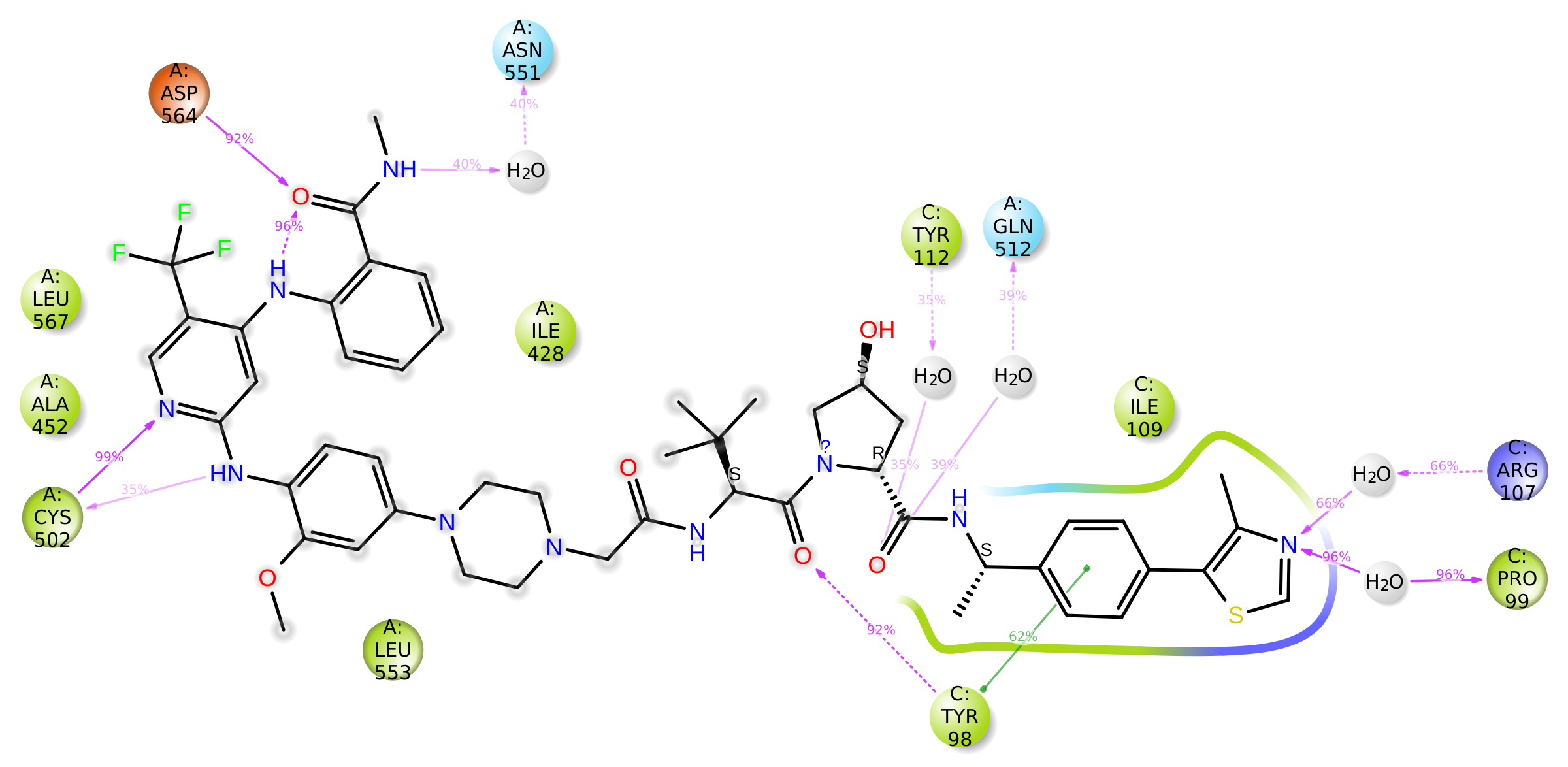 |
| --- | --- |
| 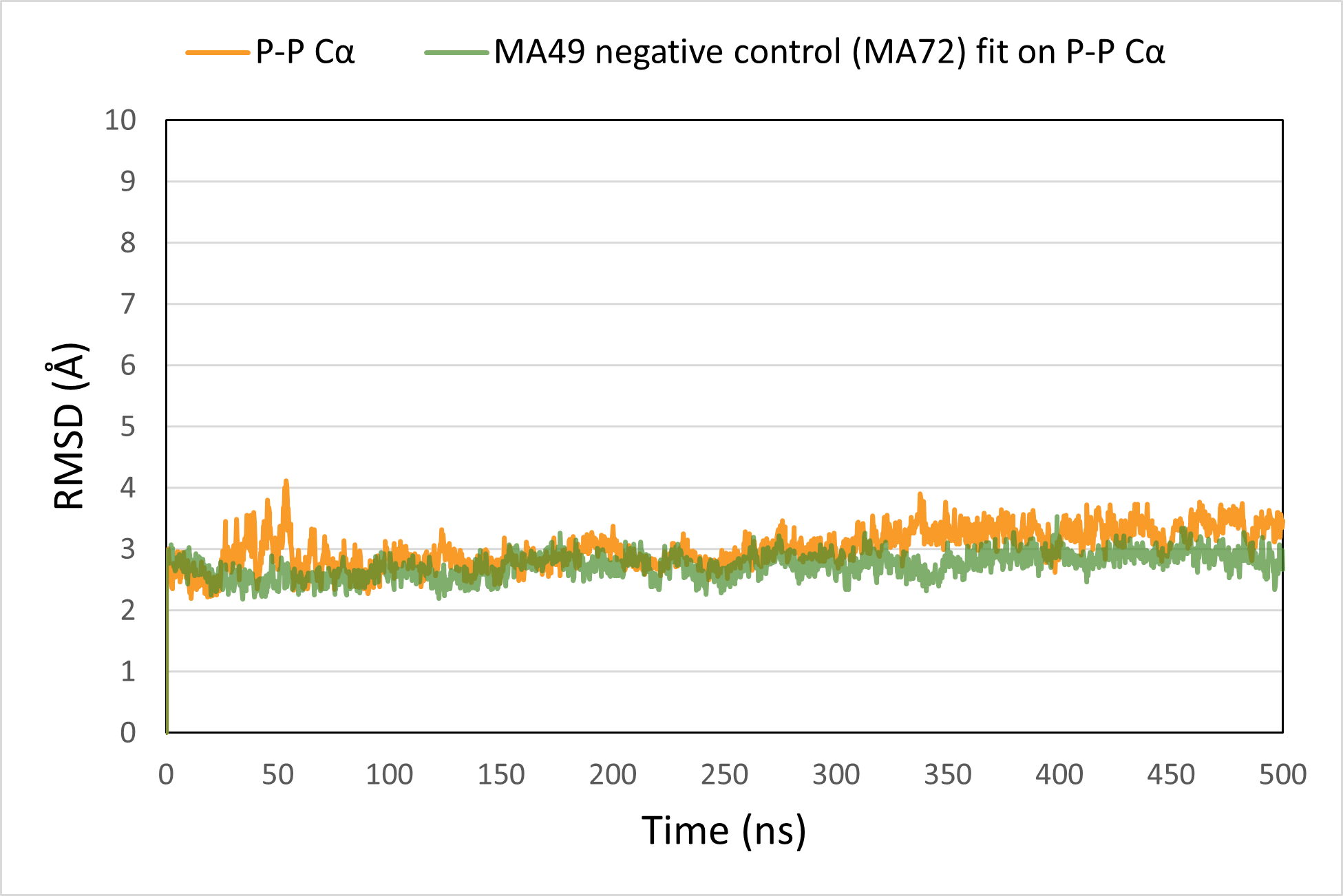 | 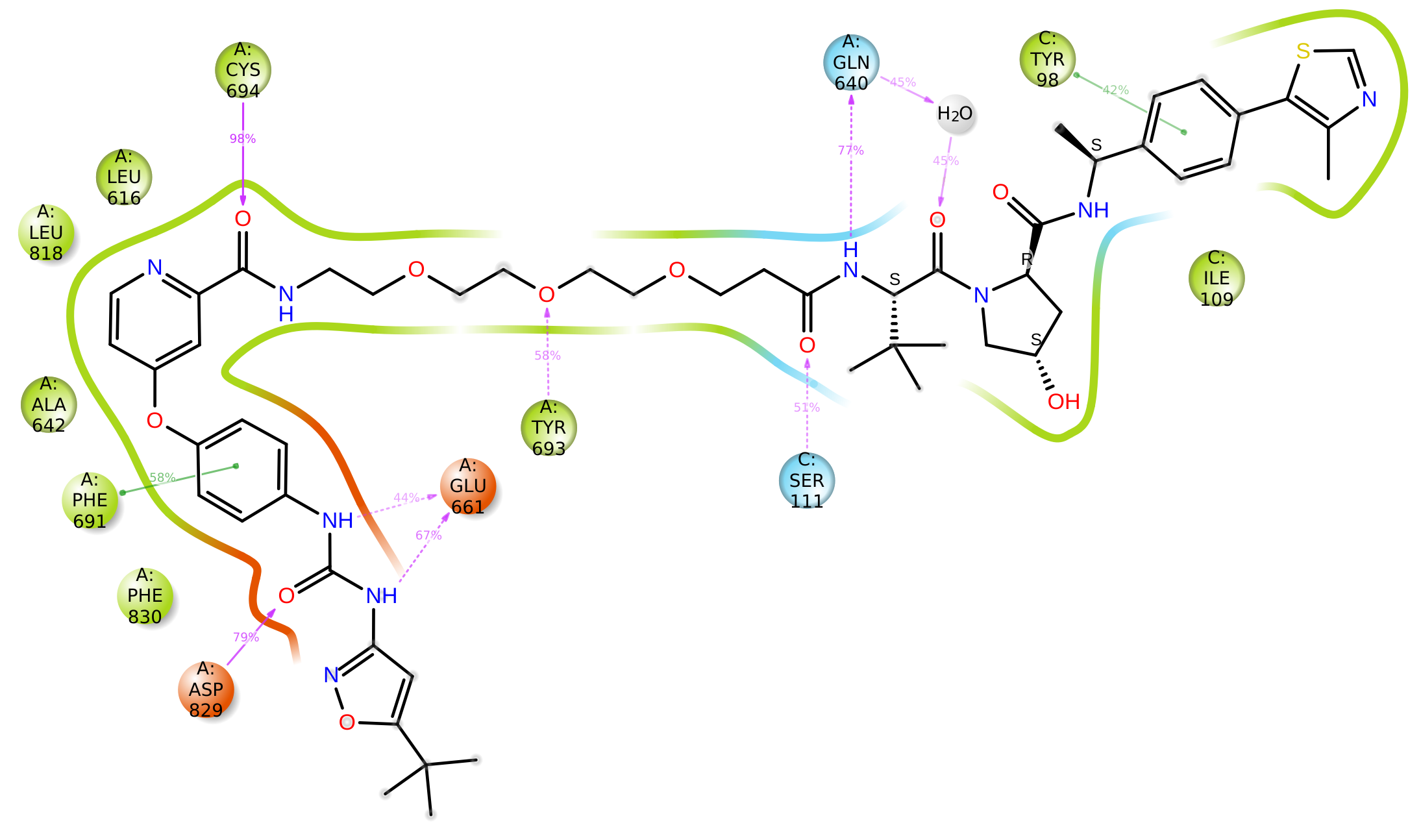 |

**Figure S14:** Analysis of the 500 ns MD simulation of the modeled negative controls of GSK215 and MA49 (MA72) ternary complexes obtained via induced fit docking. (A) RMSD values of the FAK-VHL protein Cα (orange) and -veGSK215 fitting on the protein Cα (green). (B) Schematic representation of detailed -veGSK215 atom interactions with FAK-VHL protein residues. (C) RMSD values of the FLT3-VHL protein Cα (orange) and MA72 fitting on the protein Cα (green). (D) Schematic representation of detailed MA72 atom interactions with FLT3-VHL protein residues.

(A)

(B)

(B)

(A)

| 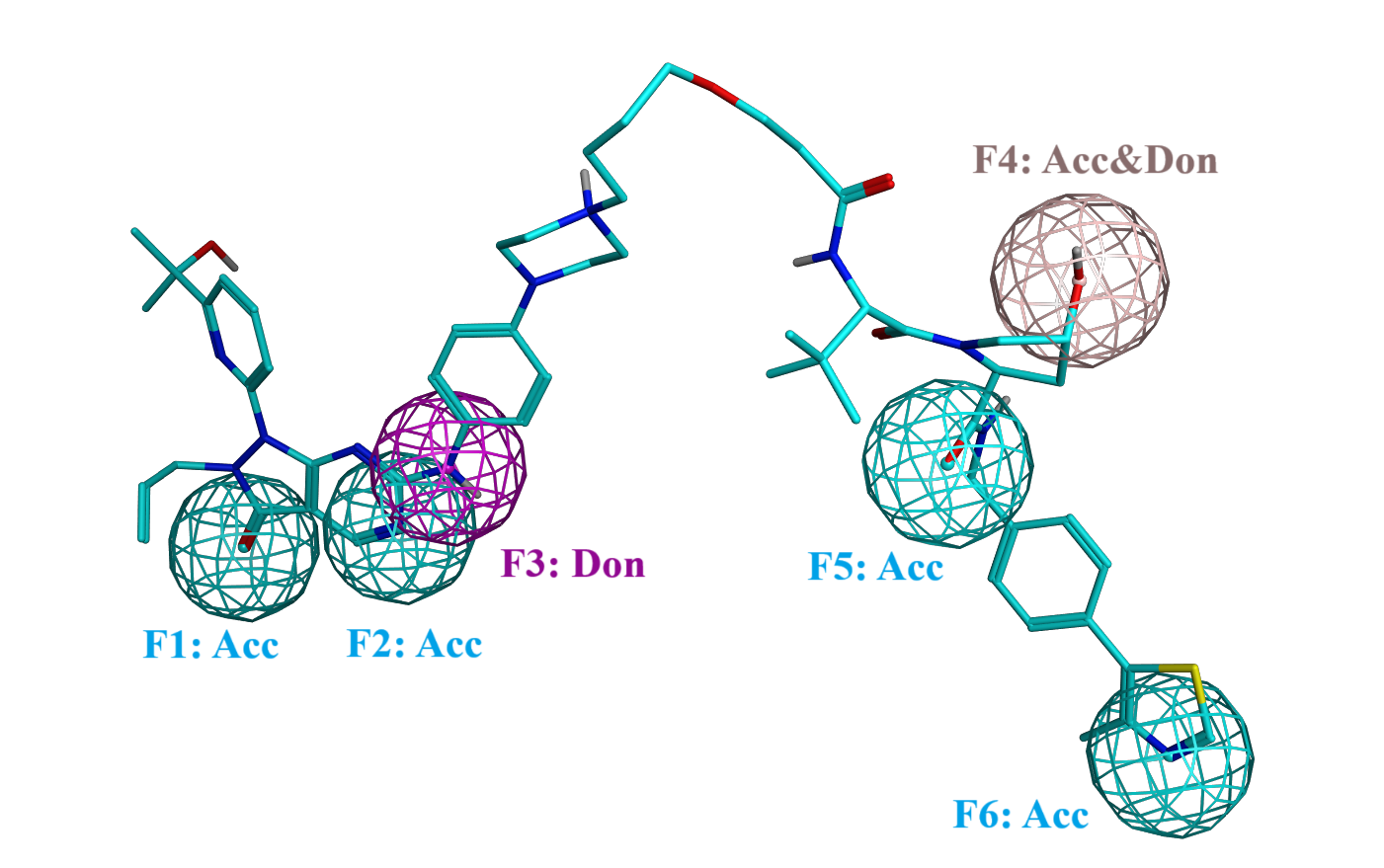 |
| --- |
| 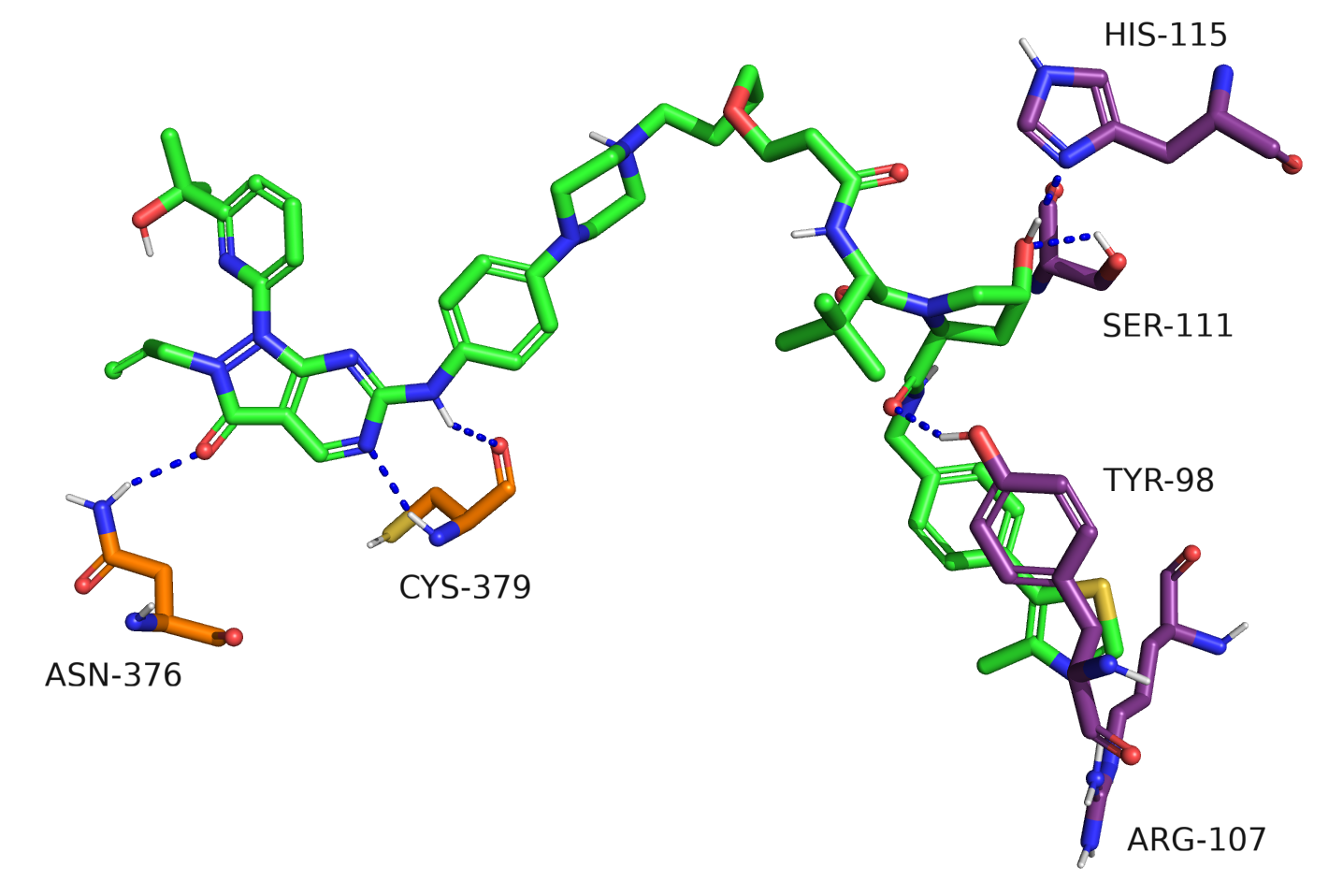 |

**Figure S15:** Detailed view of docking results within the experimental WEE1-AZD1775 PROTAC-VHL (8WDK) ternary structure. (A) Crystalized PROTAC pharmacophore features used to guide the placement of docking. (B) Interactions of PROTAC with the amino acids in the WEE1 and VHL binding pockets. Hydrogen bonds (distance below 2.5 Å) are shown as blue dashed lines.

- **Analysis of MD simulations second replicas (Figures S16 to S26):**

| 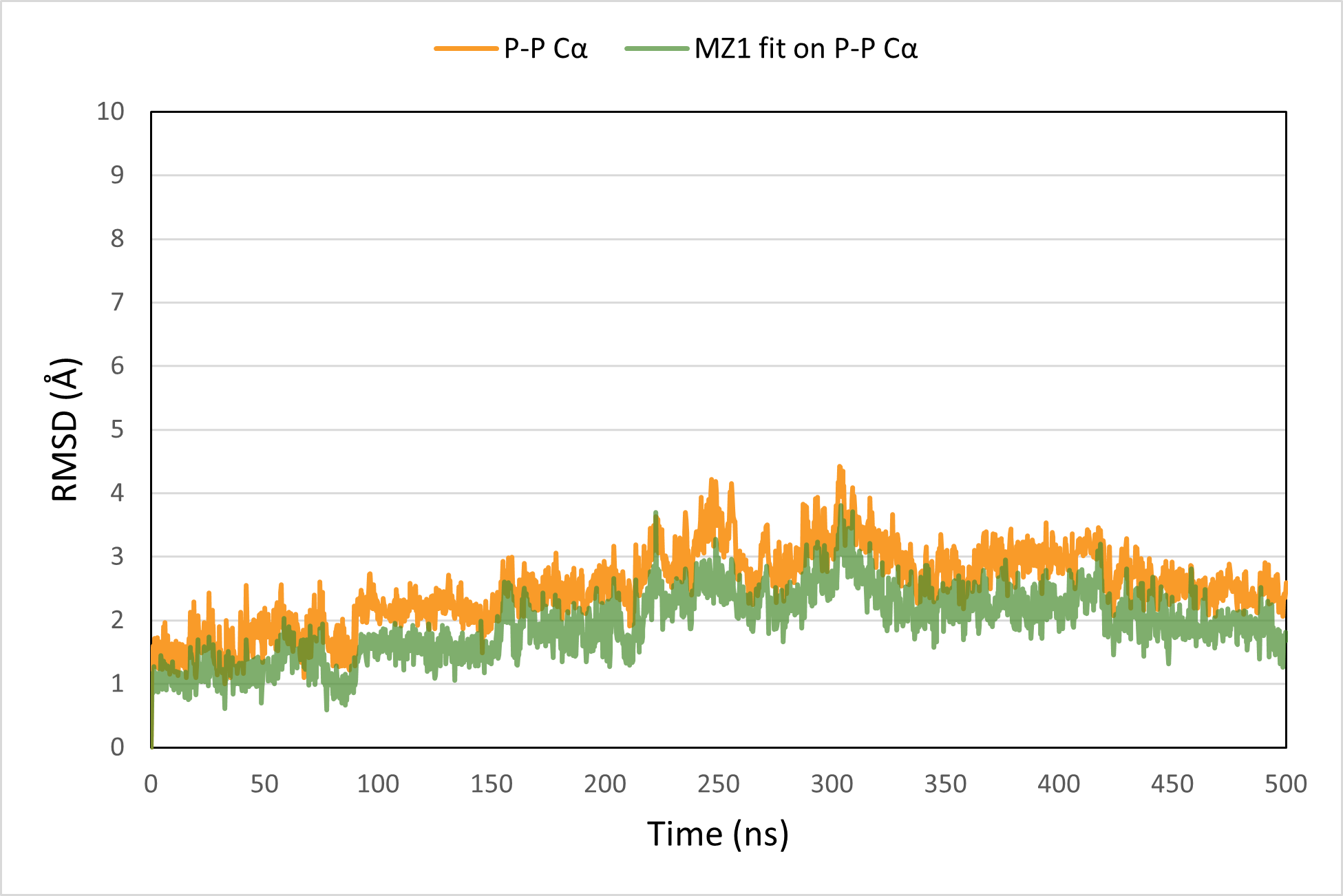 | 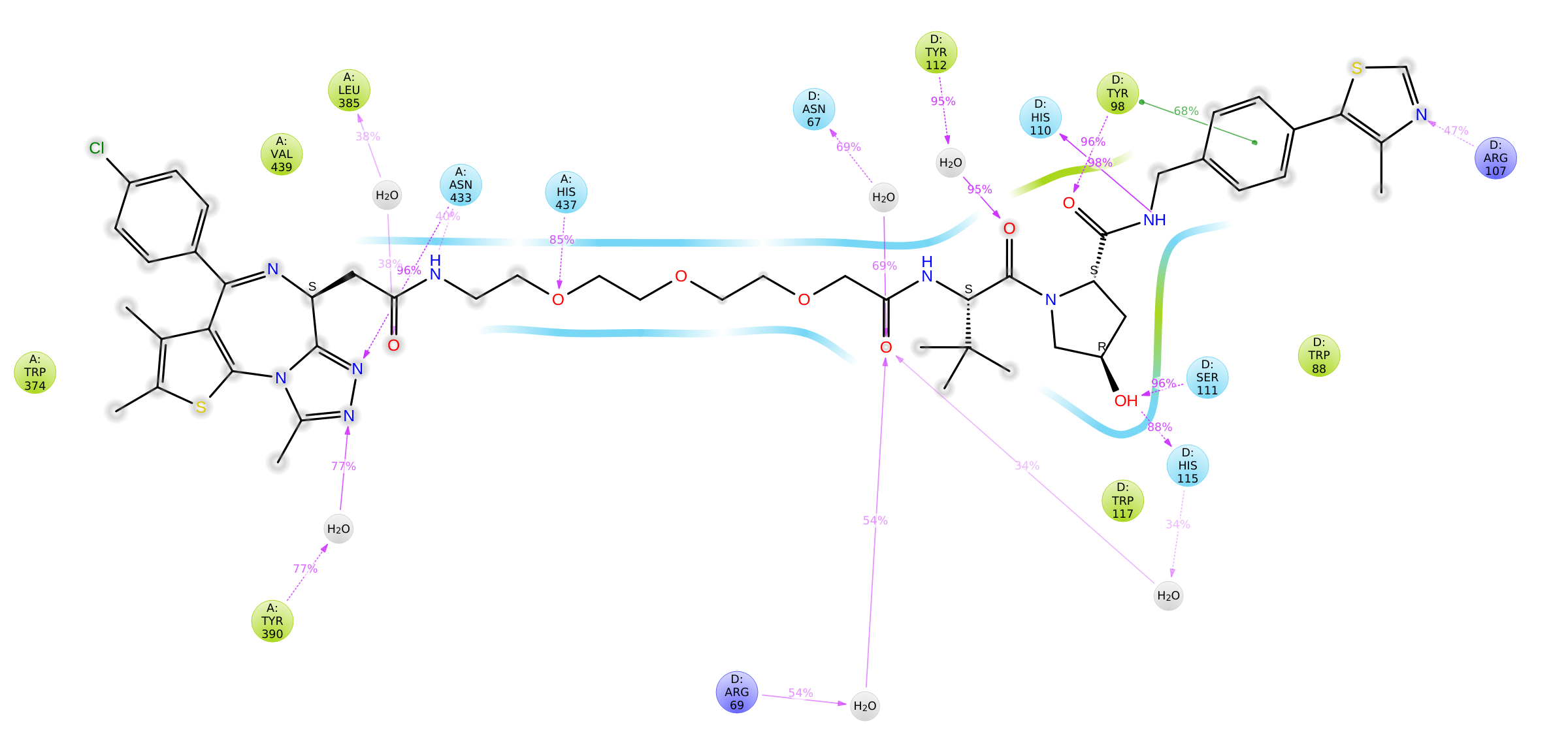 |
| --- | --- |
| 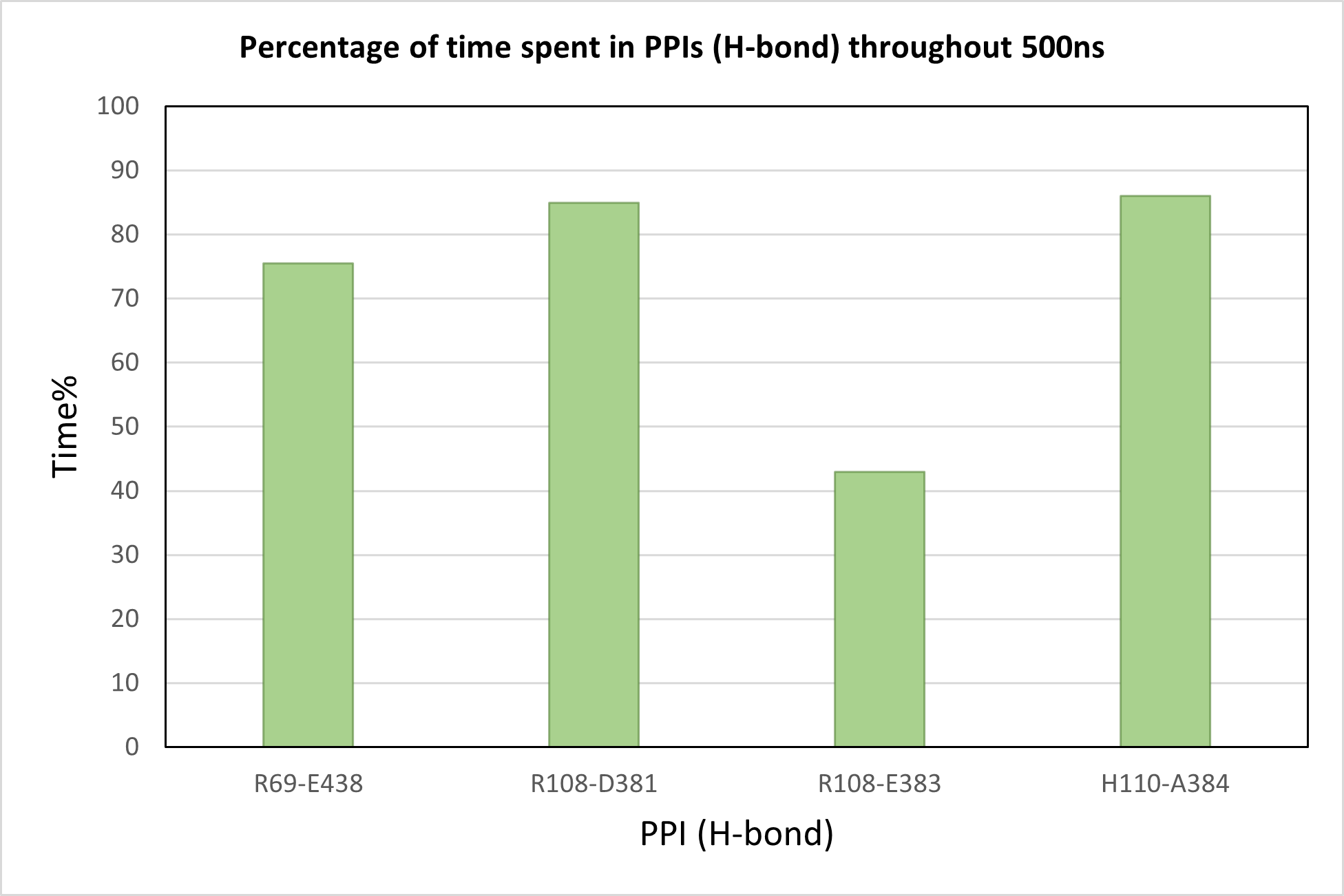 | 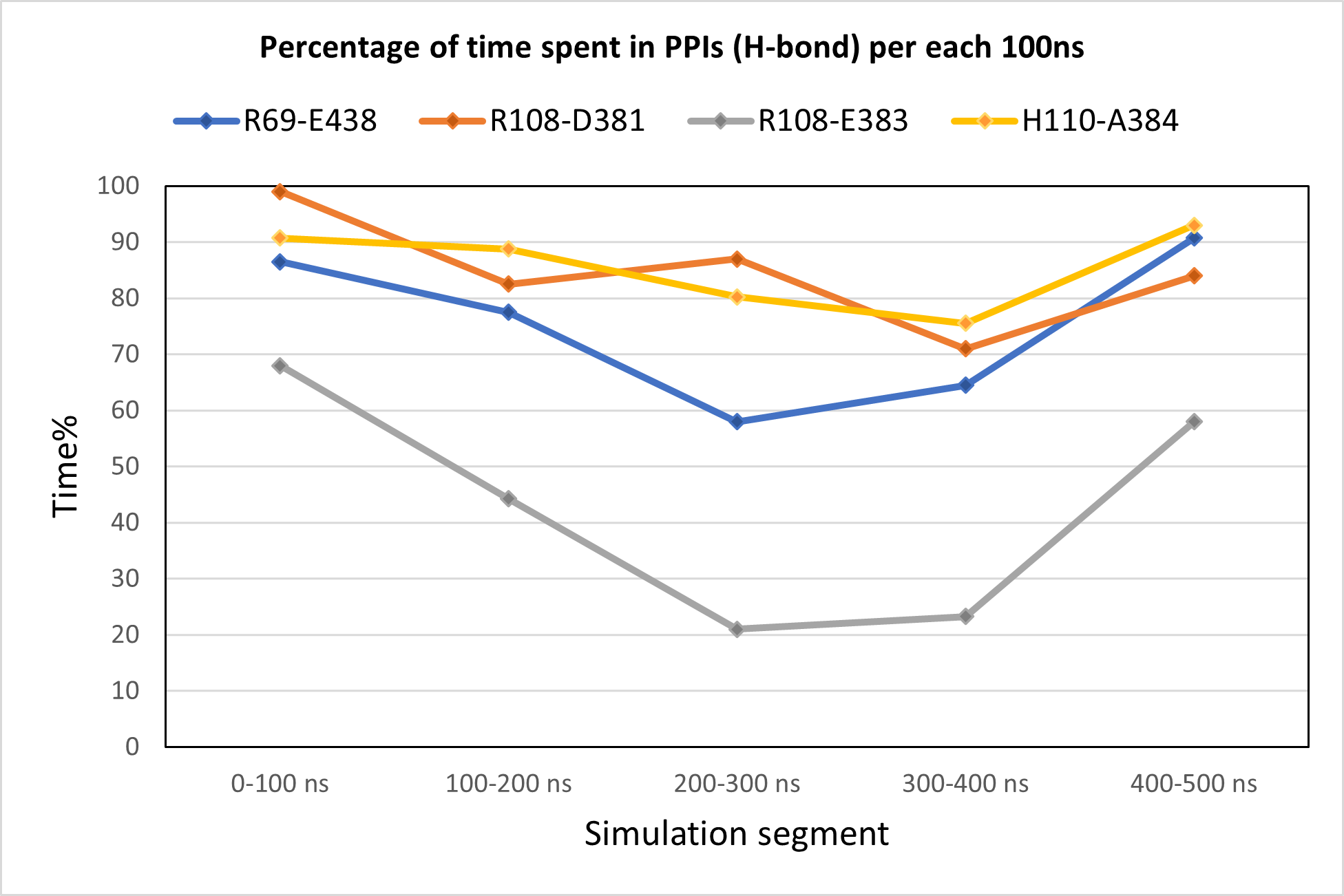 |

**Figure S16:** Analysis of the 500 ns MD simulation of the crystal BRD4BD2-MZ1-VHL ternary complex (PDB ID: 5T35). (A) RMSD values of the protein Cα (orange) and MZ1 fitting on the protein Cα (green). (B) Schematic representation of detailed MZ1 atom interactions with protein residues. (C) and (D) show the percentage of time spent in PPIs (H-bonds) over the entire 500 ns and in each 100 ns interval, respectively.

(D)

(C)

(B)

(A)

(D)

(C)

(B)

(A)

| 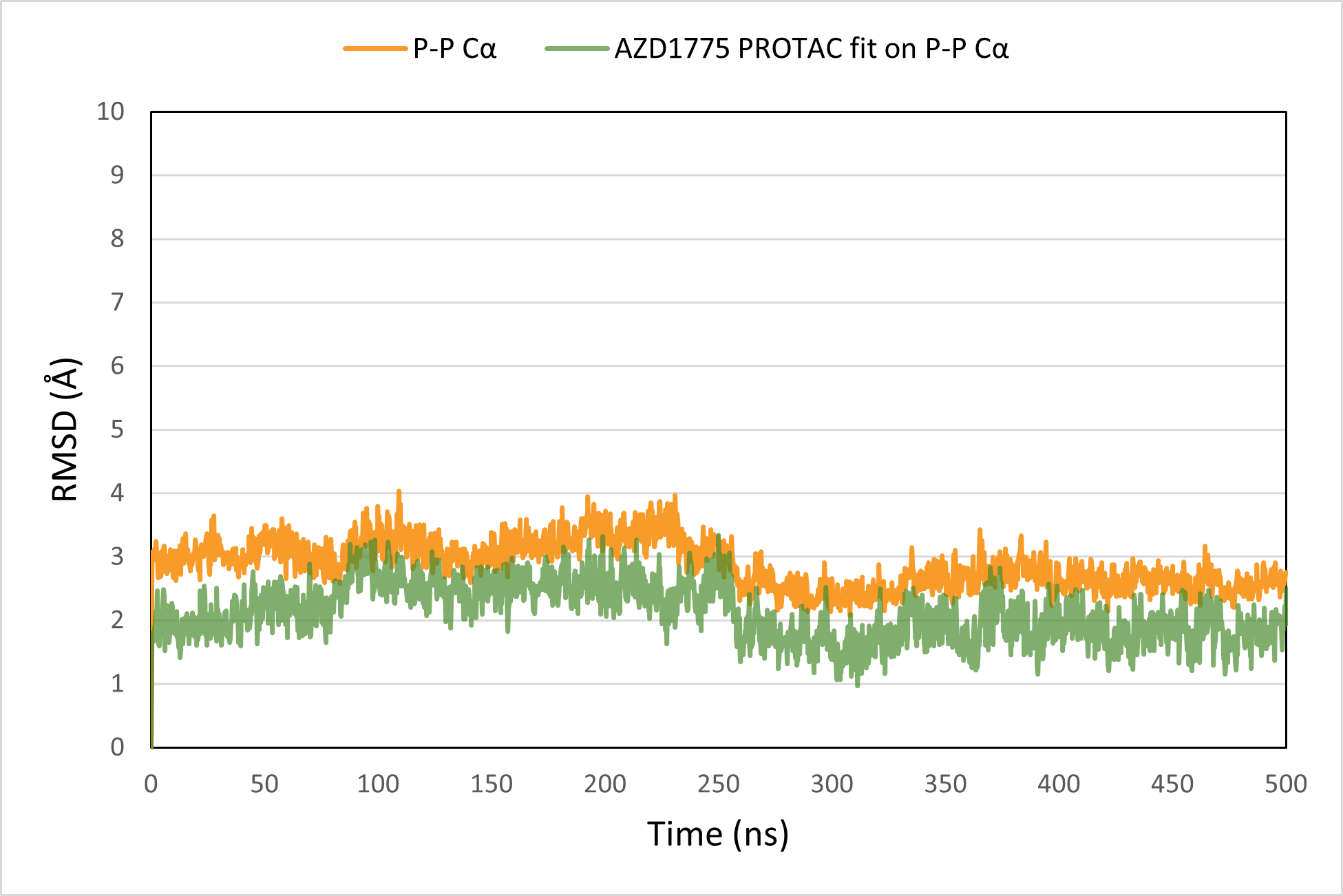 | 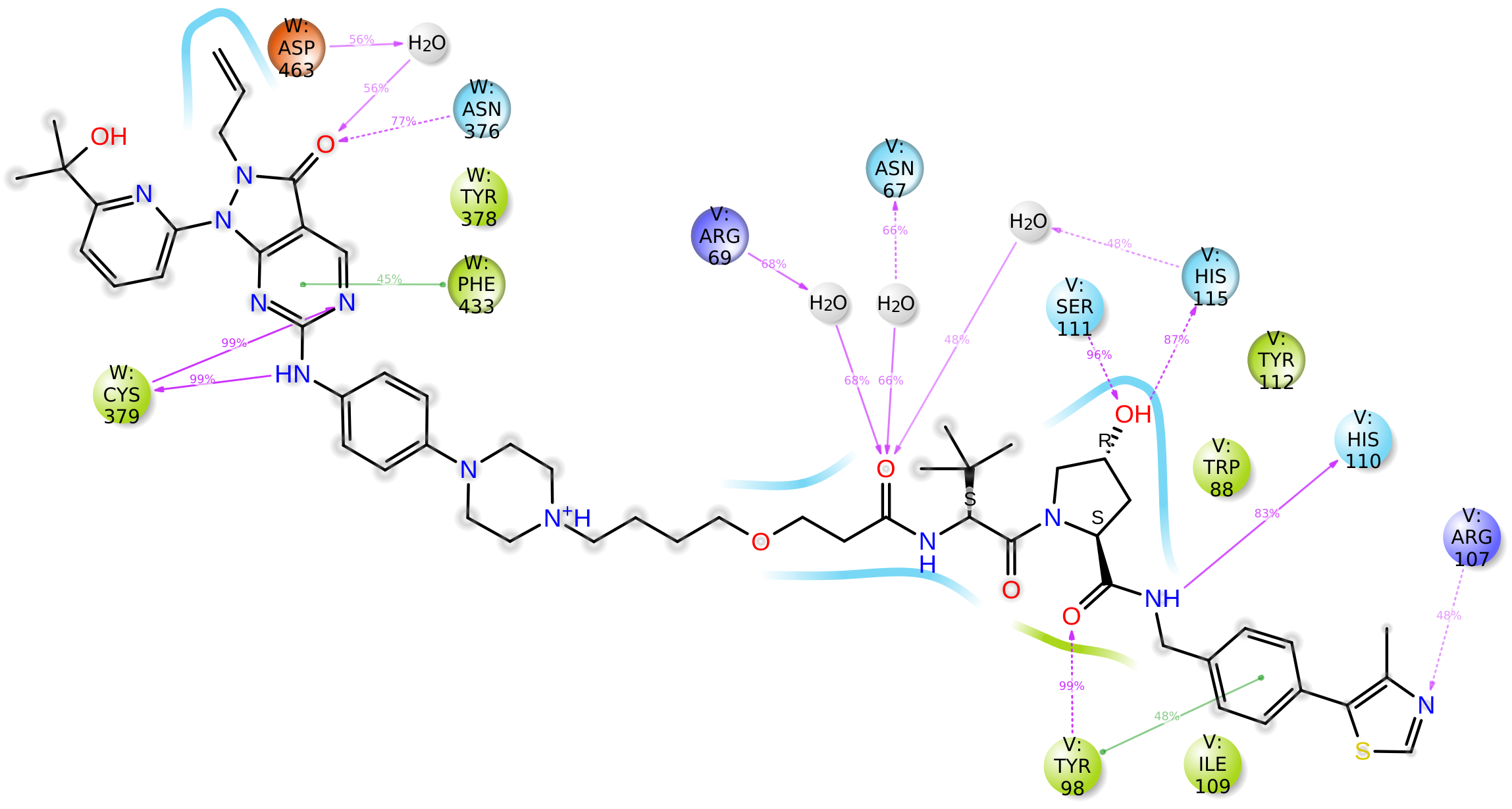 |
| --- | --- |
| 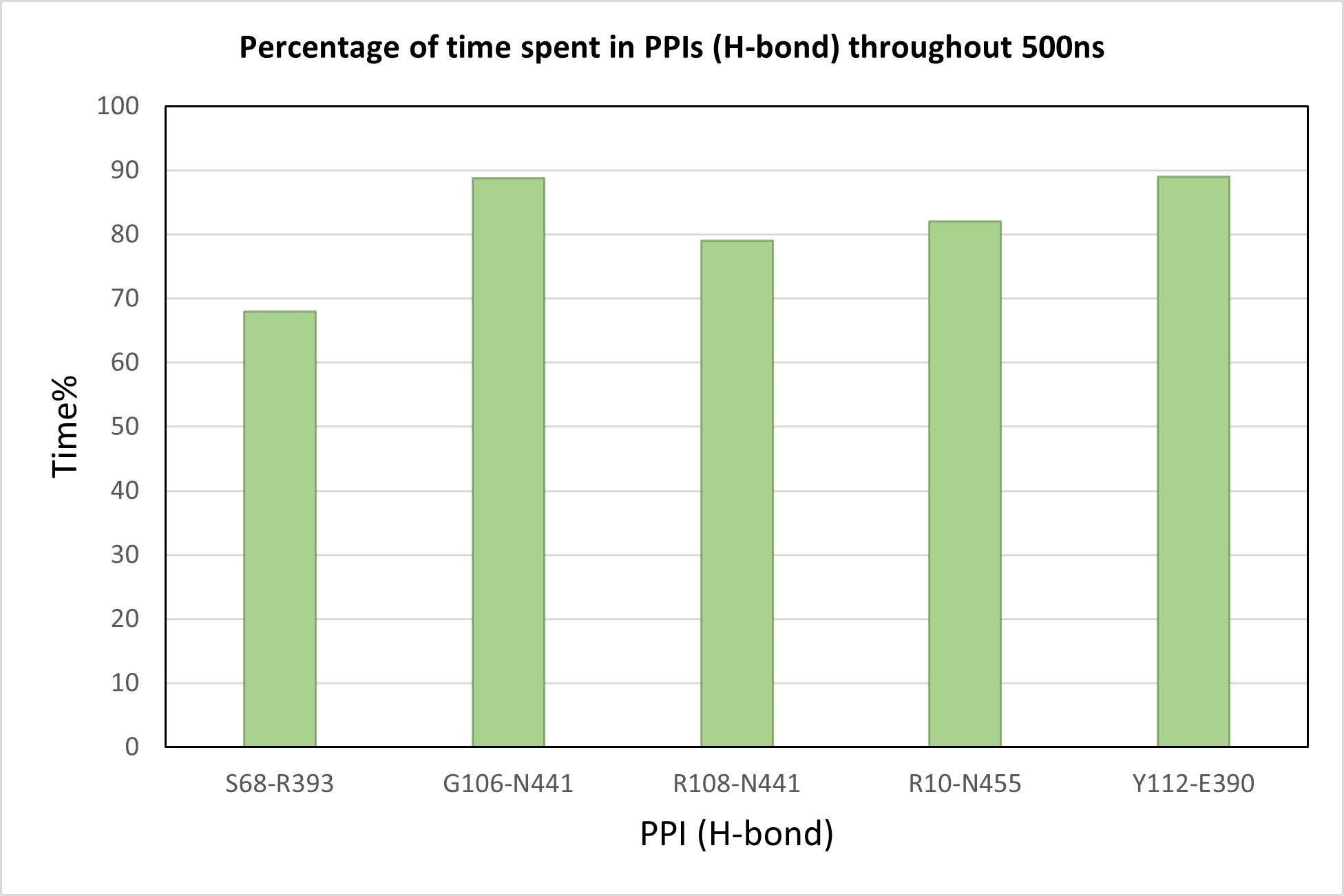 | 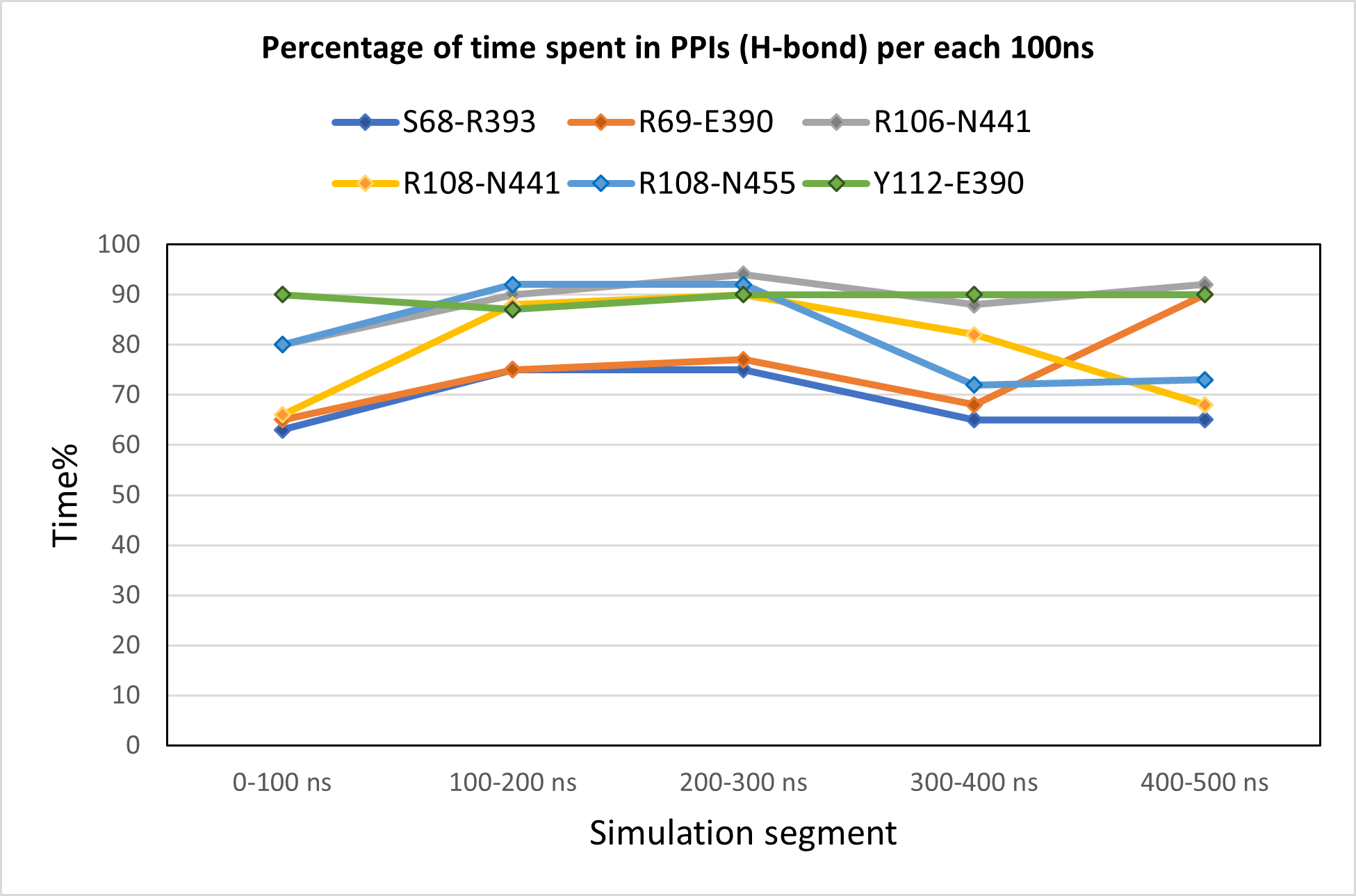 |

**Figure S17:** Analysis of the 500 ns MD simulation of the experimental WEE1-AZD1775 PROTAC-VHL ternary complex (PDB ID: 8WDK). (A) RMSD values of the protein Cα (orange) and AZD1775 PROTAC fitting on the protein Cα (green). (B) Schematic representation of detailed AZD1775 PROTAC atom interactions with protein residues. (C) and (D) show the percentage of time spent in PPIs (H-bonds) over the entire 500 ns and in each 100 ns interval, respectively.

(D)

(C)

(B)

(A)

| 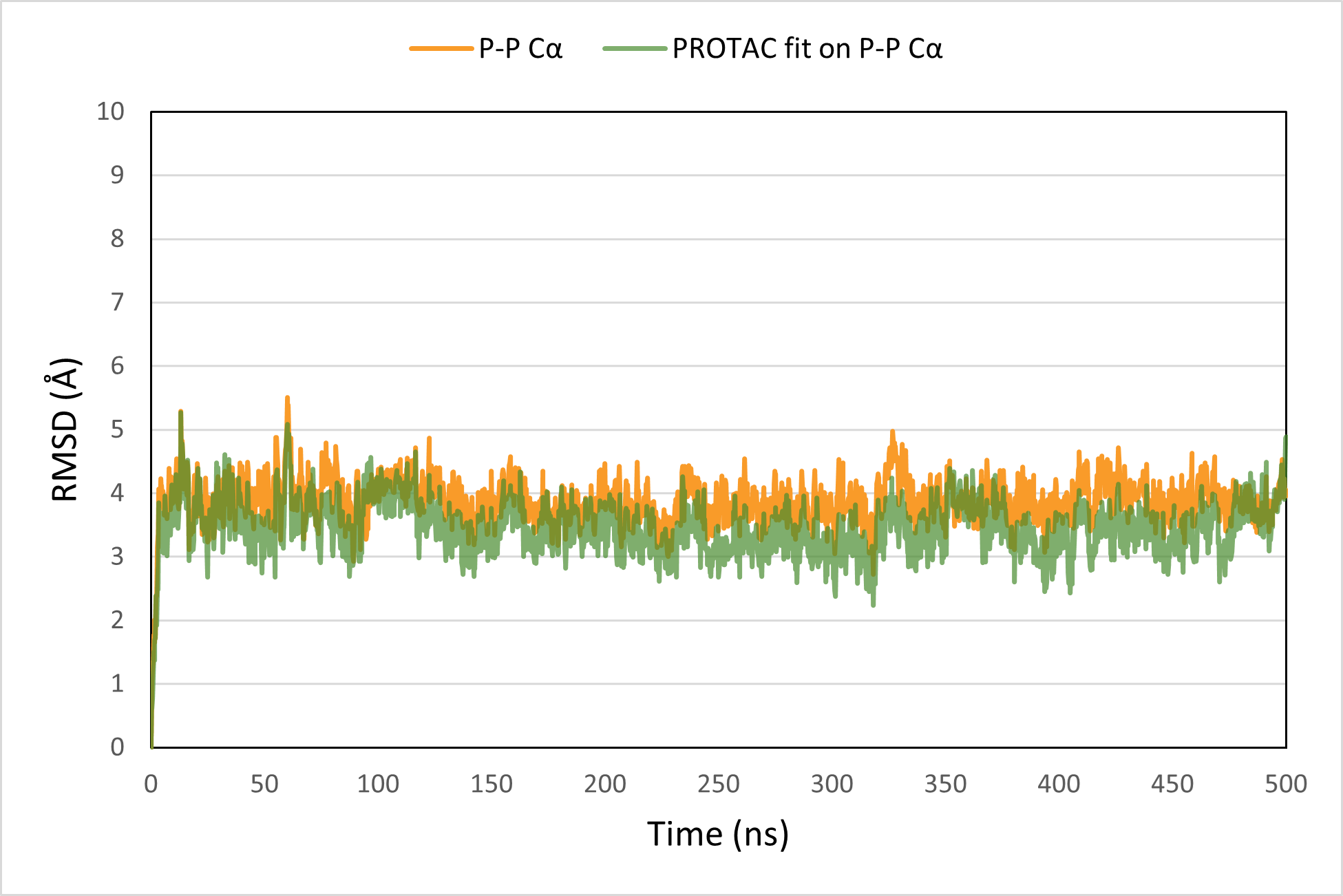 | 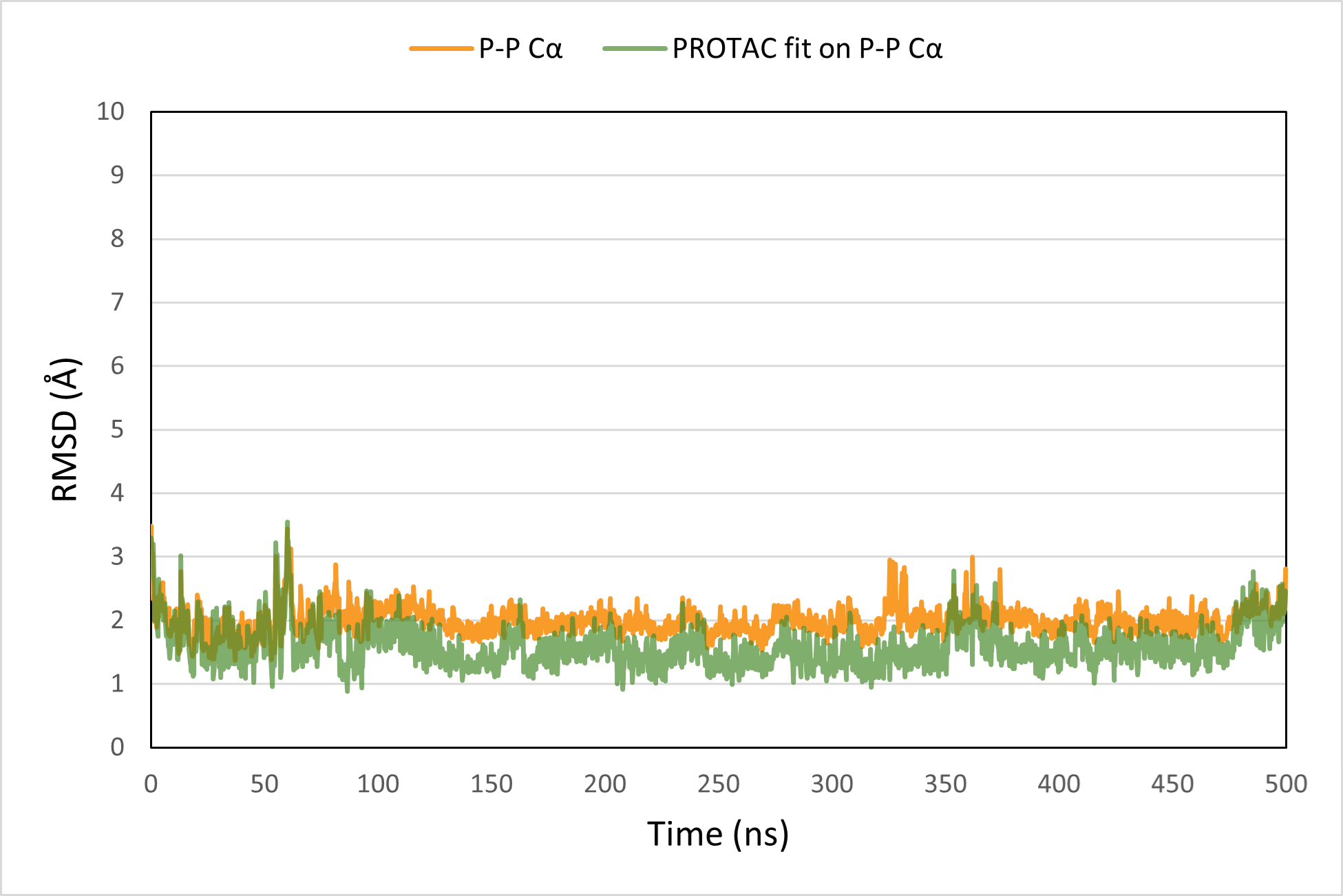 |
| --- | --- |
| 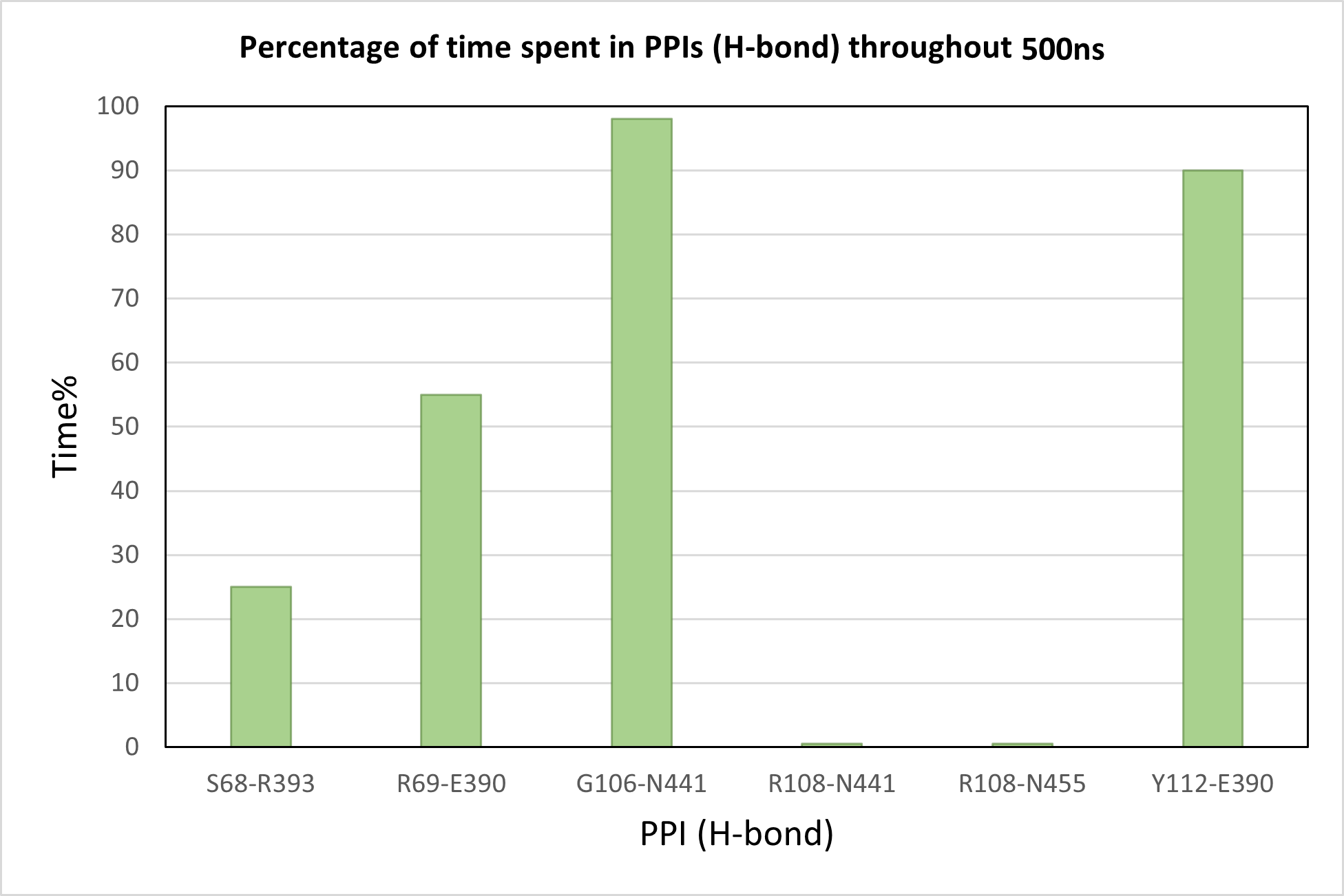 | 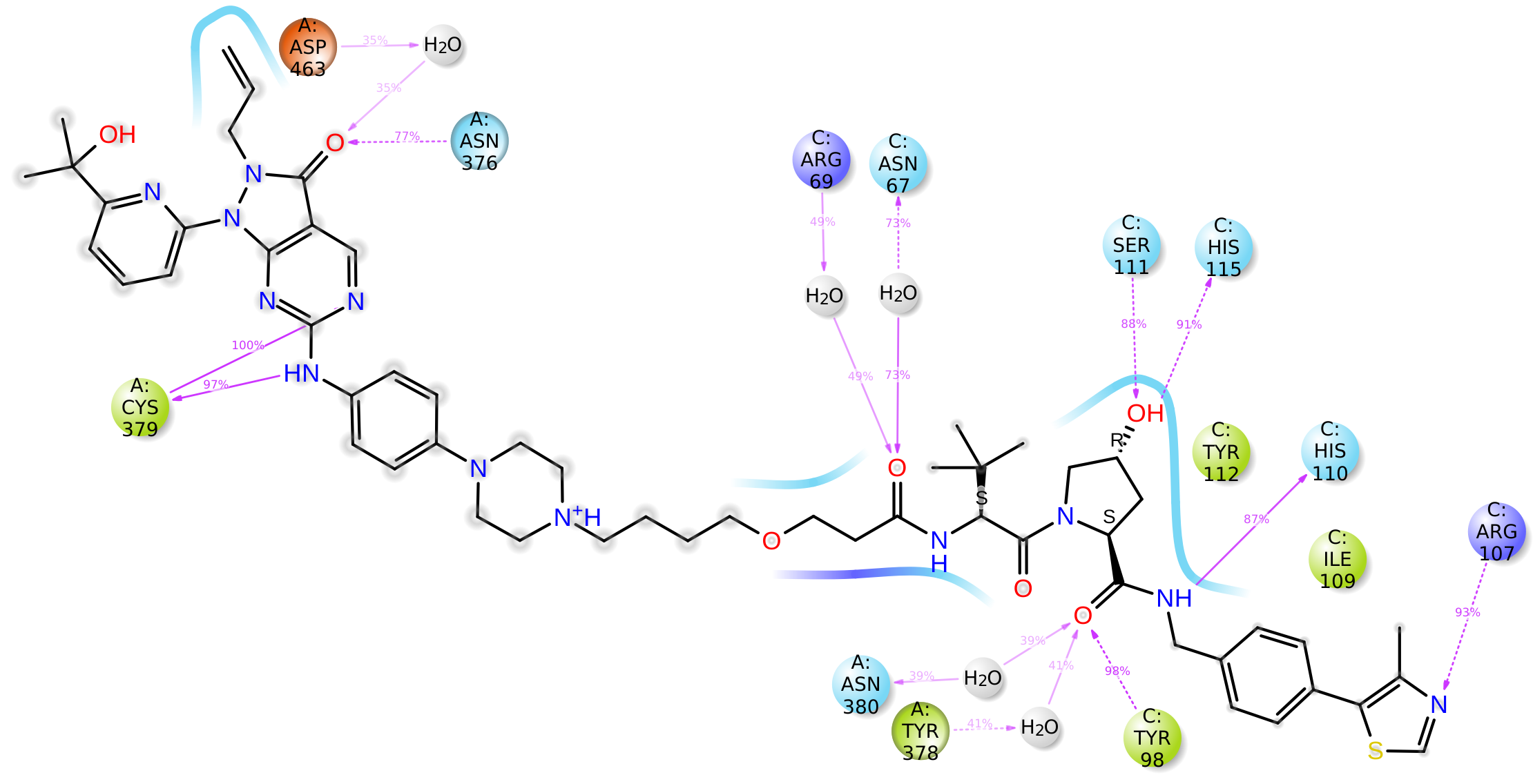 |

**Figure S18:** Analysis of 500 ns MD simulation of the modelled WEE1-AZD1775 PROTAC-VHL ternary complex obtained by Method 4B. (A) and (B) show RMSD values of the protein Cα (orange) and PROTAC fitting on protein Cα (green) using frame 0 and crystal structure as references, repectively. (C) The percentage of time spent in PPIs (H-bonds) over the entire 500 ns. (D) Schematic representation of detailed PROTAC atom interactions with protein residues.

(E)

(D)

(C)

(F)

| 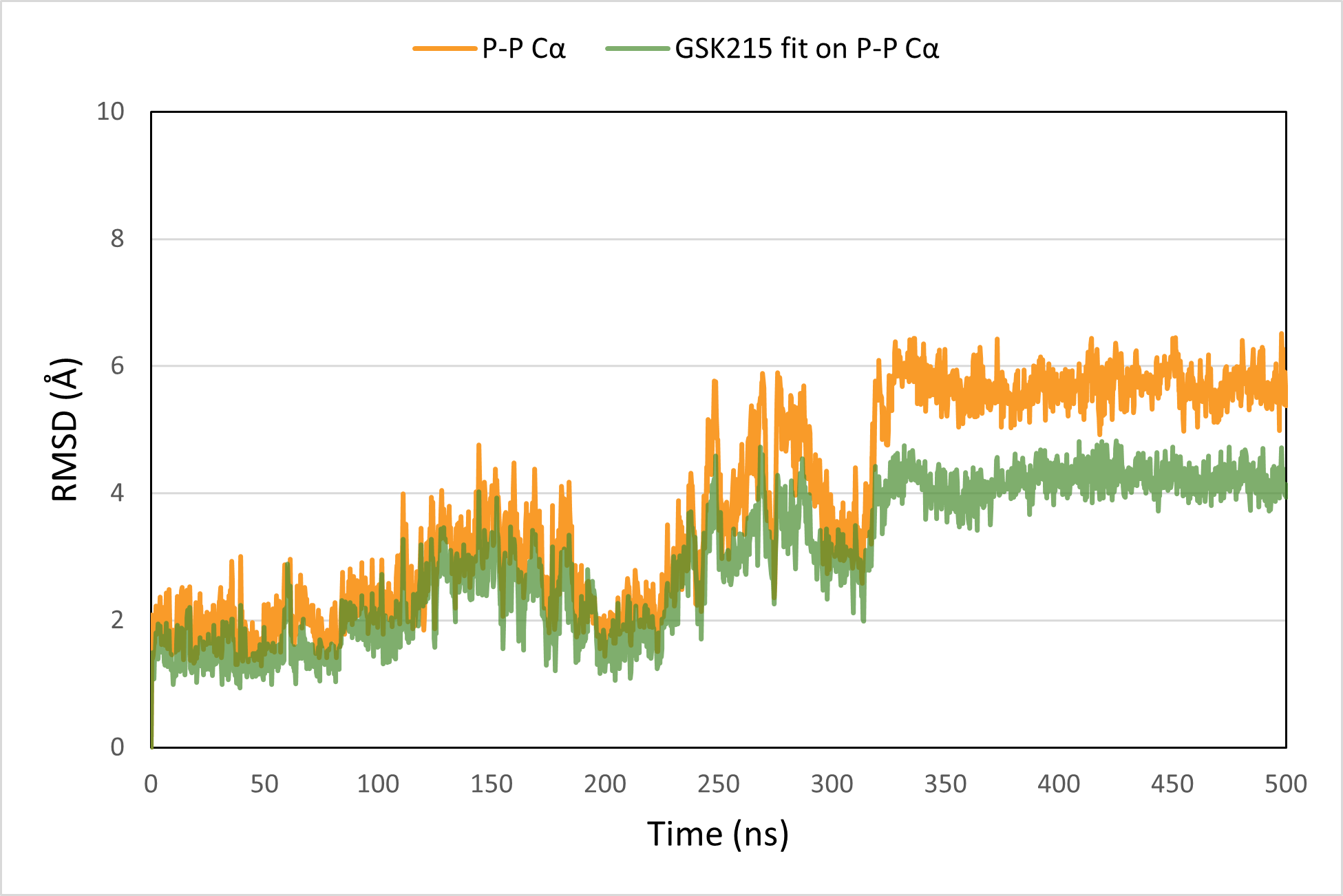 | 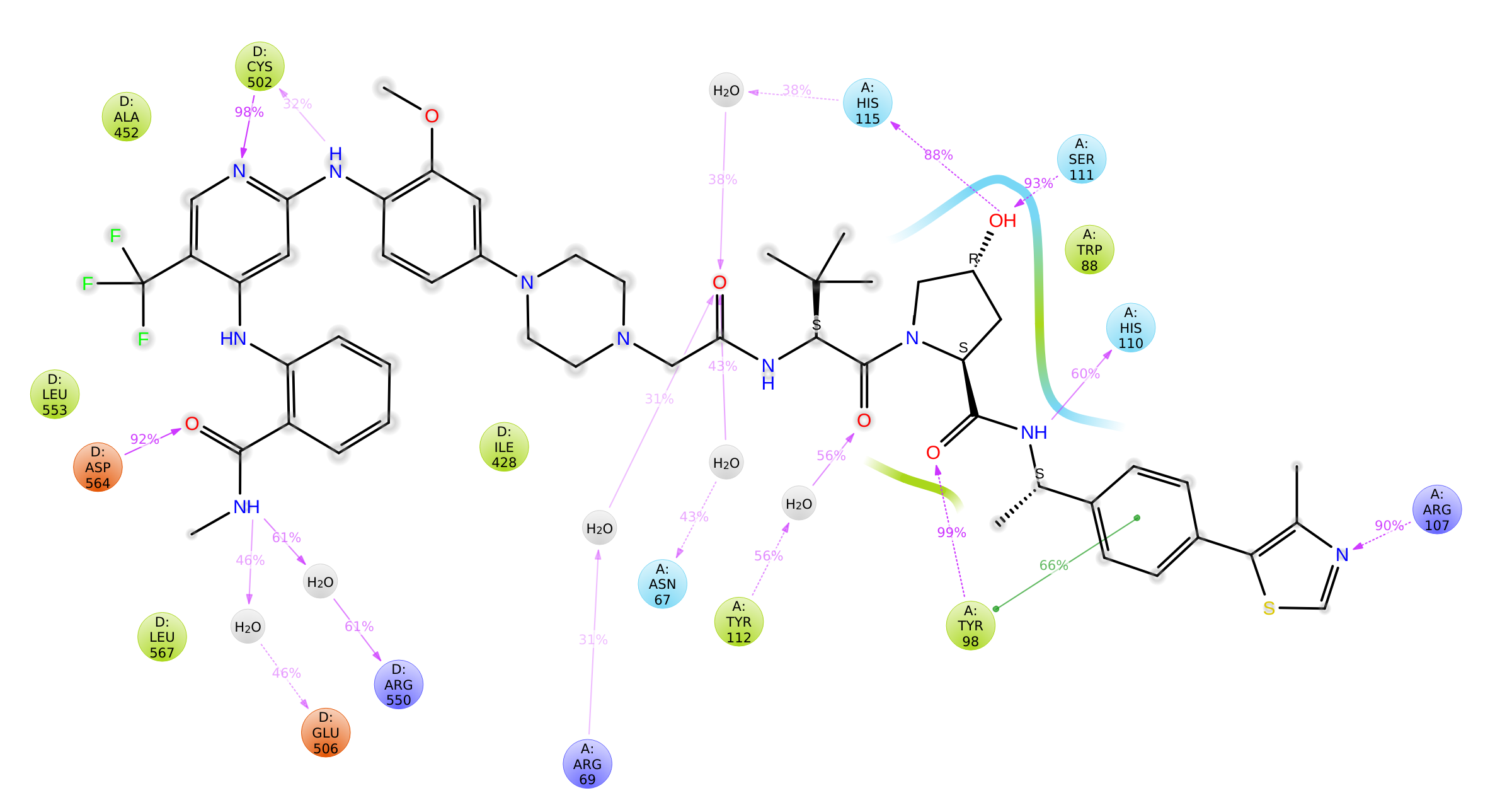 |
| --- | --- |
| 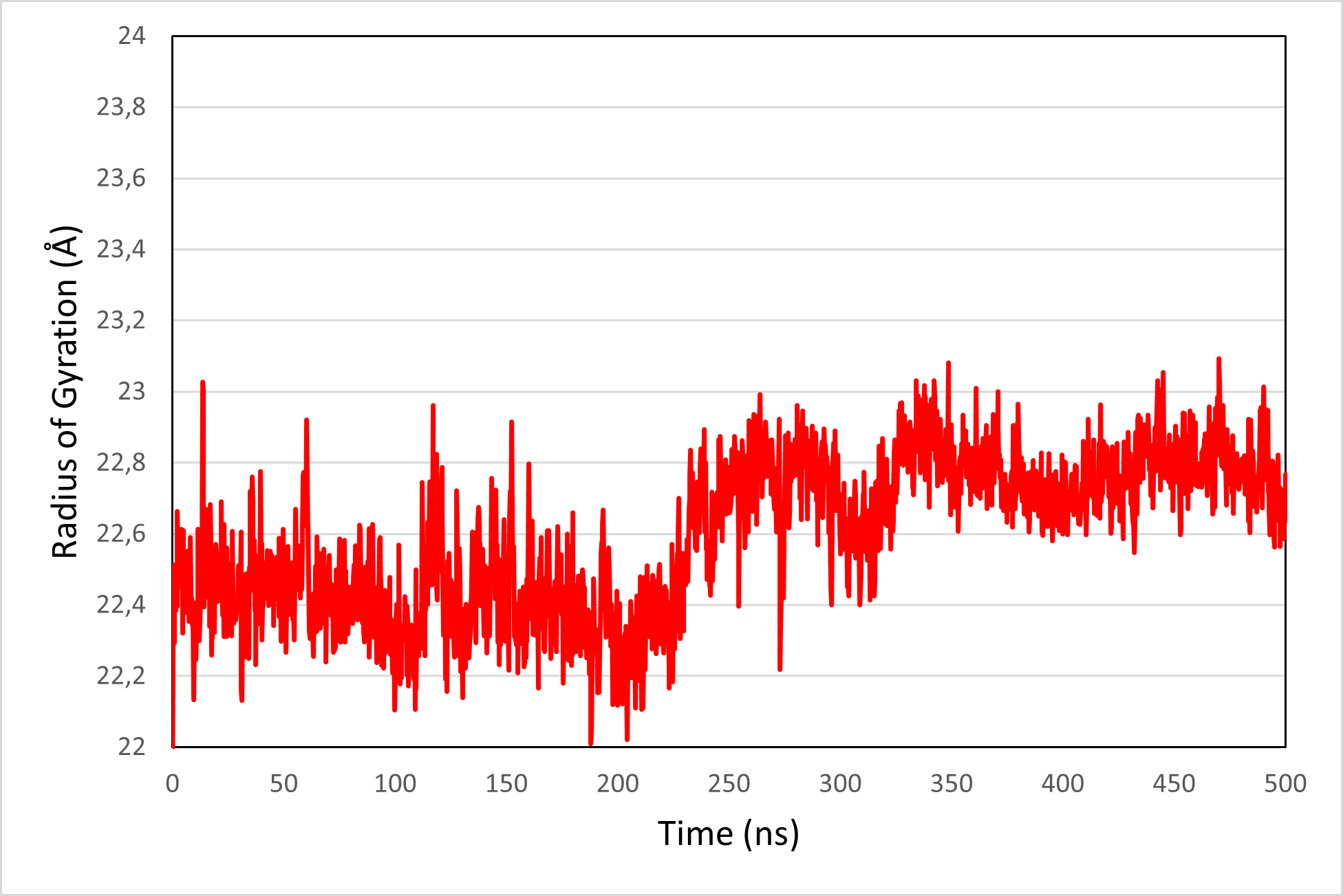 | 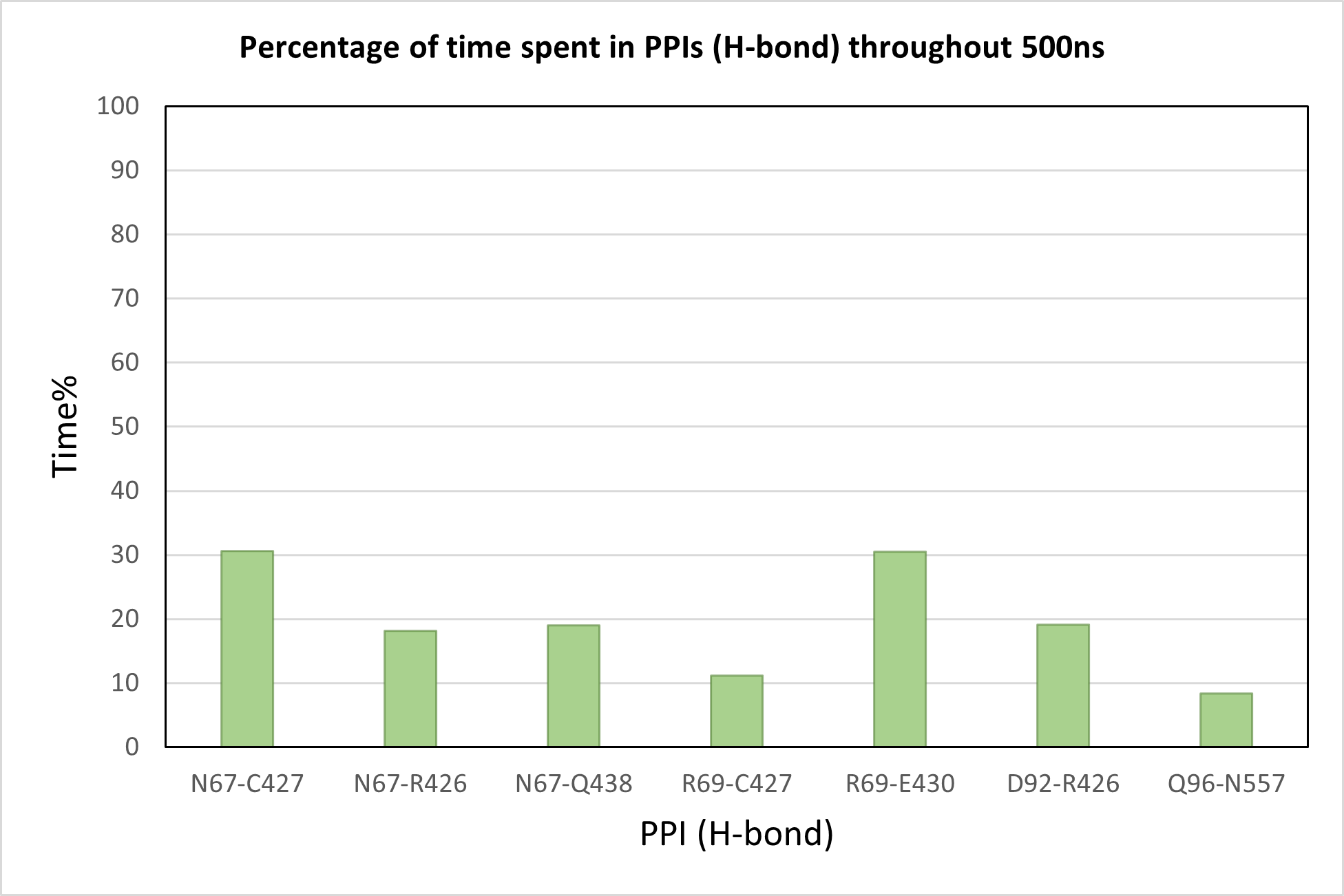 |
| 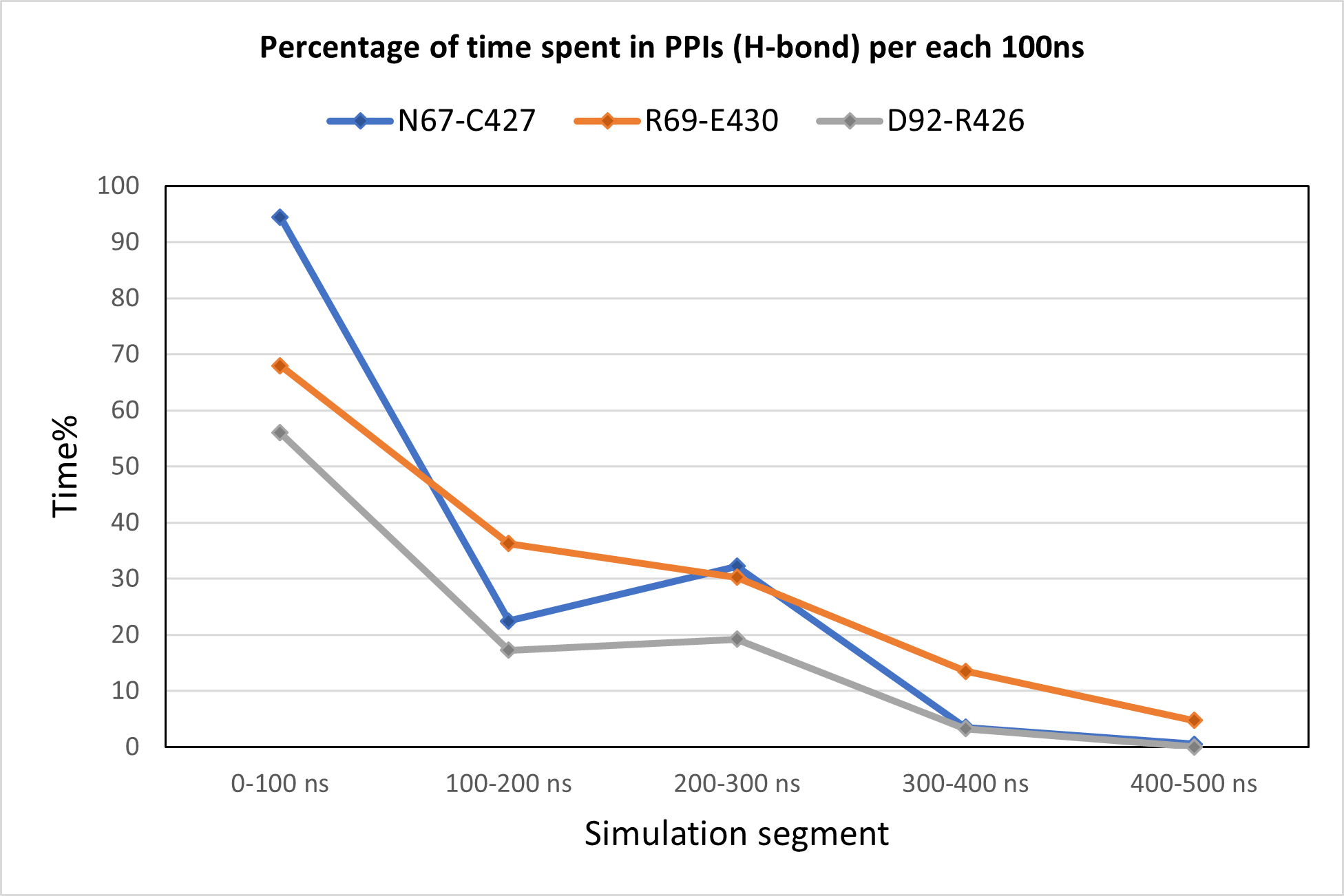 | 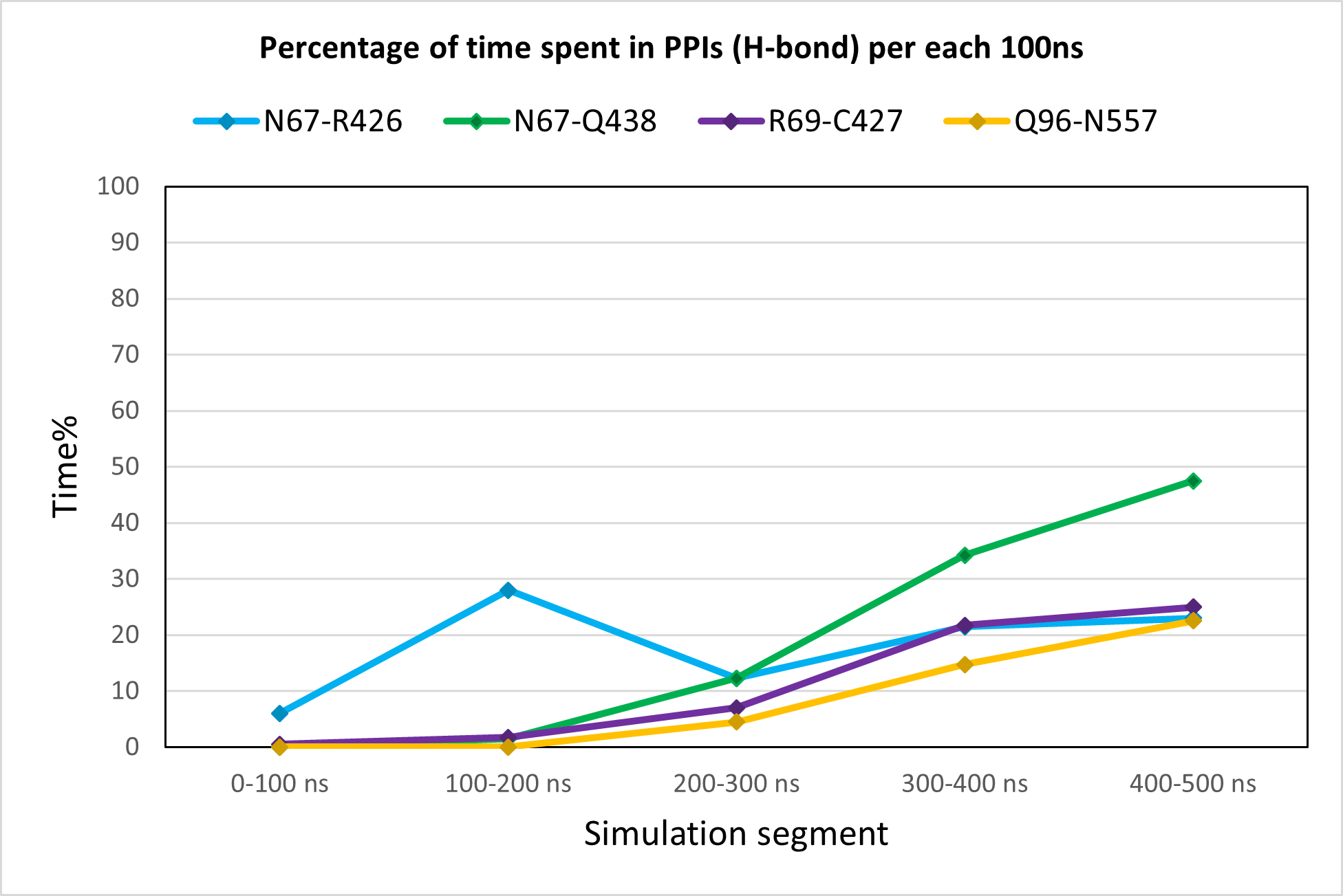 |

**Figure S19:** Analysis of the 500 ns MD simulation of the crystal FAK-GSK215-VHL ternary complex (PDB ID: 7PI4). (A) RMSD values of the protein Cα (orange) and GSK215 fitting on the protein Cα (green). (B) Schematic representation of detailed GSK215 atom interactions with protein residues. (C) Radius of gyration of FAK-GSK215-VHL ternary complex. (D-E) show the percentage of time spent in PPIs (H-bonds) over the entire 500 ns and in each 100 ns interval.

(B)

(A)

| 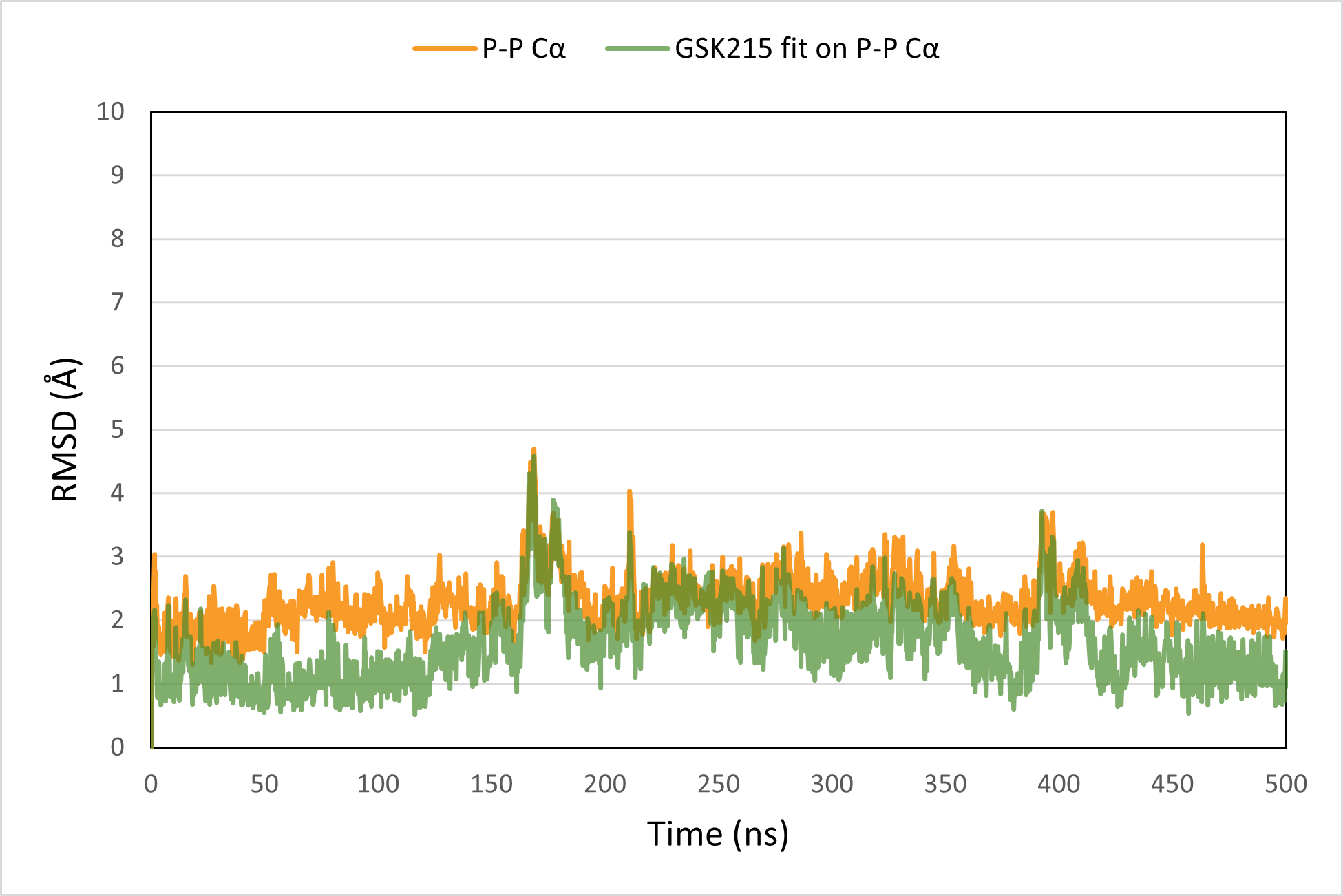 | 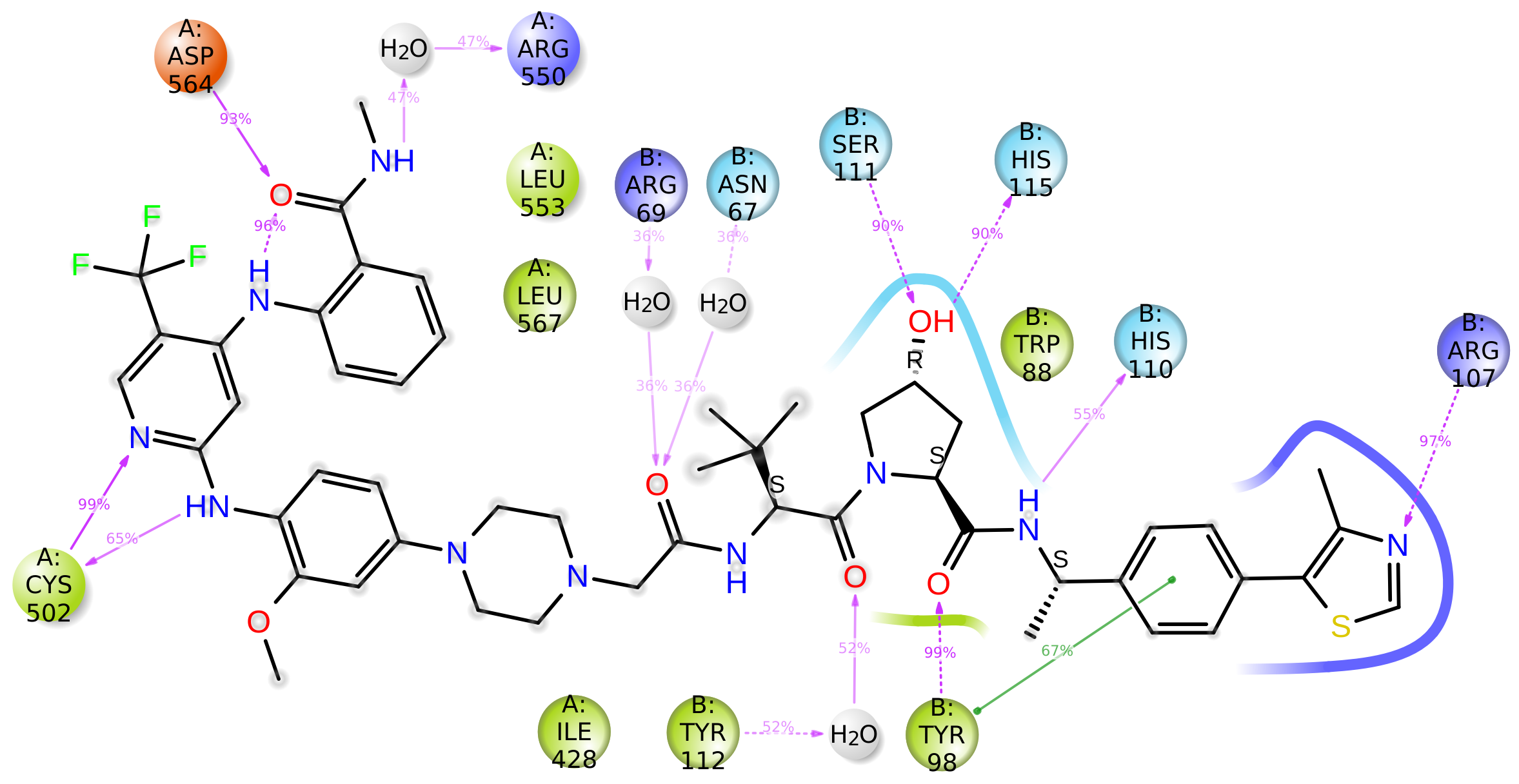 |
| --- | --- |
| 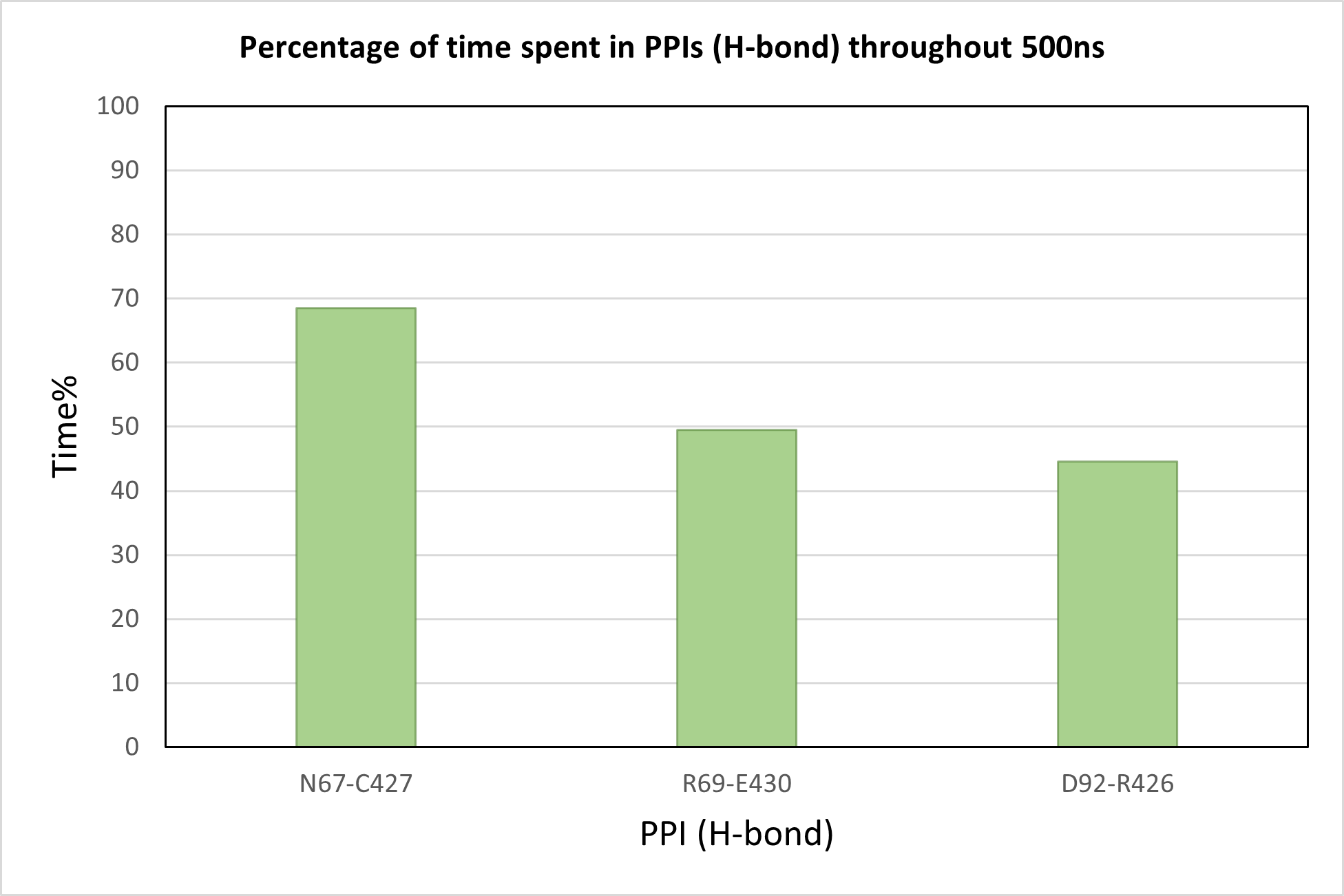 | 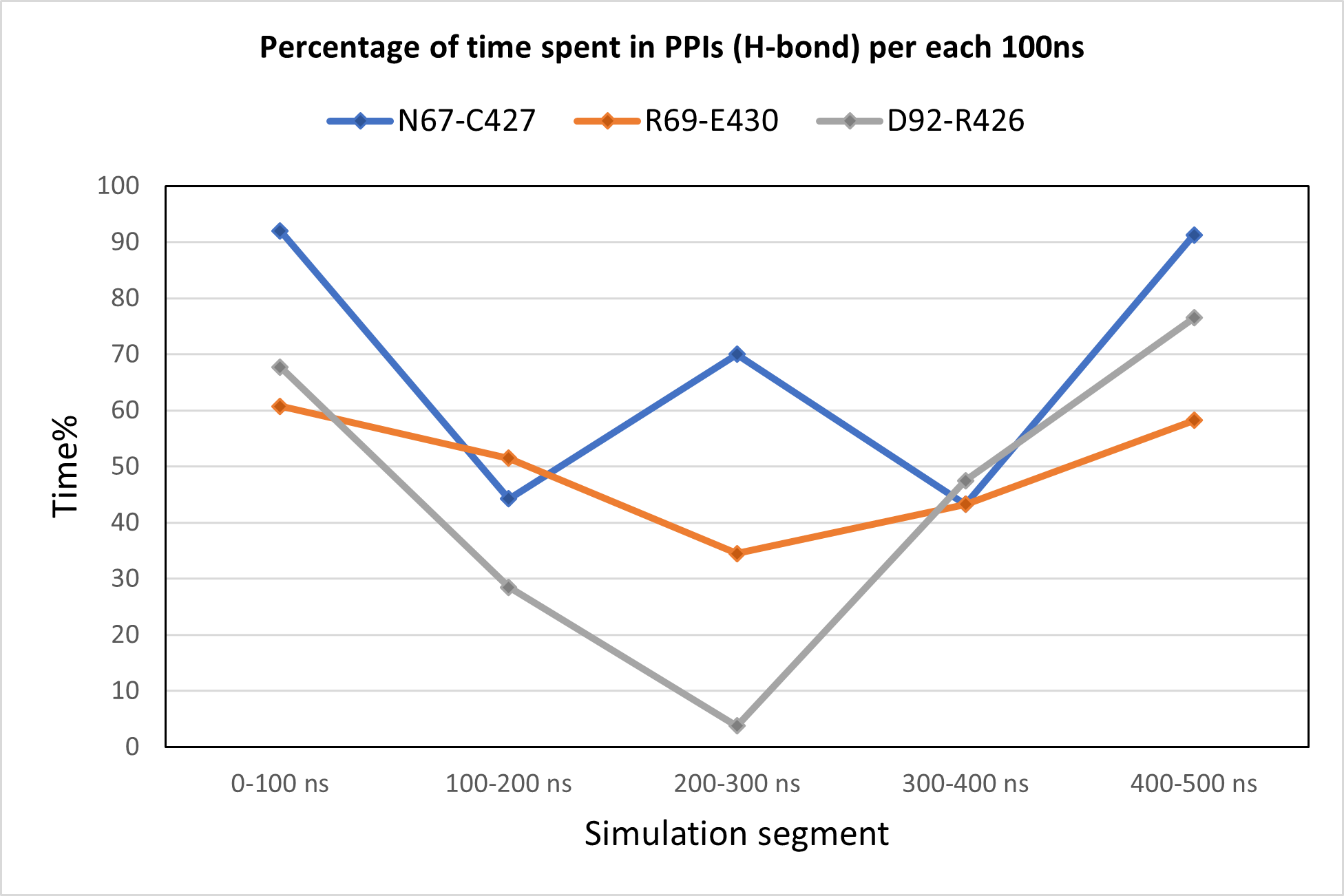 |

**Figure S20:** Analysis of the 500 ns MD simulation of the modified crystal FAK-GSK215-VHL ternary complex. (A) RMSD values of the protein Cα (orange) and GSK215 fitting on the protein Cα (green). (B) Schematic representation of detailed GSK215 atom interactions with protein residues. (C) and (D) show the percentage of time spent in PPIs (H-bonds) over the entire 500 ns and in each 100 ns interval, respectively.

(C)

(D)

(B)

(A)

(D)

| 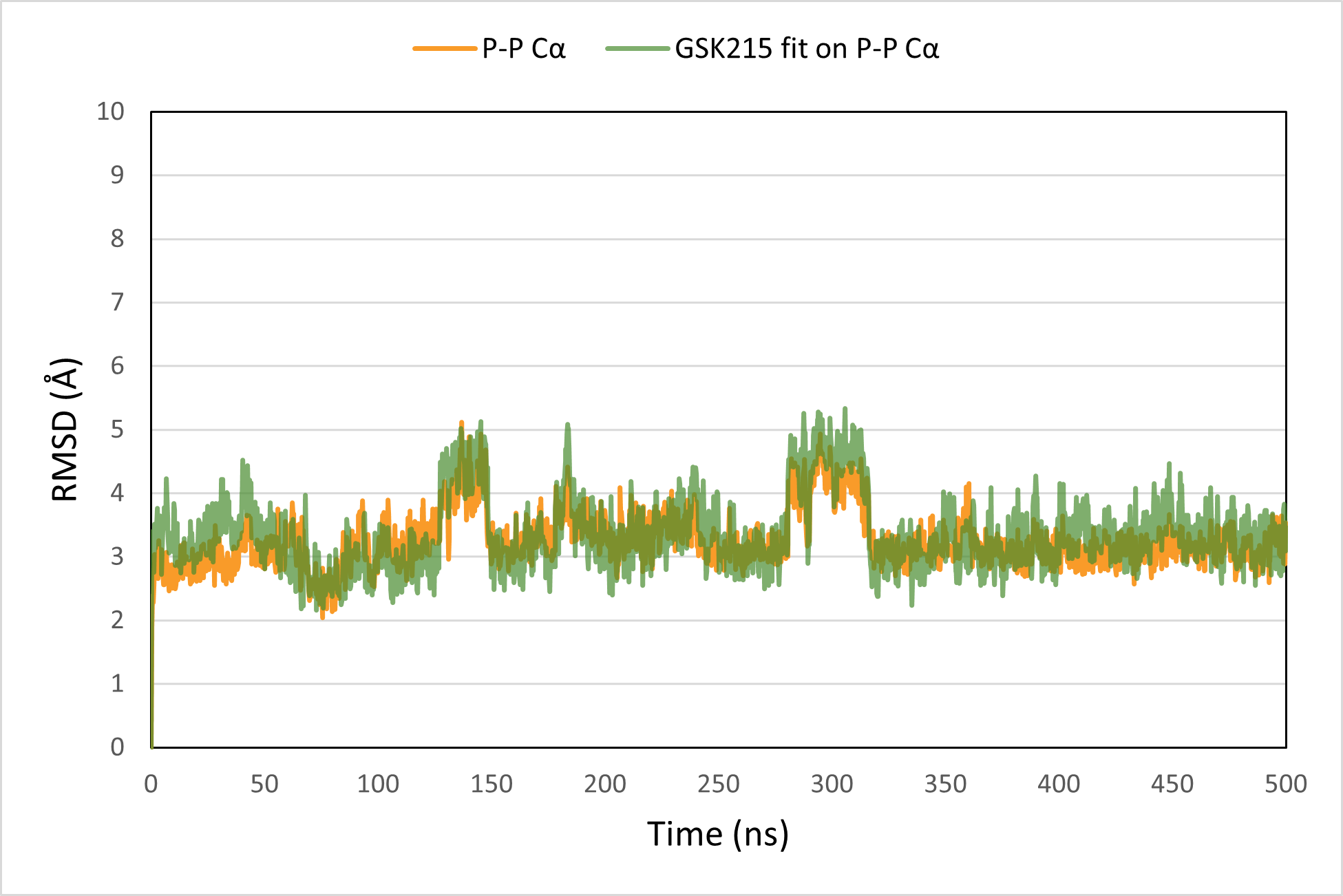 | 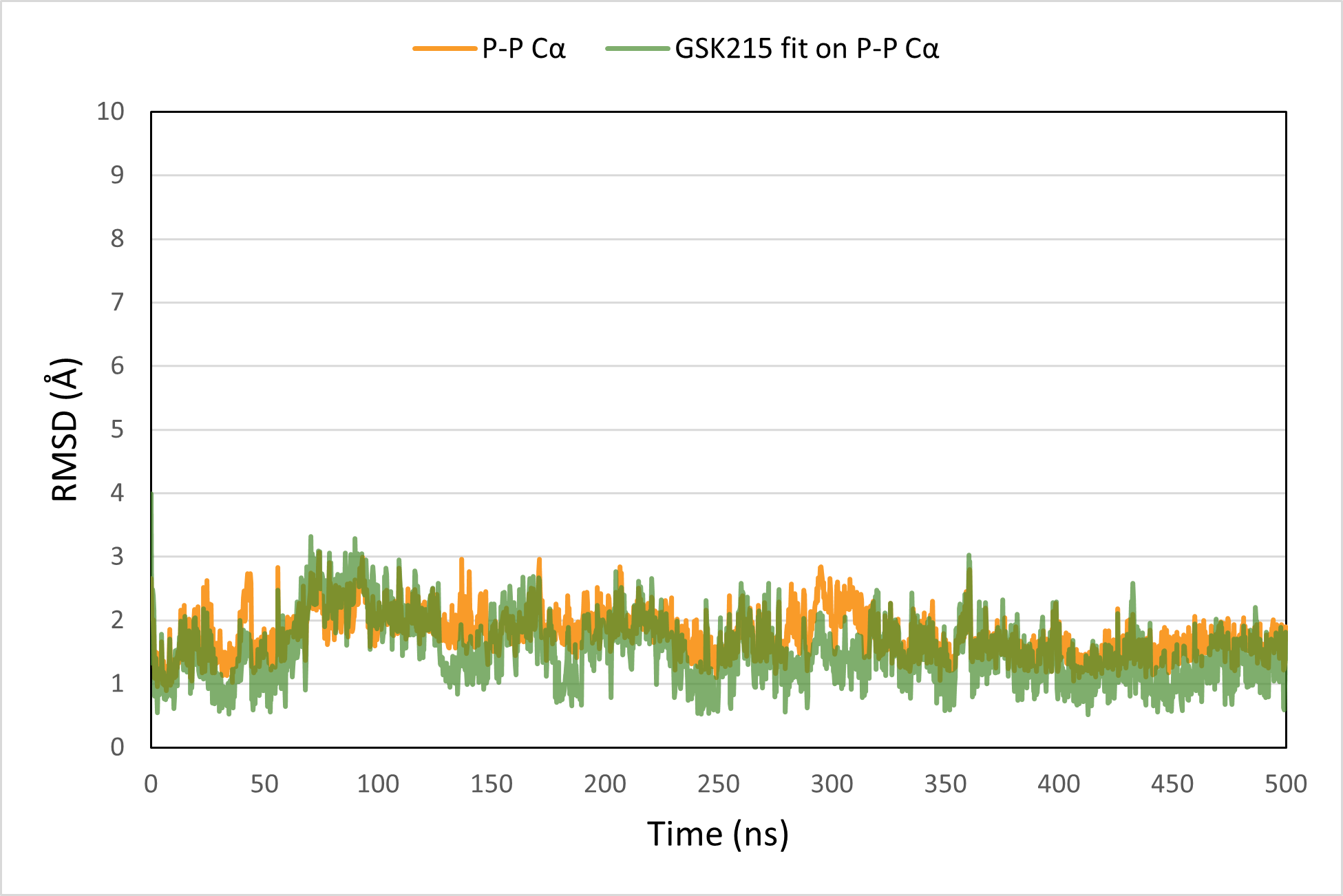 |
| --- | --- |
| 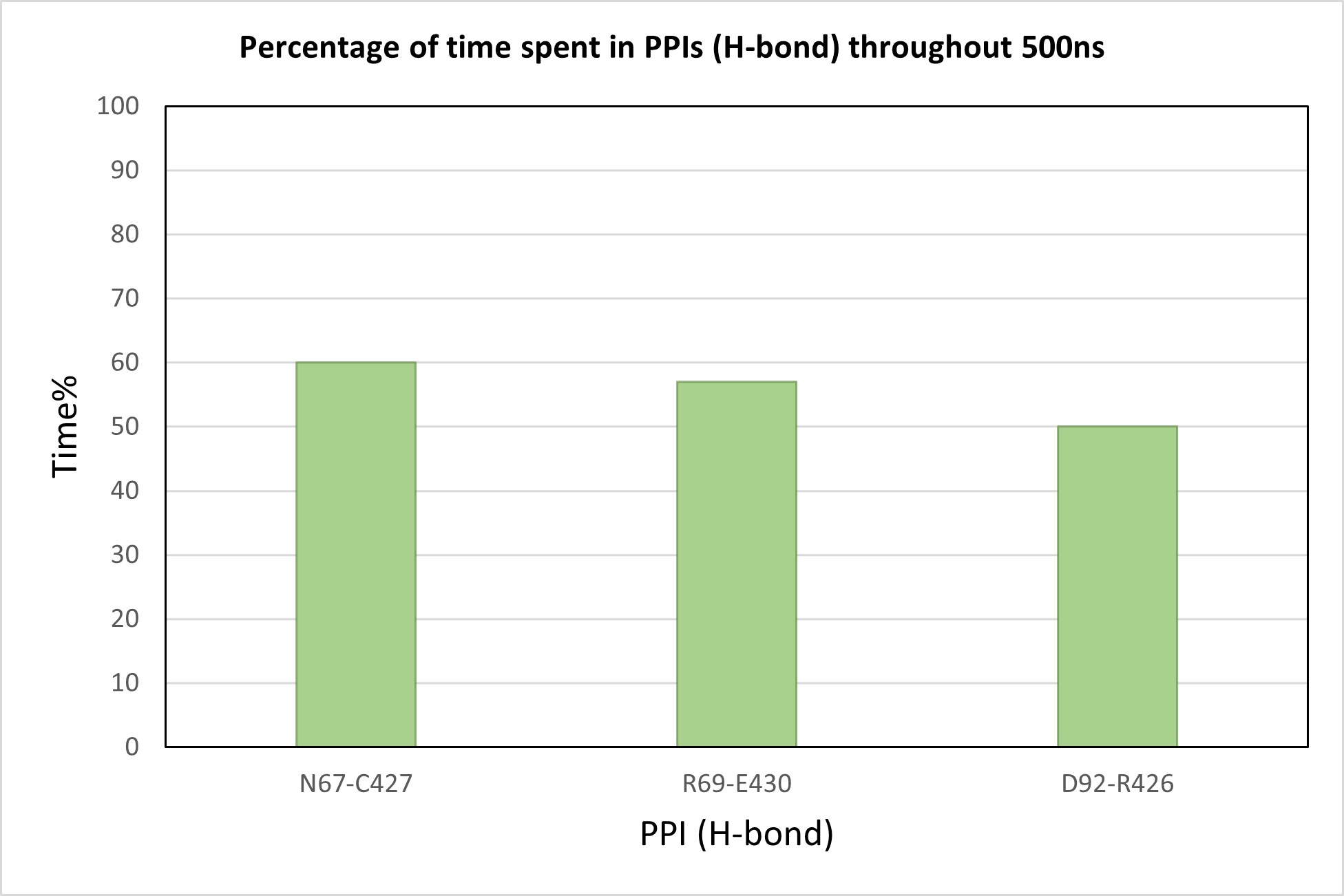 | 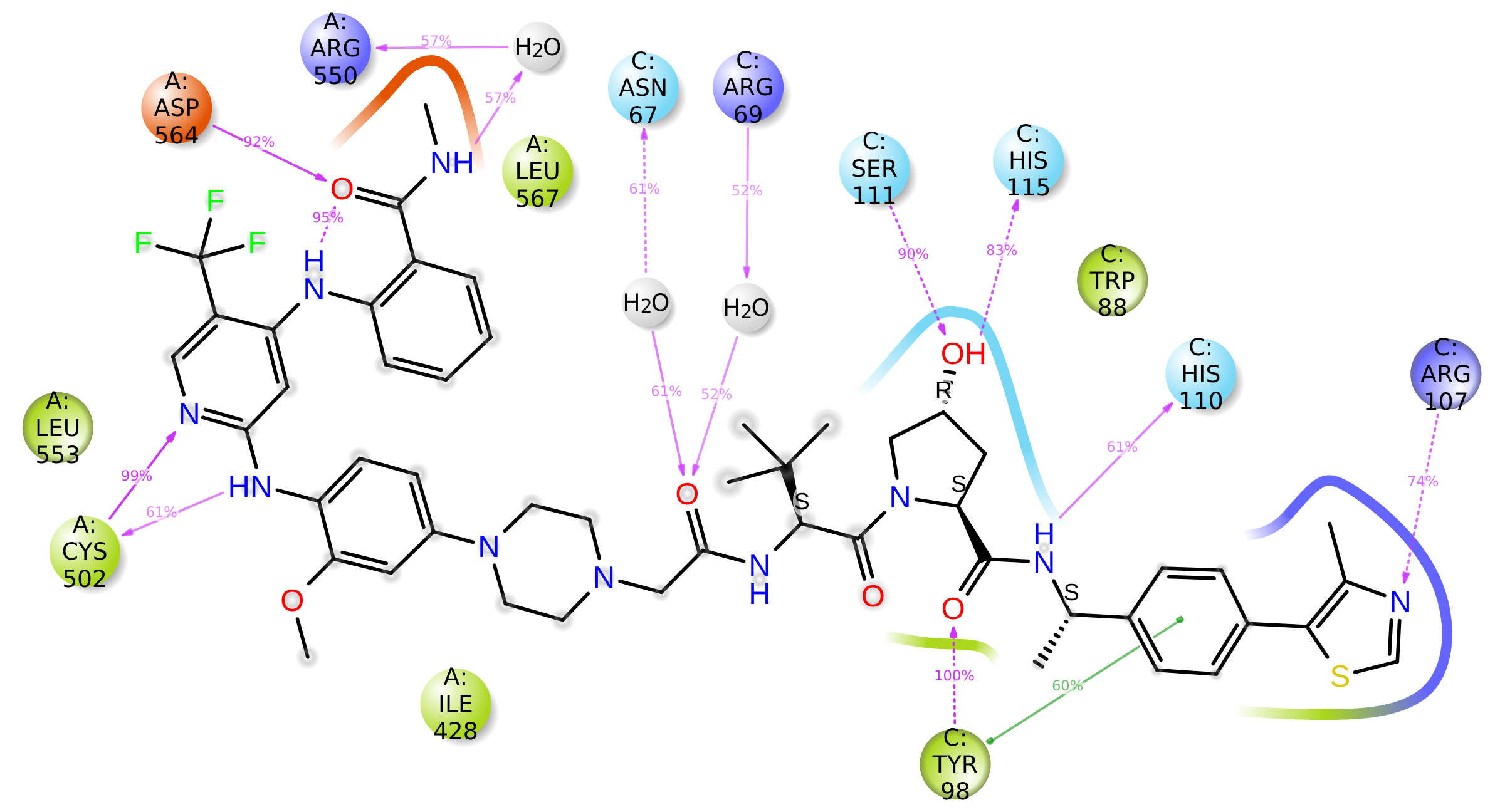 |

**Figure S21:** Analysis of 500 ns MD simulation of the modelled FAK-GSK215-VHL ternary complex obtained by Method 4B. (A) and (B) show RMSD values of the protein Cα (orange) and GSK215 fitting on protein Cα (green) using frame 0 and crystal structure as references, repectively. (C) The percentage of time spent in PPIs (H-bonds) over the entire 500 ns. (D) Schematic representation of detailed PROTAC atom interactions with protein residues.

(C)

(B)

(A)

(B)

(D)

(C)

| 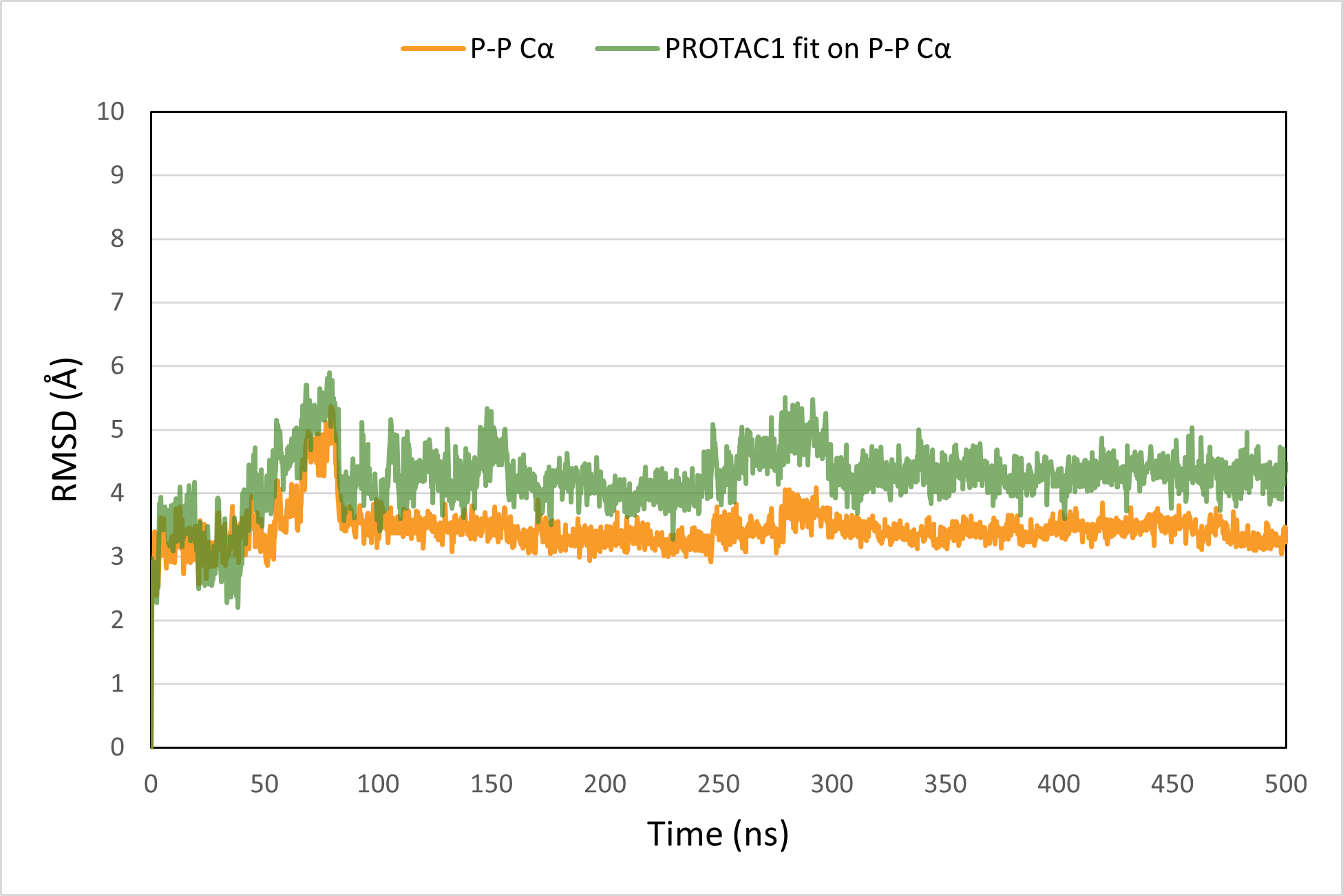 | 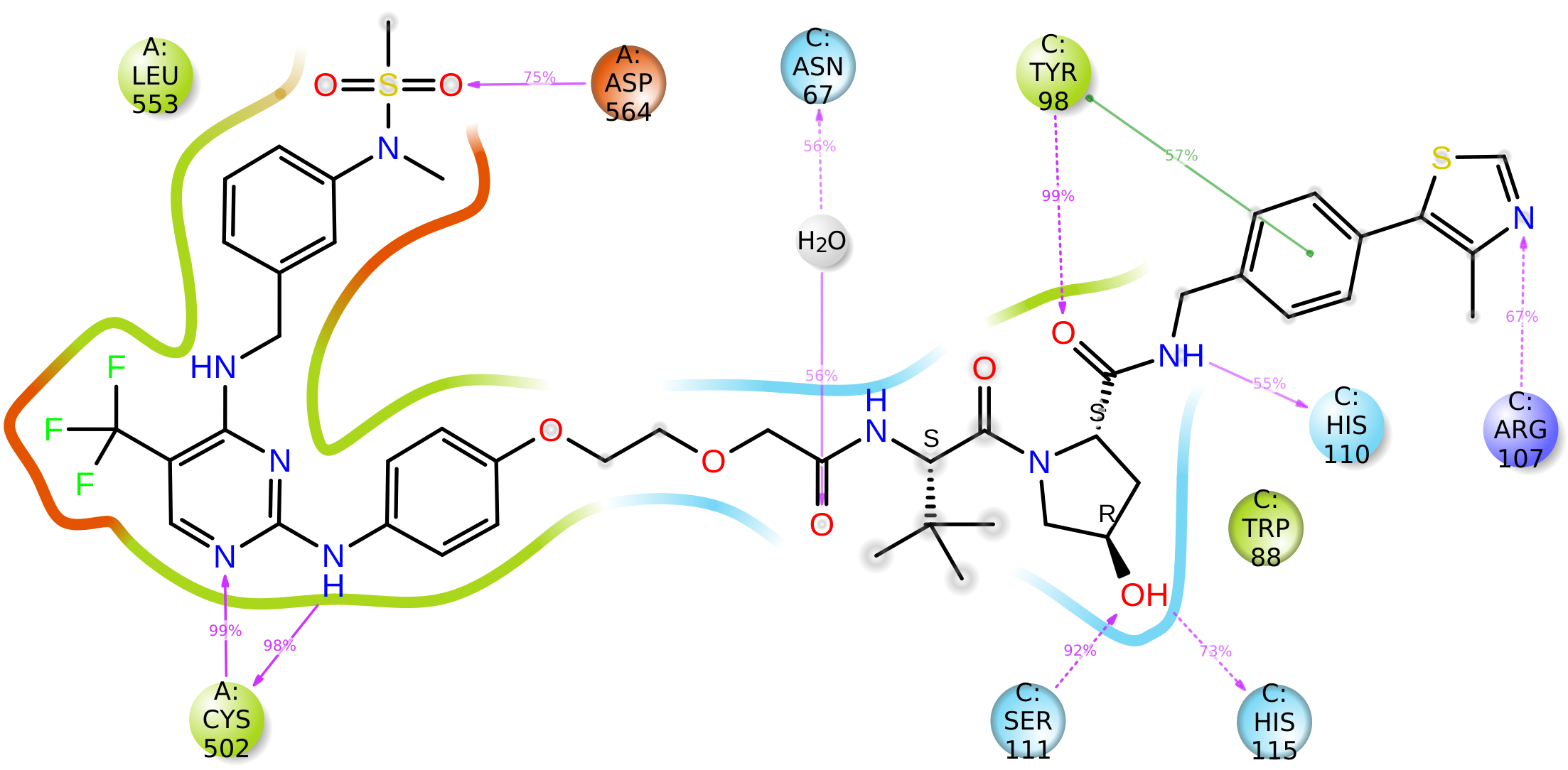 |
| --- | --- |
| 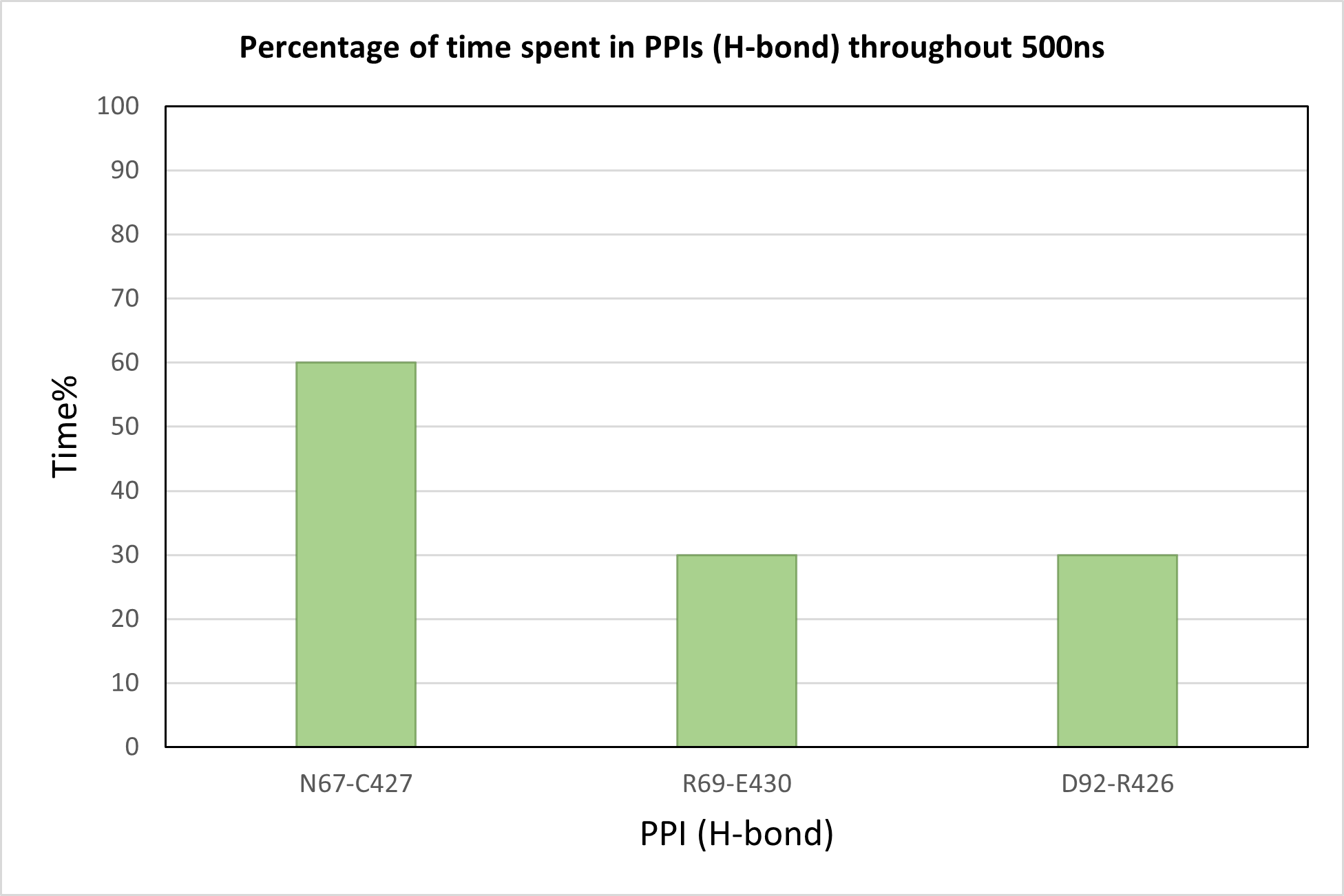 | 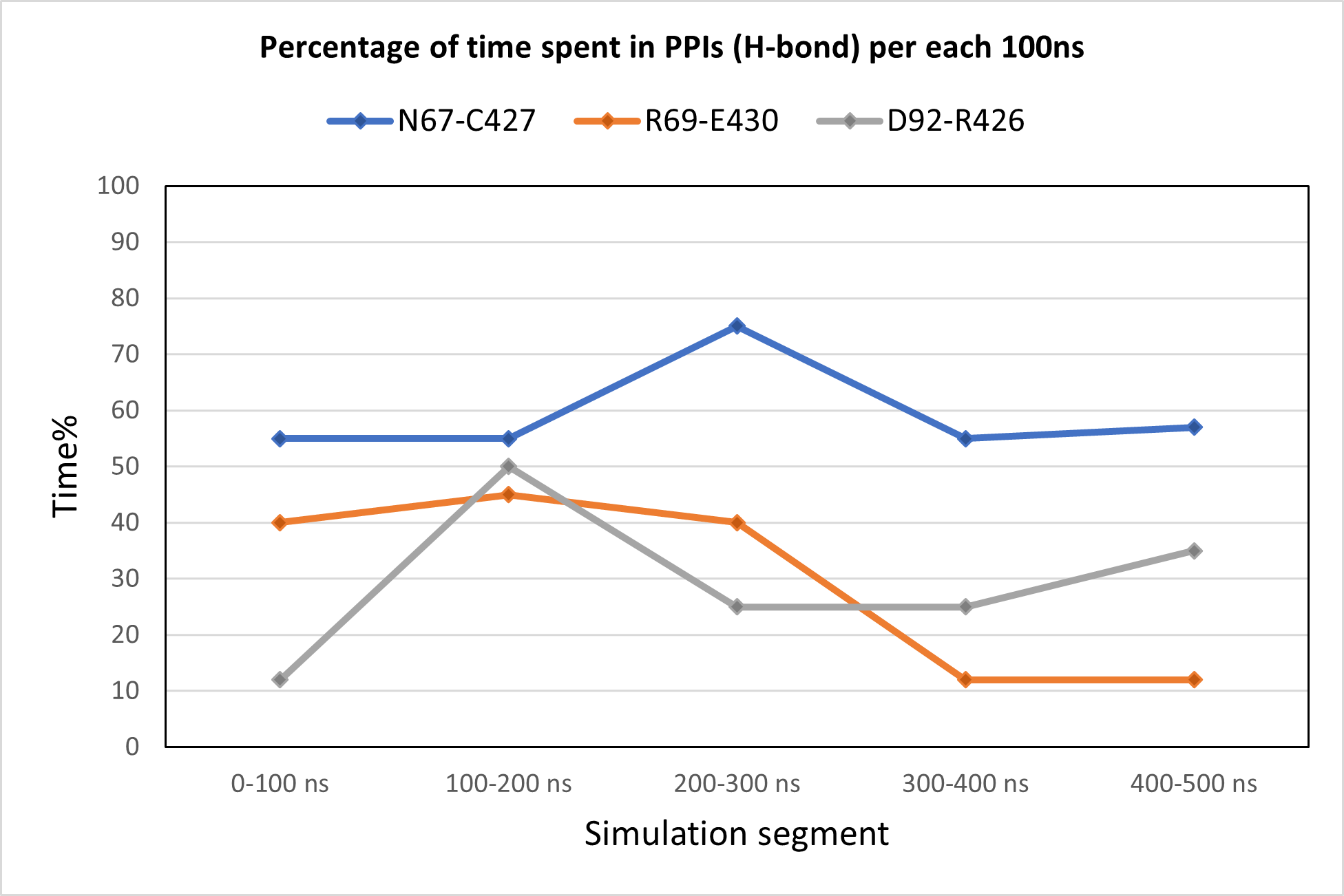 |

**Figure S22:** Analysis of the 500 ns MD simulation of the modeled FAK-PROTAC1-VHL ternary complex obtained via induced fit docking. (A) RMSD values of the protein Cα (orange) and PROTAC1 fitting on the protein Cα (green). (B) Schematic representation of detailed PROTAC1 atom interactions with protein residues. (C) and (D) show the percentage of time spent in PPIs (H-bonds) over the entire 500 ns and in each 100 ns interval, respectively.

(A)

(D)

(C)

| 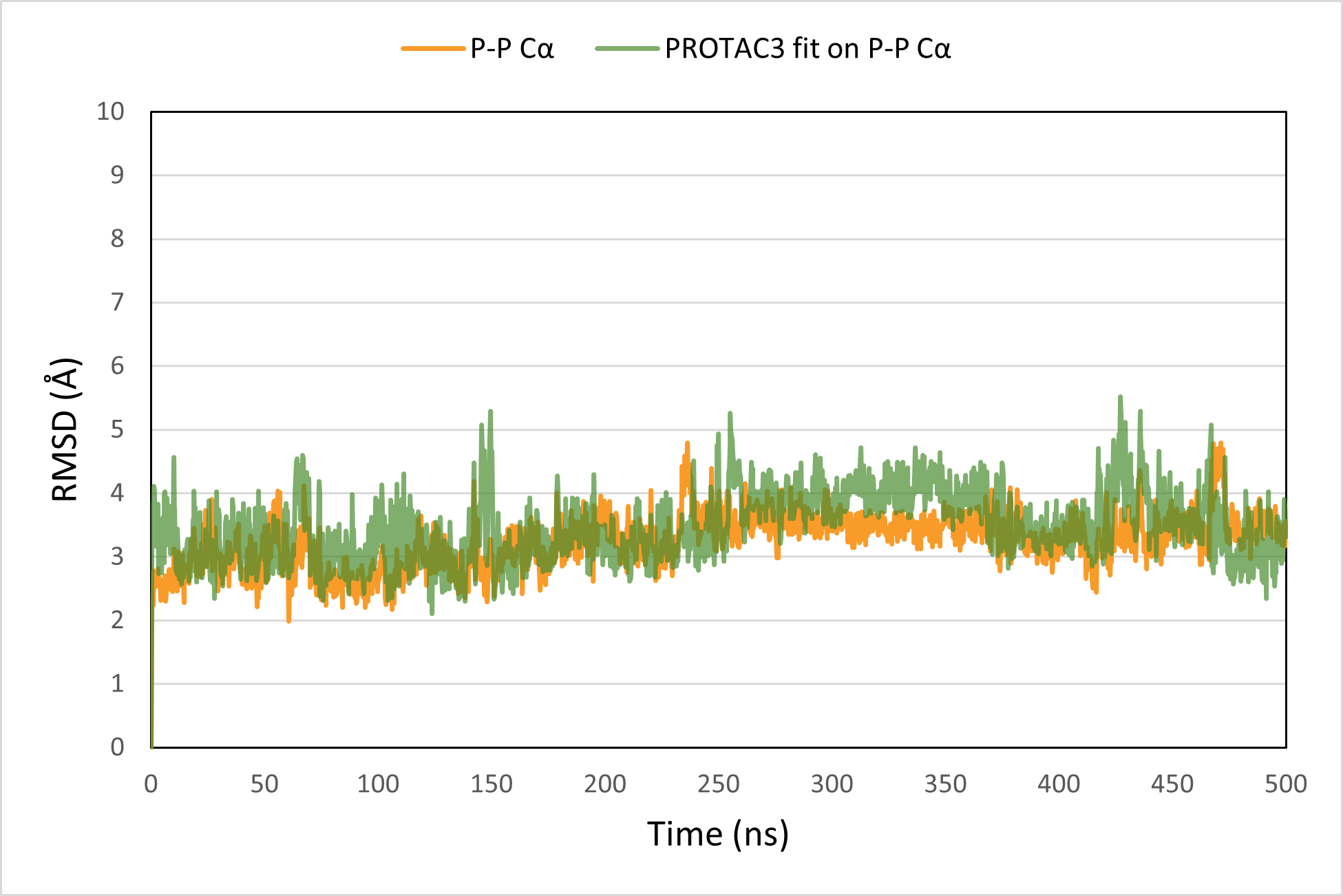 | 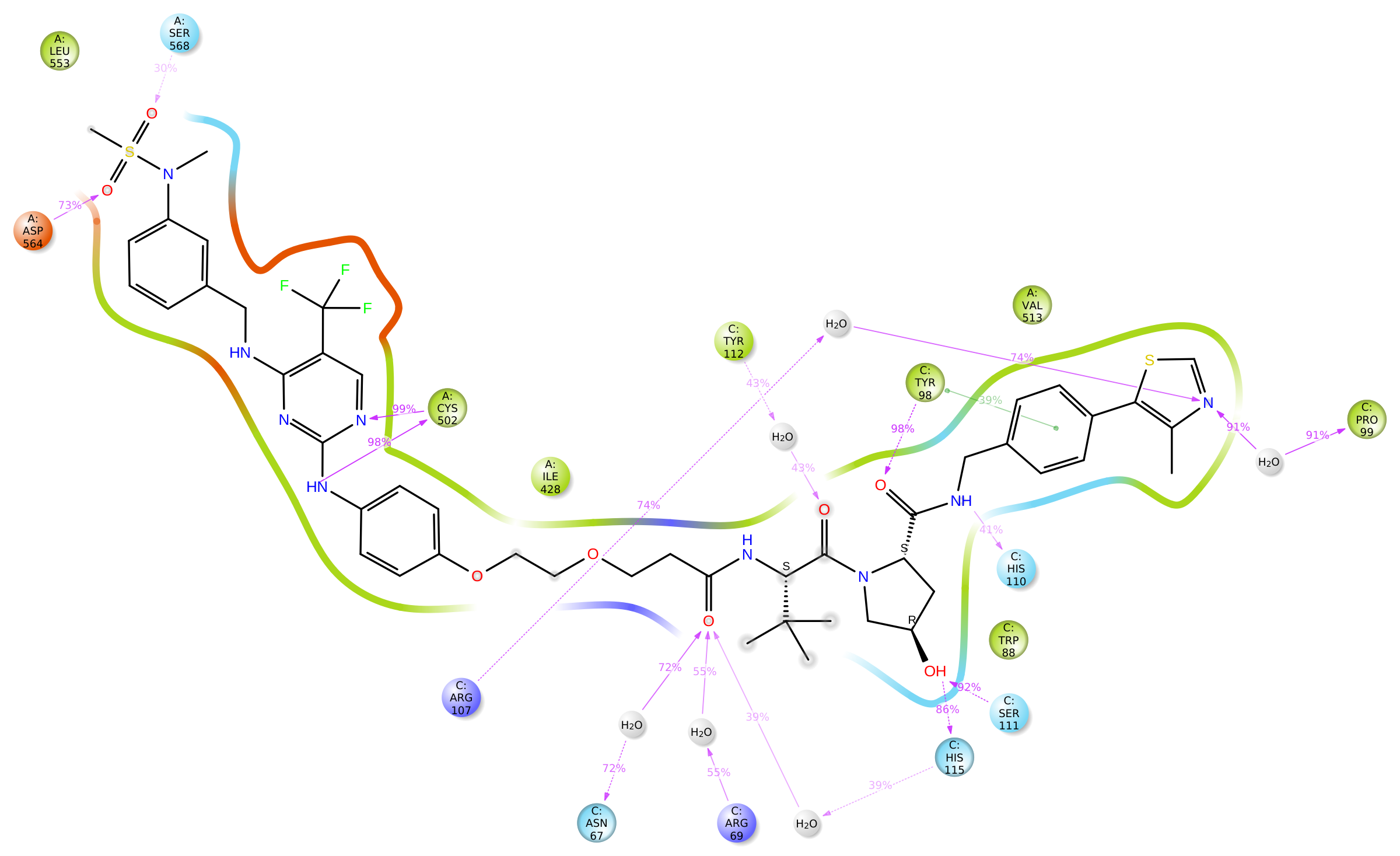 |
| --- | --- |
| 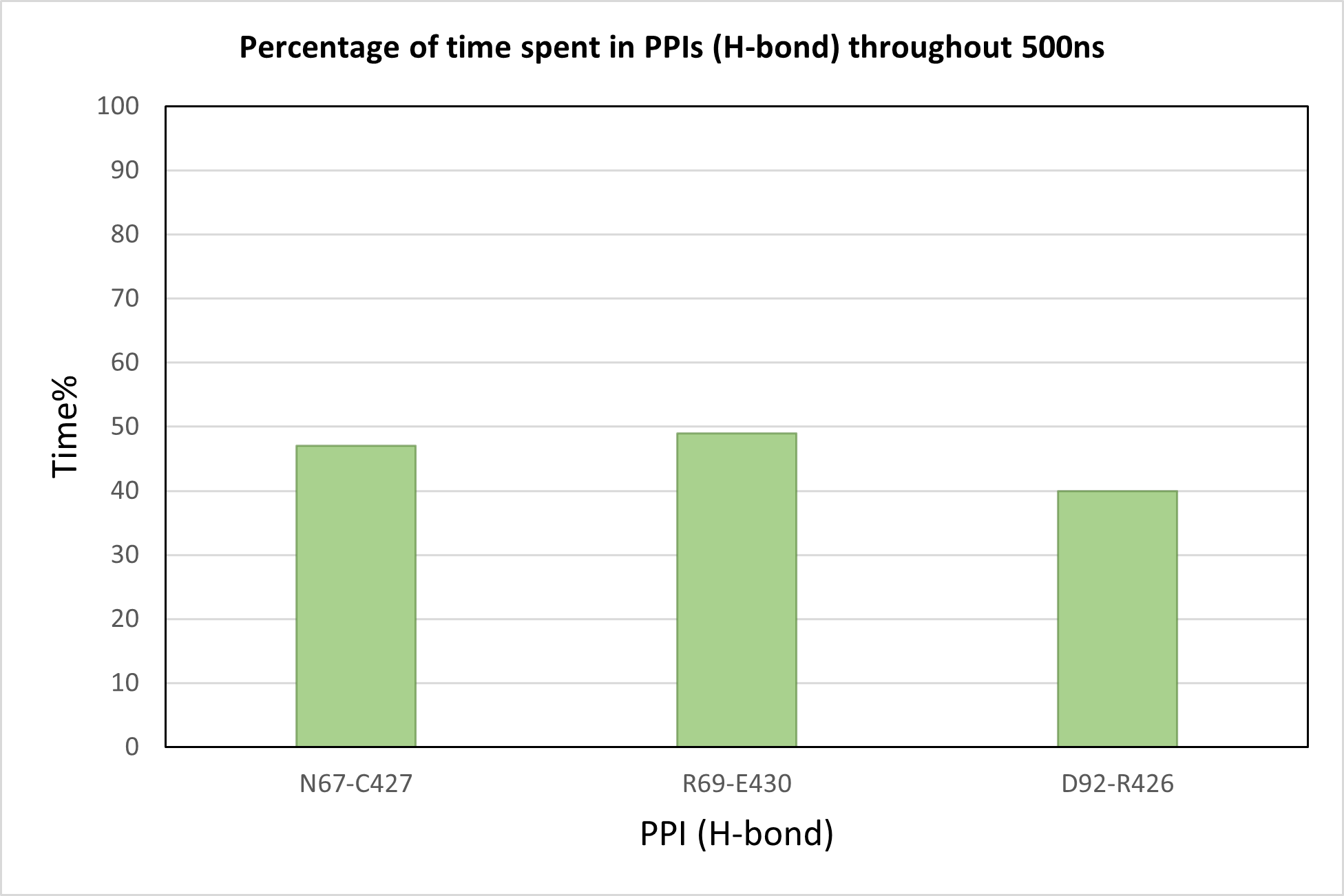 | 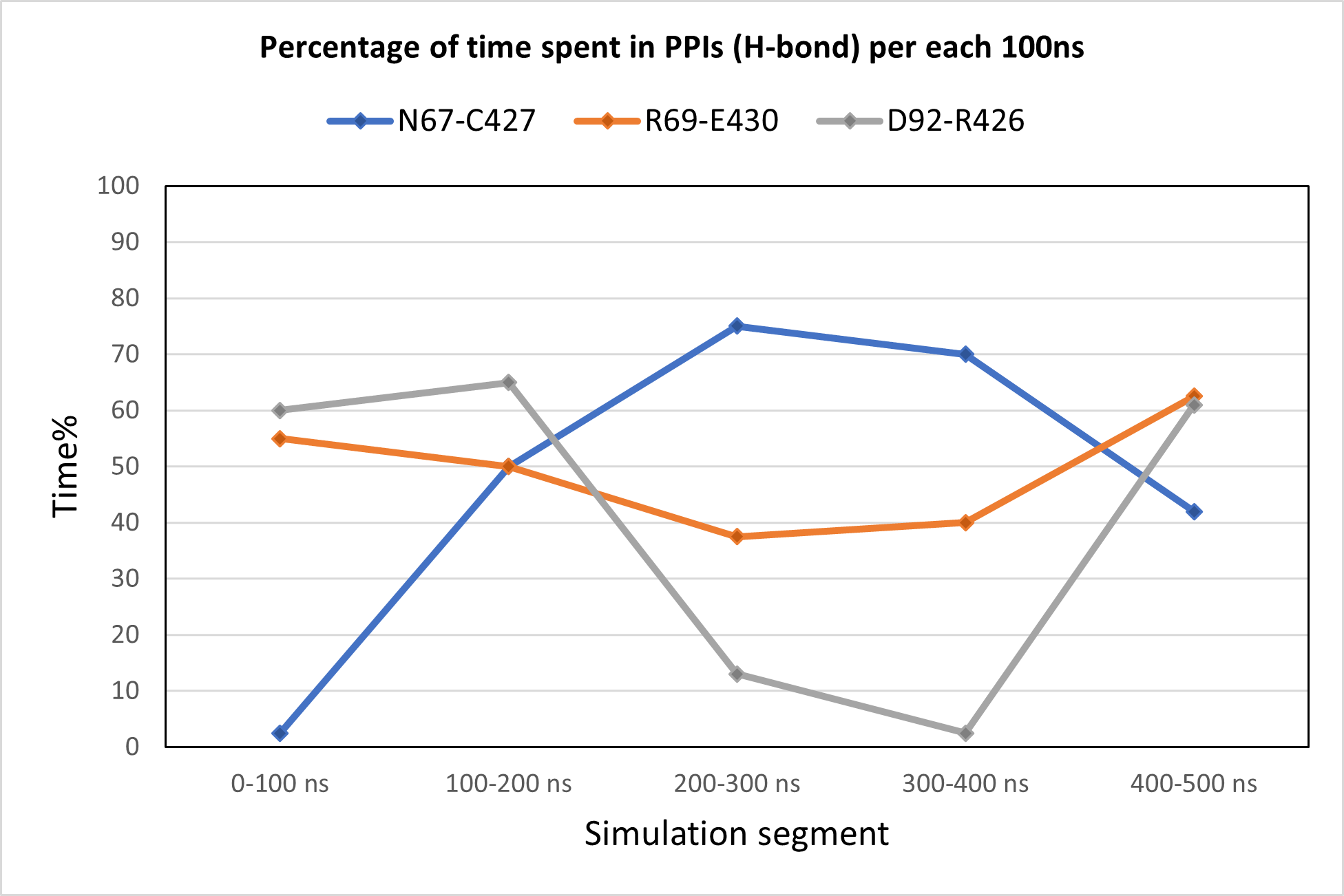 |

**Figure S23:** Analysis of the 500 ns MD simulation of the modeled FAK-PROTAC3-VHL ternary complex obtained via induced fit docking. (A) RMSD values of the protein Cα (orange) and PROTAC3 fitting on the protein Cα (green). (B) Schematic representation of detailed PROTAC3 atom interactions with protein residues. (C) and (D) show the percentage of time spent in PPIs (H-bonds) over the entire 500 ns and in each 100 ns interval, respectively.

(B)

(A)

(D)

(C)

| 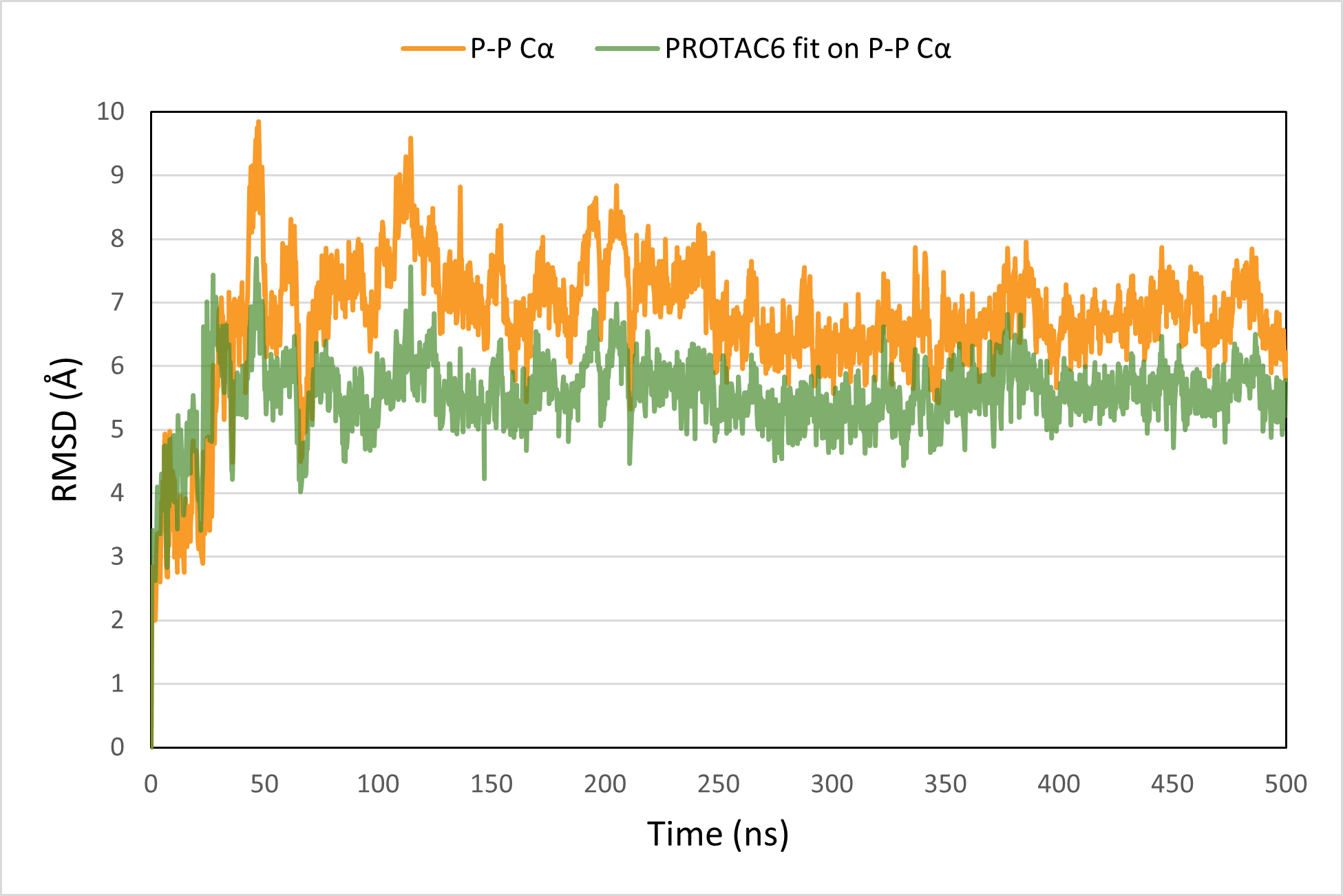 | 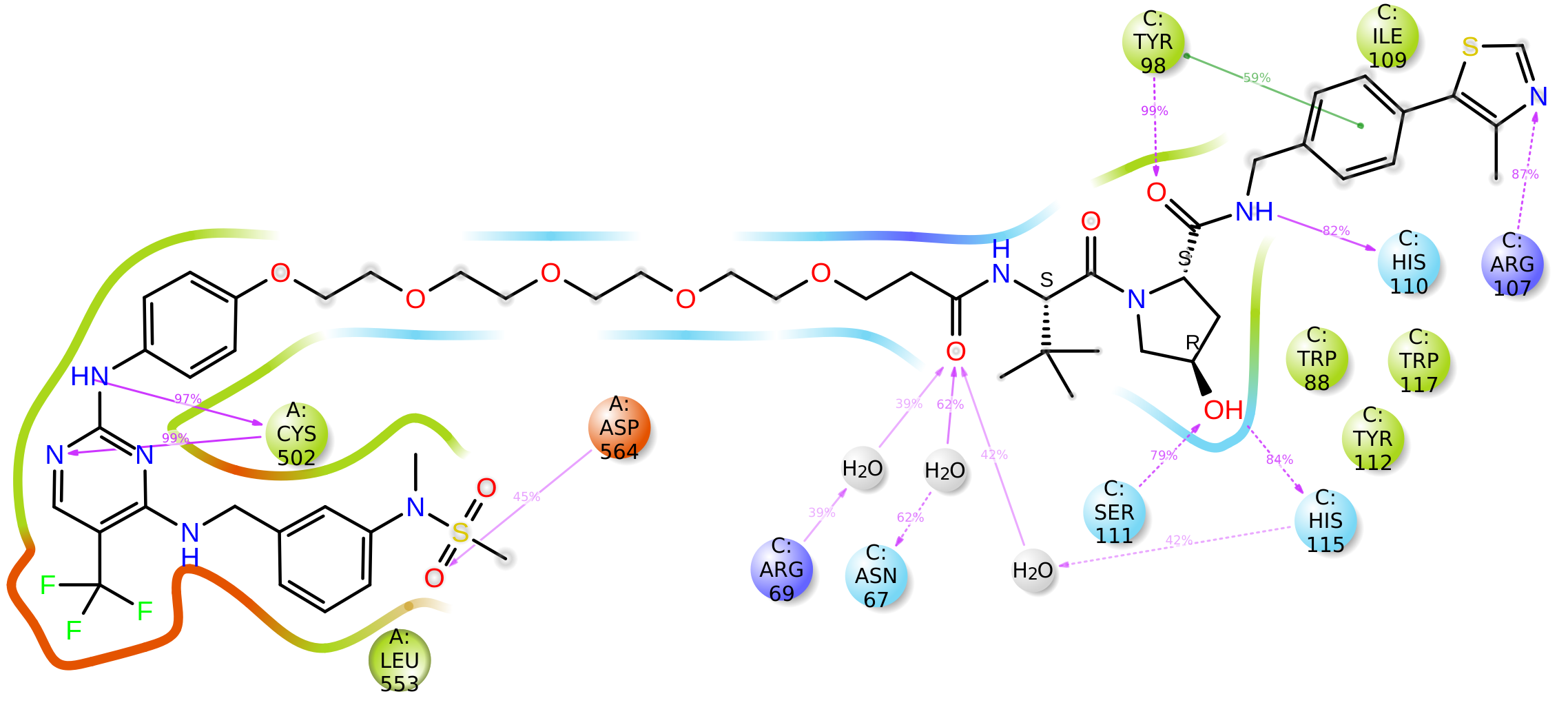 |
| --- | --- |
| 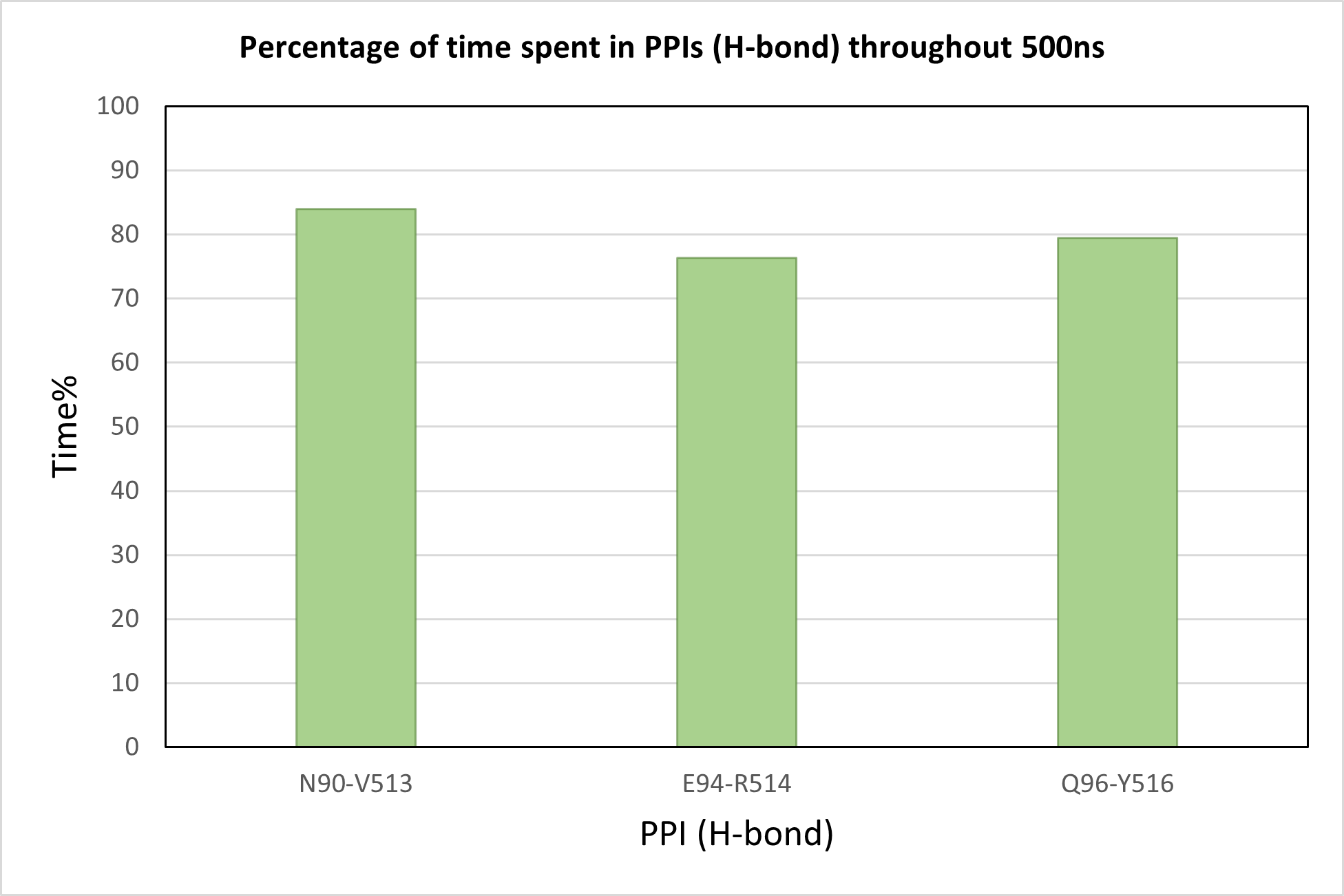 | 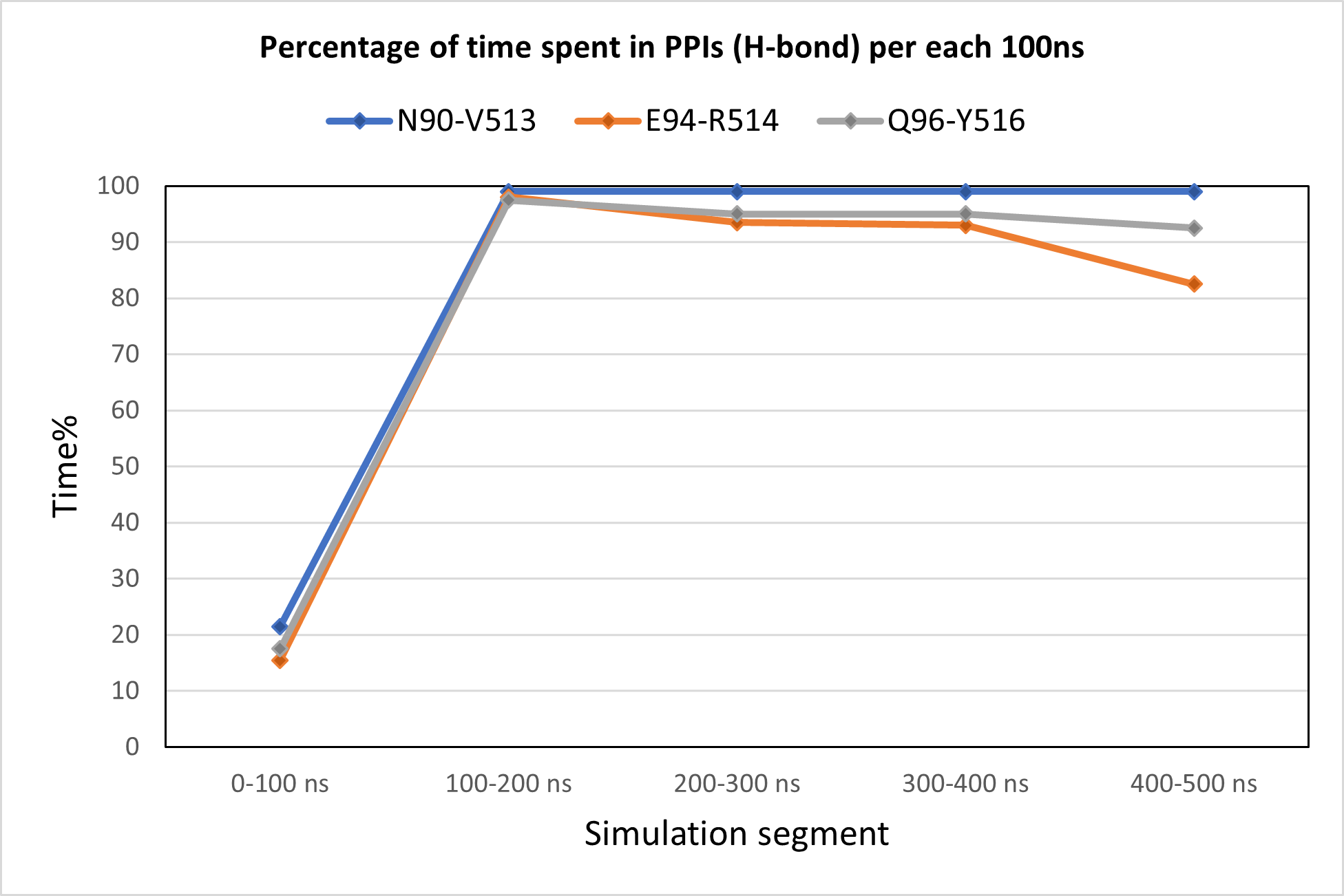 |

**Figure S24:** Analysis of the 500 ns MD simulation of the modeled FAK-PROTAC6-VHL ternary complex obtained via induced fit docking. (A) RMSD values of the protein Cα (orange) and PROTAC6 fitting on the protein Cα (green). (B) Schematic representation of detailed PROTAC6 atom interactions with protein residues. (C) and (D) show the percentage of time spent in PPIs (H-bonds) over the entire 500 ns and in each 100 ns interval, respectively.

(A)

(B)

(C)

(D)

| 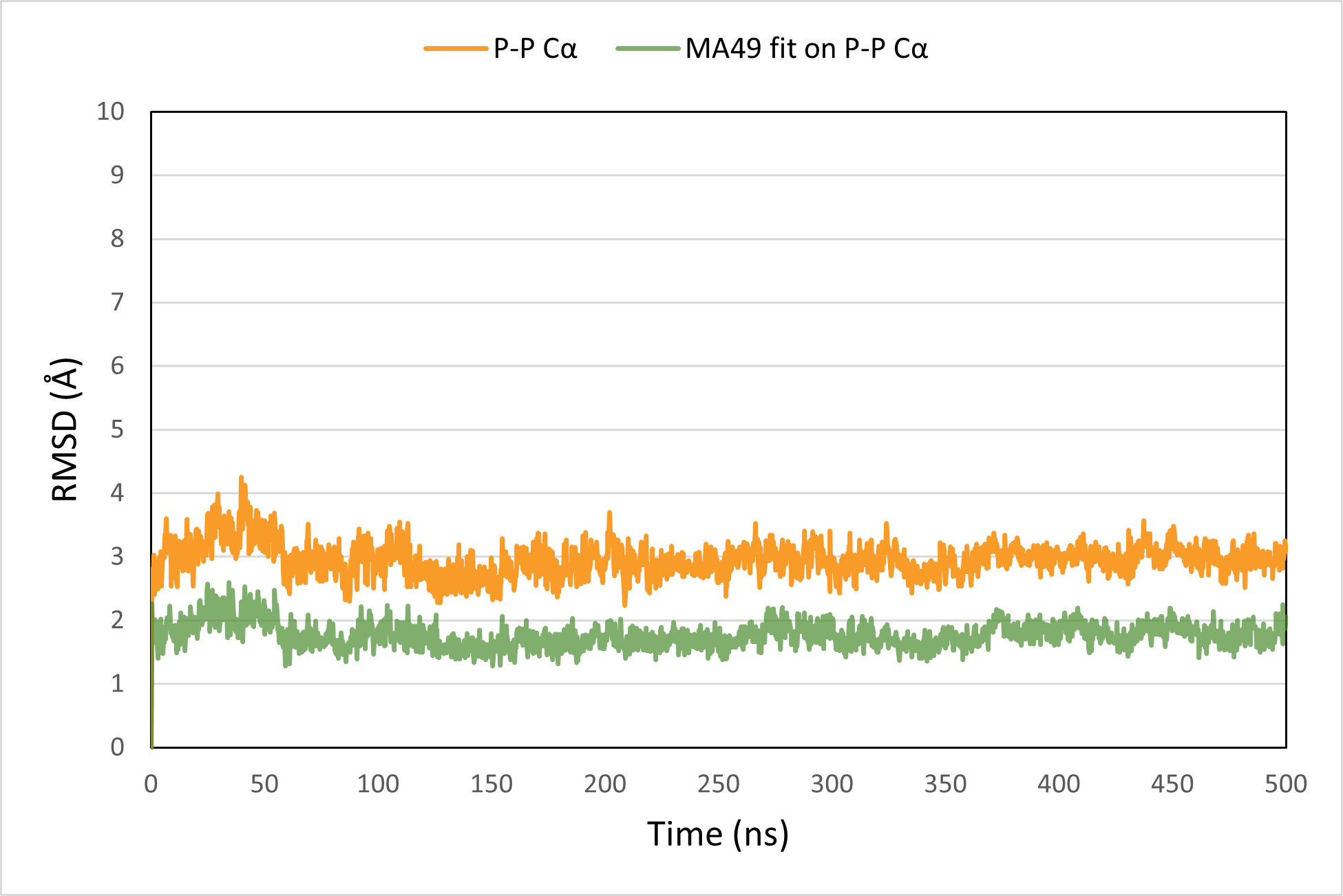 | 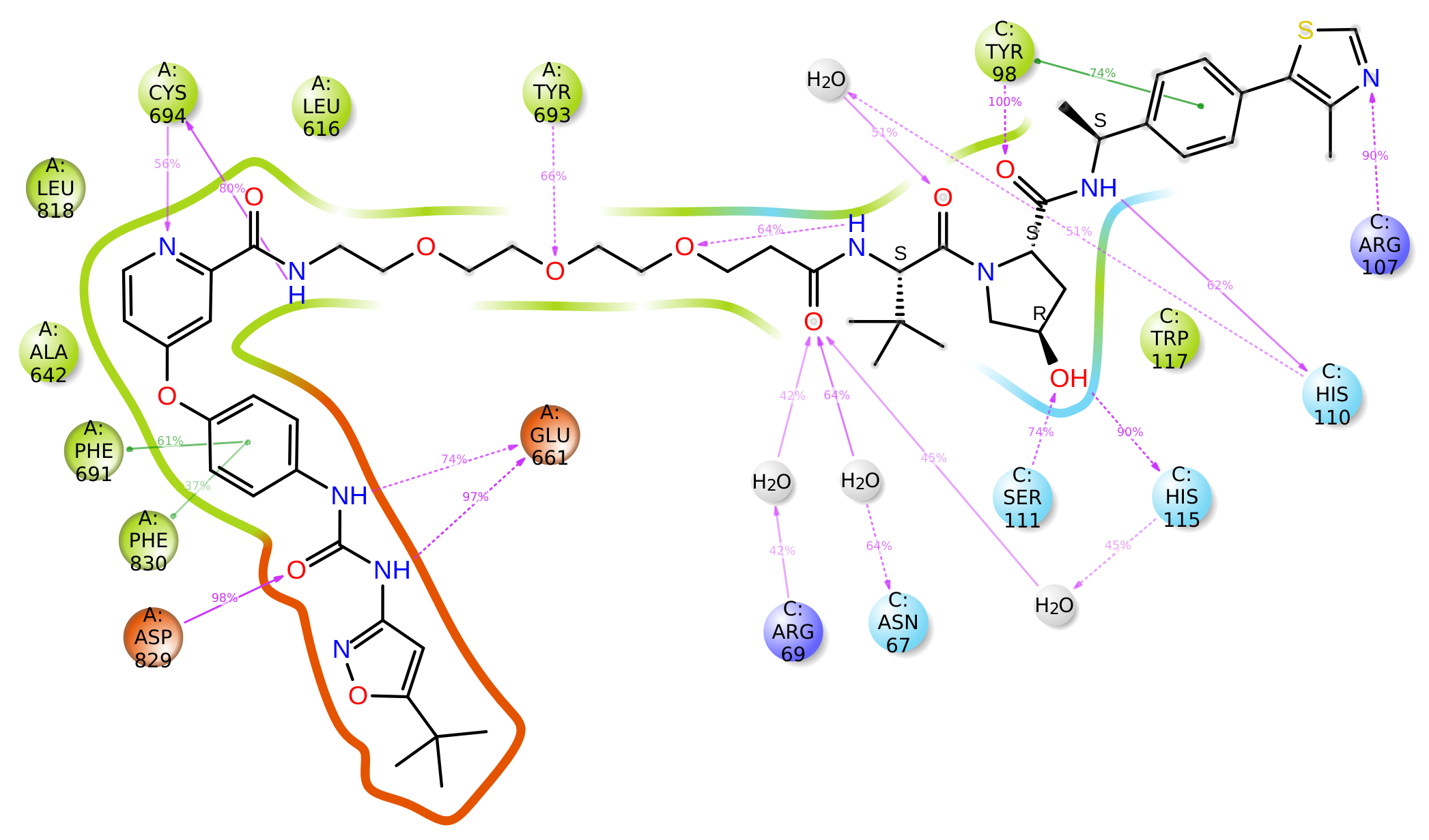 |
| --- | --- |
| 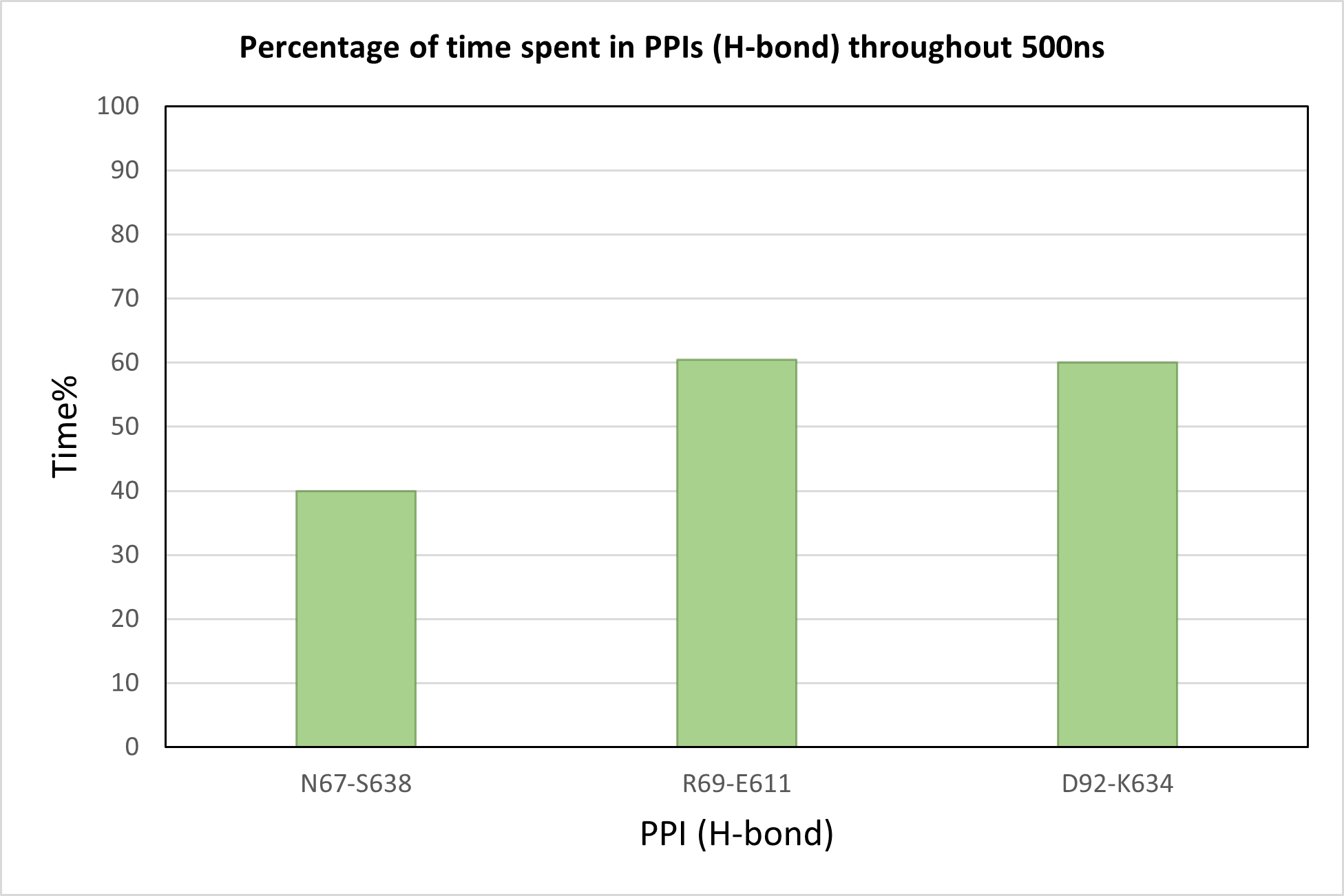 | 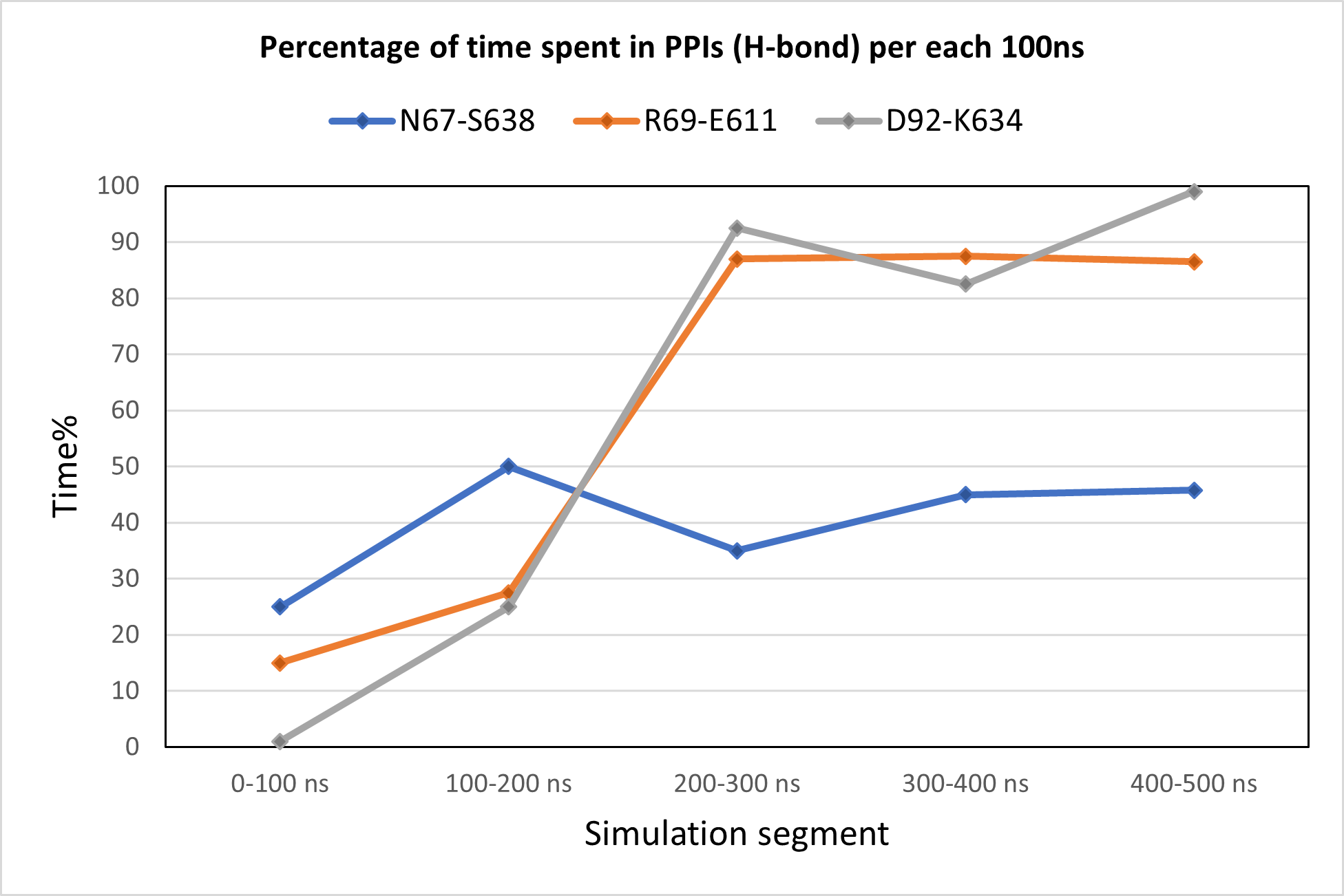 |

**Figure S25:** Analysis of the 500 ns MD simulation of the modeled FLT3-MA49-VHL ternary complex obtained by Method 4B. (A) RMSD values of the protein Cα (orange) and MA49 fitting on the protein Cα (green). (B) Schematic representation of detailed MA49 atom interactions with protein residues. (C) and (D) show the percentage of time spent in PPIs (H-bonds) over the entire 500 ns and in each 100 ns interval, respectively.

(A)

(B)

(C)

(B)

(A)

| 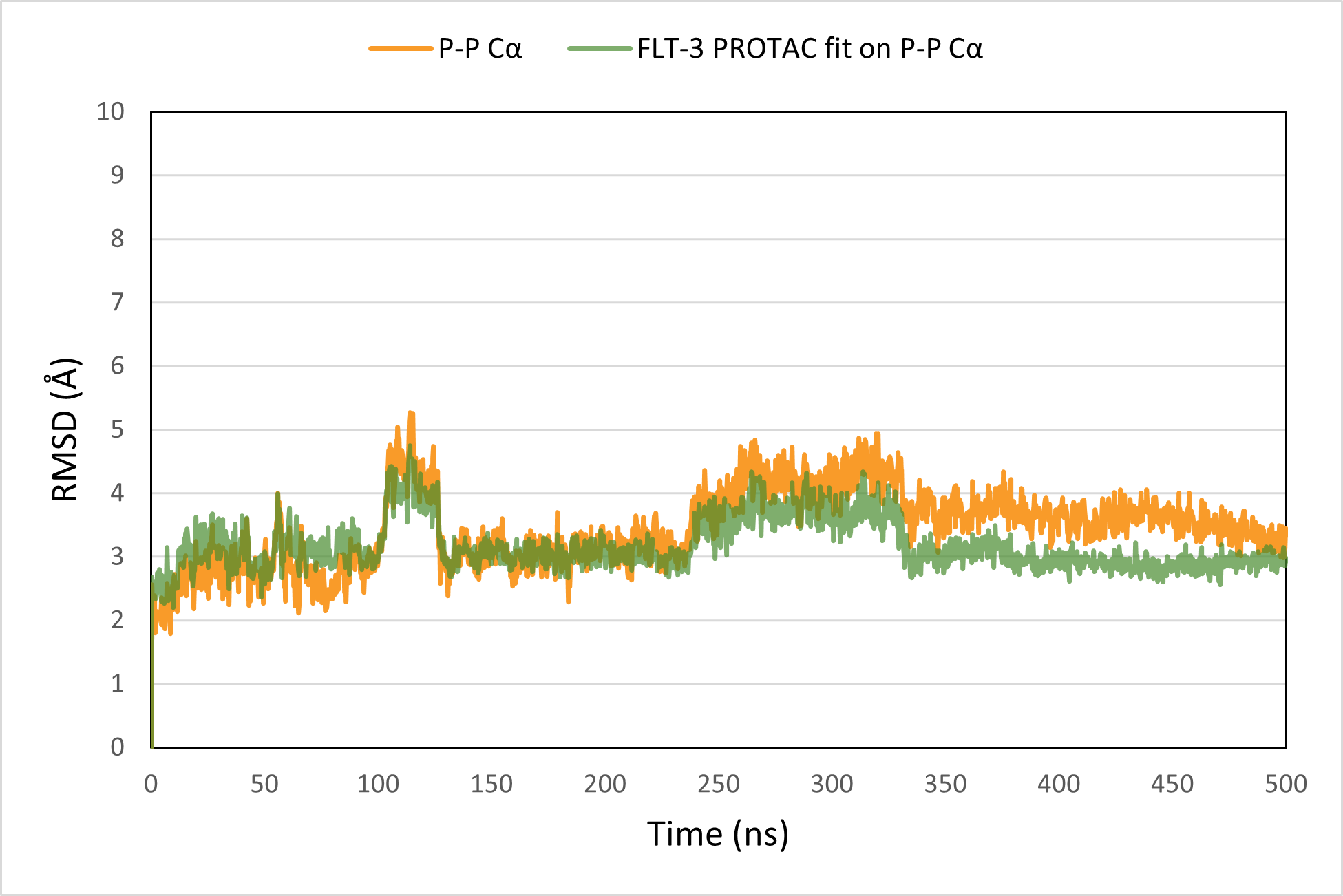 | 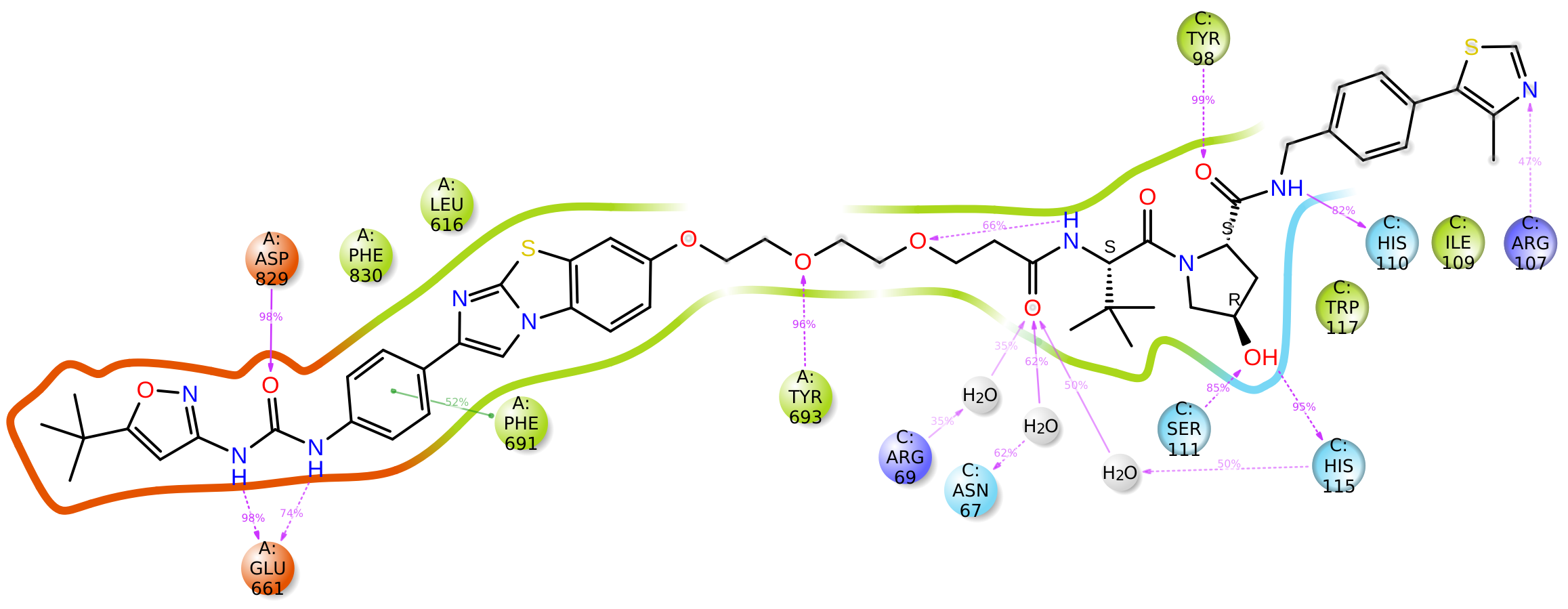 |
| --- | --- |
| 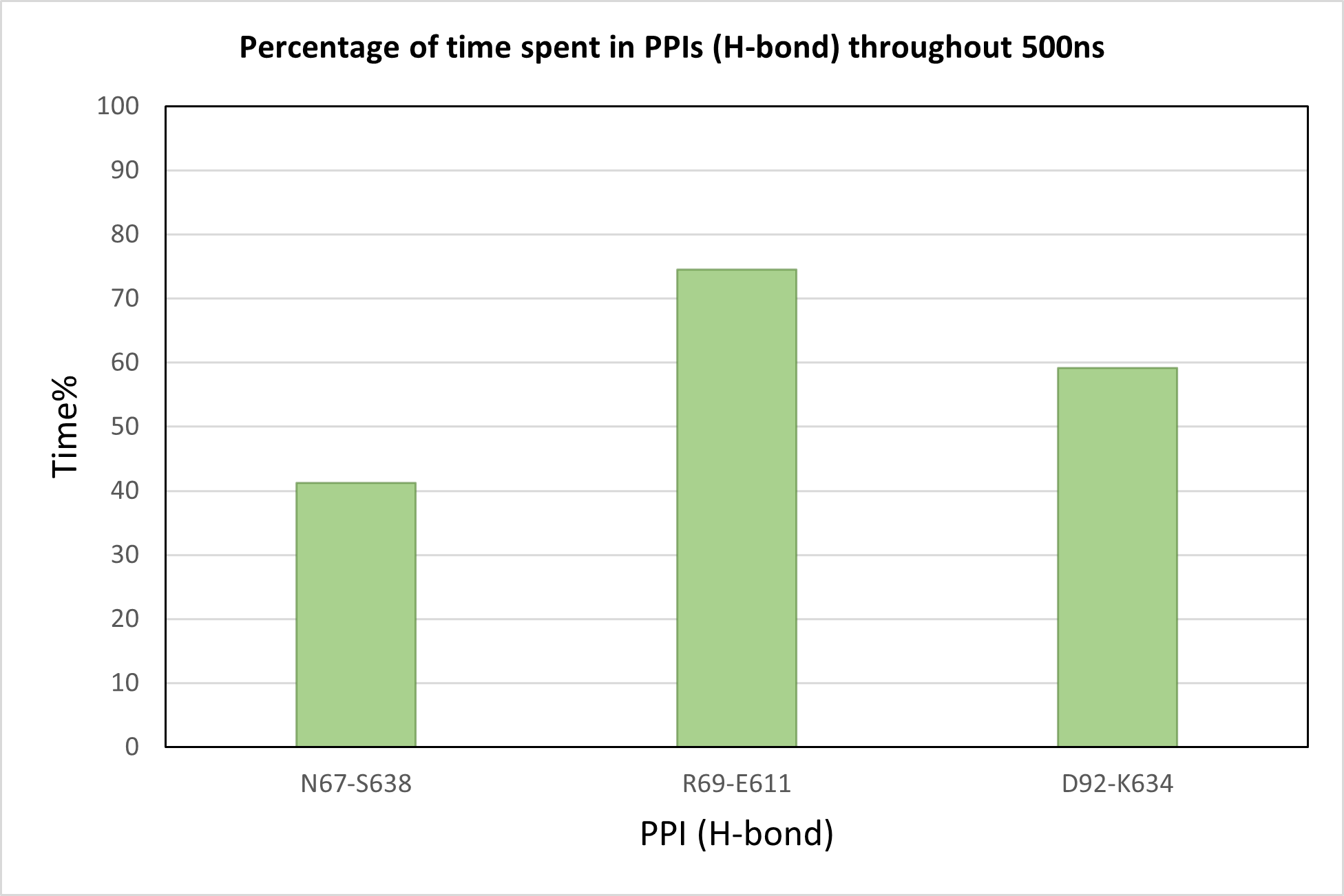 | 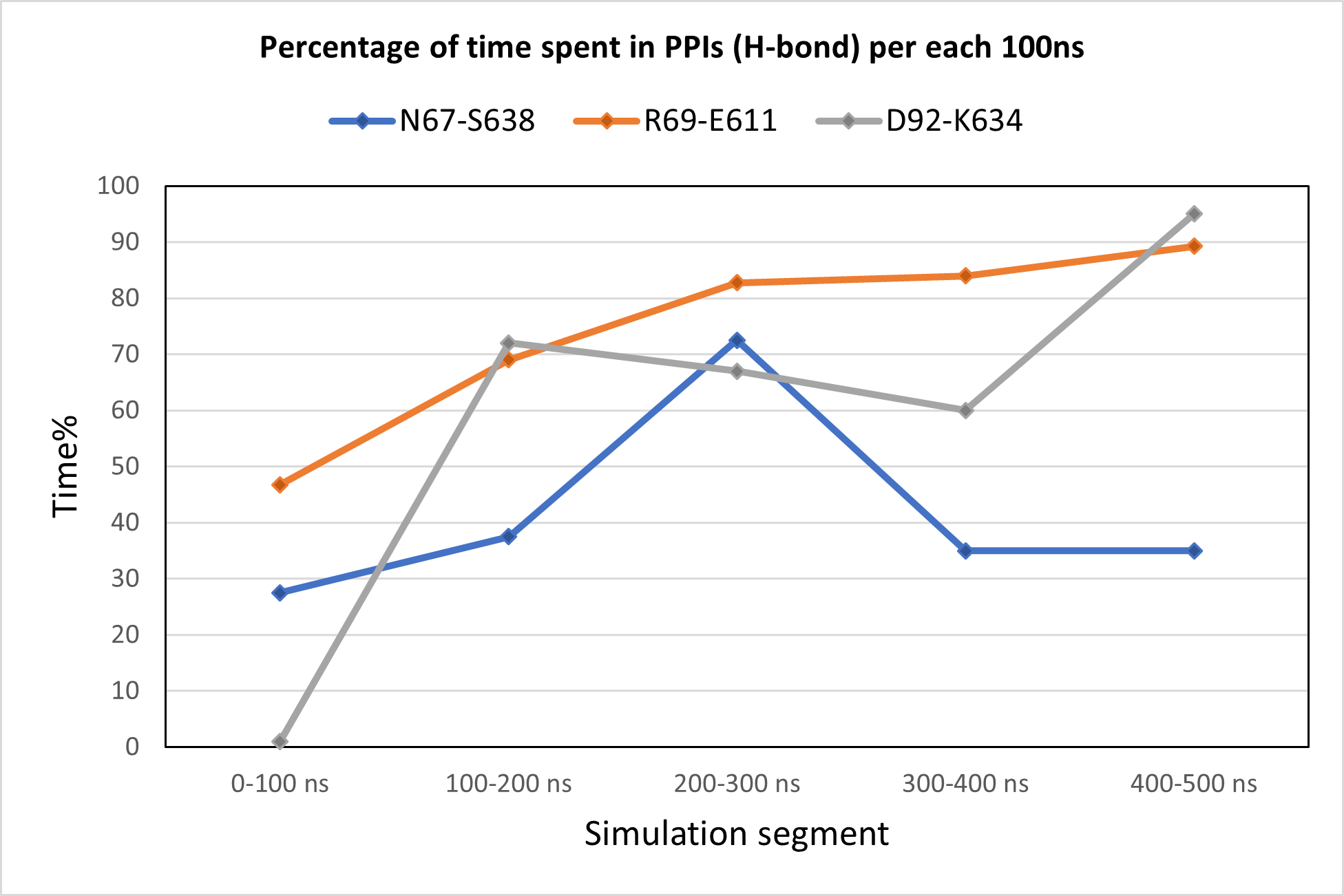 |

**Figure S26:** Analysis of the 500 ns MD simulation of the modeled FLT3 quitartinib-based PROTAC ternary complex obtained bia induced fit docking. (A) RMSD values of the protein Cα (orange) and PROTAC fitting on the protein Cα (green). (B) Schematic representation of detailed PROTAC atom interactions with protein residues. (C) and (D) show the percentage of time spent in PPIs (H-bonds) over the entire 500 ns and in each 100 ns interval, respectively.

(D)

(A)

| 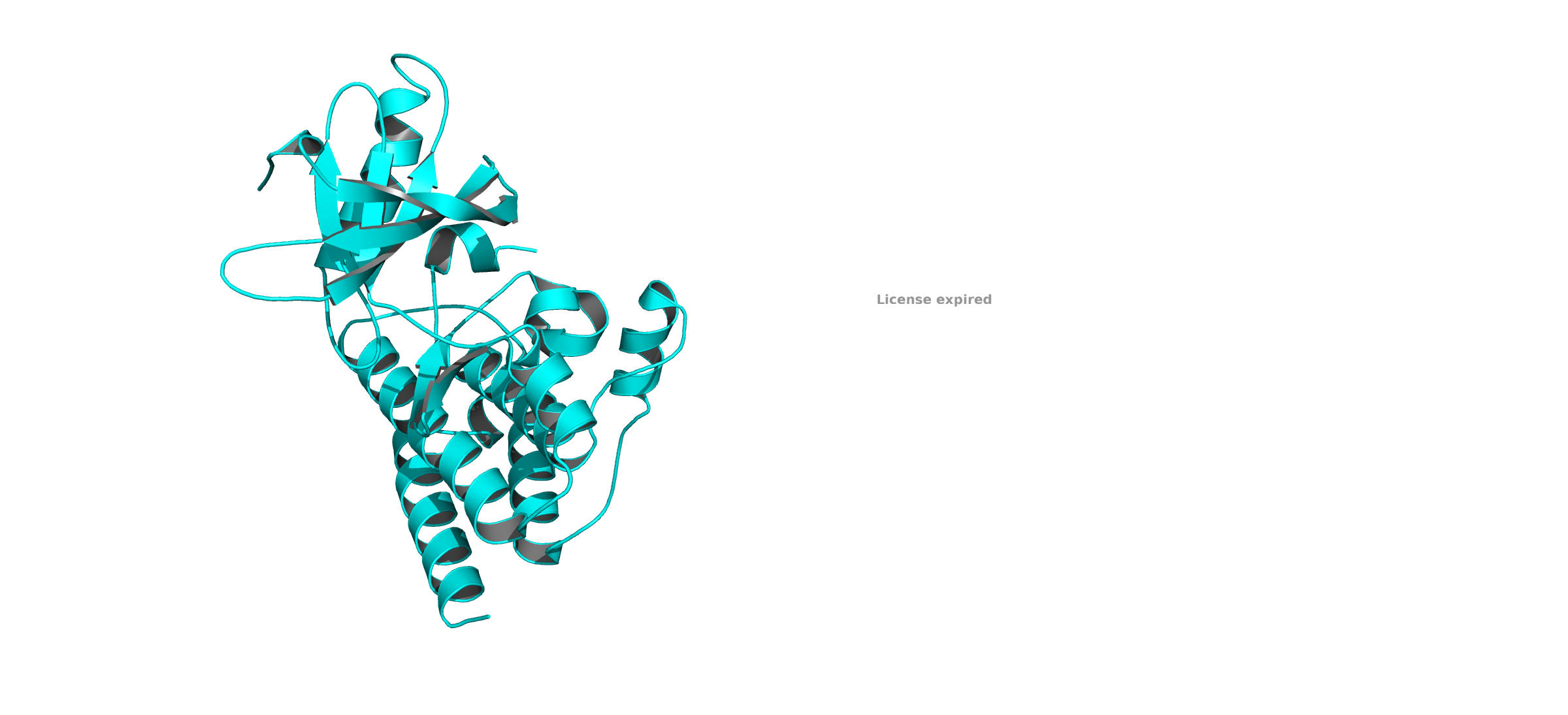 |  |
| --- | --- |

**Figure S27:** Cartoon representation of (A) FAK structure (PDB ID: 7PI4) missing the loop of residues at positions 572 to 582 and (B) FAK structure (PDB ID: 6I8Z) with the loop crystallized and colored orange.

(B)

(A)

SWISS-MODEL

|  |  |
| --- | --- |

**Figure S28:** Cartoon representation of (A) FLT3 structure (PDB ID: 4XUF) missing the loop of residues at positions 708 to 782 and (B) FLT3 structure obtained via SWISS-MODEL with the modelled loop colored orange.

(B)

**Cullin2**

**EC**

**EB**

**VHL**

**Rbx1**

**E2 ligase**

**Ubiquitin**

**FAK**

|  |
| --- |

**Figure S29:** Surface representation of the ubiquitination machinery obtained via aligning the structure of Rbx1-E2-NEDD8-Cul1-Dcn1 (PDB ID: 4P5O) on VHL-EB-EC-Cul2-Rbx1 complex (PDB ID: 5N4W) superposed via the Rbx1 and Cullin subunits. The target protein of the ternary models was added to the modeled ubiquitination machinery by superposing on the VHL subunit.
